# Boundedness and Decay for the Teukolsky Equation on Kerr Spacetimes I: The Case $|a|\ll M$

**DOI:** 10.1007/s40818-018-0058-8

**Published:** 2019-01-08

**Authors:** Mihalis Dafermos, Gustav Holzegel, Igor Rodnianski

**Affiliations:** 1 Department of Pure Mathematics and Mathematical Statistics, University of Cambridge, Wilberforce Road, Cambridge, CB3 0WA UK; 2 Department of Mathematics, Princeton University, Fine Hall, Washington Road, Princeton, NJ 08544 USA; 3 Department of Mathematics, Imperial College London, South Kensington Campus, London, SW7 2AZ UK

**Keywords:** Kerr black hole, Teukolsky equation, General relativity

## Abstract

We prove boundedness and polynomial decay statements for solutions of the spin $\pm \,2$ Teukolsky equation on a Kerr exterior background with parameters satisfying $|a|\ll M$. The bounds are obtained by introducing generalisations of the higher order quantities *P* and ${\underline{P}}$ used in our previous work on the linear stability of Schwarzschild. The existence of these quantities in the Schwarzschild case is related to the transformation theory of Chandrasekhar. In a followup paper, we shall extend this result to the general sub-extremal range of parameters $|a|<M$. As in the Schwarzschild case, these bounds provide the first step in proving the full linear stability of the Kerr metric to gravitational perturbations.

## Introduction

The stability of the celebrated Schwarzschild [100] and Kerr metrics [72] remains one of the most important open problems of classical general relativity and has generated a large number of studies over the years since the pioneering paper of Regge–Wheeler [98]. See [42, 43] and the introduction of [31] for recent surveys of the problem.

The ultimate question is that of *nonlinear* stability, that is to say, the dynamic stability of the Kerr family $({\mathcal {M}},g_{a,M})$ (including the Schwarzschild case $a=0$), without symmetry assumptions, as solutions to the Einstein vacuum equations

1 $$\begin{aligned} {\mathrm{Ric}}[g]=0, \end{aligned}$$

in analogy to the nonlinear stability of Minkowski space, first proven in the monumental [26]. A necessary step to understand nonlinear stability is of course proving suitable versions of *linear stability*, i.e. boundedness and decay statements for the linearisation of (1) around the Schwarzschild and Kerr solutions. This requires first imposing a gauge in which the equations (1) become well-posed. A complete study of the linear stability of Schwarzschild in a double null gauge has been obtained in our recent [31]. A key step in [31] was proving boundedness and decay for the so-called *Teukolsky equation*, to be discussed below in Sect. 1.1, which can be thought to suitably control the “gauge invariant” part of the perturbations. See already equation (2). These decay results were then used in [31] to recover appropriate estimates for the full linearisation of (1).

The purpose of the present paper is to extend the boundedness and decay results of [31] concerning the Teukolsky equation (2) from the Schwarzschild $a=0$ case to the very slowly rotating Kerr case, corresponding to parameters $|a|\ll M$. We give a rough statement of the main result already in Sect. 1.2 below.

In part II of this series, we shall obtain an analogue of our main theorem for the case of general subextremal Kerr parameters $|a|<M$. The extremal case $|a|=M$ is exceptional; see Sect. 1.3 for remarks on this and other related problems. In a separate paper, following our previous work on Schwarzschild [31], we will use the above result to show the full linear stability of the Kerr solution in an appropriate gauge.

We end this introduction in Sect. 1.4 with an outline of the paper.

### The Teukolsky Equation for General Spin

The original approach to linear stability in the Schwarzschild case centred on so-called metric perturbations, leading to the decoupled equations of Regge–Wheeler [98] and Zerilli [113]. The Regge–Wheeler equation will in fact appear below as formula (7). This approach does not, however, appear to easily generalise to Kerr. Thus, it was a fundamental advance when Teukolsky [107] identified two gauge invariant quantities which decouple from the full linearisation of (1) in the general Kerr case. The quantities, corresponding to the extremal curvature components in the Newman–Penrose formalism [93], can each be expressed by complex scalars $\upalpha ^{[\pm 2]}$ which satisfy a wave equation, now known as the Teukolsky equation:

2 $$\begin{aligned}&\Box _g \upalpha ^{[s]} +\frac{2s}{\rho ^2}(r-M)\partial _r \upalpha ^{[s]} +\frac{2s}{\rho ^2} \left( \frac{a(r-M)}{\Delta } +i\frac{\cos \theta }{\sin ^2\theta }\right) \partial _\phi \upalpha ^{[s]}\nonumber \\&\quad +\frac{2s}{\rho ^2}\left( \frac{M(r^2-a^2)}{\Delta }-r-ia\cos \theta \right) \partial _t \upalpha ^{[s]}\nonumber \\&\quad +\frac{1}{\rho ^2}(s-s^2\cot ^2\theta ) \upalpha ^{[s]}=0, \end{aligned}$$

with $s=+\,2$ and $-\,2$ respectively. The scalars are more properly thought of as spin $\pm \,2$ weighted quantities. This generalised an analogous property in the Schwarzschild case identified by Bardeen and Press [14]. These quantities govern the “gauge invariant” part of the perturbations in the sense that an admissible solution of the linearised Einstein equations whose corresponding $\upalpha ^{[\pm 2]}$ both vanish must be a combination of a linearised Kerr solution and a pure gauge solution [110].

Note that equation (2) can be considered for arbitrary values of $s\in \frac{1}{2}{\mathbb {Z}}$. For $s=0$, (2) reduces to the covariant wave equation $\Box _g\psi =0$, while for $s=\pm \, 1$, (2) arises as an equation satisfied by the extreme components of the Maxwell equations in a null frame [22].

#### Separability and the Mode Stability of Whiting and Shlapentokh-Rothman

An additional remarkable property of the Teukolsky equation (2) is that it can be formally separated, in analogy with Carter’s separation [19] of the wave equation (i.e. the case of $s=0$). The separation of the $\theta$-dependence is surprising in the case $a\ne 0$ for all *s* because the Kerr metric only admits $\partial _\phi$ and $\partial _t$ as Killing fields. It turns out that considering the ansatz

3 $$\begin{aligned} \upalpha ^{[s]} (r) e^{-i\omega t} S_{m\ell }^{[s]}(a\omega , \cos \theta ) e^{im\phi } \end{aligned}$$

where $S_{m\ell }^{[s]}(\nu , \cos \theta )$ denote spin-weighted oblate spheroidal harmonics, one can derive from (2) an ordinary differential equation for $\upalpha$, which in rescaled form (see (151)) can be written as

4 $$\begin{aligned} u'' + V^{[s]}(\omega , m, \ell , r) u = 0 \end{aligned}$$

where for $s\ne 0$, the potential $V^{[s]}$ is complex valued. (Here $'$ denotes differentiation with respect to $r^*$. See Sect. 2.1.) See already (153). The separation (3) was subsequently understood to be related to the presence of an additional Killing tensor [73].

Of course, the problem of decomposing general, initially finite-energy solutions of (2) as appropriate superpositions of (3) is intimately tied with the validity of boundedness and decay results, in view of the necessity of taking the Fourier transform in time. A preliminary question that can be addressed already solely at the level of (4) is that of “mode stability”. Mode stability is the statement that there are no initially finite-energy solutions of the form (3) with $\mathrm{Im}(\omega )>0$. This reduces to showing the non-existence of solutions of (4) with $\mathrm{Im}(\omega )>0$ and exponentially decaying boundary conditions both as $r^*\rightarrow \infty$ and $r^*\rightarrow -\infty$.

In the case $a=0$, $s=0$, then mode stability can be immediately inferred by applying the physical space energy estimate associated to the Killing vector field $\partial _t$ to a solution of the form (3). The question is highly nontrivial for $a\ne 0$, already in the case $s=0$, in view of the phenomenon of *superradiance*, connected to the presence of the so-called *ergoregion* where $\partial _t$ is spacelike. For $s=\pm \, 2$, the question is non-trivial even in the case $a=0$, as there does not exist an obvious conserved energy current. (In separated form (4), this is related to the fact that the potential $V^{[s]}$ is now complex valued.) In a remarkable paper, Whiting [111] nonetheless succeeded in proving mode stability for (2) for all *s* in the general subextremal range of parameters $|a|<M$ by cleverly exploiting certain algebraic transformations of the ode (4).

Mode stability has been extended to exclude “resonances” on the real axis, i.e. solutions *u* of (4) with $\omega \in {\mathbb {R}}$ with appropriate boundary conditions, by Shlapentokh-Rothman [104] in the case $s=0$, who had the insight that the transformations applied in [111] could be extended to the real axis using the theory of oscillatory integrals. Together with a continuity argument in *a*, [104] can be used to reprove the original [111], and this leads to certain simplifications. The argument generalises to $s=\pm \,2$. See also [6] where the techniques of [104] are combined with an alternative complex analytic treatment.

We emphasise that mode stability is a remarkable property tied to the specific form of the equation (2) and to the specific Kerr background, even for $s=0$. Indeed, mode stability fails for $a\ne 0$ when an arbitrarily small Klein–Gordon mass is added, as was first suggested by [28, 112] and proven recently in [103]. Even more surprisingly, mode stability fails when a well-chosen positive compactly supported potential is added to (2), or when the Kerr metric is itself sufficiently deformed, keeping however all its symmetries and separation properties, in a spatially compact region which can be taken arbitrarily far from the ergoregion [89].

#### Previous Work on Boundedness and Decay

The quantitative study of the Cauchy problem for (2) with $s=0$, beyond statements for fixed modes, has become an active field in recent years. The study for higher spin is still less developed beyond the Schwarzschild case. We review some relevant previous work below.

*The case*$s=0$, $|a|<M$. An early result [75] obtained boundedness for solutions to the Cauchy problem for the scalar wave equation on Schwarzschild (i.e. the case $s=0$ and $a=0$ of (2)) with regular, localised initial data. Even this involved non-trivial considerations on the event horizon, which can now be understood in a more robust way using the red-shift energy identity [39, 43]. Following intense activity in the last decade (e.g. [3, 16, 18, 36, 39–41, 43, 108]) there are now complete boundedness and decay results for (2) with $s=0$ in the full subextremal range of Kerr parameters $|a|<M$ [45].

The main difficulties in passing from $a=0$ to $a\ne 0$ arise from superradiance, mentioned already in the context of mode stability, and the fact that trapped null geodesics no longer approach a unique value of *r* in physical space. The latter is relevant because integrated local energy decay estimates, an important step in the proof of quantitative decay, must necessarily degenerate at trapping.^1^ One way of dealing with the latter difficulty is employing the separation (3) as a method of frequency localising integrated local energy decay estimates. See [40, 43]. Once such an estimate is obtained, the difficulty of superradiance can easily be overcome in the $|a|\ll M$ case as the error terms in the ergoregion are small and can be absorbed. For alternative approaches, see [3, 108].

The $|a|<M$ case appears a priori to be much more complicated. It turns out, however, that the Schwarzschild-like structure of trapping survives, when appropriately viewed in phase space. Moreover, in the high frequency regime, one can quantify superradiance with the help of the fact that, quite fortuitously, superradiant frequencies happen not to be trapped. See [45]. These good high frequency properties, together with Shlapentokh-Rothman’s real mode stability [104] and a continuity argument in *a*, allow one to extend the exact same boundedness and integrated local energy decay results originally obtained on Schwarzschild to the whole sub-extremal range $|a|<M$ of Kerr parameters. Suitable polynomial decay then follows from an application of the method of $r^p$ weighted energy estimates [38, 87]. See [45]. For comments on the extremal case $|a|=M$, see Sect. 1.3.4.

**The case** $s=\pm \,2$, $a=0$. As we remarked already above, the Teukolsky equation with $s=\pm \, 2$, $a=0$ has been studied in our previous [31] as part of our complete study of the linear stability of Schwarzschild.

The main difficulty of the $s=\pm \,2$ case as opposed to the case $s=0$, is that, as discussed already in the context of mode stability, there does not exist an obvious analogue of the conserved energy associated to the Killing field $\partial _t$. Thus, proving even just boundedness for $a=0$ is non-trivial, even just far away from the event horizon. The key to understanding (2) for $s=\pm \,2$, $a=0$ in [31] was associating quantities $P^{[\pm 2]}$ to $\upalpha ^{[\pm 2]}$ satisfying (2). These are physical space versions of transformations first considered by Chandrasekhar [22] and are defined by the expressions^2^

5 $$\begin{aligned} P^{[+2]}&= -\frac{1}{2(r-2M)} {\underline{L}} \left( \frac{r^3}{r-2M} {\underline{L}} \left( \frac{(r-2M)^2}{r} \upalpha ^{[+2]}\right) \right) \, , \end{aligned}$$

6 $$\begin{aligned} P^{[-2]}&=-\frac{1}{2(r-2M)} {L} \left( \frac{r^3}{r-2M} {L} \left( r^{-3} \upalpha ^{[-2]}\right) \right) \, . \end{aligned}$$

Here $L= \partial _t+\partial _{r*}$, ${\underline{L}}=\partial _t-\partial _{r*}$ are a null frame, where $r^*$ is the Regge–Wheeler coordinate. The quantities $\Psi ^{[+2]} = r^3 P^{[+2]}$ and $\Psi ^{[-2]}=r^3 P^{[-2]}$ can be shown to satisfy the Regge–Wheeler equation^3^
7where  denotes the spin-2-weighted Laplacian on the unit sphere8Remarkably, (7) is precisely the same equation which appeared as one of the equations governing the “metric perturbations” approach discussed at the beginning of Sect. 1.1!

Unlike (2) with $s=\pm \,2$, the above equation (7) can be estimated on Schwarzschild just as for the wave equation $s=0$, since (7) is indeed endowed with the usual structure of energy estimates. In particular, both boundedness and integrated local energy decay can be obtained. (For analysis of (7), see the previous [17, 63] as well as the self-contained treatment in [31].) Estimates for $\upalpha ^{[\pm 2]}$ could then be recovered directly by integrating (5) as transport equations from initial data. Such integration on its own would lead, however, to “loss of derivatives” in the resulting estimates for $\upalpha ^{[\pm 2]}$. The Teukolsky equation itself (2) can be viewed as a further elliptic relation which allows one to gain back these derivatives, leading finally to boundedness results without loss of derivative, as well as integrated local energy decay and pointwise decay.

We remark that, beyond (2), in the context of the full proof of linear stability in [31], further transport equations and elliptic equations could then be used to appropriately estimate the remaining gauge dependent quantities.

*Other spins.* We note that the scheme of [31] has recently been applied also to the $s=\pm \, 1$ case by Pasqualotto [94]. This gives an alternative proof of boundedness and polynomial decay for the Maxwell equations on Schwarzschild, proven originally by Blue [12]. See also [105]. Decay for Maxwell in the case $|a|\ll M$ was obtained in [4]. For a direct treatment of (2) for $s=\pm \, 1$ in the case $|a|\ll M$, generalising some of the results of [94], there is the recent [81]. (This has more recently been followed by [Ma18b]; see Sect. 1.3.9.) For the cases $s=\pm \,1/2$ and $s=\pm \,3/2$ see [106]. See [49] for the related massive Dirac equation not covered by (2). We note also the papers [52, 53].

### The Main Result and First Comments on the Proof

The aim of the present paper is to extend the analysis of (2) for $s=\pm \, 2$ from the Schwarzschild $a=0$ case considered in [31] to the very slowly rotating Kerr case with parameters $|a|\ll M$. A rough version of our main result is the following:

#### Theorem

(Rough version) Let $|a|\ll M$. Solutions $\upalpha ^{[\pm 2]}$ to the spin $s=\pm \,2$ Teukolsky equation (2) on Kerr exterior spacetimes $({\mathcal {M}}, g_{a,M})$ arising from regular localised initial data on a Cauchy hypersurface $\Sigma _0$ remain uniformly bounded and satisfy an $r^p$-weighted energy hierarchy and polynomial decay.

The precise statements embodying the above will be given as Theorem 4.1. See also immediately Corollary 4.1 and (38) for the pointwise decay statements obtained.

The proof of our Theorem combines the use of the quantities $P^{[\pm 2]}$ introduced in our previous [31] with a simplified version of the framework introduced in [40, 45] for frequency localised energy estimates, which as discussed in Sect. 1.1.2 are useful to capture the obstruction to decay associated with trapped null geodesics. (In the special case of axisymmetric solutions, this frequency localisation can be avoided and our proof can be expressed entirely in physical space. See already Sect. 1.2.5.)

The crucial observation which allows this technique to work is the following: In the scheme introduced in [31], it is not in fact absolutely necessary that the quantities $P^{[\pm 2]}$ each satisfy a completely decoupled equation (7). It would have been permissible if the equation (7) for $P^{[\pm 2]}$ was somehow still coupled to $\upalpha ^{[\pm 2]}$ on the right hand side, provided that this coupling was at a suitable “lower order”, in the sense that these lower order terms can indeed be recovered by the transport (and elliptic equations) which were used in [31] to estimate $\upalpha ^{[\pm 2]}$.

It turns out, remarkably, that when analogues of the quantities $P^{[\pm 2]}$ are defined for Kerr, even though the exact decoupling from $\upalpha ^{[\pm 2]}$, respectively, breaks down, the resulting equations indeed only couple to $\upalpha ^{[\pm 2]}$ in the “weak” sense described above.

We explain below this structure in more detail, and how it is implemented in our proof (where we will in fact be able to circumvent use of elliptic estimates).

#### The Generalisation of $P^{[\pm 2]}$ to Kerr

Our physical-space definition for $P^{[+2]}$, generalising (5), is given as

9 $$\begin{aligned} P^{[+2]} = -\frac{(r^2+a^2)^{1/2}}{2\Delta } {\underline{L}} \left( \frac{(r^2+a^2)^2}{\Delta } {\underline{L}}\left( \Delta ^2 \left( r^2+a^2\right) ^{-\frac{3}{2}} \upalpha ^{[+2]}\right) \right) \,. \end{aligned}$$

A similar formula holds for $P^{[-2]}$. See already Sect. 3.1. A computation reveals that the rescaled $\Psi ^{[+2]}=(r^2+a^2)^{\frac{3}{2}}P^{[+2]}$ satisfies an equation of the form

10 $$\begin{aligned} {\mathfrak {R}}^{[+2]} \Psi ^{[+2]} = c_1(r) \partial _\phi ({\underline{L}}\upalpha ^{[+2]}) +c_2(r) {\underline{L}} \upalpha ^{[+2]}+c_3(r)\partial _\phi \upalpha ^{[+2]} +c_4(r)\upalpha ^{[+2]},\nonumber \\ \end{aligned}$$

where ${\mathfrak {R}}^{[+2]}$ is a second order operator defined on Kerr generalising the Regge–Wheeler operator appearing on the left hand side of (7), which has good divergence properties and thus admits energy currents. Consistent with the total decoupling in the Schwarzschild case, the coefficient functions $c_i(r)$ above are all *O*(|*a*|). Provided that $\upalpha ^{[+2]}$ can indeed be viewed as being of two degrees lower in differentiability than $\Psi ^{[+2]}$, then the right hand side is “zero’th order” in $\Psi ^{[+2]}$. Let us note, however, that if we use only the transport relation (9), then the right hand side of (10) can only be viewed as “first order” in $\Psi ^{[+2]}$, as integration of (9) does not improve differentiability. Thus, to exploit fully this structure, one must also invoke in general elliptic relations connecting $\alpha ^{[+2]}$ and $\Psi ^{[+2]}$ that can be derived by revisiting equation (2) itself. As we shall see below, it turns out, however, that we shall be able to avoid invoking this by exploiting more carefully the special structure and the non-degeneration of the derivative $\partial _{r^*}\Psi ^{[\pm 2]}$. We describe in Sects. 1.2.2–1.2.3 how these terms can be controlled.

We emphasise already that the above structure of the terms appearing on the right hand side of (10) is surprising. Upon perturbing (7) one would expect higher order terms in $\Psi ^{[\pm 2]}$ to appear which cannot be incorporated in the definition of ${\mathfrak {R}}^{[+2]}$ so as to preserve its good divergence properties. We note already that in the axisymmetric case, the right hand side of (10) is of even lower order, as the $\partial _\phi$ derivatives vanish. The deeper reason why these terms cancel is not at all clear. See also the remarks in Sect. 1.2.6 below.

#### Estimates Away from Trapping

Away from trapping, it suffices to treat the right hand side of (10) as if it were at the level of a general “first order” perturbation in $\Psi ^{[+2]}$.

To see this, let us note first that suitably away from $r=3M$, the *f* and *y*-multiplier estimate of [31] leads in the Schwarzschild case to a coercive spacetime integral containing *all* first derivatives of $\Psi ^{[+2]}$ (with suitable weights towards the horizon and infinity). This coercivity property away from trapping is manifestly preserved to perturbations to Kerr for $|a|<a_0\ll M$ sufficiently small. We may add also a small multiple of the $r^\eta$-current for an $\eta >0$ to generate extra useful weights near infinity. Moreover, we may add a suitable multiple of the energy estimate associated to a vector field $\partial _t + \chi \upomega _+ \partial _\phi$ which connects the Hawking vector field on the horizon with the stationary vector field $\partial _t$. This ensures positive boundary terms on suitable spacelike and null boundaries, at the expense of generating an *O*(|*a*|) bulk term supported where $\chi '=0$, which is chosen to be away from trapping. Thus, this bulk term can again be absorbed by the coercive terms of the *f* and *y*-multipliers.

On the other hand, commutation of equation (9) by the Killing fields $\partial _t$ and $\partial _\phi$ allows one to estimate all terms involving $\upalpha$ and ${\underline{L}}\upalpha$ and their derivatives appearing on the right hand side of (10) from the spacetime estimate for $\Psi ^{[+2]}$ by appropriate transport estimates. (Here, we note that we must make use of the extra $r^\eta$ weight, just as in [31].) Thus, were it not for trapping, one could easily absorb the error terms on the right hand side of (10).

#### Frequency Localised Analysis of the Coupled System Near Trapping

In view of the above discussion, the terms on the right hand side of (10) are most dangerous near trapping. Let us take a more careful look at the structure of (9)–(10) using our frequency analysis.

At the level of the formally separated solutions (3), the operator ${\underline{L}}$ takes the form

11 $$\begin{aligned} -{\underline{L}}=\frac{d}{dr^*} +i\omega -\frac{iam}{r^2+a^2}, \end{aligned}$$

where $r^*$ is a Regge–Wheeler type coordinate, the relation (9) reads

12 $$\begin{aligned} \Psi ^{[+2]} = -\frac{1}{2} w^{-1}{\underline{L}}\left( w^{-1}{\underline{L}}\left( w\cdot u^{[+2]}\right) \right) \end{aligned}$$

where $$u^{[+2]} = \Delta \sqrt{r^2+a^2} \upalpha ^{[+2]}$$,

13 $$\begin{aligned} w:= \frac{\Delta }{(r^2+a^2)^2} \end{aligned}$$

and the “Regge–Wheeler” type equation (10) takes the form

14 $$\begin{aligned} \frac{d^2}{(dr^*)^2}{\Psi ^{[+2]}} +(\omega ^2 -{\mathcal {V}} )\Psi ^{[+2]}= & {} a\left( {\mathfrak {c}}_1(r) i m +{\mathfrak {c}}_2(r) \frac{a}{r} \right) {\underline{L}} (u^{[+2]} w)\nonumber \\&+\, a^2 w\left( {\mathfrak {c}}_3(r) \frac{1}{r} a i m + {\mathfrak {c}}_4(r) \right) (u^{[+2]} w). \qquad \end{aligned}$$

Here ${\mathcal {V}}$ is a real potential depending smoothly on *a* which reduces to the separated version of the Regge–Wheeler potential for $a=0$ and the ${\mathfrak {c}}_i$ are bounded functions. Cf. (10) and see Appendix A.3.

At the separated level, using a frequency localised version of the current *f* of [31], chosen to vanish at the (frequency-dependent) maximum of the potential ${\mathcal {V}}$ as in of [40], together with a frequency localised *y*-current and the frequency-localised energy estimate (multiplication by $\omega \Psi$) one can prove the ODE analogue of a degenerating integrated local energy decay for $\Psi ^{[\pm 2]}$, with a right hand side involving the right hand side of (14). Considerations are different in the “trapped frequency range”

15 $$\begin{aligned} 1\ll \omega ^2\sim \lambda _{m \ell }^{[s]} + s , \end{aligned}$$

and the non-trapped frequencies. (Here $\lambda _{m \ell }$ are the eigenvalues of the spin-weighted Laplacian (8) reducing to $\ell (\ell +1)-s^2 \ge 2$ in the case $a=0$.)

In the trapped frequency range (15), the above multiplier gives an estimate which can schematically be written as:

16 $$\begin{aligned}&\int _{r \sim 3M} |\Psi ^{[+2]}|^2 + |\partial _{r^*}\Psi ^{[+2]}|^2 dr^* \nonumber \\&\quad \lesssim \text {terms controllable by physical space estimates (cf.~Sect.~1.2.2)} \nonumber \\&\qquad + |a|\int _{r \sim 3M}( \omega \Psi ^{[+2]} + \partial _{r^*}\Psi ^{[+2]})\nonumber \\&\qquad \quad \Big \{ (a i m +1) {\underline{L}} (u^{[+2]} w) + a^2 \left( a i m + 1 \right) (u^{[+2]} w)\Big \}dr^* \, . \end{aligned}$$

This should be thought of as a degenerate integrated local energy decay bound for $\Psi ^{[+2]}$. Considering the right hand side of (16), we note that naive integration of (12) as a transport equation is not sufficient to control the integral on the right hand side by the left hand side. This is not surprising: In constrast to the considerations away from trapping of Sect. 1.2.2, in general now only terms which can be truly thought of as “zero’th order” in $\Psi ^{[+2]}$ can manifestly be absorbed by the left hand side of (16), in view of the absence of an $\omega ^2|\Psi ^{[+2]}|^2$ and $\Lambda |\Psi ^{[+2]}|^2$ coercive term.

One way to try to realise the right hand side of (16) as “zero’th order” in $\Psi ^{[+2]}$ would be to invoke, in addition to the transport (12), also the elliptic estimates of [31]. It turns out, however, that exploiting the presence of the good first order term $|\partial _{r^\star }\Psi ^{[+2]}|^2$ on the left hand side of (16), one can argue in a more elementary manner: Indeed, by commuting (12) with $\partial _{r^*}$ and exploiting the relation (11), one can indeed rewrite the right hand side so as to absorb it into the left hand side.

Let us note finally that for “non-trapped” frequencies (i.e. outside the frequency range (15)), one can arrange the frequency localised multiplier so that terms $m^2|\Psi ^{[+2]}|^2$ and $\omega ^2|\Psi ^{[+2]}|^2$ appear on the left hand side of (16) without degeneration. One can then easily absorb the right hand side just as in Sect. 1.2.2 treating it essentially as one would a general “first order” term.

#### Technical Comments

Let us discuss briefly the technical implementation of the above argument.

As in [40], by using the smallness of the Kerr parameter *a*, the fixed frequency analysis of Sect. 1.2.3, restricted entirely to real frequencies $\omega \in {\mathbb {R}}$, can indeed be implemented to general solutions $\upalpha ^{[\pm 2]}$ of the Cauchy problem for (2), despite the fact that we do not know a priori that solutions are square integrable in time. This requires, however, applying cutoffs to $\upalpha$ in order to justify the Fourier transform, and thus one must estimate inhomogeneous versions of (2) and thus also inhomogeneous versions of the resulting ODE (14). These inhomogeneous terms must themselves be bound by the final estimates.

As opposed to the cutoffs of [40, 45], we here will only cut off the solution in a region $r^*\in [2A_1^*,2A_2^*]$ near trapping. Thus, the resulting inhomogeneous terms will be supported in a fixed region of finite $r^*$. Moreover, the fixed frequency ODE estimates of Sect. 1.2.3 will only be applied in the region $r^*\in [A_1^*,A_2^*]$. They will be combined with physical space estimates of Sect. 1.2.2. These estimates are now coupled however via boundary terms on $r=A_1$ and $r=A_2$. The fixed frequency multipliers applied to $\Psi ^{[+2]}$ are chosen so as to be frequency independent near $A_1$ and $A_2$ and coincide precisely with those used in the physical space estimates in the *away* region. As a result, after summation over frequencies, the boundary terms in the mutliplier currents exactly cancel. This is similar to a scheme used previously in [7]. There are also boundary terms associated with the transport equations, but these can be absorbed using the smallness of *a*.

The above argument leads to a degenerate energy boundedness and integrated local energy decay for both $\Psi ^{[\pm 2]}$ and $\upalpha ^{[\pm 2]}$. This preliminary decay bound will be stated as Theorem 9.1. From Theorem 9.1, we can easily improve our estimates at the event horizon, using the red-shift technique of [39], and then we can easily infer polynomial decay using the weighted $r^p$ method of [38]—all directly in physical space.

#### The Axisymmetric Case

We have already remarked that in the axisymmetric case $\partial _\phi \upalpha ^{[\pm 2]}=0$, the right hand side of (10) is of lower order. An even more important simplification is that trapped null geodesics all asymptote to a single value of $r=r_{\mathrm{trap}}$ which is near 3*M*, independent of frequency. As a result, there is no need for frequency-localised analysis and the whole argument can be expressed entirely in physical space. This is convenient for non-linear applications. We shall explain how this simplified argument can be explicitly read off from our paper in Sect. 9.6.

#### Final Remarks

Given the analogue of [104] for $s=\pm \, 2$, the argument can in principle be applied for the whole subextremal range $|a|<M$ following the continuity argument of [45], but in the present paper we shall only consider the case $|a|\ll M$, where the lower order terms also have a useful smallness factor bounded by *a*, and the relevant multiplier currents can thus be constructed as perturbations of Schwarzschild. The general case will be considered in part II of this series, following the more general constructions of [45].

There are other generalisations of $P^{[\pm 2]}$ to Kerr which have been considered previously in the literature, see [21, 102] and the recent review [57]. In contrast to our situation, the quantities of [21, 102] do indeed satisfy decoupled equations, though the transformations must now be defined in phase space, and the transformed potentials are somewhat non-standard in their frequency dependence. It would be interesting to find an alternative argument using these transformations. We hope to emphasise with our method, however, that exact decoupling is not absolutely necessary for quantities to be useful.

### Other Related Results

We collect other related recent results concerning the stability of black holes. The literature has already become vast so the list below is in no way exhaustive. See also the surveys [42, 43].

#### Metric Perturbations

An alternative approach to linear stability in the Schwarzschild case would go through the theory of so-called metric perturbations. See for instance [60, 71] for estimates on the additional Zerilli equation which must be understood in that approach. We note the paper [35].

#### Canonical Energy

As discussed above, one of the difficulties in understanding linearised gravity is the lack of an obvious coercive energy quantity for the full system, even in the $a=0$ case. The Lagrangian structure of the Einstein equations (1) does give rise however to a notion of canonical energy, albeit somewhat non-standard in view of diffeomorphism invariance, and this can indeed be used to infer certain weak stability statements. For some recent results which have been obtained using this approach, see [69, 97] and the related [64].

#### Precise Power-Law Asymptotics

Though one expects that the decay bounds obtained here are in principle sufficient for non-linear applications, it is of considerable interest for a wide range of problems to obtain sharp asymptotics of solutions, of the type first suggested by [96]. For upper bounds on decay compatible with some of the asymptotics of [96], see [47, 90, 91]. Lower bounds were first obtained in [76]. The most satisfying results are the sharp asymptotics recently obtained by [1, 2] for the $s=0$, $a=0$ case. Such results in particular have applications to the interior structure of black holes (see [76]).

#### Extremality and the Aretakis Instability

Whereas some stability results for $s=0$ carry over to the extremal case $|a|=M$, it turns out that, already in axisymmetry [7], the *transversal* derivatives along the horizon grow polynomially [7, 8]. This phenomenon is now known as the *Aretakis instability*. The Aretakis instability has been shown to hold also in the case $s=\pm \,2$ by [77]. Understanding the non-axisymmetric case is completely open; see [5] for some of the additional new phenomena that arise.

#### Nonlinear Model Problems and Stability Under Symmetry

Though nonlinear stability of both Schwarzschild and Kerr is still open, various model problems have been considered which address some of the specific technical difficulties expected to occur.

Issues connected to the handling of decay for quadratic nonlinearities in derivatives are addressed in the models considered in [79, 80]. The Maxwell–Born–Infeld equations on Schwarzschild were recently considered in [95]. This latter system, of independent interest in the context of high energy physics, can be thought to capture at the same time aspects of both the quasilinear difficulties as well as the tensorial difficulties (at the level of $s=\pm \,1$) inherent in (1).

Turning to stability under symmetry, the literature is vast. For the Einstein–scalar field system under spherical symmetry, see [23, 36]. For the vacuum equations (1), [62] provides the first result on the non-linear stability of the Schwarzschild solution in symmetry, considering biaxial symmetry in $4+1$-dimensions. This again reduces to a $1+1$ problem. Beyond $1+1$, some aspects of the vacuum stability problem in axisymmetry are captured in a wave-map model problem whose study was initiated by [70]. Very recently, Klainerman–Szeftel [74] have announced a proof of the non-linear stability of Schwarzschild in the polarised, axisymmetric case.

#### Analogues with $\Lambda \ne 0$

There are analogues of the questions addressed here when the Schwarzschild and Kerr solutions are replaced with the Schwarzschild–(anti) de Sitter metrics and Kerr–(anti) de Sitter metrics, which are solutions of (1) when a cosmological term $\Lambda g_{\mu \nu }$ is added to the right hand side. These solutions are discussed in [20].

In the de Sitter case $\Lambda >0$, the analogous problem is to understand the stability of the spatially compact region bounded by the event and so-called cosmological horizons. Following various linear results [13, 37, 48, 59, 109] the full non-linear stability of this region has been obtained in remarkable work of Hintz–Vasy [67]. This de Sitter case is characterized by exponential decay, so many of the usual difficulties of the asymptotically flat case are not present. The stability of the “cosmological region” beyond the event horizon has been considered in [101].

The case of $\Lambda <0$ has been of considerable interest in the context of high energy physics. Already, pure AdS spacetime fails to be globally hyperbolic. In general, asymptotically AdS spacetimes have a timelike boundary at infinity where boundary conditions must be prescribed to obtained well-posed problems.

For reflective boundary conditions, the analogue of equation (2) on pure AdS space admits infinitely many periodic solutions. In view of this lack of decay in the reflective case, it is natural to conjecture instability at the non-linear level [29], once backreaction is taken into account.^4^ This nonlinear instability has indeed been seen in the seminal numerical study [15], which moreover sheds light on the relevance of resonant frequencies for calculating a time-scale for growth. Very recently, the full nonlinear instability of pure AdS space has been proven in the simplest model for which the problem can be studied [88], exploiting an alternative physical-space mechanism.

In the case of Kerr–AdS, one has logarithmic decay [65]—but in general no faster [66]—for the analogue of (2) with $s=0$, on account of the fact that trapped null geodesics, in contrast with the situation described in Sect. 1.1.2, are now stable. Again, these results may suggest instability at the non-linear level, as this slow rate of decay is in itself insufficient to control backreaction.

#### Scattering Theory

A related problem to that of proving boundedness and decay is developing a scattering theory for (2). Fixed frequency scattering theory for (2) is discussed in [22]. It was in fact the equality of the reflexion and transmission coefficients between the Teukolsky, Regge–Wheeler and Zerilli equations that first suggested the existence of Chandrasekhar’s transformations [22]. A definitive physical space scattering theory was developed in the Schwarzschild case in [32, 33] for $s=0$, see also [92], and was recently extended to the Kerr case in [44] for the full sub-extremal range of parameters $|a|<M$.

Turning to the fully non-linear theory of (1), a scattering construction of dynamic vacuum spacetimes settling down to Kerr was given in [30]. The free scattering data allowed in the latter were very restricted, however, as the radiation tail was required to decay exponentially in retarded time, and thus the spacetimes produced are measure zero in the set of small perturbations of Kerr relevant for the stability problem.

For scattering for the Maxwell equations, see [9]. For results in the $\Lambda >0$ case, see [56, 85] and references therein.

#### Stability and Instability of the Kerr Black Hole Interior

The conjectured non-linear stability of the Kerr family refers only to the *exterior* of the black hole region. Considerations in the black hole interior are of a completely different nature. The Schwarzschild case $a=0$ terminates at a spacelike singularity, whereas for the rotating Kerr case $0<|a|<M$, the Cauchy development of two-ended data can be smoothly extended beyond a Cauchy horizon. The $s=0$ case of (2) in the Kerr interior (as well as the simpler Reissner–Nordström case) has been studied in [46, 50, 51, 58, 76, 78, 83, 84], and both $C^0$-stability but also $H^1$-instability have been obtained. See [54, 55] for the extremal case. In the full nonlinear theory, it has been proven that if the Kerr exterior stability conjecture is true, then the bifurcate Cauchy horizon is globally $C^0$-stable [34]. This implies in particular that the $C^0$ inextendibility formulation of “strong cosmic censorship” is false. See [24].

#### Note Added

Very recently, [82] gave a related approach to obtaining integrated local energy decay estimates for the Teukolsky equation in the $|a|\ll M$ case, following the frequency localisation framework of [40] and again based on proving estimates for $\Psi$ defined by generalisations of the transformations used in [31].

### Outline of the Paper

We end this introduction with an outline of the paper.

We begin in Sect. 2 by recalling the notation from [45] regarding the Kerr metric and presenting the Teukolsky equation in physical space for spin $s=\pm \,2$.

We then define in Sect. 3 our generalisations to Kerr of the quantities $P^{[\pm 2]}$, the rescaled quantities $\Psi ^{[\pm 2]}$ and the intermediate quantities $\uppsi ^{[\pm 2]}$, as used in [31], and derive our generalisation of the Regge–Wheeler equation for $\Psi ^{[\pm 2]}$, now coupled to $\uppsi ^{[\pm 2]}$ and $\upalpha ^{[\pm 2]}$.

In Sect. 4 we shall define various energy quantities which will allow us in particular to formulate our definitive (non-degenerate) boundedness and decay results, stated as Theorem 4.1.

The first step in the proof of Theorem 4.1 is to obtain integrated local energy decay. In Sect. 5, we shall prove a conditional such estimate, using entirely physical space methods, for the coupled system satisfied by $\Psi ^{[\pm 2]}$, $\uppsi ^{[\pm 2}$, and $\upalpha ^{[\pm 2]}$. In view of the way this will be used, we must allow also inhomogeneous terms on the right hand side of the Teukolsky equation. We apply the physical space multiplier estimates and transport estimates and transport estimates directly from [31], except that these estimates must now be coupled. The resulting estimates (see the propositions of Sects. 5.1 and 5.2) contain on their right hand side an additional timelike boundary term on $r=A_1$ and $r=A_2$ for $A_1<3M<A_2$. To control these terms, we will have to frequency localise the estimates in the region $r\in [A_1,A_2]$. We also give certain auxiliary physical space estimates for the homogeneous Teukolsky equation and its derived quantities (Sect. 5.3).

The next three sections will thus concern frequency localisation. Sect. 6 will interpret Teukolsky’s separation of (2) for spin $s=\pm \,2$ in a framework generalising that introduced in [45] for the $s=0$ case. In Sect. 7, we define the frequency localised versions of $P^{[\pm 2]}$ and derive the coupled system of ordinary differential equations satisfied by the $P^{[\pm 2]}$ and $\upalpha ^{[\pm 2]}$. In Sect. 8 we then obtain estimates for this coupled system of ODE’s in the region $r\in [A_1,A_2]$. The main statement is summarised as Theorem 8.1 and can be thought of as a fixed frequency version of the propositions of Sects. 5.1–5.2, now valid in $r\in [A_1,A_2]$. The estimate is again conditional on controlling boundary terms, but the energy currents will have been chosen so that the most difficult of these, when formally summed, exactly cancel those appearing in the proposition of Sect. 5.1.

In Sect. 9, we shall turn in ernest to the study of the Cauchy problem for (2) to obtain a preliminary degenerate energy boundedness and integrated local energy decay estimate in physical space. This is stated as Theorem 9.1. To obtain this, we cut off our solution of (2) in the future so as to allow for frequency localisation in $r\in [A_1,A_2]$. This allows us to apply Theorem 8.1 and sum over frequencies. We apply also the propositions of Sects. 5.1–5.2 to the cutoff-solution and sum the estimates. The cutoff generates an inhomogeneous term which is however only supported in a compact spacetime region. By revisiting suitable estimates, the cutoff term can then be estimated exploiting the smallness of *a*, following [40]. (We note that the fact that these cutoffs are here supported in a fixed, finite region of *r* leads to various simplifications.) We distill a simpler purely physical-space proof for the axisymmetric case in Sect. 9.6.

The final sections will complete the proof of Theorem 4.1 from Theorem 9.1, by first applying red-shift estimates of [39] to obtain non-degenerate control at the horizon (Sect. 10) and then the $r^p$-weighted energy hierarchy of [38] (Sect. 11). This part follows closely the analogous estimates in the Schwarzschild case [31].

Some auxilliary computations are relegated to Appendix A and B.

## The Teukolsky Equation on Kerr Exterior Spacetimes

We recall in this section the Teukolsky equation on Kerr spacetimes.

We begin in Sect. 2.1 with a review of the Kerr metric. We then present the Teukolsky equation on Kerr in Sect. 2.2, focussing on the case $s=\pm \,2$. This will allows us to state a general well-posedness statement in Sect. 2.3. Finally, in Sect. 2.4 we shall recall the relation of the $s=\pm \,2$ Teukolsky equation with the system of gravitational perturbations around Kerr.

### The Kerr Metric

We review here the Kerr metric and associated structures, following the notation of [45].

#### Coordinates and Vector Fields

For each $|a|<M$, recall that the Kerr metric in Boyer–Lindquist coordinates $(r,t,\theta ,\phi )$ takes the form

17 $$\begin{aligned} g_{a,M}= & {} -\frac{\Delta }{\rho ^2} (dt-a\sin ^2\theta d\phi )^2+\frac{\rho ^2}{\Delta }dr^2 +\rho ^2d\theta ^2\nonumber \\&+\frac{\sin ^2\theta }{\rho ^2}(a dt-(r^2+a^2)d\phi )^2, \end{aligned}$$

where

18 $$\begin{aligned} r_\pm =M\pm \sqrt{M^2-a^2},\qquad \Delta = (r-r_+)(r-r_-) ,\qquad \rho ^2 =r^2+a^2\cos ^2\theta .\qquad \end{aligned}$$

We recall from [45] the fixed ambient manifold-with-boundary ${\mathcal {R}}$, diffeomorphic to ${\mathbb {R}}^+\times {\mathbb {R}}\times {\mathbb {S}}^2$ and the coordinates $( r, t^*, \theta ^*, \phi ^*)$ on ${\mathcal {R}}$ known as Kerr star coordinates.

We recall the relations

$$\begin{aligned} t(t^*, r) = t^*- {\bar{t}}(r) \ \ \ , \quad phi (\phi ^*, r) = \phi ^* - \bar{\phi }(r) \mod 2\pi \ \ \ , \ \ \ \theta = \theta ^* \end{aligned}$$

relating Boyer–Lindquist and Kerr star coordinates. We do not need here the explicit form of ${\bar{t}}(r)$ and $\bar{\phi }(r)$; see [45], Section 2.1.3 but remark that they both vanish for $r\ge 9/4M$. When expressed in Kerr star coordinates, the metric (17) (defined a priori only in the interior of ${\mathcal {R}}$) extends to a smooth metric on ${\mathcal {R}}$, i.e. it extends smoothly to the event horizon ${\mathcal {H}}^+$ defined as the boundary $\partial {{\mathcal {R}}}=\{r=r_+\}$.

It is easy to see that the coordinate vector fields $T=\partial _{t^*}$ and $\Phi =\partial _{\phi ^*}$ of the fixed coordinate system coincide for all *a*, *M* with the coordinate vector fields $\partial _t$ and $\partial _\phi$ of Boyer–Lindquist coordinates, which are Killing for the metric (17). We recall that *T* is spacelike in the so-called *ergoregion* ${\mathcal {S}}=\{\Delta -a^2\sin ^2\theta <0\}$. Setting

$$\begin{aligned} \upomega _+\doteq \frac{a}{2Mr_+}, \end{aligned}$$

we recall that the Killing field

$$\begin{aligned} K=T+\upomega _+ \Phi \end{aligned}$$

is null on the horizon ${\mathcal {H}}^+$ and is timelike in $\{r_+<r<r_++R_K\}$ for some $R_K=R_K(a_0,M)$ where $R_K\rightarrow \infty$ as $a_0\rightarrow 0$.

An additional important coordinate will be $r^*$ defined to be a function $r^*(r)$ such that

19 $$\begin{aligned} \frac{dr^*}{dr} = \frac{r^2+a^2}{\Delta } \end{aligned}$$

and centred as in [45] so that $r^*(3M)=0$. Note that $r^*\rightarrow -\infty$ as $r\rightarrow r_+$, while $r^*\rightarrow \infty$ as $r\rightarrow \infty$. Given a parameter *R* thought of as an *r*-value, we will often denote $r^*(R)$ by $R^*$.

The vector fields

20 $$\begin{aligned} L = \partial _{r^*}+T+ \frac{a}{r^2+a^2}\Phi , \qquad {\underline{L}}= -\partial _{r^*} +T +\frac{a}{r^2+a^2}\Phi , \end{aligned}$$

where $\partial _{r^*}$ is defined with respect to $(r^*,t,\theta ,\phi )$ coordinates, define principal null directions. We have the normalisation

$$\begin{aligned} g(L, {\underline{L}})=-2\frac{\Delta \rho ^2 }{(r^2+a^2)^2} \, . \end{aligned}$$

The vector field *L* extends smoothly to ${\mathcal {H}}^+$ to be parallel to the null generator, while ${\underline{L}}$ extends smoothly to ${\mathcal {H}}^+$ so as to vanish identically. The quantity $\Delta ^{-1}{\underline{L}}$ has a smooth nontrivial limit on ${\mathcal {H}}^+$. The vector fields *L* and ${\underline{L}}$ are again *T*-(and $\Phi$-)invariant.

#### Foliations and the Volume Form

For all values $\tau \in {\mathbb {R}}$, we recall that the hypersurfaces $\Sigma _\tau =\{t^*=\tau \}$ are spacelike (see [45], Section 2.2.5). We will denote the unit future normal of $\Sigma _\tau$ by $n_{\Sigma _\tau }$. We recall the notation

$$\begin{aligned} {\mathcal {R}}_0= & {} \{t^*\ge 0\}, \qquad {\mathcal {R}}_{(0,\tau )}=\{0\le t^*\le \tau \}, \qquad {\mathcal {H}}^+_0={\mathcal {R}}_0\cap {\mathcal {H}}^+, \\&{\mathcal {H}}^+_{(0,\tau )}={\mathcal {R}}_{(0,\tau )}\cap {\mathcal {H}}^+. \end{aligned}$$

For polynomial decay following the method of [38, 87], we will also require hypersurfaces $\widetilde{\Sigma }_{\tau }$ which connect the event horizon and null infinity. For this we fix some $0<\eta <1$ and define the coordinate

21 $$\begin{aligned} {\tilde{t}}^* = t^* - \xi \left( r^*\right) \left( r^* + 2M \left( \frac{2M}{r}\right) ^\eta - R_\eta ^* - 2M \left( \frac{2M}{R_\eta }\right) ^\eta - M\right) \end{aligned}$$

where $\xi$ is a smooth cut-off function equal to zero for $r \le R_\eta$ and equal to 1 for $r\ge R_\eta +M$. It is straightforward if tedious to show that for $R_\eta$ sufficiently large (and a suitably chosen function $\xi$) the hypersurfaces $\widetilde{\Sigma }_{\tau }$ defined by

22 $$\begin{aligned} \widetilde{\Sigma }_{\tau } := \{ {\tilde{t}}^* = \tau \} \, \end{aligned}$$

are smooth and spacelike everywhere, in fact $c_\eta r^{-\eta -1} \le -g\left( \nabla {\tilde{t}}^*, \nabla {\tilde{t}}^*\right) \le C_\eta r^{-\eta -1}$ indicating that the hypersurfaces become asymptotically null near infinity. We take this $R_\eta$ as fixed from now on.

We will in fact use coordinates $\left( {\tilde{t}}^*, r, \theta ,\phi ^*\right)$ and perform estimates in the spacetime regions

$$\begin{aligned} \widetilde{{\mathcal {R}}}(\tau _1,\tau _2)=\{\tau _1\le {\tilde{t}}^* \le \tau _2\}, \qquad \widetilde{{\mathcal {R}}}_0=\{{\tilde{t}}^* \ge 0\}. \end{aligned}$$

See Fig. 1.

**Fig. 1:**
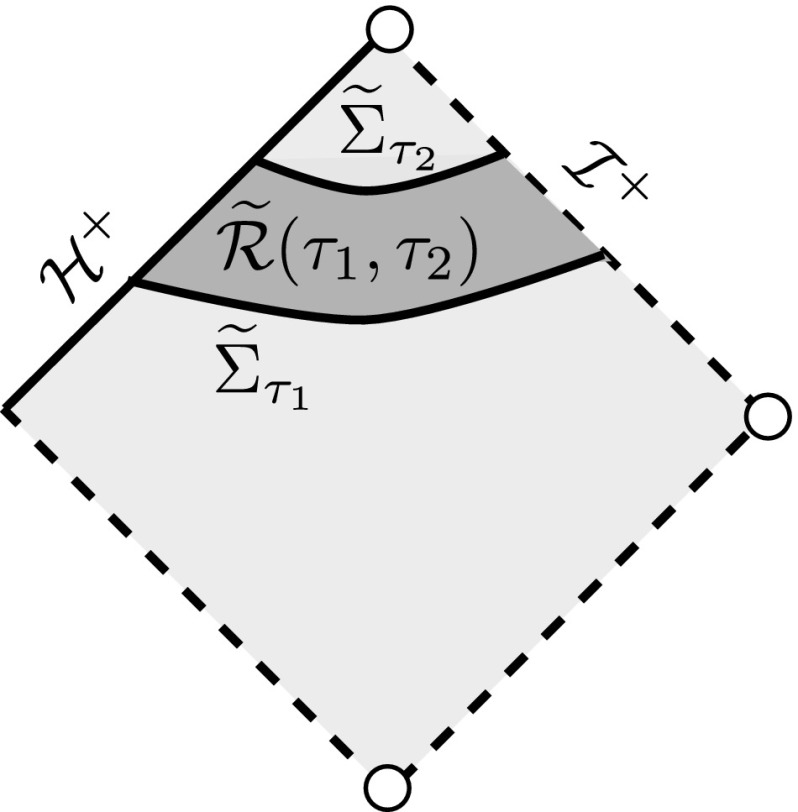
The region $$\widetilde{{\mathcal {R}}}(\tau _1,\tau _2)$$

We compute the volume form in the different coordinate systems (recalling that the *r* and $\theta$ coordinates are common to *all* coordinate systems, so $\rho ^2=r^2+a^2\cos ^2 \theta$ is unambiguously defined)

23 $$\begin{aligned} dV&=\rho ^2 dt \, dr\, \sin \theta d\theta d\phi = \frac{\rho ^2 \Delta }{\left( r^2+a^2\right) } dt \, dr^* \, \sin \theta d\theta d\phi ^* \nonumber \\&= \rho ^2 dt^* \, dr \, \sin \theta d\theta d\phi = \rho ^2 d{\tilde{t}}^* \, dr \, \sin \theta d\theta d\phi ^* \, . \end{aligned}$$

We will often use the notation

$$\begin{aligned} d\sigma =\sin \theta d\theta d\phi . \end{aligned}$$

Denoting the (timelike) unit normal to the hypersurfaces (22) by $n_{{\widetilde{\Sigma }}_{\tau }}$ we compute in coordinates $\left( r,{\tilde{t}}^*, \theta ^*,\phi ^*\right)$

24 $$\begin{aligned} \sqrt{g_{{\widetilde{\Sigma }}_{\tau }}} g\left( \frac{r^2+a^2}{\Delta } {\underline{L}}, n_{{\widetilde{\Sigma }}_{\tau }}\right)&=v\left( r,\theta \right) \rho ^2 \sin \theta \text { and } \sqrt{g_{{\widetilde{\Sigma }}_{\tau }}} g\left( L, n_{{\widetilde{\Sigma }}_{\tau }}\right) \nonumber \\&=v\left( r,\theta \right) \frac{1}{r^{1+\eta }} \rho ^2 \sin \theta \end{aligned}$$

for a function *v* with $C^{-1} \le v \le C$. In particular, the volume element on slices of constant ${\tilde{t}}^*=\tau$ satisfies

$$\begin{aligned} dV_{\widetilde{\Sigma }_{\tau }} =\sqrt{g_{\widetilde{\Sigma }_\tau }} dr d\theta d\phi = v\left( r, \theta \right) r^2 r^{-\frac{1+\eta }{2}} dr d\sigma \end{aligned}$$

for a (potentially different) function *v* with $C^{-1} \le v \le C$.

For future reference we note that, again in coordinates $\left( r,{\tilde{t}}^*,\theta ^*,\phi ^*\right)$, we have on the null hypersurfaces corresponding to the horizon and null infinity respectively the relations

25 $$\begin{aligned} \sqrt{g_{{\mathcal {H}}^+}} \ g\left( \frac{r^2+a^2}{\Delta } {\underline{L}}, L\right) = v\left( r,\theta \right) \sin \theta \ \ \ \text {and} \ \ \ \sqrt{g_{{\mathcal {I}}^+}} \ g\left( L,{\underline{L}}\right) = v\left( r,\theta \right) \rho ^2 \sin \theta \, , \end{aligned}$$

where the volume forms are understood to be themselves normalised by *L* and ${\underline{L}}$, respectively. The above will be the expressions that arise in the context of the divergence theorem.

Finally, we note the covariant identities

26 $$\begin{aligned} \nabla _a \left( \frac{1}{\rho ^2} \frac{r^2+a^2}{\Delta } {\underline{L}}^a \right) = 0 \ \ \ \text {and} \ \ \ \nabla _a \left( \frac{1}{\rho ^2} \frac{r^2+a^2}{\Delta } L^a \right) = 0 \, , \end{aligned}$$

which are most easily checked in Boyer–Lindquist coordinates.

#### The Very Slowly Rotating Case $|a|<a_0 \ll M$

In the present paper, we will restrict to the very slowly rotating case. This will allow us to exploit certain simplifications which arise from closeness to Schwarzschild.

Recall that the hypersurface $r=3M$ in Schwarzschild is known as the *photon sphere* and corresponds to the set where integrated local energy decay estimates necessarily degenerate. In the case $|a|<a_0\ll M$ the trapping is localised near $r=3M$ while the ergoregion ${\mathcal {S}}$ is localised near $r=2M$. See the general discussion in [43]. Let us quantify this below by fixing certain parameters.

We will fix parameters $A_1<3M<A_2$ sufficiently close to 3*M*. We note already that for sufficiently small $|a|<a_0\ll M$, then all future trapped null geodesics will asymptote to an *r* value contained in $r\in [A_1,A_2]$. (We shall not use this fact directly, but rather, a related property concerning the maximum of a potential function associated to the separated wave equation. See already Lemma 8.2.1.)

We moreover can choose $a_0$ small enough so that in addition, $R_K>A_1$ and so that the ergoregion satisfies ${\mathcal {S}}\subset \{r^*<4A_1^*\}$.

Fixing a cutoff function $\chi (r^*)$ which is equal to 1 for $r^*\le 4A_1^*$ and 0 for $r^*\ge 2A_1^*$ we define the vector field $T+\upomega _+\chi \Phi$. We note that by our arrangement, this vector field is now timelike for all $r>r_+$, Killing outside $\{4A_1^*<r^*<2A_1^*\}$, null on ${\mathcal {H}}^+$, and equal to *T* on $\{r^*\ge A_1^*\}$.

Finally, let us note that, if $A_1^*$ is sufficiently small, then restricting to small $a_0$, we have that $t=t^*$ for $r^*\ge 2A_1^*$ for all $|a|<a_0$.

We note in particular

$$\begin{aligned} t=t^*={\tilde{t}}^* \mathrm{\ in\ the\ region\ }2A_1^*\le r^*\le 2A^*_2. \end{aligned}$$

#### Parameters and Conventions

This paper will rely on fixing a number of parameters which will appear in the proof. We have just discussed the parameters $\eta$ and

$$\begin{aligned} A_1<3M< A_2 \end{aligned}$$

which have already been fixed.

We will also introduce fixed parameters

$$\begin{aligned} \delta _1, \delta _2,\delta _3, E \end{aligned}$$

which will be connected to adding multiplier constructions on Schwarzschild, as well as parameters $C_\sharp$, $c_\flat$, $C_\flat$ delimiting frequency ranges. In particular, eventually, these can be all thought of as fixed in terms of *M* alone.

We will introduce an additional smallness parameter $\varepsilon$ associated to the cutoffs in time. (This notation is retained from our [40].) Again, eventually, this will be fixed depending only on *M*.

Finally, we will exploit the slowly rotating assumption by employing $a_0$ as a smallness parameter, which will only be fixed at the end of the proof.

We introduce the following conventions regarding inequalities. For non-negative quantities ${\mathcal {E}}_1$ and ${\mathcal {E}}_2$, by

$$\begin{aligned} {\mathcal {E}}_1\lesssim {\mathcal {E}}_2 \end{aligned}$$

we mean that there exists a constant $C=C(M)>0$, depending only on *M*, such that

$$\begin{aligned} {\mathcal {E}}_1\le C(M) {\mathcal {E}}_2. \end{aligned}$$

We will sometimes use the notation

$$\begin{aligned} {\mathcal {E}}_1\lesssim {\mathcal {Q}}+{\mathcal {E}}_2 \end{aligned}$$

where ${\mathcal {Q}}$ is not necessarily a non-negative quantity. In this context, this will mean that there exist constants *c*(*M*), *C*(*M*) such that

$$\begin{aligned} c{\mathcal {E}}_1\le {\mathcal {Q}} +C{\mathcal {E}}_2. \end{aligned}$$

Note that two inequalities of the above form can be added when the terms ${\mathcal {Q}}$ are identical.

Before certain parameters are fixed, say $\delta _1$, we will use the notation $\lesssim _{\delta _1}$ to denote the additional dependence on $\delta _1$ of the constant $C(M,\delta _1)$ appearing in various inequalities. Only when the parameter is definitively fixed in terms of *M*, can $\lesssim _{\delta _1}$ be replaced by $\lesssim$.

On the other hand, in the context of the restriction to $a_0\ll M$, which will appear ubiquitously, the constant implicit in $\ll$ may depend on all parameters yet to be fixed. This will not cause confusion because restriction to smaller *a* will always be favourable in every estimate.

### The Teukolsky Equation for Spin Weighted Complex Functions

In this section we present the Teukolsky equation on Kerr.

We first review in Sect. 2.2.1 the notion of spin *s*-weighted complex functions and discuss some elementary properties of the spin *s*-weighted Laplacian in Sect. 2.2.2. We then recall in Sect. 2.2.3 the classical form of the Teukolsky operator for general spin. Finally, specialising to $s=\pm \, 2$ we derive in Sect. 2.2.4 rescaled quantities which satisfy an equation regular also on the horizon. It is in this form that we will be able to state well-posedness in the section that follows.

#### Spin *s*-Weighted Complex Functions on $S^2$ and ${\mathcal {R}}$

The Teukolsky equation will concern functions whose $(\theta ,\phi )$ (or equivalently $(\theta ,\phi ^*)$) dependence is that of a spin *s*-weighted function, for $s\in \frac{1}{2} {\mathbb {Z}}$. We will always represent such functions as usual functions $\upalpha (r,t,\theta ,\phi )$.

Smooth spin *s*-weighted functions on $S^2$ naturally arise, in a one-to-one fashion, from complex-valued functions on $S^3$ (viewed as the Hopf bundle) which transform in a particular way under the group action on the $S^1$ fibres of $S^3$, as will be described now. (Note that this is indeed natural as $S^3$ can be identified with the bundle of orthonormal frames on $S^2$, and the definition of the Teukolsky null curvature components indeed depends on a choice of frame on $S^2$. See Sect. 2.4.)

Viewing $S^3$ as the Hopf bundle we have a *U*(1) action on the $S^1$ fibres (corresponding to a rotation of the orthonormal frame in the tangent space of $S^2$). Introducing Euler coordinates^5^ $\left( \theta ,\phi ,\rho \right)$ on $S^3$ we denote this action by $e^{i\rho }$. Now any smooth function $F:S^3 \rightarrow {\mathbb {C}}$ which transforms as $F\left( p e^{i\rho }\right) = e^{-i\rho s} F\left( p\right)$ for $p \in S^3$ descends to a spin-weighted function on $S^2$ (by *choosing* a frame at each point). More precisely, *F* descends to a section of a complex line bundle over $S^2$ denoted traditionally by *B*(*R*). See [11, 27].

Let $Z_1, Z_2, Z_3$ be a basis of right invariant vector fields constituting a global orthonormal frame on $S^3$. In Euler coordinates we have the representation

27 $$\begin{aligned} Z_1&= -\sin \phi \partial _\theta + \cos \phi \left( \csc \theta \partial _\rho - \cot \theta \partial _\phi \right) \ , \nonumber \\ Z_2&= - \cos \phi \partial _\theta - \sin \phi \left( \csc \theta \partial _\rho - \cot \theta \partial _\phi \right) \ , \ Z_3 = \partial _\phi \, . \end{aligned}$$

A complex-valued function *F* of the Euler coordinates $\left( \theta ,\phi , \rho \right)$ is smooth on $S^3$ if for any $k_1,k_2,k_3 \in {\mathbb {N}} \cup \{0\}$ the functions $\left( Z_1\right) ^{k_1} \left( Z_2\right) ^{k_2} \left( Z_3\right) ^{k_3}F$ are smooth functions of the Euler coordinates and extend continuously to the poles of the coordinate system at $\theta =0$ and $\theta =\pi$.

Since spin *s*-weighted functions on $S^2$ arise from smooth functions on $S^3$ as discussed above, there is a natural notion of the space of smooth spin *s*-weighted functions on $S^2$: A complex-valued function *f* of the coordinates $(\theta ,\phi )$ is called a smooth spin *s*-weighted function on $S^2$ if for any $k_1,k_2,k_3 \in {\mathbb {N}} \cup \{0\}$ the functions $({\tilde{Z}}_1)^{k_1} ({\tilde{Z}}_2)^{k_2} ({\tilde{Z}}_3)^{k_3} f$ are smooth functions away from the poles and such that $e^{is\phi }({\tilde{Z}}_1)^{k_1} ({\tilde{Z}}_2)^{k_2} ({\tilde{Z}}_3)^{k_3} f$ extends continuously to the north ($\theta =0$) pole and $e^{-is\phi }({\tilde{Z}}_1)^{k_1} ({\tilde{Z}}_2)^{k_2} ({\tilde{Z}}_3)^{k_3} f$ extends continuously to the south ($\theta =\pi$) pole of the coordinate system, where

28 $$\begin{aligned} {\tilde{Z}}_1&= -\sin \phi \partial _\theta + \cos \phi \left( -is \csc \theta - \cot \theta \partial _\phi \right) \ , \nonumber \\ {\tilde{Z}}_2&= - \cos \phi \partial _\theta - \sin \phi \left( -is \csc \theta - \cot \theta \partial _\phi \right) \ , {\tilde{Z}}_3 = \partial _\phi \, . \end{aligned}$$

The space of smooth spin *s*-weighted functions on $S^2$ is denoted $\mathscr {S}^{[s]}_\infty$. Note that considered as usual functions on $S^2$, elements of $\mathscr {S}^{[s]}_\infty$ are in general not regular at $\theta =0$.

We define the Sobolev space of smooth spin *s*-weighted functions on $S^2$, denoted ${}^{[s]}H^m(\sin \theta d\theta d\phi )$ as the completion of $\mathscr {S}^{[s]}_\infty$ with respect to the norm.

$$\begin{aligned} \Vert f\Vert ^2_{{}^{[s]}H^m(\sin \theta d\theta d\phi )} = \sum _{i=0}^m \sum _{k_1+k_2+k_3=i} \int _{S^2} |({\tilde{Z}}_1)^{k_1} ({\tilde{Z}}_2)^{k_2} ({\tilde{Z}}_3)^{k_3} f|^2 \sin \theta d\theta d\phi \, . \end{aligned}$$

Note that the space $\mathscr {S}^{[s]}_{\infty }$ is dense in $L^2(\sin \theta d\theta d\phi )$.

We now define the analogous notions for functions *f* of the spacetime coordinates $\left( t^*,r,\theta ,\phi ^*\right)$.

We define a smooth complex-valued spin *s*-weighted function *f* on ${\mathcal {R}}$ to be a function $f: \left( -\infty ,\infty \right) \times \left[ 2M,\infty \right) \times \left( 0,\pi \right) \times \left[ 0, 2\pi \right)$ which is smooth in the sense that for any $k_1,k_2,k_3,k_4,k_5 \in {\mathbb {N}} \cup \{0\}$ the functions

$$\begin{aligned} ({\tilde{Z}}_1)^{k_1} ({\tilde{Z}}_2)^{k_2} ({\tilde{Z}}_3)^{k_3} \left( \partial _{t^*}\right) ^{k_4} \left( \partial _r\right) ^{k_5} f \end{aligned}$$

are smooth functions away from the poles and such that $e^{is\phi }(({\tilde{Z}}_1)^{k_1} ({\tilde{Z}}_2)^{k_2} ({\tilde{Z}}_3)^{k_3} \left( \partial _{t^*}\right) ^{k_4} \left( \partial _r\right) ^{k_5} f$ extends continuously to the north ($\theta =0$) pole and $e^{-is\phi }({\tilde{Z}}_1)^{k_1} ({\tilde{Z}}_2)^{k_2} ({\tilde{Z}}_3)^{k_3} \left( \partial _{t^*}\right) ^{k_4} \left( \partial _r\right) ^{k_5} f$ extends continuously to the south ($\theta =\pi$) pole. In particular, the restriction of *f* to fixed values of $t^*, r$ is a smooth spin *s*-weighted function on $S^2$. We denote the space of smooth complex-valued spin *s*-weighted functions on ${\mathcal {R}}$ by $\mathscr {S}_\infty ^{[s]}({\mathcal {R}})$.

We similarly define a smooth complex-valued spin *s*-weighted function *f* on a slice ${\Sigma }_\tau$ to be a function $f: \left[ 2M,\infty \right) \times \left( 0,\pi \right) \times \left[ 0, 2\pi \right)$ which is smooth in the sense that for any $k_1,k_2,k_3,k_4 \in {\mathbb {N}} \cup \{0\}$ the functions

$$\begin{aligned} ({\tilde{Z}}_1)^{k_1} ({\tilde{Z}}_2)^{k_2} ({\tilde{Z}}_3)^{k_3} \left( \partial _{r}\right) ^{k_4} f \end{aligned}$$

are smooth functions away from the poles and such that $e^{\pm is\phi } ({\tilde{Z}}_1)^{k_1} ({\tilde{Z}}_2)^{k_2} ({\tilde{Z}}_3)^{k_3} \left( \partial _{r}\right) ^{k_4} f$ extends continuously to $\theta =0$ and $\theta =\pi$ respectively. The space of such functions is denoted $\mathscr {S}_\infty ^{[s]}(\Sigma _{\tau })$. The Sobolev space ${}^{[s]}H^m(\Sigma _{\tau })$ is defined as the completion of $\mathscr {S}_\infty ^{[s]}(\Sigma _{\tau })$ with respect to the norm

$$\begin{aligned} \Vert f\Vert ^2_{{}^{[s]}H^m(\Sigma _{\tau })} = \sum _{i=0}^m \sum _{k_1+k_2+k_3+k_4=i} \int _{\Sigma _\tau } dV_{\Sigma _{\tau }} |({\tilde{Z}}_1)^{k_1} ({\tilde{Z}}_2)^{k_2} ({\tilde{Z}}_3)^{k_3} \left( \partial _r\right) ^{k_4} f|^2 \, . \end{aligned}$$

If ${\mathcal {U}}$ is an open subset of $\Sigma _\tau$ we can define $\mathscr {S}_\infty ^{[s]}({\mathcal {U}})$ and ${}^{[s]}H^m({\mathcal {U}})$ in the obvious way. This allows to define the space ${}^{[s]}H^m_{\mathrm{loc}} \left( \Sigma _\tau \right)$ as the space of functions on $\Sigma _{\tau }$ such that the restriction to any ${\mathcal {U}} \Subset \Sigma _\tau$ (meaning that there is a compact set *K* with ${\mathcal {U}} \subset K \subset \Sigma _\tau$) is in ${}^{[s]}H^m \left( {\mathcal {U}}\right)$.

We finally note that we can analogously define these spaces for the slices $\widetilde{\Sigma }_{\tau }$, i.e. define the spaces

$$\begin{aligned} \mathscr {S}_\infty ^{[s]}(\widetilde{\Sigma }_{\tau }) \ \ , \ \ {}^{[s]}H^m(\widetilde{\Sigma }_{\tau }) \ \ , \ \ {}^{[s]}H_{loc}^m(\widetilde{\Sigma }_{\tau }) \, . \end{aligned}$$

#### The Spin *s*-Weighted Laplacian

Let us note that the operator defined in the introduction,29is a smooth operator on $\mathscr {S}^{[s]}_\infty$. Indeed, a computation yields . Note also the formula $\sum _{i=1}^3 | {\tilde{Z}}_i \Xi |^2 = |\partial _{\theta } \Xi |^2 + \frac{1}{\sin ^2 \theta } | is \Xi \cos \theta + \partial _\phi \Xi |^2 +s^2 |\Xi |^2$.

The eigenfunctions of  are again in $\mathscr {S}^{[s]}_\infty$ and are known as *s*-spin weighted spherical harmonics. We shall discuss these (and their twisted analogues) further in Sect. 6.2.1.

An integration by parts yields for $\Xi \in \mathscr {S}^{[s]}_\infty$30where the right hand side is manifestly non-negative.^6^ Introducing the spinorial gradientand defining31we also have32We note that for $\Xi , \Pi \in \mathscr {S}^{[s]}_\infty$33Directly from (30) and (32) we deduce the Poincaré inequality34Combining (32) and (34) we also deduce35


#### The Teukolsky Operator for General Spin *s*

Recall that the operator

36 $$\begin{aligned} {\mathfrak {T}}^{[s]} {\upalpha }^{[s]}= & {} \Box _g {\upalpha }^{[s]} +\frac{2s}{\rho ^2}(r-M)\partial _r {\upalpha }^{[s]} +\frac{2s}{\rho ^2} \left( \frac{a(r-M)}{\Delta } +i\frac{\cos \theta }{\sin ^2\theta }\right) \partial _\phi {\upalpha }^{[s]}\nonumber \\&+\frac{2s}{\rho ^2}\left( \frac{M(r^2-a^2)}{\Delta }-r-ia\cos \theta \right) \partial _t {\upalpha }^{[s]} +\frac{1}{\rho ^2}(s-s^2\cot ^2\theta ) {\upalpha }^{[s]}\nonumber \\ \end{aligned}$$

is the traditional representation (see for instance [99]) of the Teukolsky operator with spin $s\in \frac{1}{2}{\mathbb {Z}}$. In view of the comments above, this operator is smooth on $\mathscr {S}_\infty ^{[s]}({\mathcal {R}}\setminus {\mathcal {H}}^+)$. We will say that such an $\upalpha ^{[s]}\in \mathscr {S}_\infty ^{[s]}({\mathcal {R}}\setminus {\mathcal {H}}^+)$ satisfies the Teukolsky equation if the following holds:

37 $$\begin{aligned} {\mathfrak {T}}^{[s]} {\upalpha }^{[s]}=0. \end{aligned}$$

The operator (37) is not smooth on $\mathscr {S}_\infty ^{[s]}({\mathcal {R}})$ itself. This is because it has been derived with respect to a choice of frame which degenerates at the horizon. See Sect. 2.4. To obtain a regular equation at the horizon, we must considered rescaled quantities. We turn to this now.

#### Rescaled Equations

To understand regularity issues at the horizon we must consider rescaled quantities. We will restrict here to $s=\pm \,2$.

Define

38 $$\begin{aligned} {\tilde{\upalpha }}^{[+2]} = \Delta ^2 (r^2+a^2)^{-\frac{3}{2}}\upalpha ^{[+2]}, \qquad {\tilde{\upalpha }}^{[-2]} = \Delta ^{-2}(r^2+a^2)^{-\frac{3}{2}}\upalpha ^{[-2]} \, . \end{aligned}$$

Define now the modified Teukolsky operator $\widetilde{{\mathfrak {T}}}^{[s]}$ by the relation39with  denoting the spin $\pm \, 2$ weighted Laplacian on the round sphere defined in (29) and with the first order term ${\mathfrak {t}}^{[s]}$ given by

40 $$\begin{aligned} {\mathfrak {t}}^{[+2]}&= -\,2\frac{w^\prime }{w} {\underline{L}} - 8 a w\frac{r}{r^2+a^2} \Phi \ \ \ \text {and} \ \ \ {\mathfrak {t}}^{[-2]} = +2\frac{w^\prime }{w} {L} + 8 aw \frac{r}{r^2+a^2} \Phi \ \ \ \text {where} \ \ \nonumber \\ w&:= \frac{\Delta }{\left( r^2+a^2\right) ^2}. \end{aligned}$$

One sees that (37) for $s=+\,2$ can be rewritten as

41 $$\begin{aligned} \widetilde{{\mathfrak {T}}}^{[+2]} {\tilde{\upalpha }}^{[+2]} =0. \end{aligned}$$

On the other hand, we observe that $\widetilde{{\mathfrak {T}}}^{[+2]}$ now is a smooth operator on $\mathscr {S}_\infty ^{[s]}({\mathcal {R}})$ and that its second order part is hyperbolic, in fact, it is exactly equal to $-\Box _g$.

Similarly, we see that (37) for $s=-2$ can be rewritten as

42 $$\begin{aligned} \widetilde{{\mathfrak {T}}}^{[-2]} \left( \Delta ^{2} {\tilde{\upalpha }}^{[-2]} \right) =0 \, , \end{aligned}$$

which in turn can be rewritten as

43 $$\begin{aligned} \left[ \widetilde{{\mathfrak {T}}}^{[-2]} -2\frac{\rho ^2}{\Delta }\frac{w^\prime }{w} L +{\mathfrak {t}}^{[-2]}_{aux}\right] {\tilde{\upalpha }}^{[-2]} = 0 \, , \end{aligned}$$

where

$$\begin{aligned} {\mathfrak {t}}^{[-2]}_{aux} = \frac{\rho ^2}{\Delta }\left[ - 4 \frac{\left( r^2+a^2\right) ^\prime }{(r^2+a^2)}L+2\frac{\Delta ^\prime }{\Delta }{\underline{L}} -2\left( \frac{\Delta ^\prime }{\Delta }\right) ^\prime +8 \frac{\left( r^2+a^2\right) ^\prime }{(r^2+a^2)} \frac{\Delta ^\prime }{\Delta }\right] \end{aligned}$$

is a first order operator acting smoothly on $\mathscr {S}_\infty ^{[s]}({\mathcal {R}})$. Now we observe that $\widetilde{{\mathfrak {T}}}^{[-2]} -2\frac{\rho ^2}{\Delta }\frac{w^\prime }{w} L$ also acts smoothly on $\mathscr {S}_\infty ^{[s]}({\mathcal {R}})$ and that its second order part is exactly equal to $-\Box _g$. This will allow us to state a well-posedness proposition in the section to follow.

##### Remark 2.1

The weights in (38) for $\widetilde{\upalpha }^{[+2]}$ will be useful for the global analysis of the equation, whereas the weights for $\widetilde{\upalpha }^{[-2]}$ will only be useful for the well-posedness below. For this reason, we shall define later (see Sect. 6.2.5) the different rescaled quantities $u^{[\pm 2]} = \Delta ^{\pm 1}\sqrt{r^2+a^2}\upalpha ^{[\pm 2]}$, and deal mostly with the further rescaled quantities $u^{[\pm 2]}\cdot w$. Note that

$$\begin{aligned} u^{[+2]}\cdot w= \widetilde{\upalpha }^{[+2]}, \qquad \mathrm{but}\qquad u^{[-2]}\cdot w= (r^2+a^2)^{-\frac{3}{2}}\upalpha ^{[-2]} . \end{aligned}$$

The first quantity is finite (and generically non-zero) on the horizon ${\mathcal {H}}^+$ while the second quantity is finite (and generically non-zero) on null infinity ${\mathcal {I}}^+$ which makes them useful in the global considerations below. Note also that both quantities satisfy the simple equations (41) and (42) respectively.

### Well-posedness

Standard theory yields that the Teukolsky equation in the form (41), (43) is well-posed on ${\mathcal {R}}_0$ or $\widetilde{{\mathcal {R}}}_0$ with initial data $({\tilde{\upalpha }}^{[s]}_0,{\tilde{\upalpha }}^{[s]}_1)$ defined on $\Sigma _0$ in ${}^{[s]}H^j_{\mathrm{loc}}(\Sigma _0)\times {}^{[s]}H^{j-1}_{\mathrm{loc}}(\Sigma _0)$, resp. with $\widetilde{\Sigma }_0$ replacing $\Sigma _0$. We state this as a proposition for reference:

#### Proposition 2.3.1

(Well-posedness) For $s=\pm \, 2$, let $({\tilde{\upalpha }}^{[s]}_0, {\tilde{\upalpha }}^{[s]}_1)\in {}^{[s]}H^j_{\mathrm{loc}}(\Sigma _0)\times {}^{[s]}H^{j-1}_{\mathrm{loc}}(\Sigma _0)$ be complex valued spin weighted functions with $j\ge 1$. Then there exists a unique complex valued ${\tilde{\upalpha }}^{[s]}$ on ${\mathcal {R}}_0$ satisfying (41) (equivalently $\upalpha ^{[s]}$ satisfying (37)) with ${\tilde{\upalpha }}^{[s]} \in {}^{[s]}H^{j}_{\mathrm{loc}} (\Sigma _\tau )$, $n_{\Sigma _{\tau }} {\tilde{\upalpha }}^{[s]} \in {}^{[s]}H^{j-1}_{\mathrm{loc}}(\Sigma _\tau )$ such that ${\tilde{\upalpha }}^{[s]}\big |_{\Sigma _0}={\tilde{\upalpha }}^{[s]}_0$, $(n_{\Sigma _0}{\tilde{\upalpha }}^{[s]})\big |_{\Sigma _0}=\upalpha ^{[s]}_1$. In particular, if $({\tilde{\upalpha }}^{[s]}_0, {\tilde{\upalpha }}^{[s]}_1)\in \mathscr {S}^{[s]}_{\infty }(\Sigma _0)$ then ${\tilde{\upalpha }}^{[s]}\in \mathscr {S}^{[s]}_{\infty }({\mathcal {R}}_0)$.

The same statement holds with $\widetilde{\Sigma }_0$, $\widetilde{\Sigma }_\tau$, $\widetilde{{\mathcal {R}}}_0$ in place of $\Sigma _0$, $\Sigma _\tau$, ${\mathcal {R}}_0$, respectively.

#### Proof

cf. Proposition 4.5.1 of [40]. $\square$

### Relation with the System of Gravitational Perturbations

The Teukolsky equation (2) is traditionally derived via the Newman–Penrose formalism [93]. One defines the (complex) null tetrad $\left( \ell , n, m, {\bar{m}}\right)$ by

44 $$\begin{aligned} l&= \frac{r^2+a^2}{\Delta } L \ \ \ , \ \ \ n = \frac{r^2+a^2}{2\rho ^2} {\underline{L}} \ \ \ , \ \ \nonumber \\ m&= \frac{1}{\sqrt{2}(r+ia \cos \theta )} \left( ia\sin \theta \partial _t + \partial _{\theta } + \frac{i}{\sin \theta } \partial _\phi \right) \, , \end{aligned}$$

which is normalised such that

$$\begin{aligned} g\left( l,n\right) = - \,1 \ \ \ , \ \ \ g \left( m, {\bar{m}}\right) = 1 \ \ \ , \ \ \ g\left( m,m\right) = g\left( {\bar{m}},{\bar{m}}\right) = 0 \, . \end{aligned}$$

Note that we can obtain an associated real spacetime null frame $\left( \ell , n, e_1,e_2\right)$ by defining $e_1 = \frac{1}{\sqrt{2}} \left( m + {\bar{m}}\right)$ and $e_2 = \frac{1}{\sqrt{2}i} \left( m - {\bar{m}}\right)$, which then satisfies in particular $g\left( e_1,e_1\right) = g\left( e_2,e_2\right) =1$ and $g\left( e_1,e_2\right) =0$.

The extremal Newman–Penrose curvature scalars are defined as the following components of the spacetime Weyl tensor^7^

45 $$\begin{aligned} {\varvec{\Psi }}_0 = - W \left( l,m,l,m\right) \ \ \ , \ \ \ {\varvec{\Psi }}_4 = -\, W \left( n, {\overline{m}}, n, {\overline{m}}\right) \, . \end{aligned}$$

Both ${\varvec{\Psi }}_0$ and ${\varvec{\Psi }}_4$ vanish for the exact Kerr metric. Remarkably, upon linearising the Einstein vacuum equations (1) (using the above frame) the linearised components ${\varvec{\Psi }}_0$ and ${\varvec{\Psi }}_4$ are gauge invariant (with respect to infinitesimal changes of both the frame and the coordinates) and moreover satisfy decoupled equations. Indeed, one may check that $\upalpha ^{[-2]}= \left( r-ia\cos \theta \right) ^4{\varvec{\Psi }}_4$ and $\upalpha ^{[+2]}= {\varvec{\Psi }}_0$ satisfy precisely the Teukolsky equation (2) for $s=-2$ and $s=2$ respectively.

Instead of defining spin *s*-weighted complex functions ${\varvec{\Psi }}_0$, ${\varvec{\Psi }}_4$ one may (equivalently) define symmetric traceless 2-tensors $\alpha$ and $\underline{\alpha }$ (living in an appropriate bundle of *horizontal* tensors) by

$$\begin{aligned} \alpha \left( e_A, e_B\right) = W \left( L,e_A, L, e_B\right) \ \ \ , \ \ \ \underline{\alpha } \left( e_A, e_B\right) = W \left( {\underline{L}},e_A, {\underline{L}}, e_B\right) \, . \end{aligned}$$

Using the symmetry and the trace properties of the Weyl tensor we derive the relations

$$\begin{aligned} \underline{\alpha } \left( e_1,e_1\right) =-\underline{\alpha } \left( e_2,e_2\right) = -\frac{1}{2} \left( \frac{2\rho ^2}{r^2+a^2}\right) ^2 \left( {\varvec{\Psi }}_4 + \overline{{\varvec{\Psi }}_4}\right) \end{aligned}$$

and

$$\begin{aligned} \underline{\alpha } \left( e_1,e_2\right) = \underline{\alpha } \left( e_2,e_1\right) = +\frac{1}{2}i\left( \frac{2\rho ^2}{r^2+a^2}\right) ^2 \left( {\varvec{\Psi }}_4 - \overline{{\varvec{\Psi }}_4}\right) \, , \end{aligned}$$

which relate the spin 2-weighted complex function and the tensorial version of the curvature components. Of course similar formulae are easily derived for $\alpha$.

We can now connect directly to our previous [31] where we wrote down the Teukolsky equation for the symmetric traceless tensors $\alpha$ and $\underline{\alpha }$ in the Schwarzschild spacetime.

As a final remark we note that in the Schwarzschild case considered in [31] the null frame used to define the extremal Weyl components arose directly from a double null foliation of the spacetime. In stark contrast, the algebraically special null frame $\left( l, n, e_1,e_2\right)$ in Kerr for $a\ne 0$ does *not* arise from a double null foliation of that spacetime.

## Generalised Chandrasekhar Transformations for $s=\pm \, 2$

In this section, we generalise the physical space reformulations of Chandrasekhar’s transformations, given in [31], to Kerr.

In accordance with the conventions of our present paper, we will consider complex scalar spin $\pm \,2$ weighted quantities $\upalpha ^{[\pm 2]}$ in place of the tensorial ones of [31]. We begin in Sect. 3.1 with the definitions of the quantities $P^{[\pm 2]}$ associated to quantities $\upalpha ^{[\pm 2]}$. If $\upalpha ^{[\pm 2]}$ satisfy the (inhomogeneous) Teukolsky equation, then we show in Sect. 3.2 that $P^{[\pm 2]}$ will satisfy an (inhomogeneous) Regge–Wheeler type equation, coupled to $\upalpha ^{[\pm 2]}$. The latter coupling vanishes in the Schwarzschild case. The precise relation with the tensorial definitions of [31] will be given in Sect. 3.3.

### The Definitions of $P^{[\pm 2]}, \Psi ^{[\pm 2]}$ and $\uppsi ^{[\pm 2]}$

Given functions $\upalpha ^{[\pm 2]}$, we define

46 $$\begin{aligned} P^{[+2]}= & {} -\frac{(r^2+a^2)^{1/2}}{2\Delta } {\underline{L}}^\mu \nabla _\mu \left( \frac{(r^2+a^2)^2}{\Delta } {\underline{L}}^\mu \nabla _\mu \left( \Delta ^2 \left( r^2+a^2\right) ^{-\frac{3}{2}} \upalpha ^{[+2]}\right) \right) ,\nonumber \\ \end{aligned}$$

47 $$\begin{aligned} P^{[-2]}= & {} -\frac{(r^2+a^2)^{1/2}}{2\Delta } {L}^\mu \nabla _\mu \left( \frac{(r^2+a^2)^2}{\Delta } {L}^\mu \nabla _\mu \left( \left( r^2+a^2\right) ^{-\frac{3}{2}} \upalpha ^{[-2]}\right) \right) . \end{aligned}$$

These are our physical-space generalisations to Kerr of Chandrasekhar’s fixed frequency Schwarzschild transformation theory.

Note that if $\widetilde{\upalpha }^{[\pm 2]}\in \mathscr {S}^{[\pm 2]}_\infty ({\mathcal {U}})$ for ${\mathcal {U}}\subset {\mathcal {R}}$, then $P^{[\pm 2]}\in \mathscr {S}^{[\pm 2]}_{\infty }({\mathcal {U}})$. We will typically work with the rescaled functions

48 $$\begin{aligned} \Psi ^{[\pm 2]} = (r^2+a^2)^{\frac{3}{2}} P^{[\pm 2]}, \end{aligned}$$

which are of course again smooth.

As in [31], it will be again useful to give a name to the intermediate quantities $\uppsi ^{[\pm 2]}$ defined by

49 $$\begin{aligned} \uppsi ^{[+2]}= & {} -\frac{1}{2} \Delta ^{-\frac{3}{2}} \left( r^2+a^2\right) ^{+2} {\underline{L}}^\mu \nabla _\mu (\Delta ^2 \left( r^2+a^2\right) ^{-\frac{3}{2}} \upalpha ^{[+2]}) \end{aligned}$$

50 $$\begin{aligned} \uppsi ^{[-2]}= & {} +\frac{1}{2}\Delta ^{-\frac{3}{2}}(r^2+a^2)^2 L^\mu \nabla _\mu \left( \upalpha ^{[-2]}(r^2+a^2)^{-\frac{3}{2}}\right) . \end{aligned}$$

We can rewrite (46)–(47) as

51 $$\begin{aligned} {\underline{L}}^\mu \nabla _\mu \left( \sqrt{\Delta }{\uppsi }^{[+2]} \right)&= \Delta (r^2+a^2)^{-2} \Psi ^{[+2]}, \end{aligned}$$

52 $$\begin{aligned} L^\mu \nabla _\mu (\sqrt{\Delta } \uppsi ^{[-2]})&= - \Delta (r^2+a^2)^{-2}\Psi ^{[-2]}. \end{aligned}$$

Note that for $\widetilde{\upalpha }^{[\pm 2]}$ smooth, it is the quantities $\sqrt{\Delta }\uppsi ^{[+2]}$, $(\sqrt{\Delta })^{-1}\uppsi ^{[-2]}$ which are smooth.

### The Generalised Inhomogeneous Regge–Wheeler-Type Equation with Error

The importance of the quantities $\Psi ^{[\pm 2]}$ arises from the following fundamental proposition:

#### Proposition 3.2.1

If $\upalpha ^{[\pm 2]}$ satisfy the inhomogeneous equations

53 $$\begin{aligned} \widetilde{{\mathfrak {T}}}^{[+2]} \left( \tilde{\upalpha }^{[+2]}\right) = F^{[+2]} \ \ \ \text {and} \ \ \ \widetilde{{\mathfrak {T}}}^{[-2]} \left( \Delta ^2 \tilde{\upalpha }^{[-2]}\right) = \Delta ^2 F^{[-2]} \end{aligned}$$

then the quantities $\Psi ^{[\pm 2]}$ satisfy the equation

54 $$\begin{aligned} {\mathfrak {R}}^{[\pm 2]}\Psi ^{[\pm 2]}= -\frac{\rho ^2}{\Delta } {\mathcal {J}}^{[\pm 2]} - \frac{\rho ^2}{\Delta } {\mathfrak {G}}^{[\pm 2]} \end{aligned}$$

where55
56and

57 $$\begin{aligned} {\mathcal {J}}^{[-2]} {=}&\frac{\Delta }{\left( r^2+a^2\right) ^2} \left[ \frac{8r^2-8a^2}{r^2+a^2} a \Phi {-}20a^2 \frac{r^3-3Mr^2+ra^2+Ma^2}{\left( r^2+a^2\right) ^2} \right] \left( \sqrt{\Delta }\uppsi ^{[-2]} \right) \nonumber \\&+a^2 \frac{\Delta }{\left( r^2+a^2\right) ^2} \left[ +12 \frac{r}{r^2+a^2} a \Phi +3 \left( \frac{r^4 -a^4+10Mr^3-6Ma^2r}{(r^2+a^2)^2}\right) \right] \nonumber \\&\times \left( \upalpha ^{[-2]} \left( r^2+a^2\right) ^{-\frac{3}{2}}\right) \,, \end{aligned}$$

58 $$\begin{aligned} {\mathfrak {G}}^{[- 2]}=&\frac{1}{2} {L} \left( \frac{\left( r^2+a^2\right) ^2}{\Delta }{L} \left( \frac{\Delta ^3}{w\rho ^2} F^{[-2]}\right) \right) . \end{aligned}$$

#### Proof

Direct calculation. See Appendix. $\square$

We will call the operator ${\mathfrak {R}}^{[s]}$ defined by (55) the *generalised Regge–Wheeler operator*. We note that it has smooth coefficients on ${\mathcal {R}}_0$ and its highest order part is proportional to the wave operator. The equation (54) reduces to the usual Regge–Wheeler equation in the case $a=0$:

#### Corollary 3.1

If $a=0$ and $F^{[\pm 2]}=0$ then $\Psi ^{[\pm 2]}$ satisfies the Regge–Wheeler equation59where $\Omega ^2 = 1- \frac{2M}{r}$.

As discussed already in the introduction, we see that (54), although still coupled to $\upalpha ^{[\pm 2]}$, retains some of the good structure of (59). The operator (54) has a good divergence structure admitting estimates via integration by parts, i.e. it does not have the problematic first order terms of the Teukolsky operator $\widetilde{{\mathfrak {T}}}^{[\pm 2]}$, cf. (39). See already the divergence identities of Sect. 5.1.1. Moreover, the terms ${\mathcal {J}}^{[+2]}$ can be thought of as lower order, from the perspective of $\Psi ^{[\pm 2]}$, as they only involve up to second derivatives of $\upalpha ^{[\pm 2]}$ (via the term $\Phi (\sqrt{\Delta }\uppsi ^{[\pm 2]})$).

### Relation with the Quantities *P* and ${\underline{P}}$ of [31]

As with the tensorial quantities $$\alpha $$ and $$\underline{\alpha }$$ discussed in Sect. 2.4, in [31] the transformations to the quantities *P* and $${\underline{P}}$$ (corresponding to the complex functions $$ P^{[+2]}$$, $$ {P}^{[-2]}$$ in this paper) were again given tensorially. In particular, the Regge–Wheeler equation for the symmetric traceless tensor $$\Psi =r^5 P$$ was written tensorially using projected covariant derivatives as (cf. Corollary 7.1 of [31])60where  and  are projected (to the spheres of symmetry) covariant derivatives in the null directions,  is the covariant Laplacian associated with the metric on the spheres of symmetry acting on symmetric traceless tensors and $$\Omega ^2 = 1- \frac{2M}{r}$$. Note that unlike the operator  considered in this paper, the operator  was defined as a *negative* operator in [31].

Computing the equation satisfied by the components of $\Psi$ in the standard orthonormal frame on the spheres of symmetry one obtainsfrom which one infers that the complex-valued functions $\Psi ^{[\pm 2]}= \Psi _{11} \mp i \Psi _{12}$ satisfy the Regge–Wheeler equation (59) for $s=\pm \, 2$.^8^

## Energy Quantities and Statement of the Main Theorem

We first give certain definitions of weighted energy quantities in Sect. 4.1. This will allow us to give a precise statement of the main theorem of this paper (Theorem 4.1) in Sect. 4.2. We will finally discuss in Sect. 4.3 how the logic of the proof of Theorem 4.1 is represented by the sections that follow.

### Definitions of Weighted Energies

We will define in this section a number of weighted energies. In addition to those appearing in the statement of Theorem 4.1, we will need to consider various auxiliary quantities.

#### The Left, Right and Trapped Subregions

We will in particular need to introduce energies localised to various subregions of $\widetilde{\Sigma }_\tau$ and $\widetilde{{\mathcal {R}}}(\tau _1, \tau _2)$. In anticipation of this, let us define the following subregions

$$\begin{aligned} \widetilde{{\mathcal {R}}}^{\mathrm{left}}(\tau _1, \tau _2)= & {} \widetilde{{\mathcal {R}}}(\tau _1, \tau _2)\cap \{r\le A_1\}, \qquad \widetilde{{\mathcal {R}}}^{\mathrm{right}}(\tau _1, \tau _2) = \widetilde{{\mathcal {R}}}(\tau _1, \tau _2)\cap \{r\ge A_2\},\\&\widetilde{{\mathcal {R}}}^{\mathrm{away}}(\tau _1, \tau _2) = \widetilde{{\mathcal {R}}}^{\mathrm{left}}(\tau _1, \tau _2)\cup \widetilde{{\mathcal {R}}}^{\mathrm{right}}(\tau _1, \tau _2)\\&\widetilde{{\mathcal {R}}}^{\mathrm{trap}}(\tau _1, \tau _2)= \widetilde{{\mathcal {R}}}(\tau _1, \tau _2)\cap \{A_1\le r\le A_2\}. \end{aligned}$$

Note that

$$\begin{aligned} \widetilde{{\mathcal {R}}}^{\mathrm{trap}}(\tau _1, \tau _2) \cup \widetilde{{\mathcal {R}}}^{\mathrm{away}}(\tau _1, \tau _2)= & {} \widetilde{{\mathcal {R}}}^{\mathrm{trap}}(\tau _1, \tau _2) \cup \widetilde{{\mathcal {R}}}^{\mathrm{left}}(\tau _1, \tau _2)\cup \widetilde{{\mathcal {R}}}^{\mathrm{right}}(\tau _1, \tau _2)\\= & {} \widetilde{{\mathcal {R}}}(\tau _1, \tau _2). \end{aligned}$$

For $\widetilde{\Sigma }_\tau$, it will be more natural to consider

$$\begin{aligned}&\displaystyle \widetilde{\Sigma }_\tau ^{\mathrm{left}}= \widetilde{\Sigma }_\tau \cap \{r\le A_1\}, \qquad \widetilde{\Sigma }_\tau ^{\mathrm{right}}= \widetilde{\Sigma }_\tau \cap \{r\ge A_2\}, \qquad \widetilde{\Sigma }_\tau ^{\mathrm{away}}=\widetilde{\Sigma }_\tau ^{\mathrm{left}}\cup \widetilde{\Sigma }_\tau ^{\mathrm{right}}, \end{aligned}$$

See Fig. 2.

**Fig. 2:**
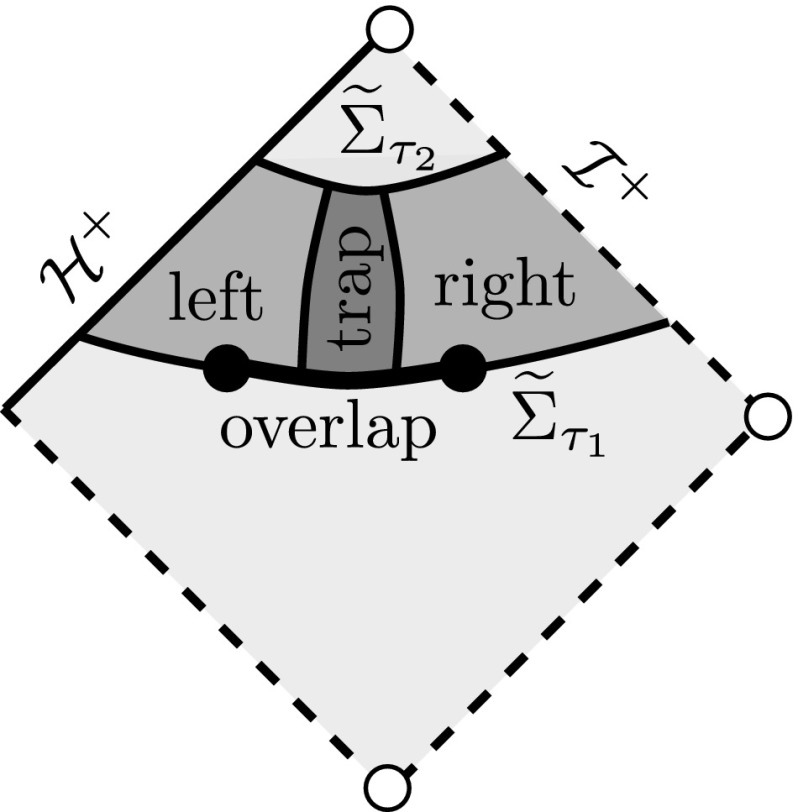
Partitioning $$\widetilde{{\mathcal {R}}}(\tau _1, \tau _2)$$ and $$\widetilde{\Sigma }_\tau $$

#### Weighted Energies for $\Psi ^{[\pm 2]}$

The energies in this section will in general be applied to $\Psi ^{[\pm 2]}$ satisfying the inhomogeneous equation (54).

Let *p* be a free parameter (which will eventually always take the values $0, \eta , 1$ or 2). We define the following weighted energies on the slices ${\widetilde{\Sigma }}_{\tau }$61We remark that an overbar indicates that the energy has optimised weights near the horizon.

We will also consider the following energy through ${\widetilde{\Sigma }}_{\tau }^{\mathrm{away}}$:On the event horizon ${\mathcal {H}}^+$ we define the energies62On null infinity ${\mathcal {I}}^+$ we define the energiesIn addition to the energy fluxes, we will define the weighted spacetime energies63
64where $\varvec{\delta }^a_b$ is the Kronecker delta symbol and also the degenerate spacetime energies65with $\tilde{\chi }$ a radial cut-off function equal to 1 in $r^*\in (-\infty , A_1^*] \cup [A_2^*,\infty )$ and vanishing in $r^*\in \left[ A_1^*/4,A_2^*/4\right]$. Finally, we shall defineand66Note that

$$\begin{aligned} {{\mathbb {I}}}^{\mathrm{deg}}_p \left[ \Psi ^{[\pm 2]}\right] (\tau _1,\tau _2) \lesssim {{\mathbb {I}}}^{\mathrm{away}}_p \left[ \Psi ^{[\pm 2]}\right] \left( \tau _1,\tau _2\right) + {\mathbb {I}}^{\mathrm{trap}}[\Psi ^{[\pm 2]}](\tau _1,\tau _2). \end{aligned}$$

#### Weighted Energies for $\upalpha ^{[+2]}, \uppsi ^{[+2]}$

The quantities in this section will in general be applied to $\upalpha ^{[+2]}$, $\uppsi ^{[+2]}$ arising from a solution ${\tilde{\upalpha }}^{[+2]}$ of the inhomogeneous equation (53).

We define the following energy densities

67 $$\begin{aligned} e_p \left[ \upalpha ^{[+2]}\right]&= \sum _{\Gamma \in \{id, \Phi \}} \Big | \Gamma \left( \upalpha ^{[+2]} \Delta ^2 \left( r^2+a^2\right) ^{-1}\right) \Big |^2 r^{-\varvec{\delta }^p_2\eta } r^p\nonumber \\&\quad + \Big |T\left( \upalpha ^{[+2]} \Delta ^2 \left( r^2+a^2\right) ^{-1}\right) \Big |^2 r^{2-\eta } \, , \end{aligned}$$

68 $$\begin{aligned} e_p \left[ \uppsi ^{[+2]}\right]&= \sum _{\Gamma \in \{id, \Phi \}} \Big | \Gamma \left( \uppsi ^{[+2]} \sqrt{\Delta }\right) \Big |^2 r^{-\varvec{\delta }^p_2\eta } r^p + \Big |T\left( \uppsi ^{[+2]} \sqrt{\Delta }\right) \Big |^2 r^{2-\eta } \, . \end{aligned}$$

With these, we define the following weighted energies on the slices ${\widetilde{\Sigma }}_{\tau }$:

69 $$\begin{aligned} {\mathbb {E}}_{{\widetilde{\Sigma }}_{\tau },p} \left[ \upalpha ^{[+2]}\right] \left( \tau \right)&= \int _{{\widetilde{\Sigma }}_{\tau }} dr d\sigma \ e_p \left[ \upalpha ^{[+2]}\right] , \nonumber \\ {\mathbb {E}}_{{\widetilde{\Sigma }}_{\tau },p} \left[ \uppsi ^{[+2]}\right] \left( \tau \right)&= \int _{{\widetilde{\Sigma }}_{\tau }} dr d\sigma \ e_p \left[ \uppsi ^{[+2]}\right] \, . \end{aligned}$$

##### Remark 4.1

We remark already that while these energies contain the *T* and the $\Phi$ derivative only, we can obtain also the *L* and the ${\underline{L}}$ derivative if we control in addition the energy (61) of $\Psi ^{[+2]}$. This is because of the relations (107) and (108) and the relation $L = -{\underline{L}} + 2T + \frac{2a}{r^2+a^2}\Phi$.

It will be useful to also consider separately

70 $$\begin{aligned} {\mathbb {E}}^{\mathrm{left}}_{{\widetilde{\Sigma }}_{\tau },p} \left[ \upalpha ^{[+2]}\right] \left( \tau \right)&= \int _{{\widetilde{\Sigma }}_{\tau }^{\mathrm{left}}} dr d\sigma e_p \left[ \upalpha ^{[+2]}\right] \, , \nonumber \\ {\mathbb {E}}^{\mathrm{left}}_{{\widetilde{\Sigma }}_{\tau },p} \left[ \uppsi ^{[+2]}\right] \left( \tau \right)&= \int _{{\widetilde{\Sigma }}_{\tau }^{\mathrm{left}}} dr d\sigma \ e_p \left[ \uppsi ^{[+2]}\right] \, , \end{aligned}$$

71 $$\begin{aligned} {\mathbb {E}}^{\mathrm{right}}_{{\widetilde{\Sigma }}_{\tau },p} \left[ \upalpha ^{[+2]}\right] \left( \tau \right)&= \int _{{\widetilde{\Sigma }}_{\tau }^{\mathrm{right}}} dr d\sigma \ e_p \left[ \upalpha ^{[+2]}\right] \, , \nonumber \\ {\mathbb {E}}^{\mathrm{right}}_{{\widetilde{\Sigma }}_{\tau },p} \left[ \uppsi ^{[+2]}\right] \left( \tau \right)&= \int _{{\widetilde{\Sigma }}_{\tau }^{\mathrm{right}}} dr d\sigma \ e_p \left[ \uppsi ^{[+2]}\right] \, . \end{aligned}$$

We also use the notation ${\mathbb {E}}^{\mathrm{away}}_{{\widetilde{\Sigma }}_{\tau },p}$ for the sum of the left and the right energies. On (timelike) hypersurfaces of constant $r=A>r_+$ we define

72 $$\begin{aligned} {\mathbb {E}}_{r=A} \left[ \upalpha ^{[+2]}\right] \left( \tau _1,\tau _2\right)&= \int _{\tau _1}^{\tau _2} d\tau d\sigma \ e_p \left[ \upalpha ^{[+2]}\right] \Bigg |_{r=A} \ , \nonumber \\ {\mathbb {E}}_{r=A} \left[ \uppsi ^{[+2]}\right] \left( \tau _1,\tau _2\right)&=\int _{\tau _1}^{\tau _2} d\tau d\sigma \ e_p \left[ \uppsi ^{[+2]}\right] \Bigg |_{r=A} . \end{aligned}$$

In the limit $r \rightarrow r_+$ we obtain the energies the event horizon ${\mathcal {H}}^+$ which we denote

73 $$\begin{aligned} {\mathbb {E}}_{{\mathcal {H}}^+} \left[ \upalpha ^{[+2]}\right] \left( \tau _1,\tau _2\right)&= \int _{\tau _1}^{\tau _2} d\tau d\sigma \ e_p \left[ \upalpha ^{[+2]}\right] \Bigg |_{r=r_+} \, , \,\nonumber \\ {\mathbb {E}}_{{\mathcal {H}}^+} \left[ \uppsi ^{[+2]}\right] \left( \tau _1,\tau _2\right)&=\int _{\tau _1}^{\tau _2} d\tau d\sigma \ e_p \left[ \uppsi ^{[+2]}\right] \Bigg |_{r=r_+} \, . \end{aligned}$$

We also define the following weighted spacetime energies

$$\begin{aligned} {\mathbb {I}}_p \left[ \upalpha ^{[+2]}\right] \left( \tau _1,\tau _2\right)&= \int _{\tau _1}^{\tau _2} d\tau \int _{{\widetilde{\Sigma }}_{\tau }} dr d\sigma \ \frac{1}{r} e_p \left[ \upalpha ^{[+2]}\right] \ \ , \\ {\mathbb {I}}_p \left[ \uppsi ^{[+2]}\right] \left( \tau _1,\tau _2\right)&= \int _{\tau _1}^{\tau _2} d\tau \int _{{\widetilde{\Sigma }}_{\tau }} dr d\sigma \ \frac{1}{r} e_p \left[ \uppsi ^{[+2]}\right] \, . \end{aligned}$$

As with the fluxes, it will be useful to also define

74 $$\begin{aligned} {\mathbb {I}}_p^{\mathrm{left}} \left[ \upalpha ^{[+2]}\right] \left( \tau _1,\tau _2\right)&= \int _{\tau _1}^{\tau _2} d\tau \int _{{\widetilde{\Sigma }}_{\tau }^{\mathrm{left}}} dr\, d\sigma \ \frac{1}{r} e_p \left[ \upalpha ^{[+2]}\right] \, , \end{aligned}$$

75 $$\begin{aligned} {\mathbb {I}}_p^{\mathrm{left}} \left[ \uppsi ^{[+2]}\right] \left( \tau _1,\tau _2\right)&= \int _{\tau _1}^{\tau _2} d\tau \int _{{\widetilde{\Sigma }}_{\tau }^{\mathrm{left}} } dr\, d\sigma \ \frac{1}{r} e_p \left[ \uppsi ^{[+2]}\right] \, , \end{aligned}$$

76 $$\begin{aligned} {\mathbb {I}}_p^{\mathrm{right}} \left[ \upalpha ^{[+2]}\right] \left( \tau _1,\tau _2\right)&= \int _{\tau _1}^{\tau _2} d\tau \int _{{\widetilde{\Sigma }}_{\tau }^{\mathrm{right}} } dr\, d\sigma \ \frac{1}{r} e_p \left[ \upalpha ^{[+2]}\right] \, , \end{aligned}$$

77 $$\begin{aligned} {\mathbb {I}}_p^{\mathrm{right}} \left[ \uppsi ^{[+2]}\right] \left( \tau _1,\tau _2\right)&= \int _{\tau _1}^{\tau _2} d\tau \int _{{\widetilde{\Sigma }}_{\tau }^{\mathrm{right}} } dr\, d\sigma \ \frac{1}{r} e_p \left[ \uppsi ^{[+2]}\right] \, . \end{aligned}$$

Finally, we define

78 $$\begin{aligned} {\mathbb {I}}^{\mathrm{trap}}\left[ \upalpha ^{[+2]}\right] (\tau _1,\tau _2)= & {} \int _{\tau _1}^{\tau _2} d\tau \int _{\widetilde{\Sigma }_\tau ^{\mathrm{trap}}} dr\, d\sigma e_{0}\left[ \upalpha ^{[+2]}\right] \, , \end{aligned}$$

79 $$\begin{aligned} {\mathbb {I}}^{\mathrm{trap}}\left[ \uppsi ^{[+2]}\right] (\tau _1,\tau _2)= & {} \int _{\tau _1}^{\tau _2} d\tau \int _{\widetilde{\Sigma }_\tau ^{\mathrm{trap}}} dr\, d\sigma \, e_{0}\left[ \uppsi ^{[+2]}\right] . \end{aligned}$$

We note the relations

80 $$\begin{aligned}&{\mathbb {I}}_p \left[ \upalpha ^{[+2]}\right] \left( \tau _1,\tau _2\right) \lesssim {\mathbb {I}}_p^{\mathrm{left}} \left[ \upalpha ^{[+2]}\right] \left( \tau _1,\tau _2\right) + {\mathbb {I}}^{\mathrm{trap}}\left[ \upalpha ^{[+2]}\right] (\tau _1,\tau _2)\nonumber \\&\quad +\,{\mathbb {I}}_p^{\mathrm{right}} \left[ \upalpha ^{[+2]}\right] \left( \tau _1,\tau _2\right) . \end{aligned}$$

#### Weighted Energies for $\upalpha ^{[-2]}, \uppsi ^{[-2]}$

The quantities in this section will in general be applied to $\upalpha ^{[-2]}$, $\uppsi ^{[-2]}$ arising from a solution ${\tilde{\upalpha }}^{[-2]}$ of the inhomogeneous equation (53).

We define the following weighted energies on the slices ${\widetilde{\Sigma }}_{\tau }$:^9^

81 $$\begin{aligned} {\mathbb {E}}_{{\widetilde{\Sigma }}_{\tau }} \left[ \upalpha ^{[-2]}\right] \left( \tau \right)&= \int _{{\widetilde{\Sigma }}_{\tau }} dr d\sigma \sum _{\Gamma \in \{id, T, \Phi \}} \Big |\Gamma \left( \frac{\sqrt{r^2+a^2} \alpha ^{[-2]}}{\Delta ^2}\right) \Big |^2 r^{-1-\eta } \,, \end{aligned}$$

82 $$\begin{aligned} {\mathbb {E}}_{{\widetilde{\Sigma }}_{\tau }} \left[ \uppsi ^{[-2]}\right] \left( \tau \right)&= \int _{{\widetilde{\Sigma }}_{\tau }} dr d\sigma \sum _{\Gamma \in \{id, T, \Phi \}} \Big | \Gamma \left( \frac{{\uppsi }^{[-2]}(r^2+a^2)}{\sqrt{\Delta }} \right) \Big |^2 r^{-1-\eta }\, . \end{aligned}$$

We also define the energies

$$\begin{aligned} \overline{{\mathbb {E}}}_{{\widetilde{\Sigma }}_{\tau }} \left[ \upalpha ^{[-2]}\right] \left( \tau \right) \ \ , \ \ \overline{{\mathbb {E}}}_{{\widetilde{\Sigma }}_{\tau }} \left[ \uppsi ^{[-2]}\right] \left( \tau \right) \ \ \end{aligned}$$

by adding to the set $\Gamma$ in the energies without the overbar the vectorfield $\frac{r^2+a^2}{\Delta }{\underline{L}}$. Hence an overbar again indicates that the energy has been improved near the horizon.

##### Remark 4.2

In analogy with Remark 4.1, note that in view of the relations (116) and (117) controlling the energies above and in addition the energy (61) allows one to control also the *L* derivative of $\upalpha ^{[-2]}$ and $\uppsi ^{[-2]}$. Together these allow one to control the ${\underline{L}}$ derivative of $\upalpha ^{[-2]}$ and $\uppsi ^{[-2]}$ (without the $\Delta ^{-1}$-weight near the horizon) in view of the relation ${\underline{L}} = -{L} + 2T + \frac{2a}{r^2+a^2}\Phi$.

We define

$$\begin{aligned} {\mathbb {E}}^{\mathrm{left}}_{{\widetilde{\Sigma }}_{\tau }} \left[ \upalpha ^{[-2]}\right] , \qquad {\mathbb {E}}^{\mathrm{left}}_{{\widetilde{\Sigma }}_{\tau }} \left[ \uppsi ^{[+2]}\right] , \qquad {\mathbb {E}}^{\mathrm{right}}_{{\widetilde{\Sigma }}_{\tau }} \left[ \upalpha ^{[-2]}\right] , \qquad {\mathbb {E}}^{\mathrm{right}}_{{\widetilde{\Sigma }}_{\tau }} \left[ \uppsi ^{[-2]]}\right] , \end{aligned}$$

by appropriately restricting the domain in (81)–(82), in analogy with the definitions (70)–(71).

On (timelike) hypersurfaces of constant $r=A>r_+$ we define

83 $$\begin{aligned} {\mathbb {E}}_{r=A} \left[ \upalpha ^{[-2]}\right] \left( \tau _1,\tau _2\right)&= \int _{\tau _1}^{\tau _2} d\tau d\sigma \sum _{\Gamma \in \{id, T, \Phi \}} \Big | \Gamma \left( \frac{\sqrt{r^2+a^2} \alpha ^{[-2]}}{\Delta ^2}\right) \Big |^2 \Bigg |_{r=A} \, , \nonumber \\ {\mathbb {E}}_{r=A} \left[ \uppsi ^{[-2]}\right] \left( \tau _1,\tau _2\right)&=\int _{\tau _1}^{\tau _2} d\tau d\sigma \sum _{\Gamma \in \{id, T, \Phi \}} \Big | \Gamma \left( \frac{{\uppsi }^{[-2]}(r^2+a^2)}{\sqrt{\Delta }} \right) \Big |^2 \Bigg |_{r=A} \, . \end{aligned}$$

In the limit $r\rightarrow \infty$ we define on null infinity ${\mathcal {I}}^+$

84 $$\begin{aligned} {\mathbb {E}}_{{\mathcal {I}}^+} \left[ \upalpha ^{[-2]}\right] \left( \tau _1,\tau _2\right)&= \int _{\tau _1}^{\tau _2} d\tau d\sigma \sum _{\Gamma \in \{id, T, \Phi \}} \Big | \Gamma \left( \frac{\sqrt{r^2+a^2} \alpha ^{[-2]}}{\Delta ^2}\right) \Big |^2 \Bigg |_{r\rightarrow \infty }\, , \nonumber \\ {\mathbb {E}}_{{\mathcal {I}}^+} \left[ \uppsi ^{[-2]}\right] \left( \tau _1,\tau _2\right)&=\int _{\tau _1}^{\tau _2} d\tau d\sigma \sum _{\Gamma \in \{id, T, \Phi \}} \Big | \Gamma \left( \frac{{\uppsi }^{[-2]}(r^2+a^2)}{\sqrt{\Delta }} \right) \Big |^2 \Bigg |_{r\rightarrow \infty } \, . \end{aligned}$$

We also define the following weighted spacetime energies

85 $$\begin{aligned} {\mathbb {I}} \left[ \upalpha ^{[-2]}\right] \left( \tau _1,\tau _2\right)&= \int _{\tau _1}^{\tau _2} d\tau \int _{{\widetilde{\Sigma }}_{\tau }} dr d\sigma \sum _{\Gamma \in \{id, T, \Phi \}} \Big |\Gamma \left( \frac{\sqrt{r^2+a^2} \alpha ^{[-2]}}{\Delta ^2} \right) \Big |^2 r^{-1-\eta } \, , \end{aligned}$$

86 $$\begin{aligned} {\mathbb {I}} \left[ \uppsi ^{[-2]}\right] \left( \tau _1,\tau _2\right)&= \int _{\tau _1}^{\tau _2} d\tau \int _{{\widetilde{\Sigma }}_{\tau }} dr d\sigma \sum _{\Gamma \in \{id, T, \Phi \}} \Big | \Gamma \left( \frac{{\uppsi }^{[-2]}(r^2+a^2)}{\sqrt{\Delta }}\right) \Big |^2 r^{-1-\eta } \, , \end{aligned}$$

and the energies

$$\begin{aligned} \overline{{\mathbb {I}}} \left[ \upalpha ^{[-2]}\right] \left( \tau _1,\tau _2\right) \ \ , \ \ \overline{{\mathbb {I}}} \left[ \uppsi ^{[-2]}\right] \left( \tau _1,\tau _2\right) \end{aligned}$$

by adding to the set $\Gamma$ appearing in the definitions (85)–(86) the vectorfield $\frac{r^2+a^2}{\Delta }{\underline{L}}$. We define again

$$\begin{aligned} {\mathbb {I}}^{\mathrm{left}} \left[ \upalpha ^{[-2]}\right] , \qquad {\mathbb {I}}^{\mathrm{left}} \left[ \uppsi ^{[-2]}\right] , \qquad {\mathbb {I}}^{\mathrm{right}} \left[ \upalpha ^{[-2]}\right] , \qquad {\mathbb {I}}^{\mathrm{right}} \left[ \uppsi ^{[-2]}\right] , \end{aligned}$$

by restricting the domain in (85)–(86), in analogy with (74)–(77). Finally, in analogy with (78)–(79), we define

87 $$\begin{aligned} {\mathbb {I}}^{\mathrm{trap}}\left[ \upalpha ^{[-2]}\right] , \qquad {\mathbb {I}}^{\mathrm{trap}}\left[ \uppsi ^{[-2]}\right] \end{aligned}$$

and we note the $[-2]$ version of (80).

### Precise Statement of the Main Theorem: Theorem 4.1

We are now ready to give a precise version of the main theorem stated in Sect. 1.2:

#### Theorem 4.1

Let $({\tilde{\upalpha }}^{[\pm 2]}_0, {\tilde{\upalpha }}^{[\pm 2]}_1)\in {}^{[\pm 2]}H^j_{\mathrm{loc}}( \widetilde{\Sigma }_0)\times {}^{[\pm 2]}H^{j-1}_{\mathrm{loc}}(\widetilde{\Sigma }_0)$ and ${\tilde{\upalpha }}^{[\pm 2]}$ be as in the well-posedness Proposition 2.3.1, and let $\upalpha ^{[\pm 2]}$, $P^{[\pm 2]}$, $\Psi ^{[\pm 2]}$, $\uppsi ^{[\pm 2]}$ be as defined by (38), (46), (47), (48), (49) and (50). Then the following estimates hold:

1. degenerate energy boundedness and integrated local energy decay as in Theorem 9.1
2. red-shifted boundedness and integrated local energy decay as in Theorem 10.1
3. the weighted $r^p$ hierarchy of estimates as in Propositions 11.2.1 and  11.2.2 ($s=+2$)
  as well as Propositions 11.3.1 and 11.3.2 ($s=-2$)
4. polynomial decay of the energy as in Theorem 11.1.

For each statement, the Sobolev exponent *j* in the initial data norm is assumed large enough so that the quantities on the right hand sides of the corresponding estimates above are well defined.

Let us note that we can easily deduce from the above an alternative version where initial data is posed (and weighted norms given) on $\Sigma _0$ instead of $\widetilde{\Sigma }_0$. We suffice here with the remark that smooth, compactly supported initial data on $\Sigma _0$ trivially give rise to initial data on $\widetilde{\Sigma }_0$ satisfying the assumptions of the above theorem.

As an example of the pointwise estimates which follow immediately from the above theorem, let us note the following pointwise corollary (recall that $0<\eta <1$ was fixed in Sect. 2.1.2):

#### Corollary 4.1

Let $({\tilde{\upalpha }}^{[\pm 2]}_0, {\tilde{\upalpha }}^{[\pm 2]}_1)$ be smooth and of compact support. Then the solution $\tilde{\upalpha }$ satisfies

$$\begin{aligned} |r^{\frac{3+\eta }{2}} \tilde{\upalpha }^{[+2]}|\le C|{{\tilde{t}}^*}|^{-(2-\eta )/2} \ \ \ \ , \ \ \ \ |r^4\tilde{\upalpha }^{[-2]}|\le C|{{\tilde{t}}^*}|^{-(2-\eta )/2} \end{aligned}$$

where *C* depends on an appropriate higher Sobolev weighted norm.

The above decay rates can be improved following [87].

#### Remark 4.3

Recall that the quantities ${\tilde{\upalpha }}^{[\pm 2]}$ are regular on the horizon and that near infinity $r^{\frac{3+\eta }{2}} \tilde{\upalpha }^{[+2]} \sim r^{\frac{5+\eta }{2}} {\upalpha }^{[+2]} \sim r^{\frac{5+\eta }{2}} {\varvec{\Psi }}_0$ and $r^4 \tilde{\upalpha }^{[-2]} \sim r^{-3} \upalpha ^{[-2]} \sim r{\varvec{\Psi }}_4$, allowing direct comparison with the null-components of curvature in an orthonormal frame (see Sect. 2.4).

#### Remark 4.4

Note that, in view of Remark 4.3, one sees that the decay in *r* provided for ${\varvec{\Psi }}_0$ by Corollary 4.1 is weaker than peeling, consistent with the fact that, just as in [26], our weighted energies do not in fact impose initially the validity of peeling. This is important since it has been shown that peeling does not hold for generic physically interesting data [25].

### The Logic of the Proof

The remainder of the paper concerns the proof of Theorem 4.1.

Sections 5–8 are preliminary: Section 5 will prove an integrated energy estimate for $\Psi ^{[\pm 2]}$, $\uppsi ^{[\pm 2]}$ and $\upalpha ^{[\pm 2]}$ arising from general solutions to the inhomogeneous $s=\pm \,2$ Teukolsky equations (53) outside of the region $r\in [A_1,A_2]$, with additional boundary terms on $r=A_i$, as well as certain auxiliary estimates (Sect. 5.3) for $\Psi ^{[\pm 2]}$, $\uppsi ^{[\pm 2]}$ and $\upalpha ^{[\pm 2]}$ arising from a solution of the homogeneous equation (37). Sects. 6–8 will concern so-called $[A_1,A_2]$-admissible solutions and will provide frequency-localised estimates in the region $[A_1,A_2]$, again with boundary terms on $r=A_i$.

The proof proper of Theorem 4.1 commences in Sect. 9 where the degenerate integrated local energy decay and boundedness statements are proven (statement 1.), using the results of Sects. 5–9, applied to a particular solution  of the inhomogeneous equation (53) which arises by cutting off a solution $\upalpha$ of the homogeneous equation so that, *when restricted to the* *r*-range $[A_1,A_2]$,  is compactly supported in $t^*\in [0,\tau _{\mathrm{final}}]$. The estimate of statement 1. follows by appropriately summing the estimates of Sects. 5 and 8 applied to . We note already that when summing, the most dangerous boundary terms on $r=A_i$ have been arranged to precisely cancel, while the error term arising from the inhomogeneous term on the right hand side of the equation of  can easily be absorbed in view of its support properties and the auxiliary estimates of Sect. 5.3. Finally, in Sect. 9.6, we will distill from our argument a simpler, purely physical space proof of statement 1. for the axisymmetric case.

The degenerate boundedness and integrated local energy decay are combined with redshift estimates in Sect. 10 to obtain statement 2.

Finally, the weighted $r^p$ estimates are obtained in Sect. 11, giving statements 3.–4.

## Conditional Physical Space Estimates

In this section, we will derive certain physical space estimates for $\Psi ^{[\pm 2]}$, $\uppsi ^{[\pm 2]}$, $\upalpha ^{[\pm 2]}$ defined above, arising from solutions $\upalpha ^{[\pm 2]}$ of the inhomogeneous version (53) of the Teukolsky equation.

We first apply in Sect. 5.1 multiplier estimates for solutions $\Psi ^{[\pm 2]}$ of the *inhomogeneous* equation (54) outside the region $r\in [A_1,A_2]$. Here, we use the good divergence structure of the generalised Regge–Wheeler operator. We then estimate in Sect. 5.2 the quantities $\uppsi ^{[\pm 2]}$ and $\upalpha ^{[\pm 2]}$ via transport estimates. Taken together, these should be viewed as providing a conditional estimate stating that an integrated energy expression for $\Psi ^{[\pm 2]}$, $\uppsi ^{[\pm 2]}$ and $\upalpha ^{[\pm 2]}$ can be controlled from initial data provided that boundary terms on $r=A_i$ can be controlled. (To understand the latter boundary term, this estimate must be combined with that obtained in Sect. 8.)

Finally, we shall need some auxiliary physical space estimates (applied throughout ${\mathcal {R}}$) for $\Psi ^{[\pm 2]}$, $\uppsi ^{[\pm 2]}$ and $\upalpha ^{[\pm 2]}$ arising from a solution of the *homogeneous* Teukolsky equation (37). These will be given in Sect. 5.3.

***Let us note that we may always assume in what follows that any*** ${\widetilde{\upalpha }}^{[\pm 2]}$ ***referred to is in*** $\mathscr {S}^{[\pm 2]}_{\infty }(\widetilde{{\mathcal {R}}}_0)$.

### Multiplier Estimates for $\Psi ^{[\pm 2]}$

We will apply multiplier estimates for $\Psi ^{[\pm 2]}$. The main result is

#### Proposition 5.1.1

Let $\upalpha ^{[\pm 2]}$ be as in Proposition 3.2.1, and $\uppsi ^{[\pm 2]}$, $\Psi ^{[\pm 2]}$ be as defined in (46), (47), (49), (50). Let $\delta _1<1$, $\delta _2<1$ and $E>1$ be parameters and let $f_0$ be defined by (100) and $y_0$ be defined by (101). Then for sufficiently small $\delta _1$ and $\delta _2$ and sufficiently large *E*, it follows that for sufficiently small $|a|<a_0\ll M$, then for any $0\le \tau _1\le \tau _2$, we have

$$\begin{aligned}&{\mathbb {E}}^{\mathrm{away}}_{{\widetilde{\Sigma }}_{\tau }, \eta }\left[ \Psi ^{[\pm 2]}\right] (\tau _2) + {\mathbb {I}}^{\mathrm{away}}_{\eta }\left[ \Psi ^{[\pm 2]}\right] (\tau _1,\tau _2) \\&\quad \lesssim _{\delta _1,\delta _2, E} {\mathbb {E}}^{\mathrm{away}}_{{\widetilde{\Sigma }}_{\tau }, \eta }\left[ \Psi ^{[\pm 2]}\right] (\tau _1)\\&\qquad +{\mathfrak {H}}^{\mathrm{away}}[\Psi ^{[\pm 2]}](\tau _1,\tau _2) +{\mathbb {Q}}_{r=A_2}\left[ \Psi ^{[\pm 2]}\right] (\tau _1,\tau _2) - {\mathbb {Q}}_{r=A_1}\left[ \Psi ^{[\pm 2]}\right] (\tau _1,\tau _2)\\&\qquad +|a|\, {\mathbb {I}}^{\mathrm{left}}_{[\eta ]}\left[ \uppsi ^{[\pm 2]}\right] (\tau _1,\tau _2) +|a|\, {\mathbb {I}}^{\mathrm{right}}_{[\eta ]}\left[ \uppsi ^{[\pm 2]}\right] (\tau _1,\tau _2)\\&\qquad +|a|\, {\mathbb {I}}^{\mathrm{left}}_{[\eta ]}\left[ \upalpha ^{[\pm 2]}\right] (\tau _1,\tau _2) +|a|\, {\mathbb {I}}^{\mathrm{right}}_{[\eta ]}\left[ \upalpha ^{[\pm 2]}\right] (\tau _1,\tau _2). \end{aligned}$$

where ${\mathbb {Q}}_{r=A_i}[\Psi ^{[\pm 2]}](\tau _1,\tau _2)$ is defined by (103) and ${\mathfrak {H}}^{\mathrm{away}}[\Psi ^{[\pm 2]}](\tau _1,\tau _2)$ is defined by (104). Moreover the subindex $\left[ \eta \right]$ on the right hand side is equal to $\eta$ in case of $s=+2$ and it is dropped entirely in case $s=-2$.

We note already that the boundary terms ${\mathbb {Q}}_{r=A_2}\left[ \Psi ^{[+2]}\right] (\tau _1,\tau _2) - {\mathbb {Q}}_{r=A_1}\left[ \Psi ^{[+2]}\right] (\tau _1,\tau _2)$ appearing above formally coincide with those of the fixed frequency identity to be obtained in Sect. 8.2. Thus these terms will cancel when all identities are summed in Sect. 9.

In what follows, our multiplier constructions will be identical for $\Psi ^{[+2]}$ and $\Psi ^{[-2]}$. We will thus denote these simply as $\Psi$. The spin weight will be explicitly denoted however for the terms arising from the right hand side of (54).

#### Multiplier Identities

The proof of Proposition 5.1.1 will rely on various multiplier identities for (54). These are analogous for standard multiplier estimates proven for solutions of the scalar wave equation and in particular generalise specific estimates which have been proven for the Regge–Wheeler equation (60) on Schwarzschild in [31].

*The* $$T+\upomega _+ \chi \Phi $$ *identity.*

Multiplying (54) by $\left( T+\upomega _+ \chi \Phi \right) \overline{\Psi }$ (recall $\chi$ was fixed in Sect. 2.1.3) and taking the real part leads to (use the formulae of Appendix B.1 and B.3 and (289))

88 $$\begin{aligned}&\left( L+{\underline{L}}\right) \Big \{F^{T+\upomega _+\chi \Phi }_{L+{\underline{L}}}\Big \} +\left( L-{\underline{L}}\right) \Big \{F^{T+\upomega _+\chi \Phi }_{L-{\underline{L}}} \Big \} + I^{T+\upomega _+\chi \Phi } \nonumber \\&\quad \equiv {\mathrm{Re}}\left( \left( -\left( T+\upomega _+ \chi \Phi \right) \overline{\Psi }\right) \left( {\mathcal {J}}^{[s]} + {\mathfrak {G}}^{[s]}\right) \right) \end{aligned}$$

where $\equiv$ denotes equality after integration with respect to the measure $\sin \theta d\theta d\phi$ and89*The* *y* *identity.* Multiplying (54) by $y\left( L-{\underline{L}}\right) \overline{\Psi }$ for a smooth radial function *y* and taking the real part produces (use the formulae of Appendix B.4)

90 $$\begin{aligned}&\left( L+{\underline{L}}\right) \Big \{F^{y}_{L+{\underline{L}}}\Big \} +\left( L-{\underline{L}}\right) \Big \{F^{y}_{L-{\underline{L}}} \Big \} + I^y\nonumber \\&\quad \equiv \mathrm{Re}\left( \left( {\mathcal {J}}^{[s]} + {\mathfrak {G}}^{[s]}\right) \left( - y\left( L-{\underline{L}}\right) \overline{\Psi }\right) \right) \end{aligned}$$

where $\equiv$ denotes equality after integration with respect to the measure $\sin \theta d\theta d\phi$ and91*The h* *identity.* Multiplying (54) by $h\overline{\Psi }$ for a smooth radial function *h* and taking real parts leads to (use the formulae of Appendix B.2)

92 $$\begin{aligned} \left( L+{\underline{L}}\right) \Big \{F^{h}_{L+{\underline{L}}}\Big \} +\left( L-{\underline{L}}\right) \Big \{F^{h}_{L-{\underline{L}}} \Big \} + I^h \equiv \mathrm{Re} \left( -\left( {\mathcal {J}}^{[s]} + {\mathfrak {G}}^{[s]}\right) h \overline{\Psi }\right) \end{aligned}$$

where $\equiv$ denotes equality after integration with respect to the measure $\sin \theta d\theta d\phi$ and93*The f* *identity.* Adding the *y*-identity with $y=f$ and the *h*-identity with $h=f^\prime$ for *f* a smooth radial function yields the identity (recall (289))

94 $$\begin{aligned} \left( L+{\underline{L}}\right) \Big \{F^{f}_{L+{\underline{L}}}\Big \} +\left( L-{\underline{L}}\right) \Big \{F^{f}_{L-{\underline{L}}} \Big \} + I^{f} \equiv \mathrm{Re} \left( -\left( {\mathcal {J}}^{[s]} + {\mathfrak {G}}^{[s]}\right) \left( f^\prime \overline{\Psi } + 2f\overline{\Psi }^\prime \right) \right) \end{aligned}$$

where $\equiv$ denotes equality after integration with respect to the measure $\sin \theta d\theta d\phi$ and95*The* $r^p$-*weighted identity.* We multiply (54) by $r^p \beta _4 \xi L\overline{\Psi }$ with $\beta _4=1+4\frac{M}{r}$ and $\xi$ a smooth radial cut-off satisfying $\xi =0$ for $r \le R$ and $\xi =1$ for $r \ge R+M$ with *R* is chosen directly below (99) depending only on *M*. After taking the real parts of the resulting identity we obtain (use the formulae of Appendix B.6)

96 $$\begin{aligned} {\underline{L}} \big \{F^{r^p}_{{\underline{L}}}\big \} +L \big \{F^{r^p}_{L} \big \}+ I^{r^p} \equiv \mathrm{Re} \left( -\left( {\mathcal {J}}^{[s]} + {\mathfrak {G}}^{[s]}\right) r^p \beta _4 \xi L\overline{\Psi }\right) \end{aligned}$$

where $\equiv$ denotes equality after integration with respect to the measure $\sin \theta d\theta d\phi$ and97
98
99It is easy to see that we can choose *R* in the cut-off function such that the coefficients of $|{L}\Psi |^2$,  and $|\Psi |^2$ in (99) are all non-negative in $r\ge R+M$ for $p \in \left[ 0,2\right]$ and we henceforth make that choice.

##### Remark 5.1

(Conversion into divergence identities) To convert the identities derived in this section into proper spacetime divergence identities (from which the boundary contributions, etc., are most easily assessed) we recall the identities (26). Since the left hand side of any multiplier identity above has the schematic form

$$\begin{aligned} L \big \{ F_L\big \} + {\underline{L}} \big \{ F_{{\underline{L}}} \big \} + I + {\mathcal {E}} = RHS \, \end{aligned}$$

with $\int {\mathcal {E}} \sin \theta d\theta d\phi =0$, we can use (26) to convert them into the divergence form

$$\begin{aligned}&\nabla _a \left( L^a \frac{1}{\rho ^2} \frac{r^2+a^2}{\Delta } F_L + {\underline{L}}^a \frac{1}{\rho ^2} \frac{r^2+a^2}{\Delta } F_{{\underline{L}}} \right) + I \frac{1}{\rho ^2} \frac{r^2+a^2}{\Delta }+ {\mathcal {E}} \frac{1}{\rho ^2} \frac{r^2+a^2}{\Delta } \\&\quad = RHS \frac{1}{\rho ^2} \frac{r^2+a^2}{\Delta } \, . \end{aligned}$$

This is easily integrated using Stokes’ theorem and making use of the formulae (24) and (25) for the normals to the spacelike hypersurfaces (and the horizon and null infinity). Therefore it is the above identity which provides the precise sense in which the *F*’s in the identities indeed correspond to boundary terms. Note the term involving ${\mathcal {E}}$ disappears after integration with respect to the spacetime volume form (23).

#### Proof of Proposition 5.1.1

We define (cf. [31])

100 $$\begin{aligned} f_0 = \left( 1-\frac{3M}{r}\right) \left( 1+\frac{M}{r}\right) , \end{aligned}$$

and

101 $$\begin{aligned} y_0=\delta _1 ( (1-\chi )f_0(r)+\chi f_0^3(r))-\delta _1^2{\tilde{\chi }} r^{-\eta } \end{aligned}$$

where ${\tilde{\chi }}$ is a cutoff function such that ${\tilde{\chi }}=0$ for $r\le 9M$ and ${\tilde{\chi }}=1$ for $r\ge 10M$. We note the following Schwarzschild proposition

##### Proposition 5.1.2

[31] In the Schwarzschild case $a=0$, thenAs a consequence, for $\delta _1$ and $\delta _2$ sufficiently small and arbitrary *E* we haveNote that in view of Remark 5.1, upon application of the divergence theorem, the left hand side leads to a term which controls the integrand of ${\mathbb {I}}^{\mathrm{deg}}_\eta$.

Returning to the Kerr case, we add

1. the *f*-identity (94) applied with $f=f_0$,
2. the *y*-identity (90) applied with $y=y_0$,
3. *E* times the $T+\upomega _+\chi \Phi$ identity (88)
4. $\delta _2$ times the $r^{\eta }$ identity (96)

integrated in the region

$$\begin{aligned} \widetilde{{\mathcal {R}}}^{\mathrm{away}}(\tau _1,\tau _2)=\widetilde{{\mathcal {R}}}(\tau _1,\tau _2)\setminus \{A_1\le r\le A_2\} \end{aligned}$$

with respect to the spacetime volume form, and apply Remark 5.1. We always will assume $E>1$ and $\delta _1<1$, $\delta _2<1$.

We have:

1. Given any $E>1$, and sufficiently small $\delta _1$, $\delta _2$, then for $|a|<a_0\ll M$ sufficiently small, the resulting bulk term is nonnegative and in fact satisfies the coercitivity estimate
  102 $$\begin{aligned}&\int _{\widetilde{{\mathcal {R}}}^{\mathrm{away}}(\tau _1,\tau _2)} \left( I^{f} +I^{y}+ EI^{T+\upomega _+\chi \Phi }+\delta _2 I^{r^{\eta }}\right) \frac{1}{\rho ^2} \frac{r^2+a^2}{\Delta } dVol\nonumber \\&\quad \gtrsim _{\delta _1,\delta _2} {\mathbb {I}}^{\mathrm{away}}_{\eta } \left[ \Psi ^{[\pm 2]}\right] (\tau _1,\tau _2). \end{aligned}$$
  This follows from (a) Proposition 5.1.2, (b) smooth dependence on *a* to infer coercivity away from the horizon and away from infinity, (c) the fact that for all *a*, the term $I^{r^\eta }$ manifestly controls the integrand of ${\mathbb {I}}^{\mathrm{away}}_\eta$ for large *r*, (d) the fact that by direct inspection, for sufficiently small $|a|<a_0\ll M$, the term $I^f+I^y$ controls the integrand of ${\mathbb {I}}^{\mathrm{away}}_{\eta }$ near the horizon.
2. For sufficiently large $E>1$, then for all $\delta _1<1$, $\delta _2<1$ the total flux terms on ${\mathcal {H}}^+$ and ${\mathcal {I}}^+$ are nonnegative. This follows from Remark 5.1 and direct inspection of the boundary terms *F* thus generated, together with the relations concerning the volume form given in Sect. 2.1.2. (To avoid appealing to the fact that the flux to ${\mathcal {I}}^+$ is well defined, we may argue as follows: The identity can be applied in a region bounded by a finite ingoing null boundary, making the region of integration compact. The flux term on this boundary is manifestly nonnegative by the choice of the multipliers. One then takes this null boundary to the limit.)
3. Again, by Remark 5.1, inspection and the relations of Sect. 2.1.2, it follows that for sufficiently large $E>1$, then for all $\delta _1<1$, $\delta _2<1$, the arising flux term on ${\tilde{t}}^*=\tau _2$ controls the energy ${\mathbb {E}}^{\mathrm{away}}_{\widetilde{\Sigma }_\tau ,\eta }\left[ \Psi ^{[\pm 2]}\right] (\tau _2)$ with a uniform constant.
4. Similarly, for sufficiently large $E>1$, then for all $\delta _1<1$, $\delta _2<1$, the initial flux term on ${\tilde{t}}^*=\tau _1$ is controlled by the energy ${\mathbb {E}}^{\mathrm{away}}_{\widetilde{\Sigma }_\tau ,\eta }\left[ \Psi ^{[\pm 2]}\right] (\tau _1)$, with a constant depending on *E*.
5. The remaining flux terms on $r=A_1$ and $r=A_2$ produce exactly the expression
  $$\begin{aligned} {\mathbb {Q}}_{r=A_2}\left[ \Psi ^{[\pm 2]}\right] (\tau _1,\tau _2) - {\mathbb {Q}}_{r=A_1}\left[ \Psi ^{[\pm 2]}\right] (\tau _1,\tau _2) \end{aligned}$$
  where (recalling (89), (91) and (95))
  103 $$\begin{aligned}&{\mathbb {Q}}_{r=A_i}(\tau _1,\tau _2) = \int _{\tau _1}^{\tau _2} dt \int _0^\pi d\theta \sin \theta \int _0^{2\pi } \nonumber \\&\quad d\phi \Big \{ 2F^{f_0}_{L-{\underline{L}}} +2F^{y_0}_{L-{\underline{L}}} + 2EF^{T+\upomega _+\chi \Phi }_{L-{\underline{L}}} \Big \}. \end{aligned}$$
  This again follows from Remark 5.1: In $\left( t,r^*,\theta ,\phi \right)$-coordinates we have that $\frac{1}{\sqrt{g_{r^* r^*}}} \partial _{r^*}$ is the unit normal to constant $r^*$ hypersurfaces and $\rho ^2 \frac{1}{\sqrt{g_{r^* r^*}}} \frac{\Delta }{r^2+a^2} \sin \theta d\theta d\phi dt$ is the induced volume element. Using that $2\partial _{r^*} = L - {\underline{L}}$ and that $\partial _{r^*}$ is orthogonal to $L+{\underline{L}}$ the result follows. Observe that there is no contribution from $F^{r^\eta }$ in (103) because that multiplier is supported away from $A_2$.
6. The inhomogeneous term involving ${\mathfrak {G}}^{[\pm 2]}$ on the right hand side of (54) generates the term
  104 $$\begin{aligned} {\mathfrak {H}}^{\mathrm{away}}[\Psi ^{[\pm 2]}](\tau _1,\tau _2)= \int _{\widetilde{{\mathcal {R}}}^{\mathrm{away}}(\tau _1,\tau _2)} {\mathfrak {G}}^{[\pm 2]}\cdot (f,y,E, \delta _1,\delta _2) \, dVol \end{aligned}$$
  where (recall again Remark 5.1)
  $$\begin{aligned}&{\mathfrak {G}}^{[\pm 2]}\cdot (f,y,E, \delta _1,\delta _2) \doteq \frac{r^2+a^2}{\rho ^2\Delta } \Big \{ E\cdot \mathrm{Re}\left( \left( -\left( T+\omega _+ \chi \Phi \right) \overline{\Psi }\right) {\mathfrak {G}}^{[\pm 2]} \right) \\&\quad + \mathrm{Re} \left( - \left( f_0^\prime \overline{\Psi } + 2f_0\overline{\Psi }^\prime \right) {\mathfrak {G}}^{[\pm 2]} \right) \\&\quad +\delta _1\mathrm{Re}\left( \left( - 2f_0 \overline{\Psi }^\prime \right) {\mathfrak {G}}^{[\pm 2]} \right) +\delta _2\cdot \mathrm{Re} \left( - \left( r^\eta \beta _k \xi L\overline{\Psi }\right) {\mathfrak {G}}^{[\pm 2]}\right) \Big \} . \end{aligned}$$
7. By Cauchy–Schwarz, the term generated by the inhomogeneous term involving ${\mathcal {J}}^{[\pm 2]}$ on the right hand side of (54) can be bounded (with a constant depending on *E*) by the expression
  $$\begin{aligned}&|a|\, {\mathbb {I}}^{\mathrm{left}}_{[\eta ]}\left[ \uppsi ^{[\pm 2]}\right] (\tau _1,\tau _2) +|a|\, {\mathbb {I}}^{\mathrm{right}}_{[\eta ]}\left[ \uppsi ^{[\pm 2]}\right] (\tau _1,\tau _2)\\&\quad +|a|\, {\mathbb {I}}^{\mathrm{left}}_{[\eta ]}\left[ \upalpha ^{[\pm 2]}\right] (\tau _1,\tau _2) +|a|\, {\mathbb {I}}^{\mathrm{right}}_{[\eta ]}\left[ \upalpha ^{[\pm 2]}\right] (\tau _1,\tau _2) + |a| {\mathbb {I}}^{\mathrm{away}}_{\eta }\left[ \Psi ^{[\pm 2]}\right] (\tau _1,\tau _2) \, , \end{aligned}$$
  with the subindex $[\eta ]=\eta$ in case of $+2$, and $[\eta ]$ being dropped entirely in case of $s=-\,2$. Note that the last term can be absorbed in view of (102), for sufficiently small $|a|<a_0\ll M$.

Thus, for *E* sufficiently large, and $\delta _1$, $\delta _2$ sufficiently small, one obtains immediately the statement of Proposition 5.1.1.

**In what follows, we will now consider ***E* **as fixed in terms of** *M*, **and thus incorporate the ***E* **dependence into the** $\lesssim$, **etc.** We will further constrain $\delta _1$ and $\delta _2$ in Sect. 8.2 and thus we will continue to denote explicitly dependence of constants on $\delta _1$, $\delta _2$.

### Transport estimates for $\uppsi ^{[\pm 2]}$ and $\alpha ^{[\pm 2]}$

For transport estimates, it is natural to consider the spin $\pm \,2$ cases separately.

#### Transport Estimates for $\uppsi ^{[+2]}$ and $\alpha ^{[+2]}$

##### Proposition 5.2.1

Let $\upalpha ^{[+2]}$ be as in Proposition 3.2.1, and $\uppsi ^{[+2]}$, $\Psi ^{[+2]}$ be as defined in (46), (49). Then we have for any $p \in \{\eta ,1,2\}$ the following estimate in $\widetilde{{\mathcal {R}}}^{\mathrm{right}}(\tau _1,\tau _2)$:

105 $$\begin{aligned}&{\mathbb {E}}^{\mathrm{right}}_{{\widetilde{\Sigma }}_{\tau },p} \left[ \upalpha ^{[+2]}\right] (\tau _2) +{\mathbb {I}}_p^{\mathrm{right}} \left[ \upalpha ^{[+2]}\right] \left( \tau _1,\tau _2 \right) + {\mathbb {E}}_{r=A_2} \left[ \upalpha ^{[+2]}\right] \left( \tau _1,\tau _2\right) \nonumber \\&\qquad +{\mathbb {E}}^{\mathrm{right}}_{{\widetilde{\Sigma }}_{\tau },p} \left[ \uppsi ^{[+2]}\right] (\tau _2) +{\mathbb {I}}_p^{\mathrm{right}} \left[ \uppsi ^{[+2]}\right] \left( \tau _1,\tau _2 \right) +{\mathbb {E}}_{r=A_2} \left[ \uppsi ^{[+2]}\right] \left( \tau _1,\tau _2\right) \nonumber \\&\quad \lesssim {\mathbb {I}}^{\mathrm{away}}_{p}\left[ \Psi ^{[+2]}\right] (\tau _1, \tau _2) + {\mathbb {E}}^{\mathrm{right}}_{{\widetilde{\Sigma }}_{\tau },p} \left[ \upalpha ^{[+2]}\right] (\tau _1) +{\mathbb {E}}^{\mathrm{right}}_{{\widetilde{\Sigma }}_{\tau },p} \left[ \uppsi ^{[+2]}\right] (\tau _1) \end{aligned}$$

and the following estimate in $\widetilde{{\mathcal {R}}}^{\mathrm{left}}(\tau _1,\tau _2)$:

106 $$\begin{aligned}&{\mathbb {E}}^{\mathrm{left}}_{{\widetilde{\Sigma }}_{\tau },p} \left[ \upalpha ^{[+2]}\right] (\tau _2) +{\mathbb {I}}_p^{\mathrm{left}} \left[ \upalpha ^{[+2]}\right] \left( \tau _1,\tau _2 \right) + {\mathbb {E}}_{{\mathcal {H}}^+} \left[ \upalpha ^{[+2]}\right] \left( \tau _1,\tau _2\right) \nonumber \\&\qquad +{\mathbb {E}}^{\mathrm{left}}_{{\widetilde{\Sigma }}_{\tau },p} \left[ \uppsi ^{[+2]}\right] (\tau _2) +{\mathbb {I}}_p^{\mathrm{left}} \left[ \uppsi ^{[+2]}\right] \left( \tau _1,\tau _2 \right) +{\mathbb {E}}_{{\mathcal {H}}^+} \left[ \uppsi ^{[+2]}\right] \left( \tau _1,\tau _2\right) \nonumber \\&\quad \lesssim {\mathbb {I}}^{\mathrm{away}}_{ p}\left[ \Psi ^{[+2]}\right] (\tau _1, \tau _2) + {\mathbb {E}}^{\mathrm{left}}_{{\widetilde{\Sigma }}_{\tau },p} \left[ \upalpha ^{[+2]}\right] (\tau _1) +{\mathbb {E}}^{\mathrm{left}}_{{\widetilde{\Sigma }}_{\tau },p} \left[ \uppsi ^{[+2]}\right] (\tau _1) \nonumber \\&\qquad + {\mathbb {E}}_{r=A_1} \left[ \upalpha ^{[+2]}\right] \left( \tau _1,\tau _2\right) + {\mathbb {E}}_{r=A_1} \left[ \uppsi ^{[+2]}\right] \left( \tau _1,\tau _2\right) . \end{aligned}$$

##### Proof

We recall from (49) and (51) the relations

107 $$\begin{aligned} -2 \frac{\Delta }{(r^2+a^2)^2} \sqrt{\Delta } \uppsi ^{[+2]}&= {\underline{L}}^a \nabla _a \left( \Delta ^2 \left( r^2+a^2\right) ^{-\frac{3}{2}} \upalpha ^{[+2]}\right) \, , \end{aligned}$$

108 $$\begin{aligned} \frac{\Delta }{(r^2+a^2)^2} \Psi ^{[+2]}&= {\underline{L}}^a \nabla _a \left( \sqrt{\Delta } \uppsi ^{[+2]}\right) \, . \end{aligned}$$

From (107) we derive for $n\ge 0$

109 $$\begin{aligned}&\nabla _a \left( r^n \frac{1}{\rho ^2}\frac{r^2+a^2}{\Delta }{\underline{L}}^a \Big |\upalpha ^{[+2]} \Delta ^2 \left( r^2+a^2\right) ^{-\frac{3}{2}}\Big |^2\right) +n \frac{ r^{n-1}}{\rho ^2} \Big |\upalpha ^{[+2]} \Delta ^2 \left( r^2+a^2\right) ^{-\frac{3}{2}}\Big |^2 \nonumber \\&\quad = -2 \frac{\left( r^2+a^2\right) ^2}{\Delta \rho ^2} w^\frac{3}{2} r^n\nonumber \\&\qquad \times \left( \uppsi ^{[+2]} \cdot \overline{ \upalpha ^{[+2]} \Delta ^2 \left( r^2+a^2\right) ^{-\frac{3}{2}}} + \overline{\uppsi ^{[+2]}} \cdot \upalpha ^{[+2]} \Delta ^2 \left( r^2+a^2\right) ^{-\frac{3}{2}}\right) \, , \end{aligned}$$

and hence

110 $$\begin{aligned}&\nabla _a \left( r^n \frac{1}{\rho ^2}\frac{r^2+a^2}{\Delta }{\underline{L}}^a \Big | \frac{\upalpha ^{[+2]} \Delta ^2}{ \left( r^2+a^2\right) ^{\frac{3}{2}}}\Big |^2\right) +\frac{n}{2} \frac{ r^{n-1}}{\rho ^2} \Big | \frac{\upalpha ^{[+2]} \Delta ^2}{ \left( r^2+a^2\right) ^{\frac{3}{2}}}\Big |^2\nonumber \\&\quad \le C \frac{1}{\rho ^2} \frac{r^{n+1}}{(r^2+a^2)^2} | \sqrt{\Delta }\uppsi ^{[+2]}|^2 \, . \end{aligned}$$

Moreover, the same estimate (110) holds replacing $\upalpha ^{[+2]}$ by $T \upalpha ^{[+2]}$ ($\Phi \upalpha ^{[+2]}$) on the left and $\uppsi ^{[+2]}$ by $T \uppsi ^{[+2]}$ ($\Phi \uppsi ^{[+2]}$) on the right since the relation (107) trivially commutes with the Killing fields *T* and $\Phi$ respectively. We will refer to those estimates as the “*T*-commuted and $\Phi$-commuted (110)” below.

Similarly from (108),

111 $$\begin{aligned}&\nabla _a \left( r^n \frac{1}{\rho ^2}\frac{r^2+a^2}{\Delta } {\underline{L}}^a |\uppsi ^{[+2]} \sqrt{\Delta }|^2\right) +\frac{n}{2} \frac{ r^{n-1}}{\rho ^2} |\uppsi ^{[+2]} \sqrt{\Delta }|^2\nonumber \\&\quad \le C_n \frac{1}{\rho ^2} \frac{r^{n+1}}{\left( r^2+a^2\right) ^2} |\Psi ^{[+2]}|^2 \, \end{aligned}$$

and the same estimate replacing $\uppsi ^{[+2]}$ by $T \uppsi ^{[+2]}$ ($\Phi \uppsi ^{[+2]}$) on the left and $\Psi ^{[+2]}$ by $T\Psi ^{[+2]}$ ($\Phi \Psi ^{[+2]}$) on the right. We again refer to the latter as the “*T*-commuted and $\Phi$-commuted (111)” below.

Let us first obtain the estimate in $\widetilde{{\mathcal {R}}}^{\mathrm{right}}(\tau _1,\tau _2)$. The case in $\widetilde{{\mathcal {R}}}^{\mathrm{left}}(\tau _1,\tau _2)$ is analogous but easier since weights in *r* do not play a role. We add

- (111) with $$n \in \{\eta ,1,2-\eta \}$$
- the $\Phi$-commuted (111) with $n \in \{\eta ,1,2-\eta \}$
- the *T*-commuted (111) with $n =2-\eta$

integrated over $\widetilde{{\mathcal {R}}}^{\mathrm{right}}(\tau _1,\tau _2)$. Combining the above we conclude for $p \in \{\eta ,1,2\}$ the estimate

112 $$\begin{aligned}&{\mathbb {E}}^{\mathrm{right}}_{{\widetilde{\Sigma }}_{\tau },p} \left[ \uppsi ^{[+2]}\right] \left( \tau _2\right) +{\mathbb {E}}_{r=A_2} \left[ \uppsi ^{[+2]}\right] \left( \tau _1,\tau _2\right) + {\mathbb {I}}^{\mathrm{right}}_p \left[ \uppsi ^{[+2]}\right] \left( \tau _1,\tau _2\right) \nonumber \\&\quad \lesssim {\mathbb {I}}^{\mathrm{away}}_p \left[ \Psi ^{[+2]}\right] \left( \tau _1,\tau _2\right) + {\mathbb {E}}^{\mathrm{right}}_{{\widetilde{\Sigma }}_{\tau },p} \left[ \uppsi ^{[+2]}\right] \left( \tau _1\right) \, . \end{aligned}$$

Turning to the estimate (110) we add

- (110) with $$n \in \{2+\eta ,3,4-\eta \}$$
- the $\Phi$-commuted (110) with $n \in \{2+\eta ,3,4-\eta \}$
- the *T*-commuted (110) with $n =4-\eta$

integrated over $\widetilde{{\mathcal {R}}}(\tau _1,\tau _2) \cap \{r \ge A_2\}$. Combining the above we conclude for $p \in \{\eta ,1,2\}$ (note that for $p=2$ there is an $\eta$-loss in the definition of the densities (67), (68), ensuring that we can indeed set $p=2$)

113 $$\begin{aligned}&{\mathbb {E}}^{\mathrm{right}}_{{\widetilde{\Sigma }}_{\tau },p} \left[ \upalpha ^{[+2]}\right] \left( \tau _2\right) + {\mathbb {E}}_{r=A_2} \left[ \upalpha ^{[+2]}\right] \left( \tau _1,\tau _2\right) + {\mathbb {I}}^{\mathrm{right}}_p \left[ \upalpha ^{[+2]}\right] \left( \tau _1,\tau _2\right) \nonumber \\&\quad \lesssim {\mathbb {I}}^{\mathrm{right}}_p \left[ \uppsi ^{[+2]}\right] \left( \tau _1,\tau _2\right) + {\mathbb {E}}^{\mathrm{right}}_{{\widetilde{\Sigma }}_{\tau },p} \left[ \upalpha ^{[+2]}\right] \left( \tau _1\right) \, . \end{aligned}$$

Combining (113) and (112) yields the desired estimate to the right of trapping.

As remarked above, the estimate in the “left region” $\widetilde{{\mathcal {R}}}^{\mathrm{left}}(\tau _1,\tau _2)$ is easier and left to the reader. $\square$

#### Transport Estimates for $\uppsi ^{[-2]}$ and $\upalpha ^{[-2]}$

##### Proposition 5.2.2

Let $\upalpha ^{[-2]}$ be as in Proposition 3.2.1, and $\uppsi ^{[-2]}$, $\Psi ^{[-2]}$ be as defined in (47), (50). Then we have the following estimate in $\widetilde{{\mathcal {R}}}^{\mathrm{right}}(\tau _1,\tau _2)$:

114 $$\begin{aligned}&{\mathbb {E}}^{\mathrm{right}}_{{\widetilde{\Sigma }}_{\tau }} \left[ \upalpha ^{[-2]}\right] (\tau _2) +{\mathbb {I}}^{\mathrm{right}} \left[ \upalpha ^{[-2]}\right] \left( \tau _1,\tau _2 \right) + {\mathbb {E}}_{{\mathcal {I}}^+} \left[ \upalpha ^{[-2]}\right] \left( \tau _1,\tau _2\right) \nonumber \\&\qquad +{\mathbb {E}}^{\mathrm{right}}_{{\widetilde{\Sigma }}_{\tau }} \left[ \uppsi ^{[-2]}\right] (\tau _2) +{\mathbb {I}}^{\mathrm{right}} \left[ \uppsi ^{[-2]}\right] \left( \tau _1,\tau _2 \right) + {\mathbb {E}}_{{\mathcal {I}}^+} \left[ \uppsi ^{[-2]}\right] \left( \tau _1,\tau _2\right) \nonumber \\&\quad \lesssim {\mathbb {I}}^{\mathrm{away}}_{ \eta }\left[ \Psi ^{[-2]}\right] (\tau _1, \tau _2) + {\mathbb {E}}^{\mathrm{right}}_{{\widetilde{\Sigma }}_{\tau }} \left[ \upalpha ^{[-2]}\right] (\tau _1) +{\mathbb {E}}^{\mathrm{right}}_{{\widetilde{\Sigma }}_{\tau }} \left[ \uppsi ^{[-2]}\right] (\tau _1) \nonumber \\&\qquad + {\mathbb {E}}_{r=A_2} \left[ \upalpha ^{[-2]}\right] \left( \tau _1,\tau _2\right) +{\mathbb {E}}_{r=A_2} \left[ \uppsi ^{[-2]}\right] \left( \tau _1,\tau _2\right) \end{aligned}$$

and the following estimate in $\widetilde{{\mathcal {R}}}^{\mathrm{left}}(\tau _1,\tau _2)$:

115 $$\begin{aligned}&{\mathbb {E}}^{\mathrm{left}}_{{\widetilde{\Sigma }}_{\tau }} \left[ \upalpha ^{[-2]}\right] (\tau _2) +{\mathbb {I}}^{\mathrm{left}} \left[ \upalpha ^{[-2]}\right] \left( \tau _1,\tau _2 \right) + {\mathbb {E}}_{r=A_1} \left[ \upalpha ^{[-2]}\right] \left( \tau _1,\tau _2\right) \nonumber \\&\qquad +{\mathbb {E}}^{\mathrm{left}}_{{\widetilde{\Sigma }}_{\tau }} \left[ \uppsi ^{[-2]}\right] (\tau _2) +{\mathbb {I}}^{\mathrm{left}} \left[ \uppsi ^{[-2]}\right] \left( \tau _1,\tau _2 \right) + {\mathbb {E}}_{r=A_1} \left[ \uppsi ^{[-2]}\right] \left( \tau _1,\tau _2\right) \nonumber \\&\quad \lesssim {\mathbb {I}}^{\mathrm{away}}_{ \eta }\left[ \Psi ^{[-2]}\right] (\tau _1, \tau _2) + {\mathbb {E}}^{\mathrm{left}}_{{\widetilde{\Sigma }}_{\tau }} \left[ \upalpha ^{[-2]}\right] (\tau _1) +{\mathbb {E}}^{\mathrm{left}}_{{\widetilde{\Sigma }}_{\tau }} \left[ \uppsi ^{[-2]}\right] (\tau _1). \end{aligned}$$

##### Remark 5.2

As the proof will show, these estimates also hold replacing ${\mathbb {I}}^{\mathrm{away}}_{ \eta }\left[ \Psi ^{[-2]}\right]$ by ${\mathbb {I}}^{\mathrm{away}}_{0}\left[ \Psi ^{[-2]}\right]$ provided we drop the two terms on null infinity ${\mathcal {I}}^+$ in (114) and weaken the *r*-weight in the energies ${\mathbb {E}}^{\mathrm{right}}_{{\widetilde{\Sigma }}_{\tau }} \left[ \upalpha ^{[-2]}\right]$ and ${\mathbb {E}}^{\mathrm{right}}_{{\widetilde{\Sigma }}_{\tau }} \left[ \uppsi ^{[-2]}\right]$ from $r^{-1-\eta }$ to $r^{-1-2\eta }$; see (81), (82). This way one could avoid the $r^\eta$ multiplier for $\Psi ^{[-2]}$ (at the cost of losing control over the generically non-vanishing fluxes on null infinity).

##### Proof

We recall the relations

116 $$\begin{aligned} 2 \frac{\Delta }{(r^2+a^2)^2} \sqrt{\Delta }{\uppsi }^{[-2]}&= L^a \nabla _a \left( \upalpha ^{[-2]} \left( r^2+a^2\right) ^{-\frac{3}{2}}\right) \, ,\end{aligned}$$

117 $$\begin{aligned} -\frac{\Delta }{(r^2+a^2)^2}\Psi ^{[-2]}&=L^a \nabla _a \left( \sqrt{\Delta } {\uppsi }^{[-2]}\right) \, . \end{aligned}$$

From (116) we derive (recall $\rho ^2=r^2+a^2\cos ^2\theta$) for any $n, \eta \in {\mathbb {R}}$

$$\begin{aligned}&\nabla _a \left( \left( \frac{\Delta }{r^2+a^2}\right) ^{-n-1+4} \left( 1+\frac{1}{r^\eta }\right) \frac{1}{\rho ^2} L^a \Big |\frac{\sqrt{r^2+a^2} \alpha ^{[-2]}}{\Delta ^2} \Big |^2\right) \\&\qquad +\left[ \left( 1+\frac{1}{r^\eta }\right) \frac{2Mn \left( r^2-a^2\right) }{ (r^2+a^2)^2} + \frac{\eta }{r^{1+\eta }} \frac{\Delta }{r^2+a^2} \right] \\&\qquad \times \frac{1}{\rho ^2} \left( \frac{\Delta }{r^2+a^2}\right) ^{-n-1+4}\Big |\frac{\sqrt{r^2+a^2} \alpha ^{[-2]}}{\Delta ^2} \Big |^2 \\&\quad = -2 \left( r^2+a^2\right) w^\frac{3}{2} \left( \frac{\Delta }{r^2+a^2}\right) ^{-n-1+2} \frac{1}{\rho ^2} \left( 1+\frac{1}{r^\eta }\right) \\&\qquad \left( \uppsi ^{[-2]} \cdot \frac{\sqrt{r^2+a^2} \overline{\alpha ^{[-2]}}}{\Delta ^2} + \overline{\uppsi ^{[-2]}} \cdot \frac{\sqrt{r^2+a^2} {\alpha ^{[-2]}}}{\Delta ^2}\right) \, , \end{aligned}$$

and hence, choosing $n=3$, we have for any $\eta >0$ the estimate

118 $$\begin{aligned}&\nabla _a \left( \left( 1+\frac{1}{r^\eta }\right) \frac{1}{\rho ^2} L^a \Big |\frac{\sqrt{r^2+a^2} \alpha ^{[-2]}}{\Delta ^2} \Big |^2\right) \nonumber \\&\qquad +\frac{1}{2} \left[ \left( 1+\frac{1}{r^\eta }\right) \frac{6M \left( r^2-a^2\right) }{ (r^2+a^2)^2} + \frac{\eta }{r^{1+\eta }} \frac{\Delta }{r^2+a^2} \right] \frac{1}{\rho ^2}\Big |\frac{\sqrt{r^2+a^2} \alpha ^{[-2]}}{\Delta ^2} \Big |^2 \nonumber \\&\quad \le C_\eta \frac{1}{\rho ^2} \Big |\frac{ \left( r^2+a^2\right) {\uppsi ^{[-2]}}}{\sqrt{\Delta }}\Big |^2 \frac{r^{1+\eta }}{\left( r^2+a^2\right) ^2} \, . \end{aligned}$$

Moreover, the same estimate holds replacing $1+ \frac{1}{r^\eta }$ by $\frac{1}{r^\eta }$ on the left and $r^{1+\eta }$ by $r^{1-\eta }$ on the right (cf. Remark 5.2). Note that the estimate (118) also holds replacing $\upalpha ^{[-2]}$ by $T\upalpha ^{[-2]}$ ($\Phi \upalpha ^{[-2]}$) and $\uppsi ^{[-2]}$ by $T\uppsi ^{[-2]}$ ($\Phi \uppsi ^{[-2]}$) in view of the relation (116) commuting trivially with the Killing field *T* and $\Phi$. We will refer to those estimates as the *T*- and $\Phi$-commuted (118) below.

From (117) we derive

119 $$\begin{aligned}&\nabla _a \left( \left( 1+\frac{1}{r^\eta }\right) \frac{1}{\rho ^2} L^a \Bigg |\frac{{\uppsi }^{[-2]}(r^2+a^2)}{\sqrt{\Delta }}\Bigg |^2\right) \nonumber \\&\qquad +\frac{1}{2} \left[ \left( 1+\frac{1}{r^\eta }\right) \frac{2M \left( r^2-a^2\right) }{ (r^2+a^2)^2} + \frac{\eta }{r^{1+\eta }} \frac{\Delta }{r^2+a^2} \right] \frac{1}{\rho ^2} \Bigg |\frac{{\uppsi ^{[-2]}}(r^2+a^2)}{\sqrt{\Delta }}\Bigg |^2 \nonumber \\&\quad \le C_\eta \frac{1}{\rho ^2} \Big | \Psi ^{[-2]}\Big |^2\frac{r^{1+\eta }}{\left( r^2+a^2\right) ^2} \, . \end{aligned}$$

Moreover, the same estimate holds replacing $1+ \frac{1}{r^\eta }$ by $\frac{1}{r^\eta }$ on the left and $r^{1+\eta }$ by $r^{1-\eta }$ on the right (cf. Remark 5.2). Note that the estimate (119) also holds replacing $\uppsi ^{[-2]}$ by $T\uppsi ^{[-2]}$ ($\Phi \uppsi ^{[-2]}$) and $\Psi ^{[-2]}$ by $T\Psi ^{[-2]}$ ($\Phi \Psi ^{[-2]}$) in view of the relation (117) commuting trivially with the Killing field *T* and $\Phi$. We will refer to this estimates as the *T*- and $\Phi$-commuted (119) below.

We are now ready to prove the estimate in $\widetilde{{\mathcal {R}}}^{\mathrm{left}}(\tau _1,\tau _2)$.

Integrating (119) and the *T*-commuted and $\Phi$-commuted (119) over $\widetilde{{\mathcal {R}}}^{\mathrm{left}}(\tau _1,\tau _2)$ produces

120 $$\begin{aligned}&{\mathbb {E}}^{\mathrm{left}}_{{\widetilde{\Sigma }}_{\tau }} \left[ \uppsi ^{[-2]}\right] \left( \tau _2\right) + {\mathbb {I}}^{\mathrm{left}} \left[ \uppsi ^{[-2]}\right] \left( \tau _1,\tau _2\right) + {\mathbb {E}}_{r=A_1} \left[ \uppsi ^{[-2]}\right] \left( \tau _1,\tau _2\right) \nonumber \\&\quad \lesssim {\mathbb {I}}_{\eta }^{\mathrm{away}} \left[ \Psi ^{[-2]}\right] \left( \tau _1,\tau _2\right) + {\mathbb {E}}^{\mathrm{left}}_{{\widetilde{\Sigma }}_{\tau }} \left[ \uppsi ^{[-2]}\right] \left( \tau _1\right) . \end{aligned}$$

Integrating (118) and the *T*-commuted and $\Phi$-commuted (118) over $\widetilde{{\mathcal {R}}}^{\mathrm{left}}(\tau _1,\tau _2)$ produces

121 $$\begin{aligned}&{\mathbb {E}}^{\mathrm{left}}_{{\widetilde{\Sigma }}_{\tau }} \left[ \upalpha ^{[-2]}\right] \left( \tau _2\right) + {\mathbb {I}}^{\mathrm{left}} \left[ \upalpha ^{[-2]}\right] \left( \tau _1,\tau _2\right) + {\mathbb {E}}_{r=A_1} \left[ \upalpha ^{[-2]}\right] \left( \tau _1,\tau _2\right) \nonumber \\&\quad \lesssim {\mathbb {I}}^{\mathrm{left}} \left[ \uppsi ^{[-2]}\right] \left( \tau _1,\tau _2\right) + {\mathbb {E}}^{\mathrm{left}}_{{\widetilde{\Sigma }}_{\tau }} \left[ \upalpha ^{[-2]}\right] \left( \tau _1\right) . \end{aligned}$$

Combining the last two estimates produces the desired estimate in $\widetilde{{\mathcal {R}}}^{\mathrm{left}}(\tau _1,\tau _2)$. The estimate in $\widetilde{{\mathcal {R}}}^{\mathrm{right}}(\tau _1,\tau _2)$ is proven entirely analogously and is again left to the reader. The only important observation is that the good $\uppsi$-spacetime term generated from (119) is stronger (in terms of *r*-weight) than what is needed on the left hand side of (118). $\square$

### Auxiliary Estimates

We collect a number of auxiliary estimates we shall require.

#### The Homogeneous $T+\upomega _+\chi \Phi$ Estimate

##### Proposition 5.3.1

Let $\upalpha ^{[\pm 2]}$ satisfy the homogeneous Teukolsky equation (37) and let $\uppsi ^{[\pm 2]}$, $\Psi ^{[\pm 2]}$ be as defined in (46), (47), (49), (50). Then we have for any $0\le \tau _1\le \tau _2$

122 $$\begin{aligned}&{\mathbb {E}}_{\widetilde{\Sigma }_\tau ,0}\left[ \Psi ^{[\pm 2]}\right] (\tau _2) \lesssim |a| {\mathbb {I}}^{\mathrm{deg}}_0\left[ \Psi ^{[\pm 2]}\right] (\tau _1,\tau _2) +|a| {\mathbb {I}}_{[\eta ]}\left[ \uppsi ^{[\pm 2]}\right] (\tau _1,\tau _2)\nonumber \\&\quad +|a| {\mathbb {I}}_{[\eta ]}\left[ \upalpha ^{[\pm 2]}\right] (\tau _1,\tau _2) +{\mathbb {E}}_{\widetilde{\Sigma }_\tau ,0}\left[ \Psi ^{[\pm 2]}\right] (\tau _1). \end{aligned}$$

Here the subindex $\left[ \eta \right]$ is equal to $\eta$ in case of $s=+2$ and it is dropped entirely in case $s=-2$.

##### Proof

The inequality (122) follows from integrating the identity (88) associated with the multiplier $T+\upomega _+\chi \Phi$ over the region $\widetilde{{\mathcal {R}}} \left( \tau _1,\tau _2\right)$ using Remark 5.1. The details are as follows. Note that ${\mathfrak {G}}^{[s]}=0$ and that for the boundary terms one has

$$\begin{aligned}&{\mathbb {E}}_{\widetilde{\Sigma }_\tau ,0}\left[ \Psi ^{[\pm 2]}\right] \left( \tau _2\right) \lesssim {\mathbb {E}}_{\widetilde{\Sigma }_\tau ,0}\left[ \Psi ^{[\pm 2]}\right] \left( \tau _1\right) \\&\quad + \int _{\widetilde{{\mathcal {R}}} \left( \tau _1,\tau _2\right) } \Big ( \left( L+{\underline{L}}\right) F_{L+{\underline{L}}}^{T+ \omega _+ \chi \Phi } + \left( L-{\underline{L}}\right) F_{L-{\underline{L}}}^{T+ \omega _+ \chi \Phi } \Big ) \frac{1}{\rho ^2} \frac{r^2+a^2}{\Delta } dVol \end{aligned}$$

while for the spacetime term clearly

123 $$\begin{aligned} \int _{\widetilde{{\mathcal {R}}} \left( \tau _1,\tau _2\right) } -I^{T+\omega _+ \chi \Phi } \frac{1}{\rho ^2} \frac{r^2+a^2}{\Delta } dVol \lesssim |a| {\mathbb {I}}^{\mathrm{deg}}_0\left[ \Psi ^{[\pm 2]}\right] (\tau _1,\tau _2) \, . \end{aligned}$$

It remains to estimate the term

124 $$\begin{aligned} \int _{\widetilde{{\mathcal {R}}} \left( \tau _1,\tau _2\right) } \text {Re} \left[ -\left( T + \omega _+ \chi \Phi \right) \overline{\Psi ^{[\pm 2]}} {\mathcal {J}}^{[\pm 2]}\right] \, . \end{aligned}$$

In view of the fact that the support of $\chi$ is away from the degeneration of ${\mathbb {I}}^{\mathrm{deg}}$ we can easily control the $\omega _+\chi \Phi$-part by the right hand side of (122) using the Cauchy–Schwarz inequality. For the remaining term $\text {Re} \left[ T \overline{\Psi ^{[\pm 2]}} {\mathcal {J}}^{[\pm 2]}\right]$ we restrict the proof to the $s=+2$ case, the $s=-2$ case being completely analogous. We recall from Proposition 3.2.1 that

$$\begin{aligned} {\mathcal {J}}^{[+2]}&=awc_1(r)\Phi \left( \sqrt{\Delta }\psi ^{[+2]}\right) +a^2wc_2(r)\left( \sqrt{\Delta }\psi ^{[+2]}\right) \\&\quad + a^3wc_3(r)\Phi \left( \Delta ^2(r^2+a^2)^{-3/2}\alpha ^{[+2]}\right) +a^2w c_4(r)\left( \Delta ^2(r^2+a^2)^{-3/2}\alpha ^{[+2]}\right) , \end{aligned}$$

where $|c_1(r)|\lesssim 1$, $|c_2(r)|\lesssim r^{-1}$, $|c_3(r)|\lesssim r^{-1}$ and $|c_4(r)|\lesssim 1$. Note that unless we are in the region near trapping all of these terms feeding into (124) are easily controlled by the right hand side of (122) using the Cauchy-Schwarz inequality. We can also assume without loss of generality $\tau _2 > \tau _1+2$ in (124) as otherwise we can again apply Cauchy–Schwarz and estimate the spacetime integral of $T\Psi ^{[+2]}$ by the supremum of the energy through each slice $\widetilde{\Sigma }_{\tau }$ and absorb the term on the left using that *a* is small.

By the above considerations it suffices to estimate for $\tau _2 > \tau _1+2$ the integral

125 $$\begin{aligned} \int _{\widetilde{{\mathcal {R}}} \left( \tau _1,\tau _2\right) } \Xi \cdot \text {Re} \left[ -T \overline{\Psi ^{[+2]}} {\mathcal {J}}^{[+2]}\right] \, , \end{aligned}$$

where $\Xi =\Xi _1\left( {\tilde{t}}^*\right) \Xi _2\left( r^*\right)$ is a smooth cutoff such that $\Xi _1$ is equal to 1 in $\left[ \tau _1+1,\tau _2-1\right]$ and vanishes for $\left( \tau _1,\tau _2\right) ^c$ while $\Xi _2$ is equal to 1 in $\left[ A^*_1,A^*_2\right]$ and vanishes in $\left( 2A^*_1,2A^*_2\right) ^c$. (Indeed, $1-\Xi$ is either supported away from trapping or in a strip of time-length 1, where one can estimate the spacetime integral of $T\Psi ^{[+2]}$ by the supremum of the energy through each slice $\widetilde{\Sigma }_{\tau }$ and absorb it on the left.) Note that now when integrating (125) by parts (in *T*, ${\underline{L}}$, *L*) there will be no boundary terms in view of the cut-off.

Let *c*(*r*) denote a general bounded real-valued function with bounded derivative in $(r_+,\infty )$. For the first term of ${\mathcal {J}}^{[+2]}$ inserted in (125) we have the identity (boundary terms vanish!)

126 $$\begin{aligned}&\int _{S^2} d\sigma \ \Xi c(r)\mathrm {Re}\left[ T\overline{\Psi ^{[+2]}}\Phi \left( \sqrt{\Delta }\psi ^{[+2]}\right) \right] = \int _{S^2}d\sigma (\Xi c(r))' \mathrm {Re}\left[ \overline{\Psi ^{[+2]}}\Phi \left( \sqrt{\Delta }\psi ^{[+2]}\right) \right] \nonumber \\&\quad + \int _{S^2}d\sigma \frac{c(r)}{2} \Xi \ \mathrm {Re}\left[ (L-{\underline{L}})\overline{\Psi ^{[+2]}}\Phi \left( \sqrt{\Delta }\psi ^{[+2]}\right) \right] \nonumber \\&\quad + \frac{1}{2} \int _{S^2}d\sigma \left( {\underline{L}} \left( \frac{\Xi ac(r)}{w(r^2+a^2)}\right) \right) \left| \Phi \left( \sqrt{\Delta }\psi ^{[+2]}\right) \right| ^2, \end{aligned}$$

obtained by exchanging $T,\Phi$, using the definition of ${\underline{L}}$ and the transformation (51). For the second term

127 $$\begin{aligned} \int _{S^2} d\sigma \ \Xi c(r)\mathrm {Re}\left[ T\overline{\Psi ^{[+2]}}\sqrt{\Delta } \psi ^{[+2]}\right] = -\int _{S^2} d\sigma \ \Xi c(r)\mathrm {Re}\left[ \overline{\Psi ^{[+2]}}T \sqrt{\Delta } \psi ^{[+2]}\right] \, , \end{aligned}$$

for the third

128 $$\begin{aligned}&\int _{S^2} d\sigma \ \Xi c(r)\mathrm {Re}\left[ T\overline{\Psi ^{[+2]}}\Phi \left( \Delta ^2(r^2+a^2)^{-3/2}\alpha ^{[+2]}\right) \right] = \nonumber \\&\quad +\int _{S^2} d\sigma \left( -{\underline{L}}\left( \frac{\Xi \ c(r)}{w}\right) \right) (r^2+a^2)^{-1/2}\mathrm {Re}\left[ T\left( \sqrt{\Delta }\overline{\psi ^{[+2]}}\right) \Phi \left( \Delta ^2(r^2+a^2)^{-1}\alpha ^{[+2]}\right) \right] \nonumber \\&\quad +\int _{S^2} d\sigma 2 \Xi \ c(r)\mathrm {Re}\left[ T\left( \sqrt{\Delta }\overline{\psi ^{[+2]}}\right) \Phi \left( \sqrt{\Delta }\overline{\psi ^{[+2]}}\right) \right] , \end{aligned}$$

obtained by using transformations (49) and (51) and integrating by parts, and for the last

129 $$\begin{aligned} \int _{S^2} d\sigma \ \Xi c(r)\mathrm {Re}\left[ T\overline{\Psi ^{[+2]}}\alpha ^{[+2]}\right] =&-\int _{S^2} d\sigma \ \Xi c(r)\mathrm {Re}\left[ \overline{\Psi ^{[+2]}}T\alpha ^{[+2]}\right] \nonumber \\&- \int _{S^2} d\sigma (T\Xi ) c(r)\mathrm {Re}\left[ \overline{\Psi ^{[+2]}} \alpha ^{[+2]}\right] . \end{aligned}$$

All terms on the right of (126)–(129) involve at most the non-degenerate derivative $\left( \Psi ^{[+2]}\right) ^\prime$, $\Psi ^{[+2]}$ itself and (at most) first derivatives of $\psi ^{[+2]}, \alpha ^{[+2]}$ and are hence easily controlled by Cauchy–Schwarz. We conclude

$$\begin{aligned}&{\mathbb {E}}_{\tilde{\Sigma }_\tau ,0}\left[ \Psi ^{[\pm 2]}\right] (\tau _2) \lesssim {\mathbb {E}}_{\tilde{\Sigma }_\tau ,0}\left[ \Psi ^{[\pm 2]}\right] (\tau _1) +|a|\sup _{\tau \in [\tau _1,\tau _1+1]\cup [\tau _2-1,\tau _2]}{\mathbb {E}}_{\widetilde{\Sigma }_\tau ,\eta }\left[ \Psi ^{[+2]}\right] (\tau )\\&\quad +|a|{\mathbb {I}}_0^{\mathrm {deg}}\left[ \Psi ^{[+2]}\right] (\tau _1,\tau _2)+|a|{\mathbb {I}}_\eta \left[ \psi ^{[+2]}\right] (\tau _1,\tau _2) + |a|{\mathbb {I}}_\eta \left[ \alpha ^{[+2]}\right] (\tau _1,\tau _2) \end{aligned}$$

for $\tau _2 > \tau _1+2$ while, as mentioned already above, for $\tau _2 \le \tau _1+2$ the same estimate holds replacing $\sup _{\tau \in [\tau _1,\tau _1+1]\cup [\tau _2-1,\tau _2]}$ by $\sup _{\tau \in [\tau _1,\tau _2]}$. Choosing $a_0$ sufficiently small we obtain the desired statement for $s=+2$ for every $\tau _1\le \tau _2$. As mentioned, for $s=-2$, the procedure can be repeated, now using the transformation (52). $\square$

##### Remark 5.3

A frequency localised version of this proof can be found in the proof of Proposition 8.4.1.

#### Local in Time Estimates

##### Proposition 5.3.2

Let $\upalpha ^{[\pm 2]}$ satisfy the homogeneous Teukolsky equation and let $\uppsi ^{[\pm 2]}$, $\Psi ^{[\pm 2]}$ be as defined in (46), (47), (49), (50). Then for any $\tau _{\mathrm{step}}>0$ there exists an $a_0\ll M$ such that for $|a|<a_0$ we have for any $\tau _1 >0$

130 $$\begin{aligned}&\sup _{\tau _1 \le \tau \le \tau _1+\tau _{\mathrm{step}}}{\mathbb {E}}_{\widetilde{\Sigma }_\tau , 0}\left[ \Psi ^{[\pm 2]}\right] (\tau )\nonumber \\&\quad \lesssim {\mathbb {E}}_{\widetilde{\Sigma }_\tau , 0}\left[ \Psi ^{[\pm 2]}\right] (\tau _1)+ |a|\tau _{\mathrm{step}}e^{C\tau _{\mathrm{step}}} {\mathbb {E}}_{\widetilde{\Sigma }_\tau , [\eta ]}\left[ \uppsi ^{[\pm 2]}\right] (\tau _1)\nonumber \\&\qquad + |a|\tau _{\mathrm{step}}e^{C\tau _{\mathrm{step}}} {\mathbb {E}}_{\widetilde{\Sigma }_\tau , [\eta ]}\left[ \upalpha ^{[\pm 2]}\right] (\tau _1), \end{aligned}$$

131 $$\begin{aligned}&{\mathbb {I}}_{0}\left[ \Psi ^{[\pm 2]}\right] (\tau _1, \tau _1+\tau _{\mathrm{step}})+ {\mathbb {I}}_{[\eta ]} \left[ \uppsi ^{[\pm 2]}\right] (\tau _1, \tau _1+\tau _{\mathrm{step}}) + {\mathbb {I}}_{[\eta ]}\left[ \upalpha ^{[\pm 2]}\right] (\tau _1, \tau _1+\tau _{\mathrm{step}})\nonumber \\&\quad \lesssim \tau _{\mathrm{step}} {\mathbb {E}}_{\widetilde{\Sigma }_\tau , 0}\left[ \Psi ^{[\pm 2]}\right] (\tau _1)+ {\mathbb {E}}_{\widetilde{\Sigma }_\tau , [\eta ]}\left[ \uppsi ^{[\pm 2]}\right] (\tau _1) + {\mathbb {E}}_{\widetilde{\Sigma }_\tau , [\eta ]}\left[ \upalpha ^{[\pm 2]}\right] (\tau _1) \end{aligned}$$

where $C=C(M)$ (and the implicit constant in $\lesssim$ is independent of both $\tau _{\mathrm{step}}$ and $\tau _1$, according to our general conventions). Here the subindex $\left[ \eta \right]$ is equal to $\eta$ in case of $s=+\,2$ and it is dropped entirely in case $s=-\,2$.

##### Proof

We first note that

132 $$\begin{aligned}&\sup _{\tau _1\le \tau \le \tau _1+\tau _{\mathrm{step}}} \left( {\mathbb {E}}_{\widetilde{\Sigma }_\tau , 0}\left[ \Psi ^{[\pm 2]}\right] (\tau ) + {\mathbb {E}}_{\widetilde{\Sigma }_\tau , [\eta ]}\left[ \uppsi ^{[\pm 2]}\right] (\tau ) +{\mathbb {E}}_{\widetilde{\Sigma }_\tau , [\eta ]}\left[ \upalpha ^{[\pm 2]}\right] (\tau )\right) \nonumber \\&\quad \lesssim e^{C\tau _{\mathrm{step}}}\left( {\mathbb {E}}_{\widetilde{\Sigma }_\tau , 0}\left[ \Psi ^{[\pm 2]}\right] (\tau _1) + {\mathbb {E}}_{\widetilde{\Sigma }_\tau , [\eta ]}\left[ \uppsi ^{[\pm 2]}\right] (\tau _1) + {\mathbb {E}}_{\widetilde{\Sigma }_\tau , [\eta ]}\left[ \upalpha ^{[\pm 2]}\right] (\tau _1)\right) . \end{aligned}$$

This follows easily by the estimates of the previous sections.

We now apply (122) with $\tau _2$ taken in $\tau _1\le \tau _2 \le \tau _1+\tau _{\mathrm{step}}$, noting that the first three terms on the right hand side can be bounded by $|a|\tau _{\mathrm{step}}$ times the right hand side of (132). Restricting $a_0$ so that in particular $|a|\tau _{\mathrm{step}}e^{C\tau _{\mathrm{step}}} < 1$ we obtain (130).

We note that we can repeat the transport estimates of Sect. 5.2, now for the homogeneous equations, and applied globally in $\widetilde{{\mathcal {R}}}(\tau _1,\tau _1+\tau _{\mathrm{step}})$, obtaining

$$\begin{aligned}&{\mathbb {I}}_{[\eta ]}\left[ \uppsi ^{[\pm 2]}\right] (\tau _1, \tau _1+\tau _{\mathrm{step}}) + {\mathbb {I}}_{[\eta ]}\left[ \upalpha ^{[\pm 2]}\right] (\tau _1, \tau _1+\tau _{\mathrm{step}})\\&\quad \lesssim {\mathbb {E}}_{\widetilde{\Sigma }_\tau , [\eta ]}\left[ \uppsi ^{[\pm 2]}\right] (\tau _1) + {\mathbb {E}}_{\widetilde{\Sigma }_\tau , [\eta ]}\left[ \upalpha ^{[\pm 2]}\right] (\tau _1)\\&\qquad + {\mathbb {I}}_\eta \left[ \Psi ^{[\pm 2]}\right] (\tau _1, \tau _1+\tau _{\mathrm{step}}). \end{aligned}$$

Note that the term is ${\mathbb {I}}_\eta$ and not ${\mathbb {I}}^{\mathrm{deg}}_\eta$.

In view of

$$\begin{aligned}&{\mathbb {I}}_\eta \left[ \Psi ^{[\pm 2]}\right] (\tau _1, \tau _1+\tau _{\mathrm{step}}) \lesssim \int _{\tau _1}^{\tau _1+\tau _{\mathrm{step}}} {\mathbb {E}}_{\widetilde{\Sigma }_\tau , 0}\left[ \Psi ^{[\pm 2]}\right] (\tau ) d\tau \\&\quad \lesssim \tau _{\mathrm{step}} \sup _{\tau _1\le \tau \le \tau _1+\tau _{\mathrm{step}}} {\mathbb {E}}_{\widetilde{\Sigma }_\tau , 0}\left[ \Psi ^{[\pm 2]}\right] (\tau ) \end{aligned}$$

(note the $\eta$ on the left but the 0 on the right hand side), we obtain (131) for sufficiently small *a*. $\square$

##### Remark 5.4

We note that a more careful examination of the Schwarzschild case and Cauchy stability yields that the inequality (131) can be proven without the $\tau _{\mathrm{step}}$ factor on the first term of right hand side, provided ${\mathbb {I}}_0$ is replaced by ${\mathbb {I}}_0^{\mathrm{deg}}$. We shall not however require this here.

## The Admissible Class and Teukolsky’s Separation

In this section we will implement Teukolsky’s separation [107] of (36) for $s=\pm \,2$.

To make sense *a priori* of the formal separation of [107], one must in particular work in a class of functions for which one can indeed take the Fourier transform in time. This requires applying the analysis to functions which satisfy certain time-integrability properties. A useful such class is the “sufficiently integrable, outgoing” class defined in [44, 45] for the $s=0$ case.

In the present paper, it turns out that we shall only require Fourier analysis in the region $r\in [A_1,A_2]$. We may thus consider the more elementary setting of what we shall call the $[A_1,A_2]$-*admissible class* where time square integrability is only required for $r\in [A_1,A_2]$. (We will in fact assume compact support in $t^*$ in this *r*-range.) This leads to a number of useful simplifications. In particular, we need not refer to the asymptotic analysis of the ODE’s as $r^*\rightarrow \pm \infty$, as was done in [44, 45], in order to infer boundary behaviour.

The section is organised as follows: We will define our elementary notion of $[A_1,A_2]$-admissible class in Sect. 6.1. We will then implement Teukolsky’s separation in Sect. 6.2, deriving the radial ODE, valid for $r\in [A_1,A_2]$.

(We note already that, in practice, the results of this section will be applied to solutions of the inhomogeneous Teukolsky equation which arises from applying a suitable cutoff to solutions of (37). The restriction of Fourier analysis to the range $r^*\in [A_1^*,A_2^*]$ will allow us to use a cutoff whose derivatives are supported in a region of finite $r^*\in [2A_1^*,2A_2^*]$, leading to additional simplifications with respect to [45]. We will only turn to this in Sect. 9.)

### The $[A_1,A_2]$-admissible Class

We define an admissible class of functions for our frequency analysis. This is to be compared with the class of *sufficiently integrable* functions from [44, 45]. Since we will only apply frequency localisation in a neighbourhood of trapping, we only consider the behaviour in the fixed *r*-region $[A_1,A_2]$ with $r_+<A_1<A_2<\infty$ defined in Sect. 2.1.3. (Recall in this region that $t=t^*={\tilde{t}}^*$.) On the other hand, for convenience, we will assume compact support in *t* for these *r*-values, as this is what we shall indeed obtain after applying cutoffs.

#### Definition 6.1

Let $a_0<M$, $|a|<a_0$ and let $g=g_{a,M}$. We say that a smooth complex valued spin$\pm \,2$ weighted function ${\tilde{\upalpha }}:{\mathcal {R}}\cap \{A_1\le r\le A_2\} \rightarrow {\mathbb {C}}$ is $[A_1,A_2]$-*admissible* if it is compactly supported in *t*.

#### Remark 6.1

One could work with the weaker condition that (cf. [45]) for all $j\ge 1$, the following holds

133 $$\begin{aligned}&\sup _{r\in [A_1,A_2]} \int _{-\infty }^\infty \int _{{\mathbb {S}}^2} \sum _{0\le i_1+i_2+i_3+i_4+i_5\le j} \left| ({\tilde{Z}}_1)^{i_1}({\tilde{Z}}_2)^{i_2}({\tilde{Z}}_3)^{i_3}T^{i_4}(\partial _r)^{i_5}{\tilde{\upalpha }} \right| ^2 \nonumber \\&\quad \times \sin \theta \, dt\, d\theta \, d\phi <\infty , \end{aligned}$$

with the only caveat that in the frequency analysis we would have to restrict to generic frequency $\omega$ for the ODE to be satisfied in the classical sense.

### Teukolsky’s Separation

We will now implement Teukolsky’s formal separation of the operator (36) in the context of $[A_1,A_2]$-admissible spin-*s* weighted functions $\upalpha ^{[s]}$ for $s=\pm \,2$.

We begin in Sect. 6.2.1 with a review of the basic properties of spin-weighted oblate spheroidal harmonics and their associated eigenvalues $\lambda ^{[s]}_{m\ell }(\nu )$. We will then turn immediately in Sect. 6.2.2 to some elementary estimates for the eigenvalues $\lambda ^{[s]}_{m\ell }(\nu )$ which will be useful later in the paper. Next, we shall apply these oblate spheroidals together with the Fourier transform in time in Sect. 6.2.3 to define coefficients $\upalpha ^{[s],(a\omega )}_{m\ell }(r)$ associated to $[A_1,A_2]$-admissible $\upalpha ^{[s]}$. We then give Proposition 6.2.1 in Sect. 6.2.4, stating that these coefficients satisfy an ordinary differential equation with respect to $r^*$; this is the content of Teukolsky’s remarkable separation of (36).

#### Spin-Weighted Oblate Spheroidal Harmonics

Let $\nu \in {\mathbb {R}}$, $s=0,\pm 2$ and consider the self-adjoint operator  defined byon $\mathscr {S}^{[s]}_\infty$, which we recall is a dense subset of $L^2(\sin \theta \, d\theta \, d\phi )$.

This has a complete collection of eigenfunctions

134 $$\begin{aligned} \{ S^{[s]}_{m\ell }(\nu , \cos \theta )e^{im\phi }\}_{m\ell } \end{aligned}$$

with eigenvalues $\lambda ^{[s]}_{m\ell }\in {\mathbb {R}}$, indexed by $m\in {\mathbb {Z}}$, $\ell \ge \mathrm{max}( |m|,|s|)$. These are known as the spin-weighted oblate^10^ spheroidal harmonics. For each fixed $m\in {\mathbb {Z}}$, the $S^{[s]}_{m\ell }$ themselves form a complete collection of eigenfunctions of the following self-adjoint operator with eigenvalues $\lambda ^{[s]}_{m \ell } \left( \nu \right)$:135
136The eigenfuctions themselves satisfy

$$\begin{aligned} S^{[s]}_{m\ell }(\nu , \cos \theta )e^{im\phi } \in \mathscr {S}_{\infty }^{[s]} \end{aligned}$$

for all $$\nu \in {\mathbb {R}}$$.

We note the following familiar special cases:

1. For $s=0$ one obtains the oblate spheroidal harmonics familiar from the angular part of the separation equation of the scalar wave equation on Kerr [45]. The case $s=0$ and $\nu =0$ recovers the standard spherical harmonics $S_{m \ell }^{[0]}(0,\cos \theta )e^{im\phi }=Y_{m \ell }$ with eigenvalues $\ell \left( \ell +1\right)$.
2. For $\nu =0$, then  is the spin-*s*-weighted Laplacian and one obtains the spin-weighted spherical harmonics, whose eigenvalues can also be determined explicitly
  137 $$\begin{aligned} \lambda _{m \ell }^{[s]} \left( 0\right) + s = \lambda _{m \ell }^{[-s]} \left( 0\right) -s = \ell \left( \ell +1\right) - s^2 \ge 2 \end{aligned}$$
  where the last inequality follows from the relation $|\ell |\ge |s|$. For future reference we note the relation 138

We finally remark also the general relation

139 $$\begin{aligned} \lambda _{m \ell }^{[s]} \left( \nu \right) + s = \lambda _{m \ell }^{[-s]} \left( \nu \right) -s \end{aligned}$$

allowing us to restrict to $s=+\, 2$ without loss of generality when obtaining estimates on the $\lambda _{m \ell }^{[s]} \left( \nu \right)$.

For various asymptotics concerning the behaviour of $\lambda _{m\ell }^{[s]}$ see [10].

#### Estimates on $\lambda ^{[s]}_{m\ell } \left( \nu \right)$ and ${\widetilde{\Lambda }}^{[s]}_{m \ell }\left( \nu \right)$

To estimate $\lambda _{m\ell }^{[s]} \left( \nu \right)$ we compute from (136)

140 $$\begin{aligned} \lambda ^{[s]}_{m \ell }\left( \nu \right) +s =&\int _0^\pi \int _0^{2\pi } d\phi \, d\theta \sin \theta \nonumber \\&\times \left[ \big | \partial _\theta \Xi ^{[s]} \big |^2 + \left( \frac{ \left( m+ s \cos \theta \right) ^2}{\sin ^2 \theta } -\nu ^2 \cos ^2\theta +2s\nu \cos \theta \right) |\Xi ^{[s]}|^2\right] \, , \end{aligned}$$

where $\Xi ^{[s]}$ denotes (shorthand instead of the full (134)) a normalised eigenfunction of the operator  with eigenvalue $\lambda _{m \ell }^{[s]}\left( a\omega \right)$. Using the variational characterisation of the lowest eigenvalue of the operator  (which is 2 for $m=0,1$ and $m\left( m+1\right) -4$ for $m\ge 2$ by (137) and the relation $|m|\le \ell$) we conclude for

141 $$\begin{aligned} \widetilde{\Lambda }^{[\pm 2]}_{m \ell }\left( \nu \right) := \lambda ^{[s]}_{m \ell }\left( \nu \right) +s + \nu ^2 + 4|\nu | \, \end{aligned}$$

the bound

142 $$\begin{aligned} \widetilde{\Lambda }^{[\pm 2]}_{m \ell }\left( \nu \right) \ge \max \left( 2, m(m+1)-4\right) \, . \end{aligned}$$

Our ode estimates in Sect. 8 will only require (142). This motivates the following

##### Definition 6.2

A triple $(\omega , m, \widetilde{\Lambda })$ will be said to be admissible if $\omega \in {\mathbb {R}}$, $m\in {\mathbb {Z}}$ and ${\widetilde{\Lambda }}\in {\mathbb {R}}$ satisfies $\widetilde{\Lambda } \ge \mathrm{max}(2, m(m+1)-4)$.

#### The Coefficients $\upalpha ^{[s],(a\omega )}_{m\ell }$ and the Plancherel Relations

Given parameters *a*, *M* and *s*, we let $\upalpha ^{[s]}$ be $[A_1,A_2]$-admissible according to Definition 6.1.

We have

143 $$\begin{aligned} \upalpha ^{[s]}(t,r,\theta ,\phi )=\frac{1}{2\pi } \int _{-\infty }^\infty e^{-i\omega t} {\hat{\upalpha }}^{[s]} (\omega , r,\theta ,\phi )d\omega . \end{aligned}$$

Setting $\nu =a\omega$, for each $\omega \in {\mathbb {R}}$ we may decompose

144 $$\begin{aligned} {\hat{\upalpha }}^{[s]}(\omega , r, \theta , \phi ) =\sum _{m\ell } \upalpha ^{[s],(a\omega )}_{m\ell } S^{[s]}_{m,\ell } (a\omega ,\cos \theta )e^{im\phi }. \end{aligned}$$

We obtain then the representation

145 $$\begin{aligned} \upalpha ^{[s]} (t,r,\theta , \phi ) = \frac{1}{\sqrt{2\pi }} \int _{-\infty }^\infty \sum _{m\ell } e^{-i\omega t} \upalpha ^{[s], (a\omega )}_{m\ell }(r) S^{[s]}_{m\ell }(a\omega , \cos \theta )e^{im\phi } d\omega .\qquad \end{aligned}$$

As in [45], we remark that for each fixed *r*, (143) and (145) are to be understood as holding in $L^2_tL^2_{{\mathbb {S}}^2}$, while (144) is to be understood in $L^2_\omega L^2_{{\mathbb {S}}^2}$. Note that if $\upalpha ^{[s]}$ satisfies Definition 6.1, then so do $\partial _t \upalpha ^{[s]}$ and $\partial _\phi \upalpha ^{[s]}$ and we have

$$\begin{aligned} \partial _t \upalpha ^{[s]}(t,r,\theta ,\phi )= & {} \frac{-i}{2\pi } \int _{-\infty }^\infty \omega e^{-i\omega t} {\hat{\upalpha }}^{[s]} (\omega , r,\theta ,\phi )d\omega \, ,\\ \partial _\phi \upalpha ^{[s]}(t,r,\theta ,\phi )= & {} \frac{i}{2\pi } \int _{-\infty }^\infty m e^{-i\omega t} {\hat{\upalpha }}^{[s]} (\omega , r,\theta ,\phi )d\omega \, , \end{aligned}$$

where these relations are to be interpreted in $L^2_tL^2_{{\mathbb {S}}^{2}}$.

We also recall as in [40, 45] the following Plancherel relations

$$\begin{aligned}&\int _0^{2\pi }\int _0^\pi \int _{-\infty }^\infty \left| \upalpha ^{[s]}\right| ^2(t,r,\theta ,\phi )\sin \theta \, d\phi \, d\theta \, dt =\int _{-\infty }^{\infty } \sum _{m\ell }\left| \upalpha ^{[s], (a\omega )}_{m\ell }(r)\right| ^2 d\omega \,,\\&\int _0^{2\pi }\int _0^\pi \int _{-\infty }^\infty {}_{1}\upalpha ^{[s]}\cdot {}_2\bar{\upalpha }^{[s]} \sin \theta \, d\phi \, d\theta \, dt =\int _{-\infty }^{\infty } \sum _{m\ell }{}_1\upalpha ^{[s], (a\omega )}_{m\ell }\cdot {}_2\bar{\upalpha }^{[s], (a\omega )}_{m\ell } d\omega \, ,\\&\int _0^{2\pi }\int _0^\pi \int _{-\infty }^\infty \left| \partial _r \upalpha ^{[s]}\right| ^2(t,r,\theta ,\phi )\sin \theta \, d\phi \, d\theta \, dt =\int _{-\infty }^{\infty } \sum _{m\ell }\left| \frac{d}{dr}\upalpha ^{[s], (a\omega )}_{m\ell }(r)\right| ^2 d\omega \, ,\\&\int _0^{2\pi }\int _0^\pi \int _{-\infty }^\infty \left| \partial _t \upalpha ^{[s]}\right| ^2(t,r,\theta ,\phi )\sin \theta \, d\phi \, d\theta \, dt =\int _{-\infty }^{\infty } \sum _{m\ell }\omega ^2\left| \upalpha ^{[s], (a\omega )}_{m\ell }(r)\right| ^2 d\omega \, , \end{aligned}$$

as well as

146 $$\begin{aligned}&\int _0^{2\pi }\int _0^\pi \int _{-\infty }^\infty \left( \left| \frac{\partial \upalpha ^{[s]}}{\partial \theta }\right| ^2+\left| \left( \frac{\partial \upalpha ^{[s]}}{\partial \phi }+is \cos \theta \upalpha ^{[s]} \right) \sin ^{-1}\theta \right| ^2\right) \nonumber \\&\qquad \times (t,r,\theta ,\phi )\sin \theta \, d\phi \, d\theta \, dt \nonumber \\&\quad = \int _{-\infty }^\infty \sum _{m \ell } \left( \lambda _{m \ell }^{[s]} \left( a\omega \right) +s \right) \left| \upalpha ^{[s], (a\omega )}_{m\ell }(r)\right| ^2 d\omega \nonumber \\&\qquad + \int _0^{2\pi }\int _0^\pi \int _{-\infty }^\infty \left( a^2 \cos ^2 \theta | \partial _t \alpha ^{[s]}|^2 +\text {Re}(-2ias \cos \theta \partial _t \alpha ^{[s]} \overline{\alpha }^{[s]})\right) \nonumber \\&\qquad \times (t,r,\theta ,\phi )\sin \theta \, d\phi \, d\theta \, dt\, . \end{aligned}$$

From the inequalities of Sect. 6.2.2 we conclude147In what follows, we shall often write $\lambda _{m \ell }^{[s],(a\omega )}$ for $\lambda _{m \ell }^{[s]} \left( a\omega \right)$ and ${\widetilde{\Lambda }}_{m \ell }^{[s],(a\omega )}$ for ${\widetilde{\Lambda }}_{m \ell }^{[s]} \left( a\omega \right)$.

#### The Radial ODE

We here state a proposition that implements Teukolsky’s formal separation of (36) in the context of $[A_1,A_2]$-admissible spin-weighted functions.

Fix $|a|<M$ and $s=0,\pm \,2$. Let $\upalpha ^{[s]}$ be an $[A_1,A_2]$-admissible spin weighted functions and $\upalpha ^{[s], (a\omega )}_{m\ell }$ be as defined in Sect. 6.2.3. Note that (recall (38)) defining

148 $$\begin{aligned} F^{[+2]} = \widetilde{{\mathfrak {T}}}^{[+2]} \tilde{\upalpha }^{[+2]} \ \ \ , \ \ \ \Delta ^2 F^{[-2]} = \widetilde{{\mathfrak {T}}}^{[-2]} \left( \Delta ^2 \tilde{\upalpha }^{[-2]}\right) \end{aligned}$$

we have that $F^{[s]}$ is also $[A_1,A_2]$-admissible and the coefficients $F^{[s],(a\omega )}_{m\ell }$ can be defined.

Let us first introduce the following shorthand notation

$$\begin{aligned} \kappa = \left( r^2+a^2\right) \omega - am \end{aligned}$$

and

149 $$\begin{aligned} \Lambda ^{[s],(a\omega )}_{m \ell } = \lambda _{m\ell }^{[s], (a\omega )} + a^2 \omega ^2 -2am\omega . \end{aligned}$$

We have the following

##### Proposition 6.2.1

Fix $|a|<M$ and $s=0,\pm 2$. Let $\upalpha ^{[s]}$ be an $[A_1,A_2]$-admissible spin weighted function, $F^{[s]}$ be as defined in (148), with coefficients $\upalpha ^{[s], (a\omega )}_{m\ell }$, $(\rho ^2F)^{[s], (a\omega )}_{m\ell }$ as defined above. Then $\upalpha ^{[s], (a\omega )}_{m\ell }$ is smooth in $r\in [A_1,A_2]$ and satisfies the ordinary differential equation

150 $$\begin{aligned}&\frac{1}{\Delta ^s} \frac{d}{dr} \left( \Delta ^{s+1} \frac{d\upalpha ^{[s], (a\omega )}_{m\ell }}{dr}\right) + \left( \frac{\kappa ^2-2is\left( r-M\right) \kappa }{\Delta } + 4is\omega r - \Lambda ^{[s], (a\omega )}_{m\ell } \right) \upalpha ^{[s], (a\omega )}_{m\ell } \nonumber \\&\quad = \frac{\left( r^2+a^2\right) ^{7/2}}{\rho ^2 \Delta ^{1+s/2}} F^{[s], (a\omega )}_{m\ell }. \end{aligned}$$

In view of our definitions, the proof is immediate from the usual formal derivation of (150). See [68]. The $s=0$ case corresponds precisely to Proposition 5.2.1 of [45].

Note the difference between (149) and our $\widetilde{\Lambda }^{[s],(a\omega )}_{m \ell }$ in (141). It is only the latter quantity which will appear in the estimates of this paper. We have retained (149) to faciliate comparison with the literature.

#### The Rescaled Coefficients *u*

Let us fix parameters $|a|<M$ and *s*, and consider $\upalpha ^{[s]}$ as in the statement of Proposition 6.2.1.

Define the rescaled^11^ quantities

151 $$\begin{aligned} u^{[s], (a\omega )}_{m\ell }(r)= & {} \Delta ^{s/2} \sqrt{r^2+a^2}\, \upalpha ^{[s], (a\omega )}_{m\ell }\left( r\right) , \end{aligned}$$

152 $$\begin{aligned} H^{[s], (a\omega )}_{m\ell }= & {} \frac{\Delta }{\rho ^2 w} F^{[s], (a\omega )}_{m\ell } . \end{aligned}$$

Equation (150) then reduces to

153 $$\begin{aligned} \frac{d^2}{(dr^*)^2} u^{[s], (a\omega )}_{m\ell } + V^{[s], (a\omega )}_{m\ell }\left( r^*\right) u =H^{[s], (a\omega )}_{m\ell } \end{aligned}$$

for

$$\begin{aligned} V^{[s], (a\omega )}_{m\ell }\left( r^*\right) =\frac{\Delta }{\left( r^2+a^2\right) ^2} {\tilde{V}}^{[s], (a\omega )}_{m\ell }+ V^{[s]}_{0}, \end{aligned}$$

with

$$\begin{aligned}&{\tilde{V}}^{[s], (a\omega )}_{m\ell } := \frac{\kappa ^2-2is\left( r-M\right) \kappa }{\Delta } + 4is\omega r - \Lambda ^{[s]}_{m\ell }, \\&V_{0}^{[s]} := \frac{\Delta ^{-s/2+1}}{\left( r^2+a^2\right) ^\frac{3}{2}} \frac{d}{dr} \left( \Delta ^{s+1} \frac{d}{dr} \left( \frac{\Delta ^{-s/2}}{\sqrt{r^2+a^2}}\right) \right) . \end{aligned}$$

For $s=0$, this reduces to the form of the separated wave equation used in [45].

## The Frequency-Localised Transformations

In this section, we will define frequency localised versions of the quantities $P^{[\pm 2]}$, $\Psi ^{[\pm 2]}$, $\uppsi ^{[\pm 2]}$ of Sect. 3 and the Regge–Wheeler type equation (54).

We begin in Sect. 7.1 with the definitions of the frequency localised version of the null frame *L*, ${\underline{L}}$. We then derive in Sect. 7.2 the frequency localised expression for $\Psi ^{[\pm 2]}$ followed in Sect. 7.3 with the frequency localised form of (54).

***In what follows in this section, we will always assume*** $\upalpha ^{[\pm 2]}$ ***is as in Proposition*** 6.2.1 ***with corresponding*** $u^{[\pm 2], (a\omega )}_{m\ell }$.

### The Separated Null Frame

Note that (following the conventions in [45]) we have the following formal analogues:

$$\begin{aligned} -\,i\omega \sim \partial _t,\\ im \sim \partial _\phi . \end{aligned}$$

We define the separated frame operators (corresponding to the principal null directions (20)) by

154 $$\begin{aligned} L= & {} \frac{d}{dr^*}-i\omega +\frac{iam}{r^2+a^2}, \end{aligned}$$

155 $$\begin{aligned} -{\underline{L}}= & {} \frac{d}{dr^*} +i\omega -\frac{iam}{r^2+a^2}. \end{aligned}$$

We have retained the notation of (20) without fear of confusion.

Also note that (138) implies the following formal analogue:


### The Frequency Localised Coefficients $P^{[\pm 2], (a\omega )}_{m\ell }, \Psi ^{[\pm 2], (a\omega )}_{m\ell }$ and $\psi ^{[\pm 2],(a\omega )}_{m\ell }$

We may now understand the relations between the quantities of Sect. 3.1 at the frequency localised level.

#### Proposition 7.2.1

Let ${\upalpha }^{[\pm 2]}$ be as in Proposition 6.2.1 and consider $P^{[+2]}$, $\Psi ^{[+2]}$ and $\uppsi ^{[+2]}$ defined by (46), (48) and (49), respectively, and consider $P^{[-2]}$, $\Psi ^{[-2]}$ and $\uppsi ^{[-2]}$ defined by (47), (48) and (50), respectively.

Let $u^{[\pm 2], (a\omega )}_{m\ell }$ be the arising coefficient of $\upalpha ^{[\pm 2]}$. Then $P^{[\pm 2]}$, $\Psi ^{[\pm 2]}$ and $\uppsi ^{[\pm 2]}$ are $[A_1,A_2]$-admissible spin weighted functions and their coefficients $P^{[\pm 2], (a\omega )}_{m\ell }$, $\Psi ^{[\pm 2], (a\omega )}_{m\ell }$ and $\psi ^{[\pm 2], (a\omega )}_{m\ell }$ are related by

156 $$\begin{aligned}&\left( r^2+a^2\right) \sqrt{w} \cdot {\psi }^{[+2], (a\omega )}_{m\ell } = - \frac{1}{2} \frac{1}{w}{\underline{L}} \left( u^{[+2], (a\omega )}_{m\ell } \cdot w\right) , \end{aligned}$$

157 $$\begin{aligned}&\Psi ^{[+2], (a\omega )}_{m\ell } = \left( r^2+a^2\right) ^{3/2} {P}^{[+2], (a\omega )}_{m\ell } = \frac{1}{w} {\underline{L}} \left( \left( r^2+a^2\right) \sqrt{w} \cdot \psi ^{[+2], (a\omega )}_{m\ell } \right) \nonumber \\&\quad = -\frac{1}{2} \frac{1}{w} {\underline{L}} \left( \frac{1}{w}{\underline{L}} \left( u^{[+2], (a\omega )}_{m\ell } \cdot w\right) \right) , \end{aligned}$$

158 $$\begin{aligned}&\left( r^2+a^2\right) \sqrt{w} \cdot {\psi }^{[-2], (a\omega )}_{m\ell } = \frac{1}{2} \frac{1}{w} L \left( {u}^{[-2],(a\omega )}_{m\ell } \cdot w\right) \, , \end{aligned}$$

159 $$\begin{aligned}&\Psi ^{[-2],(a\omega )}_{m\ell } = \left( r^2+a^2\right) ^{3/2} {P}^{[-2],(a\omega )}_{m\ell } =- \frac{1}{w} L \left( \left( r^2+a^2\right) \sqrt{w} \cdot {\psi }^{[-2],(a\omega )}_{m\ell } \right) \nonumber \\&\quad = -\frac{1}{2} \frac{1}{w} L \left( \frac{1}{w} L \left( {u}^{[-2],(a\omega )}_{m\ell } \cdot w\right) \right) . \end{aligned}$$

### The Frequency Localised Regge–Wheeler Equation (54) for $\Psi ^{[\pm 2],(a\omega )}_{m\ell }$

A straightforward computation now leads to

#### Proposition 7.3.1

Under the assumptions of Proposition 7.2.1, the ${\Psi }^{[\pm 2], (a\omega )}_{m\ell }$ satisfy the equation

160 $$\begin{aligned} \left( \Psi ^{[s],(a\omega )}_{m\ell }\right) ^{\prime \prime }&+ \left( \omega ^2 - {\mathcal {V}}^{[s],(a\omega )}_{m\ell } \right) \Psi ^{[s],(a\omega )}_{m\ell }= {\mathcal {J}}^{[s],(a\omega )}_{m\ell } + {\mathfrak {G}}^{[s],(a\omega )}_{m\ell } \, , \end{aligned}$$

where the potential ${\mathcal {V}}^{[s],(a\omega )}_{m\ell }$ is real and defined by

161 $$\begin{aligned} {\mathcal {V}}^{[s],(a\omega )}_{m\ell }&=\frac{ \Delta \left( \lambda ^{[s]}_{m \ell } +a^2\omega ^2+s^2+s\right) + 4Mram\omega -a^2 m^2 }{\left( r^2+a^2\right) ^2}\nonumber \\&\quad -\frac{\Delta }{(r^2+a^2)^2}\frac{6Mr (r^2-a^2)}{(r^2+a^2)^2} -7 a^2 \frac{\Delta ^2}{(r^2+a^2)^4} \nonumber \\&= {\mathcal {V}}^{[s]}_0 + {\mathcal {V}}_1 + {\mathcal {V}}_2 \, . \end{aligned}$$

and the inhomogeneous terms by

162 $$\begin{aligned}&{\mathcal {J}}^{[s],(a\omega )}_{m\ell } = \frac{\Delta }{\left( r^2+a^2\right) ^2} \left[ s\frac{-4r^2+4a^2}{r^2+a^2} aim -20a^2 \frac{r^3-3Mr^2+ra^2+Ma^2}{\left( r^2+a^2\right) ^2}\right] \nonumber \\&\quad \times \left( \sqrt{\Delta }\psi ^{[s],(a\omega )}_{m\ell } \right) \nonumber \\&\quad +a^2 \frac{\Delta }{\left( r^2+a^2\right) ^2} \left[ -6s \frac{r}{r^2+a^2} aim +3 \left( \frac{r^4 -a^4+10Mr^3-6Ma^2r}{(r^2+a^2)^2}\right) \right] \nonumber \\&\quad \times \left( u^{[s],(a\omega )}_{m\ell }\frac{\Delta }{\left( r^2+a^2\right) ^2}\right) , \nonumber \\&{\mathfrak {G}}^{[+2],(a\omega )}_{m\ell } =\frac{1}{2} {\underline{L}} \left( \frac{\left( r^2+a^2\right) ^2}{\Delta }{\underline{L}} \left( \frac{\Delta }{w\rho ^2} F^{[+2],(a\omega )}_{m\ell }\right) \right) , \nonumber \\&{\mathfrak {G}}^{[-2],(a\omega )}_{m\ell } =\frac{1}{2} {L} \left( \frac{\left( r^2+a^2\right) ^2}{\Delta }{L} \left( \frac{\Delta ^3}{w\rho ^2} F^{[-2],(a\omega )}_{m\ell }\right) \right) . \end{aligned}$$

#### Proof

See Appendix A. $$\square $$

#### Remark 7.1

Note that ${\mathcal {J}}^{[s]}$ vanishes for $a=0$. The second line of ${\mathcal {J}}^{[s]}$ contains only linear terms in *m* (i.e. corresponding to only first derivatives in physical space). The first line contains in this sense “first” and “zero” derivatives of $\psi ^{[s]}$ and hence at most (certain) “second” derivatives of $u^{[s]}$.

#### Remark 7.2

We may rewrite the potential

163 $$\begin{aligned} {\mathcal {V}}^{[\pm 2]}_0 = \frac{ \Delta \left( \widetilde{\Lambda }^{[\pm 2]}-4|a\omega | +4\right) + 4Mram\omega -a^2 m^2 }{\left( r^2+a^2\right) ^2}. \end{aligned}$$

Here we see the dependence in the spin is entirely contained in the definition of $\widetilde{\Lambda }^{[\pm 2]}$.

#### Remark 7.3

Let us note finally that if, for a fixed frequency triple $(\omega , m, \widetilde{\Lambda })$, *u* is simply assumed to be a smooth solution of the ODE (153) where $\lambda _{m\ell }^{[s]}(a\omega )$ is replaced by the quantity defined by $\widetilde{\Lambda }-s-(a\omega )^2-4|a\omega |$ in view of (141), and *P*, $\Psi$, $\psi$ are defined by relations (156), (157), (158), (159), then the identities of Proposition 7.3.1 again hold.

## Frequency-Localised Estimates in $r\in [A_1,A_2]$

The present section deals entirely with the system of relations satisfied by

$$\begin{aligned} u^{(a\omega )}_{m\ell }, \qquad \psi ^{(a\omega )}_{m\ell }, \qquad \Psi ^{(a\omega )}_{m\ell } \end{aligned}$$

at fixed frequency in the region $r\in [A_1,A_2]$, for given inhomogeneous terms. The main result will be Theorem 8.1, stated in Sect. 8.1, which can be thought of as a fixed frequency version of an integrated local energy estimate for all quantities near trapping, with boundary terms $\mathrm{Q}(A_i)$ which will eventually cancel the boundary terms appearing on the right hand side of Proposition 5.1.1 of Sect. 5.

We shall prove multiplier estimates for (160) in Sect. 8.2 and transport estimates for (156)–(159) in Sect. 8.3. Together with an integration by parts argument, the transport estimates will allow us to bound in Sect. 8.4 the inhomogeneous terms on the right hand side of (160) arising from the coupling of the Regge–Wheeler equation for $\Psi ^{(a\omega )}_{m\ell }$ with $u^{(a\omega )}_{m\ell }$ and $\psi ^{(a\omega )}_{m\ell }$, thus will allow to complete the proof of Theorem 8.1

Just like with the analogous Theorem 8.1 of [45], the results of this section can be understood as results about ODE’s, independently of the particular framework of Sect. 6. We have thus tried to give as self-contained a statement as possible.

### Statement of Theorem 8.1: The Main Fixed Frequency Estimates

In the present section we consider the coupled system of ODEs satisfied by *u*, $\psi$ and $\Psi$ and state a fixed frequency analogue of local integrated energy decay, in the region $r\in [A_1,A_2]$ near trapping.

#### Frequency Localised Norms

Before formulating the theorem, we define certain energy norms.

In view of Remark 7.3, the natural setting of the theorem refers only to an admissible frequency triple $(\omega , m, \widetilde{\Lambda })$ (cf. Definition 6.2) and associated solutions $u^{[\pm 2]}$ of (153) on $[A_1,A_2]$ and $\psi ^{[\pm 2]}$, $\Psi ^{[\pm 2]}$ defined by (156)–(159), where $\lambda _{m\ell }^{[s]}(a\omega )$ is replaced by the quantity defined by $\widetilde{\Lambda }-s-(a\omega )^2-4|a\omega |$ in view of (141). Recall that all derived ordinary differential identities follow, in particular (54), as does the estimate (142) of Sect. 6.2.2. In practice, of course, we will always apply this for $u^{[\pm 2]}$ equal to $u^{[\pm 2],(a\omega )}_{m\ell }$ and $\widetilde{\Lambda }$ equal to $\widetilde{\Lambda }^{[s],(a\omega )}_{m\ell }$.

Given the above, let us define the quantities

$$\begin{aligned} \Vert {\mathfrak {d}} \Psi ^{[\pm 2]}\Vert ^2&= \int _{A_1^*}^{A_2^*}\left[ \left| (\Psi ^{[\pm 2]})'\right| ^2 + \left( \left( 1-r^{-1}r_{\mathrm{trap}}\right) ^2\left( \omega ^2 + {\widetilde{\Lambda }}\right) + 1\right) \left| (\Psi ^{[\pm 2]})\right| ^2\right] \, dr^*,\\ \Vert {\mathfrak {d}}\psi ^{[\pm 2]}\Vert ^2&=\int _{A_1^*}^{A_2^*}(\omega ^2+m^2+1)|\psi ^{[\pm 2]}|^2 dr^*,\\ \Vert {\mathfrak {d}}u^{[\pm 2]}\Vert ^2&=\int _{A_1^*}^{A_2^*}(\omega ^2+m^2+1)|u^{[\pm 2]}|^2 dr^*, \end{aligned}$$

as well as the boundary energies for $i=1,2$:

$$\begin{aligned} \Vert {\mathfrak {d}}\psi ^{[\pm 2]}\Vert ^2(A_i)= & {} (\omega ^2+m^2+1)|\psi ^{[\pm 2]}(A_i)|^2, \\ \Vert {\mathfrak {d}}u^{[\pm 2]}\Vert ^2(A_i)= & {} (\omega ^2+m^2+1)|u^{[\pm 2]}(A_i)|^2. \end{aligned}$$

In the above, $r_{\mathrm{trap}}$ is a parameter depending on *M*, *a* and the frequency triple $(\omega , m, \widetilde{\Lambda })$ to be determined later. For “trapped” frequencies, we will have $r^*_{\mathrm{trap}} \in [A_1^*/4,A_2^*/4]$, but it will be important that in various high frequency but untrapped frequency ranges, we can take $r_{\mathrm{trap}}=0$.

Note that since this is a region of fixed finite *r*, bounded away from infinity and the horizon, no *r*-weights or $\Delta$-factors need appear in the above norms.

Finally, it will be convenient if we introduce the alternate notation

$$\begin{aligned} A_-:=A_1, \qquad A_+:=A_2 \end{aligned}$$

which will be useful when referring to boundary terms in contexts where the choice of term depends on the spin.

#### Statement of the Theorem

##### Theorem 8.1

Given $0\le a_0 \ll M$ sufficiently small, then the following is true.

Let $0\le a\le a_0$ and let $(\omega , m, \widetilde{\Lambda })$ be an admissible frequency triple. Let $E>1$ be the parameter fixed after Proposition 5.1.1. Given a parameter $\delta _1<1$, let $f_0$, $y_0$ be defined by (100) and (100) as in the proof of Proposition 5.1.1.

Then one can choose sufficiently small $\delta _1<1$ depending only on *M*, and functions *f*, *y* and an *r*-value $r_{\mathrm{trap}}$, depending on the parameters *a*, *M* and the frequency triple $(\omega , m, \widetilde{\Lambda })$ but satisfying the uniform bounds

164 $$\begin{aligned}&r_{\mathrm{trap}}=0\, {\mathrm{or }}\, r^*_{\mathrm{trap}}\in [A^*_1/4,A^*_2/4] \end{aligned}$$

165 $$\begin{aligned}&\left| f\right| +\left| f'\right| + \left| y\right| \lesssim 1, \end{aligned}$$

166 $$\begin{aligned}&f = f_0(r), \qquad y=y_0(r)\qquad \text { for }r^*\in [A_1^*/2,A_2^*/2]^c, \end{aligned}$$

such that, for all smooth solutions $u^{[\pm 2]}$ of (153) on $[A_1,A_2]$ and associated $\psi ^{[\pm 2]}$ and $\Psi ^{[\pm 2]}$, then

167 $$\begin{aligned}&\Vert {\mathfrak {d}} \Psi ^{[\pm 2]}\Vert ^2 \lesssim {\mathfrak {H}}^{[\pm 2]} +\mathrm{Q}(A_2)-\mathrm{Q}(A_1) + |a| \sum _{i=1}^2 ( \Vert {\mathfrak {d}}\psi ^{[\pm 2]}\Vert ^2(A_i) + \Vert {\mathfrak {d}}u^{[\pm 2]}\Vert ^2(A_{i})), \end{aligned}$$

168 $$\begin{aligned}&\Vert {\mathfrak {d}}\psi ^{[\pm 2]}\Vert ^2(A_{\mp }) + \Vert {\mathfrak {d}}u^{[\pm 2]}\Vert ^2(A_{\mp })+ \Vert {\mathfrak {d}}\psi ^{[\pm 2]}\Vert ^2 +\Vert {\mathfrak {d}}u^{[\pm 2]}\Vert ^2 \nonumber \\&\quad \lesssim {\mathfrak {H}}^{[\pm 2]}+\mathrm{Q}(A_2)-\mathrm{Q}(A_1) + \Vert {\mathfrak {d}}\psi ^{[\pm 2]}\Vert ^2(A_{\pm }) + \Vert {\mathfrak {d}}u^{[\pm 2]}\Vert ^2(A_{ \pm }), \end{aligned}$$

where

169 $$\begin{aligned}&{\mathfrak {H}}^{[\pm 2]}=\int _{A_1^*}^{A_2^*} {{\mathfrak {G}}}^{[\pm 2]} \cdot (f, y, E) \cdot (\Psi ^{[\pm 2]}, {\Psi ^{[\pm 2]}}')\, dr^* ,\nonumber \\&{{\mathfrak {G}}}^{[\pm 2]} \cdot (f, y, E) \cdot (\Psi ^{[\pm 2]}, {\Psi ^{[\pm 2]}}') \doteq -2f{\mathrm{Re}}\left( {\Psi ^{[\pm 2]}}'\overline{{{\mathfrak {G}}}^{[\pm 2]} }\right) -f'{\mathrm{Re}}\left( \Psi ^{[\pm 2]}\overline{{{\mathfrak {G}}}^{[\pm 2]} }\right) \nonumber \\&\quad -\,2y\text {Re}\left( {\Psi ^{[\pm 2]}}'\overline{{{\mathfrak {G}}}^{[\pm 2]} }\right) \nonumber \\&\quad +\,E\omega \mathrm{Im}\left( {{\mathfrak {G}}}^{[\pm 2]} \overline{\Psi ^{[\pm 2]}}\right) , \end{aligned}$$

and $\mathrm{Q}$ is given by (172).

### Multiplier Estimates for $\Psi ^{[\pm 2]}$

We begin in this section with frequency localised bounds for $\Psi ^{[\pm 2]}$. Frequency localisation is necessary to capture trapping, in the style of our previous [40]. The multipliers will be frequency independent at $r=A_1$ and $r=A_2$ and will in fact match exactly those applied in Sect. 5.1. This is ensured by (166). As a result, in the setting of Sect. 9, the boundary terms $\mathrm{Q}(A_i)$ which will appear below, after summation over frequencies, will exactly cancel the terms ${\mathbb {Q}}(A_i)$ appearing in Proposition 5.1.1.

Recall the quantity $\Vert {\mathfrak {d}} \Psi ^{[\pm 2]}\Vert ^2$ defined in Sect. 8.1.1. The main result of the section is the following:

#### Proposition 8.2.1

With the assumptions of Theorem 8.1, we have

170 $$\begin{aligned} \Vert {\mathfrak {d}} \Psi ^{[\pm 2]}\Vert ^2 \lesssim {\mathfrak {H}}^{[\pm 2]}+ {\mathcal {K}}^{[\pm 2]} + \mathrm{Q}(A_2)-\mathrm{Q}(A_1) \end{aligned}$$

where ${\mathcal {K}}^{[\pm 2]}$ is defined by

$$\begin{aligned} {\mathcal {K}}^{[\pm 2]}=\int _{A_1^*}^{A_2^*} {{\mathcal {J}}}^{[\pm 2]} \cdot (f, y, E) \cdot (\Psi ^{[\pm 2]}, {\Psi ^{[\pm 2]}}')\, dr^* , \end{aligned}$$

where

171 $$\begin{aligned}&{{\mathcal {J}}}^{[\pm 2]} \cdot (f, y, E) \cdot (\Psi ^{[\pm 2]}, {\Psi ^{[\pm 2]}}') \doteq -2f\text {Re}\left( {\Psi ^{[\pm 2]}}'\overline{{{\mathcal {J}}}^{[\pm 2]} }\right) \nonumber \\&\quad -f'\text {Re}\left( \Psi ^{[\pm 2]}\overline{{{\mathcal {J}}}^{[\pm 2]} }\right) -2y\text {Re}\left( {\Psi ^{[\pm 2]}}'\overline{{{\mathcal {J}}}^{[\pm 2]} }\right) \nonumber \\&\quad +E\omega \text {Im}\left( {{\mathcal {J}}}^{[\pm 2]} \overline{\Psi ^{[\pm 2]}}\right) \end{aligned}$$

and $\mathrm{Q}$ is given by (172).

The estimate above differs from the estimate for $\Vert {\mathfrak {d}} \Psi ^{[\pm 2]}\Vert ^2$ given by (167) as it is still coupled with $u^{[\pm 2]}$ and $\psi ^{[\pm 2]}$ in view of the presence of the term ${\mathcal {K}}^{[\pm 2]}$. We will be able to replace ${\mathcal {K}}^{[\pm 2]}$ with ${\mathfrak {H}}^{[\pm 2]}$ and the additional boundary term $|a|\Vert {\mathfrak {d}}\psi ^{[\pm 2]}\Vert ^2(A_{ \pm }) + |a|\Vert {\mathfrak {d}}u^{[\pm 2]}\Vert ^2(A_{ \pm })$ appearing in (167) in Sect. 8.4.

#### Proof

The estimate (170) will be proven by using multiplier identities. The relevant frequency-localised current templates, corresponding precisely to the physical space multiplier identities used in Sect. 5.1, will be defined in Sect. 8.2.1 below. For a specific combination of these currents, the bulk term will control the integrand of the left hand side of (170) whereas the boundary terms (after summation over frequencies) will correspond precisely to the boundary terms of Proposition 5.1.1. This coercivity is stated as Proposition 8.2.2 in Sect. 8.2.2. The precise choice of the functions *f* and *y* will be frequency dependent and is carried out separately for the frequency ranges ${\mathcal {G}}_1$ and ${\mathcal {G}}_2$ in Sects. 8.2.3 and 8.2.4 respectively.

***In the rest of this subsection, we will always write*** $\Psi$ ***in the place of*** $\Psi ^{[\pm 2]}$, ***as the choice of the multipliers will not depend on the spin. We will write*** ${\mathcal {V}}$ ***in place of*** ${\mathcal {V}}^{[\pm 2]}$, ***and*** $\widetilde{\Lambda }$ ***for*** ${\widetilde{\Lambda }}^{[\pm 2]}$, ***remembering that the dependence of*** ${\mathcal {V}}^{[\pm 2]}$ ***on the spin in the context of the separation is completely contained in the different definition of ***${\widetilde{\Lambda }}^{[\pm 2]}$; ***see formula ***(163). We will only refer explicitly to $s=\pm \,2$ when discussing the inhomogeneous terms on the right hand side of (160).

#### The Frequency-Localised Multiplier Current Templates

Let us define the frequency localised multiplier currents which correspond to the physical space multipliers of Sect. 5.1:

$$\begin{aligned} \mathrm{Q}^f[\Psi ]= & {} f\left( |\Psi '|^2+(\omega ^2-{\mathcal {V}})|\Psi |^2\right) +f^\prime \mathrm{Re}\left( \Psi '\bar{\Psi }\right) -\frac{1}{2} f''|\Psi |^2,\\ \mathrm{Q}^y[\Psi ]= & {} y\left( |\Psi '|^2+(\omega ^2-{\mathcal {V}})|\Psi |^2\right) ,\\ \mathrm{Q}^T[\Psi ]= & {} -\omega \mathrm{Im}(\Psi ' \bar{\Psi }). \end{aligned}$$

If $$\Psi $$ satisfies

$$\begin{aligned} \Psi '' +{\mathcal {V}}\Psi = H \end{aligned}$$

for an admissible frequency triple $(\omega , m, \widetilde{\Lambda })$, then, since ${\mathcal {V}}$ is real, we have

$$\begin{aligned} (\mathrm{Q}^f[\Psi ])'&= 2f' |\Psi '|^2-f{\mathcal {V}}' |\Psi |^2 -\frac{1}{2}f'''|\Psi |^2 +\mathrm{Re}(2f{\bar{H}}\Psi ' +f'{\bar{H}} \Psi ),\\ (\mathrm{Q}^y[\Psi ])'&= y'(|\Psi '|^2+(\omega ^2-{\mathcal {V}})|\Psi |^2)-y{\mathcal {V}}'|\Psi |^2 +2y\mathrm{Re}({\bar{H}}\Psi '),\\ (\mathrm{Q}^T[\Psi ])'&= -\omega \mathrm{Im}(H{\bar{\Psi }}). \end{aligned}$$

Let us remark already that if $\upalpha$ is an $[A_1,A_2]$-admissible solution of the inhomogeneous Teukolsky equation (53), such that the restriction of $\upalpha$ to $r\in [A_1,A_2]$ is supported in $t=t^*={\tilde{t}}^*\in (\tau _1,\tau _2)$, then the identity corresponding to applying

$$\begin{aligned} \int d\omega \sum _{m\ell } \end{aligned}$$

to

$$\begin{aligned} \mathrm{Q}^f(A_1)+\int _{A_1^*}^{A_2^*} (\mathrm{Q}^f)'(r^*) dr^*=\mathrm{Q}^f(A_2), \end{aligned}$$

resp. with $\mathrm{Q}^y$, $\mathrm{Q}^T$, yields precisely the identities of Sect. 5.1.1 applied in the region $\widetilde{{\mathcal {R}}}^{\mathrm{trap}}(\tau _1, \tau _2)$. (Note that by our choices from Sect. 2.1.3, we have $T=T+\upomega _+\chi \Phi$ in this region, and note moreover that the boundary terms on ${\tilde{t}}^*=\tau _i$ vanish by the restriction on the support.)

#### The Total Current $\mathrm{Q}$ and Its Coercivity Properties

For all frequencies, we will apply the identity corresponding to a current of the form

172 $$\begin{aligned} \mathrm{Q}= \mathrm{Q}^f+ \mathrm{Q}^y + E\mathrm{Q}^T, \end{aligned}$$

for appropriate choices of functions *f*, *y*. The coercivity statement is given by the following:

##### Proposition 8.2.2

Let *E* and $f_0$ be as fixed in the proof of Proposition 5.1.1. Then one can choose $\delta _1<1$ sufficiently small, depending only on *M*, such that the following is true:

There exist functions *f* and *y* and a parameter $r_{\mathrm{trap}}$ depending on the parameters *a*, *M* and the frequency triple $(\omega , m, \widetilde{\Lambda })$, satisfying (164), (165) and (166) and such that $\mathrm{Q}$ defined by (172) satisfies

173 $$\begin{aligned}&\left| \Psi '\right| ^2 + \left( \left( 1-r_{\mathrm{trap}}r^{-1}\right) ^2\left( \omega ^2 + {\widetilde{\Lambda }}\right) + 1\right) \left| \Psi \right| ^2\nonumber \\&\qquad \lesssim \mathrm{Q}' - {\mathcal {J}}^{[\pm 2]}\cdot (f,y,E)\cdot (\Psi , \Psi ') -{\mathfrak {G}}^{[\pm 2]}\cdot (f,y,E)\cdot (\Psi , \Psi '). \end{aligned}$$

##### Proof

See Sects. 8.2.3 and 8.2.4 . $\square$

Let us note that integrating the equation

$$\begin{aligned} \mathrm{Q}(A_1) +\int _{A_1^*}^{A_2^*} \mathrm{Q}'(r^*) dr^* = \mathrm{Q}(A_2) \end{aligned}$$

we infer from (173) the inequality (170).

#### The ${\mathcal {G}}_1$ Range

We define the range

174 $$\begin{aligned} {\mathcal {G}}_1= \{{\widetilde{\Lambda }} \ge c_{\flat } \omega ^2 \} \cup \{ {\widetilde{\Lambda }} +\omega ^2 +m^2 \le C_{\sharp }\} \end{aligned}$$

for some $0<c_{\flat }<1$ and $C_{\sharp }>1$ which can be chosen finally to depend only on *M*. The frequency range ${\mathcal {G}}_1$ includes thus “angular-dominated frequencies” $\widetilde{\Lambda }\gg \omega ^2$, “trapped frequencies” $\widetilde{\Lambda }\sim \omega ^2$ and “low frequencies” ${\widetilde{\Lambda }} +\omega ^2 +m^2 \lesssim 1$. We have the following:

##### Proposition 8.2.3

For sufficiently small $|a|<a_0\ll M$, then for all frequency triples in ${\mathcal {G}}_1$, there exists a function *f* and a parameter $r_{\mathrm{max}}$ with the following properties for $r^*\in [A_1^*,A_2^*]$:

1. $f=f_0$ for $r^*\in [A_1^*/2,A_2^*/2]^c$ and $|f|\lesssim 1$, $|f'| \lesssim 1$ in $[A_1^*,A_2^*]$,
2. $|r_{\mathrm{max}}-3M | \le c(a,M)$ with $c(a, M)\rightarrow 0$ as $a\rightarrow 0$, in particular $a_0$ can be chosen so that $r_{\mathrm{trap}}^*\in [A_1^*/4,A_2^*/4]$; for $m=0$, $r_{\mathrm{max}}$ is independent of $\omega$ and $\widetilde{\Lambda }$,
3. $$f' \gtrsim 1 $$,
4. $$-f{\mathcal {V}}'-\frac{1}{2} f'' \gtrsim \left( {\widetilde{\Lambda }} (1-r_{\mathrm{max}}r^{-1})^2 +1 \right) $$.

##### Proof

Let ${\mathcal {V}}_{Schw}^{[\pm 2]}$ denote the potential ${\mathcal {V}}$ of (161) in the $a=0$ Schwarzschild case. Writing this potential as in (161) as

$$\begin{aligned} {\mathcal {V}}_{Schw}=({\mathcal {V}}_{Schw})_0+({\mathcal {V}}_{Schw})_1, \end{aligned}$$

we see easily that $({\mathcal {V}}_{Schw})_0$ has a unique maximum at $r=3M$, while

$$\begin{aligned}&f_0'\gtrsim r(r-2M) r^{-4}, \qquad -f_0{\mathcal {V}}_{Schw}'-\frac{1}{2}f_0''' \\&\quad \gtrsim c r(r-2M) \left( \frac{(r-3M)^2}{r^2}\ell (\ell +1)+1 \right) r^{-5}, \end{aligned}$$

so in particular, in the region $r^*\in [A_1^*,A_2^*]$, we have

$$\begin{aligned} f_0'\gtrsim 1 ,\qquad -f_0{\mathcal {V}}_{Schw}'-\frac{1}{2}f_0''' \gtrsim (1-3M/r)^2\ell (\ell +1) +1. \end{aligned}$$

We begin with a lemma concerning the behaviour of the potential ${\mathcal {V}}$ in the ${\mathcal {G}}_1$ frequency range.

##### Lemma 8.2.1

Let $0<c_{\flat }<1$ and $C_\sharp >1$ be arbitrary. For sufficiently small $|a|<a_0\ll M$, then for all frequency triples in the range ${\mathcal {G}}_1$, the potential ${\mathcal {V}}_0$ of (161) has a unique maximum $r_{\mathrm{max}}$ satisfying property 2. and

175 $$\begin{aligned} (r-r_{\mathrm{max}})^{-1}{\mathcal {V}}'_0 \gtrsim \widetilde{\Lambda } \end{aligned}$$

in $[A_1,A_2]$. If $m=0$, then $r_{\mathrm{max}}$ is manifestly independent of $\omega$ and $\widetilde{\Lambda }$.

##### Proof

This is an easy computation in view of (163). For the region ${\mathcal {G}}_1\setminus \{{\widetilde{\Lambda }} +\omega ^2 +m^2 \le C_{\sharp } \}$, one uses the bound

$$\begin{aligned} \widetilde{\Lambda }-4|a\omega | \ge \frac{1}{2}\widetilde{\Lambda } + \frac{1}{4}c_{\flat } \omega ^2 \ge \frac{1}{4} \widetilde{\Lambda }+\frac{1}{4} c_{\flat }\omega ^2 +\frac{1}{16}m^2 \,\, \mathrm{\ in\ } \,\, {\mathcal {G}}_1\setminus \{{\widetilde{\Lambda }} +\omega ^2 +m^2 \le C_{\sharp } \} \end{aligned}$$

and the smallness of *a*. For the region $\{{\widetilde{\Lambda }} +\omega ^2 +m^2 \le C_{\sharp } \}$ it suffices to use the general bound $\widetilde{\Lambda }\ge 1$ and the smallness of *a*. Notice that according to our conventions, the constant in the $\gtrsim$ indeed only depends on *M*, since smallness of *a* can be used to absorb the $c_{\flat }$ and $C_\sharp$ dependence. $\square$

Let $\chi (r^*)$ be a cutoff function such that $\chi =1$ in $[A_1^*/4,A_2^*/4]$ and $\chi =0$ in $[A_1^*/2,A_2^*/2]^c$. We define now

176 $$\begin{aligned} f= \left( 1-\frac{3M +\chi (r^*)({r}_{\mathrm{max}}-3M)}{r}\right) \left( 1+\frac{M}{r}\right) . \end{aligned}$$

This function obviously satisfies property 1. and is easily seen to satisfy property 3.

It remains to show property 4. By (175) and the definition of *f* we have

$$\begin{aligned} -f{\mathcal {V}}_0' \gtrsim {\widetilde{\Lambda }} (1-r_{\mathrm{max}}r^{-1})^2 \, . \end{aligned}$$

On the other hand, for $|a|\ll a_0<M$ sufficiently small, we have that $|f_0'''-f''' |\le c(a)$, and thus

$$\begin{aligned} -f{\mathcal {V}}_0' -\frac{1}{2}f''' \gtrsim ({\widetilde{\Lambda }} (1-r_{\mathrm{max}}r^{-1})^2+1). \end{aligned}$$

Finally, we note that ${\mathcal {V}}={\mathcal {V}}_0+{\mathcal {V}}_1+{\mathcal {V}}_2$, and we have $|{\mathcal {V}}_1-({\mathcal {V}}_{Schw})_1|\le c(a)$, $|{\mathcal {V}}_2|\le c(a)$ with $c(a)\rightarrow 0$.

We have

$$\begin{aligned} -f{\mathcal {V}} '-\frac{1}{2}f''' = -f{\mathcal {V}}_0'-\frac{1}{2}f''' -f({\mathcal {V}}_{Schw})_1' +f({\mathcal {V}}_1'-({\mathcal {V}}_{Schw})_1' ) -f{\mathcal {V}}_2' \end{aligned}$$

It follows readily that property 4. indeed holds for frequencies in ${\mathcal {G}}_1$. $\square$

Now, given a parameter $\delta _1<1$, we define the function

177 $$\begin{aligned} y_1= \delta _1((1-\chi )f+\chi f^3)). \end{aligned}$$

Note that this function satisfies (166). We compute

178 $$\begin{aligned} y'_1 = \delta _1( (1-\chi )f' + 3\chi f^2 f' - \chi ' f +\chi ' f^3) \gtrsim \delta _1 (r-r_{\mathrm{max}})^2 \end{aligned}$$

where we are using also that $|f|\le 1$ implies that $|f^3|\le |f|$.

Note on the other hand that for sufficiently small $|a|<a_0\ll M$, we have

$$\begin{aligned} |{\mathcal {V}} |\lesssim \widetilde{\Lambda }+1 \, ,\qquad |{\mathcal {V}}'| \lesssim \widetilde{\Lambda }+1 \end{aligned}$$

in $r^*\in [A_1^*,A_2^*]$ for all frequencies in ${\mathcal {G}}_1$, in view of the general bound

179 $$\begin{aligned} \frac{1}{4}m^2 + 1 \le \widetilde{\Lambda } \end{aligned}$$

and the bound

$$\begin{aligned} \omega ^2 \le c_{\flat }^{-1} \widetilde{\Lambda } +C_\sharp , \end{aligned}$$

which holds in ${\mathcal {G}}_1$. Thus

$$\begin{aligned} y'_1 {\mathcal {V}} - y_1{\mathcal {V}}' \lesssim \delta _1(\widetilde{\Lambda }( 1-r_{\mathrm{max}}r^{-1})^2+1). \end{aligned}$$

It follows that we may choose $\delta _1$ sufficiently small so as for

180 $$\begin{aligned} -f{\mathcal {V}}'-\frac{1}{2}f''' -y_1'{\mathcal {V}} +y_1{\mathcal {V}}' +y_1'\omega ^2 \gtrsim (\widetilde{\Lambda }+\delta _1 \omega ^2) (1-r_{\mathrm{max}}r^{-1})^2 +1.\qquad \end{aligned}$$

**Henceforth**, $\delta _1$ **will be fixed.** In particular, according to our conventions, we may replace the $\delta _1$ factor by 1 on the right hand side of (180).

In view of (180) and (178), examining the identities of Sect. 8.2.1, we have obtained the degenerate coercivity of $(\mathrm{Q}^f+\mathrm{Q}^{y_1})'$.

We would like to improve this coercivity in the “angular-dominated” subrange of ${\mathcal {G}}_1$. Let us now introduce a new parameter $C_{\flat } \gg 1$ and consider the range

181 $$\begin{aligned} {\mathcal {G}}_1\cap \{ \widetilde{\Lambda } \ge C_{\flat } \omega ^2\}. \end{aligned}$$

Noting that we have

$$\begin{aligned} {\mathcal {V}} \gtrsim \widetilde{\Lambda } +1 \end{aligned}$$

in ${\mathcal {G}}_1$, it follows that for $C_{\flat }$ sufficiently large, we have

$$\begin{aligned} {\mathcal {V}} -\omega ^2 \gtrsim {\mathcal {V}} \gtrsim \widetilde{\Lambda } \gtrsim \widetilde{\Lambda }+\omega ^2 \end{aligned}$$

in (181). **Henceforth**, $C_{\flat }$ **will be fixed**. We may now define a new small parameter $\delta _3>0$ and define a function

$$\begin{aligned} y_2= \delta _3 (r_{\mathrm{max}}-r^*)\chi , \end{aligned}$$

where $\chi$ is the cutoff from above. We have that for frequency triples in (181),

$$\begin{aligned} y_2'(\omega ^2 -{\mathcal {V}})\gtrsim \delta _3, \qquad -y_2{\mathcal {V}}' \lesssim \delta _3 (\widetilde{\Lambda }(1-r_{\mathrm{max}}r^{-1})^2 +1) \end{aligned}$$

in $$[A_1^*/4,A_2^*/4]$$, while

$$\begin{aligned} y_2'{\mathcal {V}}-y_2{\mathcal {V}}' \lesssim \delta _3 (\widetilde{\Lambda }(1-r_{\mathrm{max}}r^{-1})^2 +1), \qquad |y'_2| \lesssim \delta _3 \end{aligned}$$

in $[A_1^*, A_2^*]$. In particular, we may choose $\delta _3$ sufficiently small, with the smallness requirement depending only on *M*, so that, defining

182 $$\begin{aligned} y=y_1 +y_2, \end{aligned}$$

we have

183 $$\begin{aligned}&2f'+y' \gtrsim 1,\qquad -f{\mathcal {V}}'-\frac{1}{2}f''' -y'{\mathcal {V}} +y{\mathcal {V}}' \nonumber \\&\quad +\,\omega ^2 y' \gtrsim (\widetilde{\Lambda }+\omega ^2)(\delta _3+ (1-r_{\mathrm{max}}r^{-1})^2) +1 \end{aligned}$$

in (181). **Henceforth**, $\delta _3$ **will be fixed.**

We are ready now for our final definitions. In the range (181), we define *y* by (182). Since $\delta _3$ is now fixed we may now write

$$\begin{aligned} (\delta _3+(1-r_{\mathrm{max}}r^{-1})^2) \gtrsim 1. \end{aligned}$$

We thus can set $r_{\mathrm{trap}}=0$.

For the remaining frequencies in ${\mathcal {G}}_1$, i.e. for frequencies in ${\mathcal {G}}_1 \cap \{\widetilde{\Lambda } < C_{\flat }\omega ^2\}$, we define simply $y=y_1$ and $r_{\mathrm{trap}} = r_{\mathrm{max}}$.

Finally, we consider the current

$$\begin{aligned} E\mathrm{Q}^T \end{aligned}$$

for *E* the parameter fixed in Sect. 5.1.

Thus, applying the identity corresponding to (172) in view of (178), (180) and (183), we obtain that Proposition 8.2.2 holds for all frequencies in ${\mathcal {G}}_1$.

#### The ${\mathcal {G}}_2$ Range

We define this frequency range to be the complement of ${\mathcal {G}}_1$, i.e.

184 $$\begin{aligned} {\mathcal {G}}_2 =\{\omega ^2> c_{\flat }^{-1} {\widetilde{\Lambda }} \} \cap \{{\widetilde{\Lambda }} +\omega ^2+m^2 > C_\sharp \}. \end{aligned}$$

These are the “time-dominated” large frequencies.

We may choose $c_{\flat }$ sufficiently small, and $C_\sharp$ sufficiently large, so that for sufficiently small $|a|<a_0\ll M$, we have

185 $$\begin{aligned} \omega ^2-{\mathcal {V}} \ge \frac{1}{2}\omega ^2, \qquad |{\mathcal {V}}'| \le \frac{1}{2}\omega ^2\qquad \mathrm{\ in\ }{\mathcal {G}}_2 \end{aligned}$$

**Henceforth**, $c_{\flat }$ **and **$C_\sharp$ **will be fixed by the above restriction.** We note that it is certainly the case that $C_{\flat } \ge c_{\flat }$.

Consider the function $$f_0 $$ of the previous section. We define simply $$f=f_0$$ for frequencies in $${\mathcal {G}}_2$$.

Given the parameter $\delta _1$ fixed in Sect. 8.2.3, we define now $y=\delta _1 f$. It follows from (185) that in the range ${\mathcal {G}}_2$ we have

$$\begin{aligned} (2f' +y')\gtrsim 1, \qquad -( f{\mathcal {V}}' +y{\mathcal {V}}') -\frac{1}{2} f''' +y' (\omega ^2-{\mathcal {V}}) \gtrsim \omega ^2 \gtrsim (\omega ^2 +\widetilde{\Lambda }^2 +1 ). \end{aligned}$$

We may define thus the parameter $r_{\mathrm{trap}}=0$ for the frequency range ${\mathcal {G}}_2$.

Finally, we may again add

$$\begin{aligned} E\mathrm{Q}^T \end{aligned}$$

for *E* the parameter fixed in Sect. 5.1.

Thus again applying the identity to (172) with the above definitions we obtain that Proposition 8.2.2 holds for all frequencies in ${\mathcal {G}}_2$.

Since ${\mathcal {G}}_1\cup {\mathcal {G}}_2$ contains all admissible frequencies, the results of this section together with Section 8.2.3 imply that Proposition 8.2.2, and thus (170), indeed holds.

The proof of Proposition 8.2.1 is now complete. $\square$

**Let us recall that in the course of the above proof, we have fixed the parameter **$\delta _1$. **This allows us to fix also** $\delta _2$ **of Proposition** 5.1.1. **Since** *E* **has been fixed previously, it follows that all dependences on parameters can be removed from the** $\lesssim$ **in the statement of Proposition** 5.1.1.

### Transport Estimates for $\psi ^{[\pm 2]}$ and $u^{[\pm 2]}$

In this section we will prove frequency-localised versions for the transport estimates of [31] to obtain estimates for $u^{[+2]}$ and $\psi ^{[+2]}$ from $\Psi ^{[+2]}$ as well as for $u^{[-2]}$ and $\psi ^{[-2]}$ from $\Psi ^{[-2]}$, localised in $r\in [A_1,A_2]$.

The main result of the section is:

#### Proposition 8.3.1

With the assumptions of Theorem 8.1, we have the following estimates:

186 $$\begin{aligned}&\Vert {\mathfrak {d}}\psi ^{[\pm 2]}\Vert ^2(A_{ \mp }) + \Vert {\mathfrak {d}}u^{[\pm 2]}\Vert ^2(A_{ \mp })+ \Vert {\mathfrak {d}}\psi ^{[\pm 2]}\Vert ^2 +\Vert {\mathfrak {d}}u^{[\pm 2]}\Vert ^2 \nonumber \\&\quad \lesssim \Vert {\mathfrak {d}} \Psi ^{[\pm 2]}\Vert ^2 + \Vert {\mathfrak {d}}\psi ^{[\pm 2]}\Vert ^2(A_{ \pm }) + \Vert {\mathfrak {d}}u^{[\pm 2]}\Vert ^2(A_{ \mp }). \end{aligned}$$

#### Proof

We consider first the case $+2$ of (186).

Adding the identity arising from multiplying (157) by $r \sqrt{\Delta } \ \overline{\psi ^{[+2]}}$ and its complex conjugate by $r \sqrt{\Delta }\psi ^{[+2]}$ leads after integration and applying Cauchy–Schwarz on the right hand side to the estimate

187 $$\begin{aligned} r |\sqrt{\Delta } \psi ^{[+2]}|^2 \left( A_1^*\right) + \int _{A_1^*}^{A_2^*} dr^* |\sqrt{\Delta } \psi ^{[+2]}|^2 \lesssim \int _{A_1^*}^{A_2^*}dr^* |\Psi ^{[+2]}|^2 +r |\sqrt{\Delta } \psi ^{[+2]}|^2 \left( A_2^*\right) \, . \end{aligned}$$

Similarly, adding the identity arising from multiplying (156) by $r\overline{u^{[+2]}}w$ and its complex conjugate by $r u^{[+2]}w$ leads after integration and applying Cauchy–Schwarz on the right hand side to the estimate

188 $$\begin{aligned} r |u^{[+2]}w|^2 \left( A_1^*\right) + \int _{A_1^*}^{A_2^*} dr^* \ |u^{[+2]}w|^2 \lesssim \int _{A_1^*}^{A_2^*} dr^* | \psi ^{[+2]}|^2 +r |u^{[+2]}w|^2 \left( A_2^*\right) \, . \end{aligned}$$

Combining (187) and (188) yields (186) without the $m^2$ and $\omega ^2$ terms in the norms on the left.

To obtain the estimate with the $m^2$ and $\omega ^2$ terms we define the frequency ranges

$$\begin{aligned} {{{\mathcal {F}}}}^{\sharp }=\left\{ \omega ^2\ge \frac{1}{4}C_{\flat }^{-1} m^2 \right\} ,\qquad {{{\mathcal {F}}}}^{\flat }=\left\{ \omega ^2<\frac{1}{4}C_{\flat }^{-1} m^2\right\} \end{aligned}$$

where $C_{\flat }$ is the constant of Sect. 8.2.3. In view of the general bound (179) which holds for all admissible frequencies, it follows that in the frequency range ${{{\mathcal {F}}}}^{\flat }$, we have

$$\begin{aligned} C_{\flat } \omega ^2 < \frac{1}{4} m^2 \le \widetilde{\Lambda } \end{aligned}$$

and thus ${{{\mathcal {F}}}}^{\flat }$ is contained in the frequency range (181). It follows that $r_{\mathrm{trap}}=0$ for ${{{\mathcal {F}}}}^{\flat }$, i.e. these frequencies are not “trapped”.

Suppose first that $(\omega ,m)$ lie in the frequency range ${{{\mathcal {F}}}}^{\flat }$. Since $r_{\mathrm{trap}}=0$, we have

189 $$\begin{aligned} \int _{A_1^*}^{A_2^*}\left[ |(\Psi ^{[\pm 2]})'|^2+ ( \widetilde{\Lambda }^2+m^2+\omega ^2+1)|\Psi ^{[\pm 2]}|^2\right] dr^* \lesssim \Vert {\mathfrak {d}}\Psi ^{[\pm 2]} \Vert ^2. \end{aligned}$$

Multiplying thus (157) and (156) by *m* and $\omega$ and repeating the argument leading to (187) and (188) immediately leads to (186).

Suppose on the other hand that $(\omega , m)$ lie in the frequency range ${{{\mathcal {F}}}}^{\sharp }$. Here we do not have the $m^2$ and $\omega ^2$ in (189) and thus we proceed as follows. Commuting (157) by $\frac{d}{dr^*}$ leads to the identity

190 $$\begin{aligned}&\left( \frac{d}{dr^*} - i \omega + \frac{iam}{r^2+a^2} \right) \left( \sqrt{\Delta } \psi ^{[+2]}\right) ^\prime = - w \left( \Psi ^{[+2]}\right) ^\prime - w^\prime \Psi ^{[+2]} \nonumber \\&\qquad \quad + 2r\frac{iam}{r^2+a^2} w \cdot \sqrt{\Delta }\psi ^{[+2]} \, . \end{aligned}$$

Multiplying this by $r \left( \sqrt{\Delta } \overline{\psi ^{[+2]}}\right) ^\prime$ and adding the complex conjugate multiplied by $r \left( \sqrt{\Delta } {\psi ^{[+2]}}\right) ^\prime$ we find, upon integration and using Cauchy–Schwarz on the right hand side, the estimate

191 $$\begin{aligned}&r \Big |\left( \sqrt{\Delta } \psi ^{[+2]}\right) ^\prime \Big |^2 \left( A_1^*\right) + \int _{A_1^*}^{A_2^*} dr^* \ \Big |\left( \sqrt{\Delta } \psi ^{[+2]}\right) ^\prime \Big |^2\nonumber \\&\quad \lesssim r \Big |\left( \sqrt{\Delta } \psi ^{[+2]}\right) ^\prime \Big |^2 \left( A_2^*\right) + \Vert {\mathfrak {d}} \Psi ^{[\pm 2]}\Vert ^2 \nonumber \\&\qquad + \int _{A_1^*}^{A_2^*} dr^* a^2 m^2 |\sqrt{\Delta } \psi ^{[+2]}|^2 \, . \end{aligned}$$

Using the pointwise relation (157) and the definition of the norm $\Vert {\mathfrak {d}} \Psi ^{[\pm 2]}\Vert$ (as well as the simple fact that for $i=1,2$ $| \Psi ^{\pm 2}|^2 \left( A_i^*\right) \lesssim \Vert {\mathfrak {d}} \Psi ^{[\pm 2]}\Vert ^2$), the estimate (191) is also valid replacing on the left hand side $\Big |\left( \sqrt{\Delta } \psi ^{[+2]}\right) ^\prime \Big |^2$ by $\Big |{\underline{L}} \left( \sqrt{\Delta } \psi ^{[+2]}\right) \Big |^2=|w\Psi ^{[+2]}|^2$. Using the relation (155) we therefore deduce

192 $$\begin{aligned}&\left( \omega - \frac{am}{r^2+a^2} \right) ^2 \Big | \sqrt{\Delta } \psi ^{[+2]}\Big |^2 \left( A_1^*\right) + \int _{A_1^*}^{A_2^*} dr^* \left( \omega - \frac{am}{r^2+a^2} \right) ^2 \Big | \sqrt{\Delta } \psi ^{[+2]}\Big |^2 \nonumber \\&\quad \lesssim \Vert {\mathfrak {d}} \Psi ^{[\pm 2]}\Vert ^2 + \int _{A_1^*}^{A_2^*} dr^* a^2m^2 |\sqrt{\Delta } \psi ^{[+2]}|^2 + \left( \omega - \frac{am}{r^2+a^2} \right) ^2 \Big | \sqrt{\Delta } \psi ^{[+2]}\Big |^2 \left( A_2^*\right) \, . \end{aligned}$$

In the range ${{{\mathcal {F}}}}^{\sharp }$, restricting to sufficiently small $|a|<a_0\ll M$, we have that

$$\begin{aligned} \omega ^2 \lesssim \left( \omega - \frac{am}{r^2+a^2} \right) ^2 \lesssim \omega ^2. \end{aligned}$$

It follows that in the inequality (192), we can replace the factor in round bracket on the left hand side simply by $\omega ^2$ and absorb the second term on the right by the left hand side. This establishes (186) for the $\psi ^{[+2]}$-norm on the left. We can now multiply (188) by $m^2$ and $\omega ^2$ and use the estimate just obtained for $\psi ^{[+2]}$ to establish the estimate (186) also for the $u^{[+2]}w$-term. The proof of (186) is now complete.

To prove (186) for $s=-2$ one follows the identical argument but choosing the multiplier $\frac{1}{r}$ instead of *r*. $\square$

### Controlling the Inhomogeneous Term ${\mathcal {K}}^{[\pm 2]}$ in Proposition 8.2.1

#### Proposition 8.4.1

The term

$$\begin{aligned} {\mathcal {K}}^{[\pm 2]}=\int _{A_1^*}^{A_2^*} {{\mathcal {J}}}^{[\pm 2]} \cdot (f, y, E) \cdot (\Psi ^{[\pm 2]}, {\Psi ^{[\pm 2]}}')\, dr^* \end{aligned}$$

appearing in Proposition 8.2.1 satisfies

193 $$\begin{aligned}&\big |{\mathcal {K}}^{[\pm 2]}\big | \lesssim |a|\Vert {\mathfrak {d}} \Psi ^{[\pm 2]}\Vert ^2 + |a|\Vert {\mathfrak {d}}\psi ^{[\pm 2]}\Vert ^2 + |a|\Vert {\mathfrak {d}}u^{[\pm 2]}\Vert ^2 \nonumber \\&\quad + |a| \sum _{i=1}^2 ( \Vert {\mathfrak {d}}\psi ^{[\pm 2]}\Vert ^2(A_i) + \Vert {\mathfrak {d}}u^{[\pm 2]}\Vert ^2(A_{i})). \end{aligned}$$

#### Proof

Since *f*, $f^\prime$ and *y* are all uniformly bounded we have by Cauchy–Schwarz:

194 $$\begin{aligned}&\int _{A_1^*}^{A_2^*} \big |f\text {Re}\left( {\Psi ^{[\pm 2]}}'\overline{{{\mathcal {J}}}^{[\pm 2]} }\right) \big | + \big |f'\text {Re}\left( \Psi ^{[\pm 2]}\overline{{{\mathcal {J}}}^{[\pm 2]} }\right) \big | + \big | y\text {Re}\left( {\Psi ^{[\pm 2]}}'\overline{{{\mathcal {J}}}^{[\pm 2]} }\right) \big | \nonumber \\&\quad \lesssim |a|\Vert {\mathfrak {d}} \Psi ^{[\pm 2]}\Vert ^2 + |a|\Vert {\mathfrak {d}}\psi ^{[\pm 2]}\Vert ^2 + |a|\Vert {\mathfrak {d}}u^{[\pm 2]}\Vert ^2 \, . \end{aligned}$$

For the last remaining term, $\int _{A_1^*}^{A_2^*} \omega \text {Im}\left( {{\mathcal {J}}}^{[\pm 2]} \overline{\Psi ^{[\pm 2]}}\right)$, we observe that we only need to estimate

195 $$\begin{aligned} \Big | \int _{A_1^*}^{A_2^*} {\mathfrak {c}}\left( r\right) \text {Im}\left( im\psi ^{[\pm 2]} \omega \overline{\Psi ^{[\pm 2]}}\right) \Big | \ \ \ \ \text {and} \ \ \ \ \Big | \int _{A_1^*}^{A_2^*} {\mathfrak {c}}\left( r\right) \text {Im}\left( imu^{[\pm 2]} \omega \overline{\Psi ^{[\pm 2]}}\right) \Big | \, , \end{aligned}$$

where ${\mathfrak {c}}\left( r\right)$ denotes a generic bounded real-valued function with uniformly bounded derivative in $\left[ A^*_1,A^*_2\right]$ (whose explicit form may change in the estimates below). This is because the other terms appearing in ${\mathcal {J}}^{[\pm 2]}$ are again easily controlled via Cauchy–Schwarz and satisfy the estimate (194). We show how to estimate these terms for $s=+\,2$, the case $s=-\,2$ being completely analogous.

For the first term of (195) we have

196 $$\begin{aligned}&\int _{A_1^*}^{A_2^*} {\mathfrak {c}}\left( r\right) \text {Im}\left( im\psi ^{[+ 2]} \omega \overline{\Psi ^{[+2]}}\right) = \int _{A_1^*}^{A_2^*} {\mathfrak {c}}\left( r\right) \nonumber \\&\qquad \times \text {Im}\left( m \overline{\Psi ^{[+2]}} \left( - {\underline{L}} \psi ^{[+2]} - \left( \psi ^{[+2]}\right) ^\prime + \frac{iam}{r^2+a^2} \psi ^{[+2]} \right) \right) \nonumber \\&\quad = \int _{A_1^*}^{A_2^*} {\mathfrak {c}}\left( r\right) \text {Im} \left( m\overline{\Psi ^{[+2]}} \psi ^{[+2]} \right) + {\mathfrak {c}}\left( r\right) \text {Im} \left( \overline{\Psi ^{[+2]}} m \psi ^{[+2]} \right) \Big |^{A_2^*}_{A_1^*}\nonumber \\&\qquad + \int _{A_1^*}^{A_2^*} {\mathfrak {c}}\left( r\right) \text {Im} \left( \overline{\Psi ^{[+2]}}^\prime m \psi ^{[+2]} \right) \nonumber \\&\qquad + \int _{A_1^*}^{A_2^*}\text {Im} \left( \left( - {\mathfrak {c}}\left( r\right) m\overline{\psi ^{[+2]}}^\prime + {\mathfrak {c}}\left( r\right) m\overline{\psi ^{[+2]}}\right) i am \psi ^{[+2]}\right) \end{aligned}$$

where we have used the (frequency localised) relation between ${\Psi ^{[+2]}}$ and ${\psi }^{[+2]}$ twice. Now the first three terms on the right hand side are again easily controlled using Cauchy–Schwarz (as well as the simple fact that for $i=1,2$ $| \Psi ^{[\pm 2]}|^2 \left( A_i^*\right) \lesssim \Vert {\mathfrak {d}} \Psi ^{[\pm 2]}\Vert ^2$). For the term in the last line we integrate the first summand by parts while the second is already manifestly controlled by $\Vert {\mathfrak {d}}\psi ^{[\pm 2]}\Vert ^2$. This leads immediately to (193).

For the second term of (195), write

197 $$\begin{aligned} \int _{A_1^*}^{A_2^*} {\mathfrak {c}}\left( r\right) \text {Im}\left( im u^{[+ 2]} \omega \overline{\Psi ^{[+2]}}\right) =\int _{A_1^*}^{A_2^*} \text {Re}\left( m\overline{u^{[+ 2]}} \omega \left( {\mathfrak {c}}\left( r\right) {\underline{L}} \psi ^{[+2]} + {\mathfrak {c}}\left( r\right) \psi ^{[+2]}\right) \right) \, . \end{aligned}$$

The second term on the right is already manifestly controlled by $\Vert {\mathfrak {d}}\psi ^{[\pm 2]}\Vert ^2$ and for the first we integrate by parts

198 $$\begin{aligned}&-\int _{A_1^*}^{A_2^*} \text {Re}\left( m\overline{u^{[+ 2]}} \omega \left( {\mathfrak {c}}\left( r\right) {\underline{L}} \psi ^{[+2]} \right) \right) = \text {Re} \left( m \overline{u^{[+ 2]}} \omega {\mathfrak {c}}\left( r\right) \psi ^{[+2]}\right) \Big |_{A_1^*}^{A_2^*} \nonumber \\&\quad + \int _{A_1^*}^{A_2^*} {\mathfrak {c}}\left( r\right) m\omega |\psi ^{[+2]}|^2 + {\mathfrak {c}}\left( r\right) \text {Re} \left( m \omega \overline{u^{[+ 2]}} \psi ^{[+2]}\right) \end{aligned}$$

from which the estimate (193) is easily obtained. $\square$

Putting together Propositions 8.2.1, 8.3.1 and 8.4.1 , we obtain Theorem 8.1.

## Back to Physical Space: Energy Boundedness and Integrated Local Energy Decay

We now turn in this section in ernest to the study of the Cauchy problem for (37) for $s=\pm \,2$. The main result of this section will be a uniform (degenerate) energy boundedness and integrated energy decay statement. This will be stated as Theorem 9.1 of Sect. 9.1. This corresponds to statement 1. of the main result of the paper, Theorem 4.1.

The remainder of the section will then be devoted to the proof of Theorem 9.1. We first define in Sect. 9.2 a cutoff version  of our solution $\upalpha ^{[\pm 2]}$ of (37) such that  satisfies an inhomogeneous equation (53), whose inhomogeneous term  is localised in time to be supported only “near” ${\tilde{t}}^*=0$ and “near” ${\tilde{t}}^*=\tau _{\mathrm{final}}$ and in space to be supported only in $r^*=[2A_1^*,2A_2^*]$. The cutoff is such that restricted to $r\in [A_1,A_2]$,  is compactly supported in ${\tilde{t}}^*\in [0,\tau _{\mathrm{final}}]$. This allows us in Sect. 9.3 to then apply the results of Sect. 8 to such , summing the resulting estimate over frequencies. In Sect. 9.4 we shall combine this estimate with the conditional estimates of Sect. 5, using also the auxiliary estimates of Sect. 5.3 to obtain a global integrated energy decay statement, with an error term, however, on the right side arising from the cutoff. Finally, we shall bound this latter error terms associated to the cutoff in Sect. 9.5, again using the auxiliary estimates of Sect. 5.3, allowing us to infer the statement of Theorem 9.1.

As remarked in Sect. 1.2.5, in the axisymmetric case, one can directly distill from the calculations of this paper an alternative, simpler proof of Theorem 9.1 expressed entirely in physical space. We do this in Sect. 9.6.

### Statement of Degenerate Boundedness and Integrated Energy Decay

#### Theorem 9.1

Let $\upalpha ^{[\pm 2]}$, $\Psi ^{[\pm 2]}$ and $\uppsi ^{[\pm 2]}$ be as in Theorem 4.1.

Then, for , we have the following estimates

- the basic degenerate Morawetz estimate
  199 $$\begin{aligned}&{{\mathbb {I}}}^{\mathrm{deg}}_{\eta } \left[ \Psi ^{[+2]}\right] \left( 0, \tau _{\mathrm{final}}\right) +{\mathbb {I}}_{\eta } \left[ \uppsi ^{[+2]}\right] \left( 0, \tau _{\mathrm{final}}\right) +{\mathbb {I}}_{\eta } \left[ \upalpha ^{[+2]}\right] \left( 0, \tau _{\mathrm{final}}\right) \nonumber \\&\quad \lesssim \ \ {{\mathbb {E}}}_{{\widetilde{\Sigma }}_{\tau },\eta } \left[ \Psi ^{[+2]}\right] \left( 0\right) +{\mathbb {E}}_{{\widetilde{\Sigma }}_{\tau },\eta } \left[ \uppsi ^{[+2]}\right] \left( 0\right) + {\mathbb {E}}_{{\widetilde{\Sigma }}_{\tau },\eta } \left[ \upalpha ^{[+2]}\right] \left( 0\right) \end{aligned}$$
- the $\eta$-weighted energy boundedness estimate
  200 $$\begin{aligned}&{{\mathbb {E}}}_{{\mathcal {H}}^+} \left[ \Psi ^{[+2]}\right] \left( 0, \tau _{\mathrm{final}}\right) + {{\mathbb {E}}}_{{\widetilde{\Sigma }}_{\tau },\eta } \left[ \Psi ^{[+2]}\right] \left( \tau _{\mathrm{final}}\right) \nonumber \\&\quad \lesssim \ \ {{\mathbb {E}}}_{{\widetilde{\Sigma }}_{\tau },\eta } \left[ \Psi ^{[+2]}\right] \left( 0\right) +{\mathbb {E}}_{{\widetilde{\Sigma }}_{\tau },\eta } \left[ \uppsi ^{[+2]}\right] \left( 0\right) + {\mathbb {E}}_{{\widetilde{\Sigma }}_{\tau },\eta } \left[ \upalpha ^{[+2]}\right] \left( 0\right) \, . \end{aligned}$$

Similarly, for , we have

- the basic degenerate Morawetz estimate
  201 $$\begin{aligned}&\ {{\mathbb {I}}}^{\mathrm{deg}}_{\eta } \left[ \Psi ^{[-2]}\right] \left( 0, \tau _{\mathrm{final}}\right) +{\mathbb {I}} \left[ \uppsi ^{[-2]}\right] \left( 0, \tau _{\mathrm{final}}\right) +{\mathbb {I}}\left[ \upalpha ^{[-2]}\right] \left( 0,\tau _{\mathrm{final}}\right) \nonumber \\&\quad \lesssim \ \ {{\mathbb {E}}}_{{\widetilde{\Sigma }}_{\tau },\eta } \left[ \Psi ^{[-2]}\right] \left( 0\right) +{\mathbb {E}}_{{\widetilde{\Sigma }}_{\tau }} \left[ \uppsi ^{[-2]}\right] \left( 0\right) + {\mathbb {E}}_{{\widetilde{\Sigma }}_{\tau }} \left[ \upalpha ^{[-2]}\right] \left( 0\right) \end{aligned}$$
- the $\eta$-weighted energy boundedness estimate
  202 $$\begin{aligned}&{{\mathbb {E}}}_{{\mathcal {H}}^+} \left[ \Psi ^{[-2]}\right] \left( 0, \tau _{\mathrm{final}}\right) + {{\mathbb {E}}}_{{\widetilde{\Sigma }}_{\tau },\eta } \left[ \Psi ^{[-2]}\right] \left( \tau _{\mathrm{final}}\right) \nonumber \\&\quad \lesssim \ \ {{\mathbb {E}}}_{{\widetilde{\Sigma }}_{\tau },\eta } \left[ \Psi ^{[-2]}\right] \left( 0\right) +{\mathbb {E}}_{{\widetilde{\Sigma }}_{\tau }} \left[ \uppsi ^{[-2]}\right] \left( 0\right) + {\mathbb {E}}_{{\widetilde{\Sigma }}_{\tau }} \left[ \upalpha ^{[-2]}\right] \left( 0\right) \, . \end{aligned}$$

#### Remark 9.1

In the case $s=-2$ one can prove these estimates using only the ${\mathbb {E}}_{{\widetilde{\Sigma }}_{\tau },0}\left[ \Psi ^{[-2]}\right]$-energy. However, that energy is insufficient to eventually control the energy flux of $r^{-3} \alpha ^{[-2]}$ through null infinity, which is why we kept the estimate as symmetric with the $s=+2$-case as possible. See also Remark 5.2.

In the proof of the theorem, we may assume for convenience that the data $(\tilde{\upalpha }^{[\pm 2]}_0,\tilde{\upalpha }^{[\pm 2]}_1)$ are smooth. It follows that all associated appropriately rescaled quantities $\Psi ^{[\pm 2]}$, etc., are smooth in ${\mathcal {R}}_0$. To ease notation we define the data quantities

203 $$\begin{aligned} {\mathbb {D}}^{[+ 2]} \left( 0\right)&= {{\mathbb {E}}}_{{\widetilde{\Sigma }}_{\tau },\eta } \left[ \Psi ^{[+2]}\right] \left( 0\right) +{\mathbb {E}}_{{\widetilde{\Sigma }}_{\tau },\eta } \left[ \uppsi ^{[+2]}\right] \left( 0\right) + {\mathbb {E}}_{{\widetilde{\Sigma }}_{\tau },\eta } \left[ \upalpha ^{[+2]}\right] \left( 0\right) \, ,\nonumber \\ {\mathbb {D}}^{[- 2]} \left( 0\right)&= {{\mathbb {E}}}_{{\widetilde{\Sigma }}_{\tau },\eta } \left[ \Psi ^{[+2]}\right] \left( 0\right) +{\mathbb {E}}_{{\widetilde{\Sigma }}_{\tau }} \left[ \uppsi ^{[+2]}\right] \left( 0\right) + {\mathbb {E}}_{{\widetilde{\Sigma }}_{\tau }} \left[ \upalpha ^{[+2]}\right] \left( 0\right) \, . \end{aligned}$$

### The Past and Future Cutoffs

Let $\varepsilon >0$ be a parameter to be determined. Fix $\tau _{\mathrm{final}}>0$. One easily sees that one can choose a smooth function $\Xi :{\mathbb {R}}\times {\mathbb {R}}\rightarrow [0,1]$ with the properties:

204 $$\begin{aligned} {\left\{ \begin{array}{ll} \Xi =0 &{}\text { if } (r^*,{\tilde{t}}^*)\in [A_1^*,A_2^*]\times \{(-\infty ,0]\cup [\tau _{\mathrm{final}},\infty )\}\\ \Xi =1 &{}\text { if } (r^*,{\tilde{t}}^*)\in \{(-\infty , 2A_1^*]\cup [2A_2^*,\infty )\} \times {\mathbb {R}} \cup [2A_1^*,2A_2^*]\times [\varepsilon ^{-1},\tau _{\mathrm{final}}-\varepsilon ^{-1}]\\ \partial _{r^*}\Xi =0 &{}\text { if } (r^*,{\tilde{t}}^*)\in [A_1^*,A_2^*]\times (-\infty ,\infty )\\ |\partial _{{\tilde{t}}^*}^{k_1}\Xi | \lesssim \varepsilon &{}\text { if } (r^*,{\tilde{t}}^*)\in [A_1^*,A_2^*]\times \{[0,\varepsilon ^{-1}]\cup [\tau _{\mathrm{final}}-\varepsilon ^{-1},\tau _{\mathrm{final}}]\}\\ |\partial _{{\tilde{t}}^*}^{k_1} \partial _{{\tilde{r}}^*}^{k_2} \Xi |\lesssim 1 &{}\text { for all } (r^*,{\tilde{t}}^*)\in {\mathbb {R}}\times {\mathbb {R}}\ \end{array}\right. } \end{aligned}$$

for all $$k_1, k_2\ge 0$$.

Define now205We note that  and satisfies (53) with inhomogeneity given by206We define now  to be given by (38),  to be given by (46)–(47),  to be given by (48) and  to be given by (49)–(50), where all quantities now have .

**Fig. 3:**
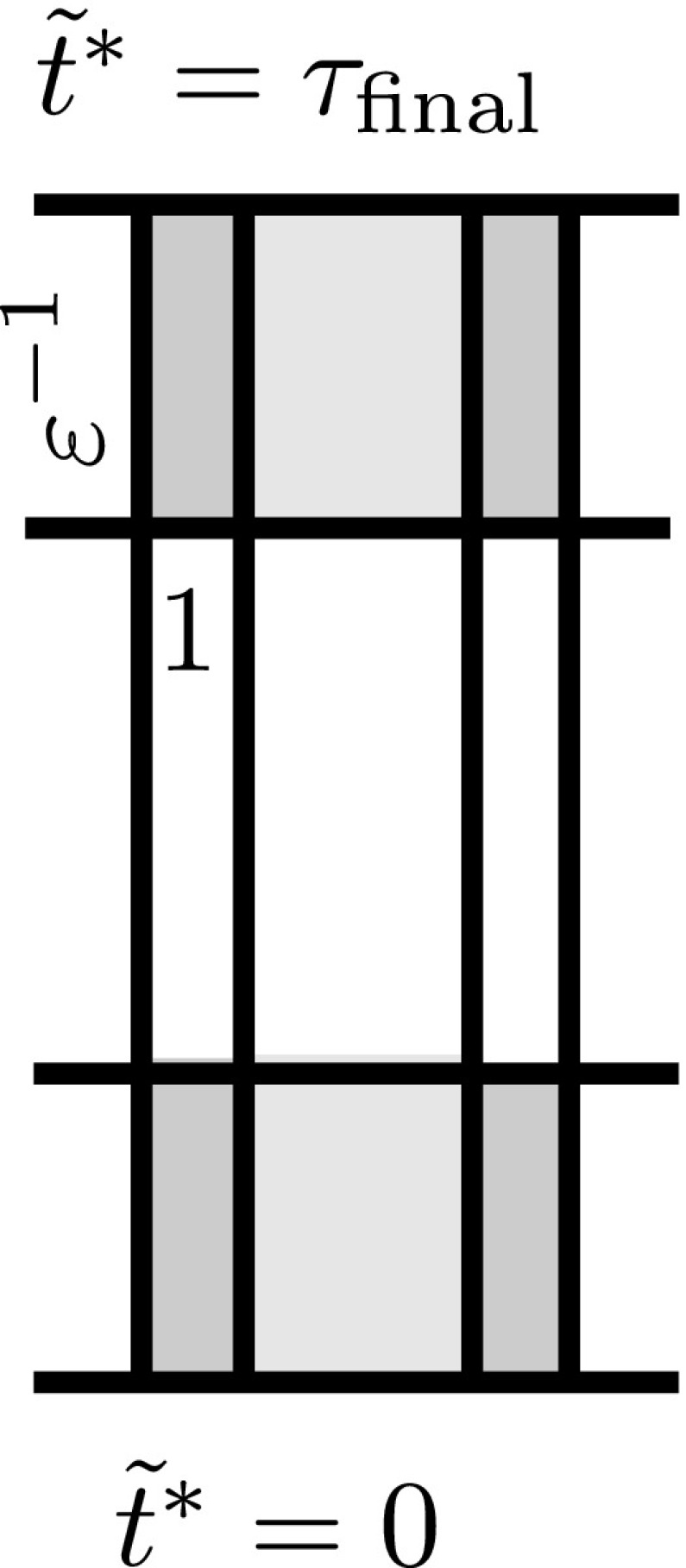
Support of $\nabla \Xi$ restricted to $0\le {\tilde{t}}^*\le \tau _{\mathrm{final}}$

We note that  restricted to $0\le {\tilde{t}}^*\le \tau _{\mathrm{final}}$ is supported in the support of $\nabla \Xi$ (see the shaded regions of Fig. 3):

207 $$\begin{aligned} \big (\{0\le {\tilde{t}}^* \le \varepsilon ^{-1} \}\cup \{\tau _{\mathrm{final}}-\varepsilon ^{-1} \le {\tilde{t}}^*\le \tau _{\mathrm{final}}\}\big ) \bigcap \{2A_1^* \le r^*\le 2A_2^*\} \end{aligned}$$

whilein $$\{A_1\le r\le A_2\}\cap ( \{{\tilde{t}}^*\le 0\}\cup \{{\tilde{t}}^*\ge \tau _{\mathrm{final}}\}$$.

Let us already note the following proposition

#### Proposition 9.2.1

Let  be as above and let ${\mathfrak {G}}^{\pm 2}$ be the inhomogeneous term associated to the generalised Regge–Wheeler equation (54) arising from  according to (56) and (58). Then we have the estimates

208 $$\begin{aligned}&\int _{\widetilde{{\mathcal {R}}}^{\mathrm{trap}}(0,\tau _{\mathrm{final}})} |{\mathfrak {G}}^{[\pm 2]}|^2 dVol \lesssim \varepsilon ^2 \left( {\mathbb {I}}^{\mathrm{trap}}[\upalpha ^{[\pm 2]}](0,\varepsilon ^{-1}) +{\mathbb {I}}^{\mathrm{trap}}[\uppsi ^{[\pm 2]}](0,\varepsilon ^{-1})\right) \nonumber \\&\qquad + \varepsilon ^2\left( {\mathbb {I}}^{\mathrm{trap}}[\upalpha ^{[\pm 2]}](\tau _{\mathrm{final}}-\varepsilon ^{-1},\tau _{\mathrm{final}}) +{\mathbb {I}}^{\mathrm{trap}}[\uppsi ^{[\pm 2]}](\tau _{\mathrm{final}}-\varepsilon ^{-1},\tau _{\mathrm{final}})\right) \nonumber \\&\qquad +\varepsilon \sup _ {0\le \tau \le \tau _{\mathrm{final}}}{\mathbb {E}}_{\widetilde{\Sigma }_\tau ,0} [\Psi ^{[\pm 2]}], \end{aligned}$$

209 $$\begin{aligned}&\int _{\widetilde{{\mathcal {R}}}^{\mathrm{away}}(0,\tau _{\mathrm{final}})} |{\mathfrak {G}}^{[\pm 2]}|^2 dVol \lesssim {\mathbb {I}}_{[\eta ]}[\upalpha ^{[\pm 2]}](0,\varepsilon ^{-1}) +{\mathbb {I}}_{[\eta ]}[\uppsi ^{[ \pm 2]}](0,\varepsilon ^{-1})\nonumber \\&\qquad + {\mathbb {I}}_{[\eta ]}[\upalpha ^{[\pm 2]}](\tau _{\mathrm{final}}-\varepsilon ^{-1},\tau _{\mathrm{final}}) +{\mathbb {I}}_{[\eta ]}[\uppsi ^{[ \pm 2]}](\tau _{\mathrm{final}}-\varepsilon ^{-1},\tau _{\mathrm{final}})\nonumber \\&\qquad +\varepsilon ^{-1}\sup _ {0\le \tau \le \tau _{\mathrm{final}}} {\mathbb {E}}_{\widetilde{\Sigma }_\tau ,0} [\Psi ^{[\pm 2]}]. \end{aligned}$$

Here the subindex $\left[ \eta \right]$ is equal to $\eta$ in case of $s=+2$ and it is dropped entirely in case $s=-2$.

#### Remark 9.2

As the proof shows and is already clear from the support of the cut-offs, only the spacetime integrals in the overlap region are needed on the right hand side of (209).

#### Proof

We first prove (208). Note that the support of ${\mathfrak {G}}$ is manifestly contained in the support (207) of . Moreover, one easily sees that one obtains sum of terms containing

$$\begin{aligned}&L\Psi ^{[\pm 2]}, {\underline{L}}\Psi ^{[\pm 2]}, \Psi ^{[\pm 2]}, T\Psi ^{[\pm 2]}, \Phi \Psi ^{[\pm 2]}, L\uppsi ^{[\pm 2]}, {\underline{L}}\uppsi ^{[\pm 2]}, T\uppsi ^{[\pm 2]}, \Phi \uppsi ^{[\pm 2]}, \uppsi ^{[\pm 2]},\\&L\upalpha ^{[\pm 2]}, {\underline{L}}\upalpha ^{[\pm 2]}, T\upalpha ^{[\pm 2]}, \Phi \upalpha ^{[\pm 2]}, \upalpha ^{[\pm 2]} \end{aligned}$$

with *r* and horizon weights which are uniformly bounded in view of the support. From the conditions (204) defining $\Xi$, it follows that

210 $$\begin{aligned} |L^{k_1}{\underline{L}}^{k_2}T^{k_3}\Xi | \lesssim \varepsilon \qquad \mathrm{for\ }r\in [A_1,A_2] \end{aligned}$$

for any $k_1+k_2+k_3\ge 1$, where we have used also that $t=t^*={\tilde{t}}^*$ in this region by our choices in Sect. 2.1.3. It follows that all terms in the expression for ${\mathfrak {G}}$ pick up an $\varepsilon$ factor. The inequality (208) now follows from Cauchy–Schwarz, the definition of the norms and Remarks 4.1 and 4.2 , where in addition we have appealed to the coarea formula and size of ${\tilde{t}}^*$-support for the term involving $\Psi ^{[\pm 2]}$.

The proof is the same for (209), except that the nontrivial *r* dependence of $\Xi$ given by (204) means that $\varepsilon$ on the right hand side of (210) must now be replaced by 1 outside of $r\in [A_1,A_2]$, and thus the $\varepsilon ^2$ factor of (208) is no longer present in the right hand side of the final estimate. $\square$

We will in fact not use the bound (209) directly, but similar bounds for physical space terms that arise from multiplying ${\mathfrak {G}}\Psi$ and ${\mathfrak {G}}\partial _r\Psi$.

### The Summed Relation

In view of the support of  and the smoothness of (206), it follows that  manifestly satisfies the $[A_1,A_2]$-admissibility condition of Definition 6.1. In a slight abuse of notation, we will denote the coefficients of , , etc., without the  subscript.^12^

We define thus the coefficients $u^{[\pm 2], (a\omega )}_{m\ell }$ and we apply Theorem 8.1 with the admissible frequency triple $(\omega , m, \widetilde{\Lambda }^{[\pm 2], (a\omega )}_{m\ell })$. We now integrate over $\omega$ and sum over frequencies:

$$\begin{aligned} \int _{-\infty }^\infty d\omega \sum _{m\ell } . \end{aligned}$$

From summing the relations (167)–(169), we hence obtain in view of the Plancherel relations of Sect. 6.2.3 (applied to ,  and ):

#### Proposition 9.3.1

Let the assumptions of Theorem 9.1 hold. Define the cut-off quantities ,  and  as in (205), (38) and (46)–(50). Then we have the estimates211
212where


### Global Physical Space Estimates

Let us first combine the above estimates with the conditional physical space estimates proven in Sect. 5.

#### Proposition 9.4.1

Let the assumptions of Theorem 9.1 hold. Define the cut-off quantities ,  and  as in (205), (38) and (46)–(50). Then we have the estimates213
214whereIn the above, the subindex $\left[ \eta \right]$ is equal to $\eta$ in case of $s=+\,2$ and it is dropped entirely in case $s=-\,2$.

#### Proof

We add the estimates of Proposition 9.3.1 with those of Sect. 5 as follows.

Let us consider first the $+2$ case. We first add the first estimate (105) of Proposition 5.2.1 (applied to  and  with $\tau _1=0$, $\tau _2=\tau _{\mathrm{final}}$ and with $p=\eta$) to a suitable constant times the estimate (212) of Proposition 9.3.1. The constant ensures that the terms ${\mathbb {E}}_{r=A_2}$ on the left hand side of (105) is sufficient to absorb the analogous term on the right hand side of (212). Finally, we now add to the previous combination a suitable constant times the second estimate (106) of Proposition 5.2.1, again so that the boundary terms on ${\mathbb {E}}_{r=A_1}$ are now absorbed. We obtain thus215
216in the case of $+2$. For the $-2$ case, we choose the relative constants in the reverse order, starting with the second estimate (115) of Proposition 5.2.2. We obtain again (215) in the $-2$ case, as well as the estimate (216) for the boundary terms.

We now similarly add Proposition 5.1.1 (applied to  with $\tau _1=0$, $\tau _2=\tau _{\mathrm{final}}$) to (211), noting that the ${\mathbb {Q}}$ boundary terms exactly cancel. This gives thus217We fix now a sufficiently small parameter *e* depending only on *M*. It follows that, restricting to $a_0\ll e$, we may sum $e\times$ (215) with (217) to absorb both the first term on the right hand side of (215) and the middle two terms on the right hand side of (217). The desired (213) follows.

The estimate (214) again follows from (216) and (213). $\square$

In the rest of this subsection, we proceed to remove the  from the quantities on the left hand side of (213).

Putting together the local-in-time Proposition 5.3.2 and the $(T+\chi \upomega _+ \Phi )$-energy estimate Proposition 5.3.1 we obtain first the following:

#### Proposition 9.4.2

With the notation of Proposition 9.4.1, we have the additional estimates218
219
220
221Here the subindex $\left[ \eta \right]$ is equal to $\eta$ in case of $s=+2$ and it is dropped entirely in case $s=-2$.

#### Proof

For estimate (218) one applies Proposition 5.3.2 (applied with $\tau _1=0$ and with $\tau _{\mathrm{step}}=\varepsilon ^{-1}$) and Proposition 9.4.1 and the fact that the cutoff $\Xi =1$ identically in the region ${\tilde{t}}^*\in [\varepsilon ^{-1},\tau _{\mathrm{final}}-\varepsilon ^{-1}]$ and in the region $\{r^*\ge 2A_2^*\}\cup \{r^*\le 2A_1^*\}$. Estimate (219) follows similarly from (214).

Estimate (220) now follows from (218) and Proposition 5.3.1 applied with $\tau _1=0$ and $0\le \tau _2\le \tau _{\mathrm{final}}- \varepsilon ^{-1}$.

Finally, to obtain (221), we argue as follows. Revisiting the transport estimates of Sect. 5.2, we can estimate the left hand side from initial data, ${\mathbb {I}}_{\eta }^{\mathrm{deg}}[\Psi ^{[\pm 2]}](0,\tau _{\mathrm{final}}-\varepsilon ^{-1})$, the left hand side of (219) and the left hand side of (220). $\square$

Using once again the auxiliary estimates of Sect. 5.3, we can now improve this to:

#### Proposition 9.4.3


222
223


#### Proof

Appealing to Proposition 5.3.2 with $\tau _1=\tau _{\mathrm{final}}-\varepsilon ^{-1}$ and with $\tau _{\mathrm{step}}=\varepsilon ^{-1}$, and using (221), we obtain224Finally, we apply (220) to absorb the last term on the right hand side, obtaining thus (222). Repeating now the proof of (220) we obtain (223). $\square$

### Controlling the Term  and Finishing the Proof of Theorem 9.1

Finally, we control the error term  arising from the cutoff.

#### Proposition 9.5.1


225


#### Proof

Recallinglet us further partition  as  where we define226
227We will show the above estimate for ${\mathfrak {H}}_1$, ${\mathfrak {H}}_2$ and .

Let us first deal with the term . This is supported in

228 $$\begin{aligned} \Big (\{ 0\le {\tilde{t}}^*\le \varepsilon ^{-1} \}\cup \{ \tau _{\mathrm{final}}-\varepsilon ^{-1}\le {\tilde{t}}^*\le \tau _{\mathrm{final}} \} \Big ) \cap \{2A_1^*\le r^*\le 2A_2^*\} \end{aligned}$$

and consists of quadratic terms one of which always contains a $\Psi ^{[\pm 2]}$-term. Thus, by Cauchy–Schwarz this can easily be bounded by the first three lines of the right hand side of (225), where an $\varepsilon ^{-1}$ factor is introduced on the $\Psi$ term, compensated by an $\varepsilon$ on the other terms. (The extra $\varepsilon$ factor in $\varepsilon ^{-2}$ arises from estimating a spacetime integral by the supremum. Cf. the proof of (209).)

For ${\mathfrak {H}}_1$, by the exact Plancherel formulae of Sect. 6.2.3, the integral (226) transforms into a physical space integral supported in

229 $$\begin{aligned} \Big (\{ 0\le {\tilde{t}}^*\le \varepsilon ^{-1} \}\cup \{ \tau _{\mathrm{final}}-\varepsilon ^{-1}\le {\tilde{t}}^*\le \tau _{\mathrm{final}} \} \Big ) \cap \{A_1^*\le r^*\le A_2^*\} \end{aligned}$$

which similarly to before, is obviously estimable from the first three lines of the right hand side of (225). (In fact, one could replace the factor $\varepsilon ^{-2}$ with 1, since, just as in the proof of (208), ${\tilde{t}}^*$ derivatives of the cutoff $\Xi$ always generate extra $\varepsilon$ factors; we will use this idea below for estimating the remaining term.)

For ${\mathfrak {H}}_2$, we first apply Cauchy–Schwarz, introducing a $\varepsilon ^{-1}$,

$$\begin{aligned} \left| {\mathfrak {H}}_2^{[\pm 2]} \right| \lesssim \int _{-\infty }^{\infty }\sum _{m\ell }\int _{A_1^*}^{A_2^*}dr^* \varepsilon ^{-1} \Vert {\mathfrak {G}}^{(a\omega )}_{m\ell }\Vert ^2 + \varepsilon \Vert (\Psi ^{[\pm 2], (a\omega )}_{m\ell }, (\Psi ')^{[\pm 2], (a\omega )}_{m\ell })\Vert ^2, \end{aligned}$$

where we have used (165) to bound the *f*, $f'$ and *y* factors uniformly over frequencies. We now apply Plancherel. We note that by Proposition 9.2.1, the first term on the right hand side is bounded by $\varepsilon ^{-1}\times$ the right hand side of (208) while the second term is manifestly bounded by

$$\begin{aligned} \varepsilon {\mathbb {I}}^{\mathrm{trap}}[\Psi ^{[\pm 2]}](0,\tau _{\mathrm{final}}). \end{aligned}$$

We obtain (225) for ${\mathfrak {H}}_2$, finishing the proof. $\square$

#### Proposition 9.5.2

For sufficiently small $a_0\ll \varepsilon \ll 1$, we have

230 $$\begin{aligned} \left| {\mathfrak {H}}^{[\pm 2]} \right| \lesssim \varepsilon ^{-3}{\mathbb {D}}^{[\pm 2]}(0) \end{aligned}$$

#### Proof

Apply Proposition 5.3.1 of Sect. 5.3 to the estimate of Proposition 9.5.1 and combine with Proposition 9.4.3. $\square$

Now let $\varepsilon$ be fixed by the requirement of the above proposition. From (230) and Proposition 9.4.3 all statements of Theorem 9.1 now follow.

### Note on the Axisymmetric Case: A Pure Physical-Space Proof

We note that in the axisymmetric case $\partial _\phi \upalpha ^{[\pm 2]}=0$, the physical space multiplier and transport estimates of Sect. 5 can be applied directly *globally* in the region $\widetilde{{\mathcal {R}}}(\tau _1,\tau _2)$, i.e. without the restriction to $\widetilde{{\mathcal {R}}}^{\mathrm{away}}(\tau _1,\tau _2)$. This leads already to a much shorter proof of Theorem 9.1 which can be expressed entirely with physical space methods. We explain how this physical-space proof can be distilled directly from the more general calculations of Sect. 8 done at fixed frequency.

Given $|a|<a_0\ll M$ sufficiently small, let $r_{\mathrm{trap}}$ be the unique value given by Lemma 8.2.1 and define *f* by (176) and *y* by $\delta _1((1-\chi )f+\chi f^3-\delta _1{\tilde{\chi }}(r)r^{-\eta })$ where $\chi$ is the cutoff appearing in (177) and ${\tilde{\chi }}$ is the cutoff appearing in (101). The calculation of Sect. 8.2.3 now shows that the coercivity property of the physical space current $I^f+I^y$ holds globally in $\widetilde{{\mathcal {R}}}^{\mathrm{trap}}(\tau _1,\tau _2)$ and thus (102) holds when integrated globally in $\widetilde{{\mathcal {R}}}(\tau _1,\tau _2)$, i.e. without restriction to the “away” region and with ${\mathbb {I}}^{\mathrm{away}}$ replaced by ${\mathbb {I}}^{\mathrm{deg}}_\eta$. One also produces an estimate for the future boundary term:

231 $$\begin{aligned} {\mathbb {E}}_\eta \left[ \Psi ^{\pm 2}\right] (\tau _2) \end{aligned}$$

in view of property 3. of the proof of Proposition 5.1.1.

We apply this estimate then in the region $\widetilde{{\mathcal {R}}}(0,\tau _2)$ directly to $\Psi ^{[\pm 2]}$ arising from a solution $\upalpha ^{[\pm 2]}$ of the homogeneous Teukolsky equation (37).

We must estimate the error term arising from the coupling ${\mathcal {J}}^{[\pm 2]}$. For this we turn first to global transport estimates.

Note that in the axisymmetric case, the simple estimate applied in Sect. 8.3 for frequencies in the range ${{\mathcal {F}}}^\sharp$ applies now for all frequencies (since ${{\mathcal {F}}}^\flat =\emptyset$ if $m=0$) and corresponds to commuting the transport equations by $\partial _{r*}$ and integrating by parts. This physical space procedure, say in the $[+2]$ case, allows one to obtain the estimate

232 $$\begin{aligned}&{\mathbb {E}}_{r=A_1}\left[ \upalpha ^{[+2]}\right] (0,\tau _2) +{\mathbb {E}}_{r=A_1}\left[ \uppsi ^{[+2]}\right] (0,\tau _2) +{\mathbb {E}}^{\mathrm{trap}}\left[ \upalpha ^{[+2]}\right] (\tau _2) +{\mathbb {E}}^{\mathrm{trap}}\left[ \upalpha ^{[+2]}\right] (\tau _2)\nonumber \\&\quad +{\mathbb {I}}^{\mathrm{trap}} \left[ \upalpha ^{[+2]}\right] (0,\tau _2) +{\mathbb {I}}^{\mathrm{trap}}\left[ \uppsi ^{[+2]}\right] (0,\tau _2) \lesssim {\mathbb {I}}^{\mathrm{trap}}\left[ \Psi ^{[+2]}\right] (0,\tau _2) +{\mathbb {D}}^{[+2]}(0). \end{aligned}$$

Note that ${\mathbb {I}}^{\mathrm{trap}}(0,\tau _2)$ is degenerate and thus controlled by ${\mathbb {I}}^{\mathrm{deg}}_\eta (0,\tau _2)$. Summing (232) with the estimates obtained from (105) and (106), as in the proof of Proposition 9.4.1, allows one to estimate finally

233 $$\begin{aligned}&{\mathbb {E}}_{\eta }\left[ \upalpha ^{[+2]}\right] (\tau _2) +{\mathbb {E}}_{\eta } \left[ \upalpha ^{[+2]}\right] (\tau _2) +{\mathbb {I}}_{[\eta ]} \left[ \upalpha ^{[\pm 2]}\right] (\tau _1,\tau _2)\nonumber \\&\quad +{\mathbb {I}}_{[\eta ]} \left[ \uppsi ^{[\pm 2]} \right] (\tau _1,\tau _2) \lesssim {\mathbb {I}}^{\mathrm{deg}}_\eta \left[ \Psi ^{[\pm 2]}\right] (\tau _1,\tau _2)+ {\mathbb {D}}^{[\pm 2]}(0). \end{aligned}$$

With this we estimate the new contribution to ${\mathcal {J}}^{\pm 2]}$ coming from the region ${\mathcal {R}}^{\mathrm{trap}}(\tau _1,\tau _2)$. The only difficult term is the one arising from the *T* multiplier. In the fixed frequency estimate of Sect. 8.4, this corresponded to passing an $\omega$ from $\psi$ to $\Psi$ before applying Cauchy–Schwarz. In physical space, this corresponds simply to integration by parts in *t*. By this physical space estimate, we obtain that the resulting term is bounded by

234 $$\begin{aligned}&|a| {\mathbb {I}}^{\mathrm{trap}}[\Psi ](0,\tau _2) + |a| {\mathbb {I}}^{\mathrm{trap}} [\upalpha ](0,\tau _2) +|a| {\mathbb {I}}^{\mathrm{trap}} [\uppsi ] (0,\tau _2) \nonumber \\&\quad + |a| {\mathbb {E}}^{\mathrm{trap}}_{\widetilde{\Sigma }_{\tau }}\left[ \Psi ^{[\pm 2]}\right] (\tau _2) +|a|{\mathbb {E}}^{\mathrm{trap}}_{\widetilde{\Sigma }_{\tau }}\left[ \upalpha ^{[\pm 2]}\right] (\tau _2) +|a|{\mathbb {E}}^{\mathrm{trap}}_{\widetilde{\Sigma }_{\tau }}\left[ \uppsi ^{[\pm 2]}\right] (\tau _2) +|a| {\mathbb {D}}^{[\pm 2]}(0), \end{aligned}$$

where the future boundary terms arise from this integration by parts. (Note that all other terms in ${\mathcal {J}}^{[\pm 2]}$ are estimated by the first line of (234) alone.) Combining with the original statement of Proposition 9.4.1, this yields

235 $$\begin{aligned} {\mathbb {E}}_{\widetilde{\Sigma }_\tau ,\eta }\left[ \Psi ^{[\pm 2]}\right] +{\mathbb {I}}^{\mathrm{deg}}_\eta \left[ \Psi ^{[\pm 2]}\right] (\tau _1,\tau _2) \lesssim {\mathbb {D}}^{[\pm 2]}(0) + (234). \end{aligned}$$

In view of (233), for sufficiently small $|a|<a_0\ll M$, one can absorb the terms (234) on the right hand side of (235) into the left hand side. The remaining statements of Theorem 9.1 follow immediately.

## The Redshift Effect and Its Associated Morawetz Estimate

In this section we will obtain statement 2. of Theorem 4.1 concerning the boundedness and integrated local energy decay of the so-called red-shifted energy. The required statement is contained in Theorem 10.1 below.

### Statement of Red-Shifted Boundedness and Integrated Decay

#### Theorem 10.1

Let $\upalpha ^{[\pm 2]}$, $\Psi ^{[\pm 2]}$ and $\uppsi ^{[\pm 2]}$ be as in Theorem 4.1. Then the following holds for any $\tau _2 > \tau _1\ge 0$.

For

- the basic degenerate Morawetz estimate
  236 $$\begin{aligned}&\ \overline{{\mathbb {I}}}^{\mathrm{deg}}_{\eta } \left[ \Psi ^{[+2]}\right] \left( \tau _1,\tau _2\right) +{\mathbb {I}}_\eta \left[ \uppsi ^{[+2]}\right] \left( \tau _1,\tau _2\right) +{\mathbb {I}}_\eta \left[ \upalpha ^{[+2]}\right] \left( \tau _1,\tau _2\right) \nonumber \\&\quad \lesssim \ \ \overline{{\mathbb {E}}}_{{\widetilde{\Sigma }}_{\tau },\eta } \left[ \Psi ^{[+2]}\right] \left( \tau _1\right) +{\mathbb {E}}_{{\widetilde{\Sigma }}_{\tau },\eta } \left[ \uppsi ^{[+2]}\right] \left( \tau _1\right) + {\mathbb {E}}_{{\widetilde{\Sigma }}_{\tau },\eta } \left[ \upalpha ^{[+2]}\right] \left( \tau _1\right) \, , \end{aligned}$$
- the basic non-degenerate Morawetz estimate
  237 $$\begin{aligned}&\overline{{\mathbb {I}}}_{\eta } \left[ \Psi ^{[+2]}\right] \left( \tau _1,\tau _2\right) \lesssim \ \ \overline{{\mathbb {E}}}_{{\widetilde{\Sigma }}_{\tau },\eta } \left[ \Psi ^{[+2]}\right] \left( \tau _1\right) +{\mathbb {E}}_{{\widetilde{\Sigma }}_{\tau },\eta } \left[ \uppsi ^{[+2]}\right] \left( \tau _1\right) \nonumber \\&\quad + {\mathbb {E}}_{{\widetilde{\Sigma }}_{\tau },\eta } \left[ \upalpha ^{[+2]}\right] \left( \tau _1\right) \nonumber \\&\quad + \overline{{\mathbb {E}}}_{{\widetilde{\Sigma }}_{\tau },\eta } \left[ T\Psi ^{[+2]}\right] \left( \tau _1\right) +{\mathbb {E}}_{{\widetilde{\Sigma }}_{\tau },\eta } \left[ T\uppsi ^{[+2]}\right] \left( \tau _1\right) + {\mathbb {E}}_{{\widetilde{\Sigma }}_{\tau },\eta } \left[ T\upalpha ^{[+2]}\right] \left( \tau _1\right) \, , \end{aligned}$$
- the $\eta$-weighted energy boundedness estimate
  238 $$\begin{aligned}&\overline{{\mathbb {E}}}_{{\mathcal {H}}^+} \left[ \Psi ^{[+2]}\right] \left( \tau _1,\tau _2\right) + \overline{{\mathbb {E}}}_{{\widetilde{\Sigma }}_{\tau },\eta } \left[ \Psi ^{[+2]}\right] \left( \tau _2\right) \nonumber \\&\quad \lesssim \overline{{\mathbb {E}}}_{{\widetilde{\Sigma }}_{\tau },\eta } \left[ \Psi ^{[+2]}\right] \left( \tau _1\right) +{\mathbb {E}}_{{\widetilde{\Sigma }}_{\tau },\eta } \left[ \uppsi ^{[+2]}\right] \left( \tau _1\right) + {\mathbb {E}}_{{\widetilde{\Sigma }}_{\tau },\eta } \left[ \upalpha ^{[+2]}\right] \left( \tau _1\right) \, . \end{aligned}$$

For

- the basic degenerate Morawetz estimate
  239 $$\begin{aligned}&\overline{{\mathbb {I}}}^{\mathrm{deg}}_{\eta } \left[ \Psi ^{[-2]}\right] \left( \tau _1,\tau _2\right) +{\mathbb {I}} \left[ \uppsi ^{[-2]}\right] \left( \tau _1,\tau _2\right) +{\mathbb {I}} \left[ \upalpha ^{[-2]}\right] \left( \tau _1,\tau _2\right) \nonumber \\&\quad \lesssim \ \ \overline{{\mathbb {E}}}_{{\widetilde{\Sigma }}_{\tau },\eta } \left[ \Psi ^{[-2]}\right] \left( \tau _1\right) +{\mathbb {E}}_{{\widetilde{\Sigma }}_{\tau }} \left[ \uppsi ^{[-2]}\right] \left( \tau _1\right) + {\mathbb {E}}_{{\widetilde{\Sigma }}_{\tau }} \left[ \upalpha ^{[-2]}\right] \left( \tau _1\right) \, , \end{aligned}$$
- the basic non-degenerate Morawetz estimate
  240 $$\begin{aligned}&\overline{{\mathbb {I}}}_{\eta } \left[ \Psi ^{[-2]}\right] \left( \tau _1,\tau _2\right) \lesssim \ \ \overline{{\mathbb {E}}}_{{\widetilde{\Sigma }}_{\tau },\eta } \left[ \Psi ^{[-2]}\right] \left( \tau _1\right) +{\mathbb {E}}_{{\widetilde{\Sigma }}_{\tau }} \left[ \uppsi ^{[-2]}\right] \left( \tau _1\right) + {\mathbb {E}}_{{\widetilde{\Sigma }}_{\tau }} \left[ \upalpha ^{[-2]}\right] \left( \tau _1\right) \nonumber \\&\quad + \overline{{\mathbb {E}}}_{{\widetilde{\Sigma }}_{\tau },\eta } \left[ T\Psi ^{[-2]}\right] \left( \tau _1\right) +{\mathbb {E}}_{{\widetilde{\Sigma }}_{\tau }} \left[ T\uppsi ^{[-2]}\right] \left( \tau _1\right) + {\mathbb {E}}_{{\widetilde{\Sigma }}_{\tau }} \left[ T\upalpha ^{[-2]}\right] \left( \tau _1\right) \, , . \end{aligned}$$
- the $\eta$-weighted energy boundedness estimate
  241 $$\begin{aligned}&\overline{{\mathbb {E}}}_{{\mathcal {H}}^+} \left[ \Psi ^{[-2]}\right] \left( \tau _1,\tau _2\right) +\overline{{\mathbb {E}}}_{{\widetilde{\Sigma }}_{\tau },\eta } \left[ \Psi ^{[-2]}\right] \left( \tau _2\right) \nonumber \\&\quad \lesssim \overline{{\mathbb {E}}}_{{\widetilde{\Sigma }}_{\tau },\eta } \left[ \Psi ^{[-2]}\right] \left( \tau _1\right) +{\mathbb {E}}_{{\widetilde{\Sigma }}_{\tau }} \left[ \uppsi ^{[-2]}\right] \left( \tau _1\right) + {\mathbb {E}}_{{\widetilde{\Sigma }}_{\tau }} \left[ \upalpha ^{[-2]}\right] \left( \tau _1\right) \, . \end{aligned}$$

### Proof of Theorem 10.1

We only prove the $s=+\,2$ case. The $s=-\,2$ case is completely analogous and slightly easier because the term ${\mathcal {J}}^{[-2]}$ has stronger degeneration near the event horizon. Note that in Sect. 9 we have already proven the estimates (236) and (238) provided we drop all overbars from the energies that appear. The estimate (237), which does not degenerate in a neighbourhood of $r=3M$ but loses a derivative, is a simple corollary of (236) and (238) again provided we drop all overbars from the energies. Hence the only task left is to improve the ${\underline{L}}$ derivative in the energies that appear. This is achieved using the redshift multiplier of [39, 43]:

*The redshift identity.* Recall the notational conventions of Sect. 5.1.1. Multiplying (54) by $Y=\frac{1}{w} \xi {\underline{L}}\overline{\Psi }$ (with $\xi$ a smooth radial cut-off function equal to 1 for $r \in \left[ r_+, r_+ +\frac{1}{4}M\right]$ and equal to zero for $r \ge r_+ + \frac{1}{2}M$) and taking the real parts yields (use the formulae of Appendix B.5)

242 $$\begin{aligned} {\underline{L}} \big \{F^{Y}_{{\underline{L}}}\big \} +L \big \{F^{Y}_{L} \big \} + I^{Y} \equiv \mathrm{Re} \left( -\left( {\mathcal {J}}^{[s]} + {\mathfrak {G}}^{[s]}\right) \frac{1}{w} \xi {\underline{L}}\overline{\Psi }\right) \end{aligned}$$

where243
244
245We apply the identity (242) to the equation satisfied by $\Psi ^{[+2]}$. In particular, ${\mathfrak {G}}^{[s]}=0$ because $\alpha ^{[+2]}$ satisfies the *homogeneous* Teukolsky equation. Upon integration over $\widetilde{{\mathcal {R}}}\left( \tau _1,\tau _2\right)$ (recalling Remark 5.1) we obtain (236) and (238) after making the following observations:

- The first term in $F^Y_L$ and the first term in $F^Y_{{\underline{L}}}$ are manifestly non-negative and produce precisely the desired improvement in the ${\underline{L}}$ derivative and the missing angular derivative in the horizon term respectively. All other terms appearing as boundary terms can now be controlled using Cauchy–Schwarz and (35) by the energies without the overbar (sometimes borrowing an $\epsilon$ from the just obtained good ${\underline{L}}$-derivative term and the good angular term respectively is required).
- Examining (245), the term $\frac{1}{2} \xi \frac{w^\prime }{w^2} | {\underline{L}} \Psi |^2$ is manifestly positive and produces precisely the desired improvement of the $|{\underline{L}} \Psi |^2$ in the spacetime energy without the overbar. All other terms can be controlled by the spacetime energy without the overbar, sometimes borrowing an $\epsilon$ from the improved $|{\underline{L}} \Psi |^2$ term.
- The error term
  $$\begin{aligned} \int _{{\mathcal {M}}\left( \tau _1,\tau _2\right) } \Big |{\mathcal {J}}^{[+2]} \frac{1}{w} \xi {\underline{L}}\overline{\Psi }\Big | \frac{1}{\rho ^2} \frac{r^2+a^2}{\Delta } \end{aligned}$$
  is controlled using Cauchy’s inequality with $\epsilon$ and the energies ${\mathbb {I}}_0 \left[ \uppsi ^{[+2]}\right] \left( \tau _1,\tau _2\right) +{\mathbb {I}}_0 \left[ \upalpha ^{[+2]}\right] \left( \tau _1,\tau _2\right)$.

Finally, the estimate (237) follows from its un-overbarred version by adding the just established (236).

## The $r^p$-Weighted Hierarchy and the Main Decay Result

To complete the proof of Theorem 4.1, it remains to obtain statements 3. and 4. concerning the $r^p$-weighted hierarchy and polynomial decay. The required statement is contained in Theorem 11.1 below.

### Statement of the Decay Theorem

#### Theorem 11.1

Let $\upalpha ^{[\pm 2]}$, $\Psi ^{[\pm 2]}$ and $\uppsi ^{[\pm 2]}$ be as in Theorem 4.1. Then the following holds for any $\tau > \tau _0=0$.

For  we have

246 $$\begin{aligned}&\overline{{\mathbb {E}}}_{{\widetilde{\Sigma }}_{\tau },\eta } \left[ \Psi ^{[+2]}\right] \left( \tau \right) + {{\mathbb {E}}}_{{\widetilde{\Sigma }}_{\tau },\eta } \left[ \uppsi ^{[+2]}\right] \left( \tau \right) + {\mathbb {E}}_{{\widetilde{\Sigma }}_{\tau },\eta } \left[ \upalpha ^{[+2]}\right] \left( \tau \right) \nonumber \\&\quad \lesssim \frac{{\mathbb {D}}_{2, 2} \left[ \Psi ^{[+2]}, \uppsi ^{[+2]}, \upalpha ^{[+2]}\right] \left( \tau _0\right) }{\tau ^{2-\eta }} \, \end{aligned}$$

for the initial data energy

$$\begin{aligned}&{\mathbb {D}}_{2, 2} \left[ \Psi ^{[+2]}, \uppsi ^{[+2]}, \upalpha ^{[+2]}\right] \left( \tau _0\right) = \sum _{k=0}^1 \left( \overline{{\mathbb {E}}}_{{\widetilde{\Sigma }}_{\tau },2} \left[ T^k\Psi ^{[+2]}\right] \left( \tau _0\right) \right. \\&\quad \left. +\, {\mathbb {E}}_{{\widetilde{\Sigma }}_{\tau },2} \left[ T^k \uppsi ^{[+2]}\right] \left( \tau _0\right) + {\mathbb {E}}_{{\widetilde{\Sigma }}_{\tau },2} \left[ T^k\upalpha ^{[+2]}\right] \left( \tau _0\right) \right) \\&\quad +\, \overline{{\mathbb {E}}}_{{\widetilde{\Sigma }}_{\tau },\eta } \left[ T^{2}\Psi ^{[+2]}\right] \left( \tau _0\right) +{\mathbb {E}}_{{\widetilde{\Sigma }}_{\tau },\eta } \left[ T^{2}\uppsi ^{[+2]}\right] \left( \tau _0\right) + {\mathbb {E}}_{{\widetilde{\Sigma }}_{\tau },\eta } \left[ T^{2}\upalpha ^{[+2]}\right] \left( \tau _0\right) \, . \end{aligned}$$

For  we have

247 $$\begin{aligned}&\overline{{\mathbb {E}}}_{{\widetilde{\Sigma }}_{\tau },\eta } \left[ \Psi ^{[-2]}\right] \left( \tau \right) + {{\mathbb {E}}}_{{\widetilde{\Sigma }}_{\tau }} \left[ \uppsi ^{[-2]}\right] \left( \tau \right) +{\mathbb {E}}_{{\widetilde{\Sigma }}_{\tau }} \left[ \upalpha ^{[-2]}\right] \left( \tau \right) \nonumber \\&\quad \lesssim \frac{{\mathbb {D}}_{2, 2} \left[ \Psi ^{[-2]}, \uppsi ^{[-2]}, \upalpha ^{[-2]}\right] \left( \tau _0\right) }{\tau ^{2-\eta }} \, \end{aligned}$$

and

248 $$\begin{aligned}&\overline{{\mathbb {E}}}_{{\widetilde{\Sigma }}_{\tau },\eta } \left[ \Psi ^{[-2]}\right] \left( \tau \right) + \overline{{\mathbb {E}}}_{{\widetilde{\Sigma }}_{\tau }} \left[ \uppsi ^{[-2]}\right] \left( \tau \right) +\overline{{\mathbb {E}}}_{{\widetilde{\Sigma }}_{\tau }} \left[ \upalpha ^{[-2]}\right] \left( \tau \right) \nonumber \\&\quad \lesssim \frac{\overline{{\mathbb {D}}}_{2, 2} \left[ \Psi ^{[-2]}, \uppsi ^{[-2]}, \upalpha ^{[-2]}\right] \left( \tau _0\right) }{\tau ^{2-\eta }} \, \end{aligned}$$

for the initial data energy

$$\begin{aligned}&{\mathbb {D}}_{2, 2} \left[ \Psi ^{[-2]}, \uppsi ^{[-2]}, \upalpha ^{[-2]}\right] \left( \tau _0\right) = \sum _{k=0}^1 \left( \overline{{\mathbb {E}}}_{{\widetilde{\Sigma }}_{\tau },2} \left[ T^k\Psi ^{[-2]}\right] \left( \tau _0\right) \right. \\&\quad \left. + {\mathbb {E}}_{{\widetilde{\Sigma }}_{\tau }} \left[ T^k \uppsi ^{[-2]}\right] \left( \tau _0\right) + {\mathbb {E}}_{{\widetilde{\Sigma }}_{\tau }} \left[ T^k\upalpha ^{[-2]}\right] \left( \tau _0\right) \right) \\&\quad + \overline{{\mathbb {E}}}_{{\widetilde{\Sigma }}_{\tau },\eta } \left[ T^{2}\Psi ^{[-2]}\right] \left( \tau _0\right) +{\mathbb {E}}_{{\widetilde{\Sigma }}_{\tau }} \left[ T^{2}\uppsi ^{[-2]}\right] \left( \tau _0\right) + {\mathbb {E}}_{{\widetilde{\Sigma }}_{\tau }} \left[ T^{2}\upalpha ^{[-2]}\right] \left( \tau _0\right) \, , \end{aligned}$$

and with $\overline{{\mathbb {D}}}_{2, 2} \left[ \Psi ^{[-2]}, \uppsi ^{[-2]}, \upalpha ^{[-2]}\right] \left( \tau _0\right)$ defined by putting an overbar on all energies appearing in ${\mathbb {D}}_{2, 2} \left[ \Psi ^{[-2]}, \uppsi ^{[-2]}, \upalpha ^{[-2]}\right] \left( \tau _0\right)$.

### Proof of Theorem 11.1 for $s=+2$

The $s=+2$ case of Theorem 11.1 will be proven in Sect. 11.2.3 by combining basic estimates from the $r^p$ hierarchy associated with the inhomogeneous wave equation satisfied by $\Psi ^{[+2]}$ (derived in Sect. 11.2.1) and basic transport estimates for $\uppsi ^{[+2]}$ and $\upalpha ^{[+2]}$ (derived in Sect. 11.2.2).

#### The Weighted $r^p$ Hierarchy for $\Psi ^{[+2]}$ in Physical Space

##### Proposition 11.2.1

Under the assumptions of Theorem 11.1 we have for any $\tau _2 > \tau _1\ge 0$ and for $p=2$, $p=1$ and $p=\eta$ the estimate

$$\begin{aligned}&\overline{{\mathbb {E}}}_{{\widetilde{\Sigma }}_{\tau },p} \left[ \Psi ^{[+2]}\right] \left( \tau _2\right) + \overline{{\mathbb {I}}}^{\mathrm{deg}}_{p} \left[ \Psi ^{[+2]}\right] \left( \tau _1,\tau _2\right) + {\mathbb {E}}_{{\mathcal {I}}^+,p} \left[ \Psi ^{[+2]}\right] \left( \tau _1,\tau _2\right) \\&\quad \lesssim \overline{{\mathbb {E}}}_{{\widetilde{\Sigma }}_{\tau },p} \left[ \Psi ^{[+2]}\right] \left( \tau _1\right) +{\mathbb {E}}_{{\widetilde{\Sigma }}_{\tau },\eta } \left[ \uppsi ^{[+2]}\right] \left( \tau _1\right) + {\mathbb {E}}_{{\widetilde{\Sigma }}_{\tau },\eta } \left[ \upalpha ^{[+2]}\right] \left( \tau _1\right) \, \end{aligned}$$

and the non-degenerate estimate

$$\begin{aligned}&\overline{{\mathbb {E}}}_{{\widetilde{\Sigma }}_{\tau },p} \left[ \Psi ^{[+2]}\right] \left( \tau _2\right) + \overline{{\mathbb {I}}}_{p} \left[ \Psi ^{[+2]}\right] \left( \tau _1,\tau _2\right) + {\mathbb {E}}_{{\mathcal {I}}^+,p} \left[ \Psi ^{[+2]}\right] \left( \tau _1,\tau _2\right) \\&\quad \lesssim \overline{{\mathbb {E}}}_{{\widetilde{\Sigma }}_{\tau },p} \left[ \Psi ^{[+2]}\right] \left( \tau _1\right) +{\mathbb {E}}_{{\widetilde{\Sigma }}_{\tau },\eta } \left[ \uppsi ^{[+2]}\right] \left( \tau _1\right) + {\mathbb {E}}_{{\widetilde{\Sigma }}_{\tau },\eta } \left[ \upalpha ^{[+2]}\right] \left( \tau _1\right) \\&\qquad + \overline{{\mathbb {E}}}_{{\widetilde{\Sigma }}_{\tau },\eta } \left[ T\Psi ^{[+2]}\right] \left( \tau _1\right) +{\mathbb {E}}_{{\widetilde{\Sigma }}_{\tau },\eta } \left[ T\uppsi ^{[+2]}\right] \left( \tau _1\right) + {\mathbb {E}}_{{\widetilde{\Sigma }}_{\tau },\eta } \left[ T\upalpha ^{[+2]}\right] \left( \tau _1\right) . \end{aligned}$$

##### Proof

Given $\alpha ^{[+2]}$ we apply the multiplier identity (96) to $\Psi ^{[+2]}$. To the identity that is being produced after integration over $\widetilde{{\mathcal {R}}}(\tau _1,\tau _2)$, we can add a large constant *B* (depending only on *M*) times the basic estimate (236) such that the following holds: For the boundary term we have for all $p \in \left[ \eta ,2\right]$

249 $$\begin{aligned}&\int _{\widetilde{{\mathcal {R}}}(\tau _1,\tau _2)} \left( L \big \{ F^{r^p}_L\big \} + {\underline{L}} \big \{ F^{r^p}_{{\underline{L}}} \big \}\right) \frac{1}{\rho ^2} \frac{r^2+a^2}{\Delta } dVol + B \cdot \overline{{\mathbb {E}}}_{\widetilde{\Sigma }_\tau ,\eta } \left[ \Psi ^{[+2]}\right] (\tau _2) \nonumber \\&\quad \gtrsim b \cdot \overline{{\mathbb {E}}}_{\widetilde{\Sigma }_\tau ,p} \left[ \Psi ^{[+2]}\right] (\tau _2) - B \cdot \overline{{\mathbb {E}}}_{\widetilde{\Sigma }_\tau ,p} \left[ \Psi ^{[+2]}\right] (\tau _1) + b\ {\mathbb {E}}_{{\mathcal {I}}^+,p} \left[ \Psi ^{[+2]}\right] \left( \tau _1,\tau _2\right) \, . \end{aligned}$$

For the spacetime term we have

250 $$\begin{aligned}&\int _{\widetilde{{\mathcal {R}}}(\tau _1,\tau _2)} \left( I^{r^{p}}\right) \frac{1}{\rho ^2} \frac{r^2+a^2}{\Delta } dVol + B \cdot \overline{{\mathbb {I}}}^{deg}_{\eta } \left[ \Psi ^{[+2]}\right] (\tau _1,\tau _2)\nonumber \\&\quad \ge b \cdot \overline{{\mathbb {I}}}^{\mathrm{deg}}_{p} \left[ \Psi ^{[+2]}\right] (\tau _1,\tau _2) \, \end{aligned}$$

for $p \in \left[ \eta ,2\right)$ and for $p=2$

251 $$\begin{aligned}&\sum _{p=2-\eta , p=2} \int _{\widetilde{{\mathcal {R}}}(\tau _1,\tau _2)} \left( I^{r^{p}}\right) \frac{1}{\rho ^2} \frac{r^2+a^2}{\Delta } dVol + B \cdot \overline{{\mathbb {I}}}^{deg}_{\eta } \left[ \Psi ^{[+2]}\right] (\tau _1,\tau _2) \nonumber \\&\qquad \ge b \cdot \overline{{\mathbb {I}}}^{\mathrm{deg}}_{2} \left[ \Psi ^{[+2]}\right] (\tau _1,\tau _2) \, , \end{aligned}$$

the latter case being special because for $p=2$ we lose control of the angular derivatives in (99). For the error term (which in view of $\xi$ being supported for large *r* is supported for large *r*) we have, for any $\lambda >0$,

$$\begin{aligned}&\int _{\widetilde{{\mathcal {R}}}(\tau _1,\tau _2)} \Big | {\mathcal {J}}^{[+2]}| |\xi | |\beta _4| |r^p L \Psi ^{[+2]}| \frac{r^2+a^2}{\Delta \rho ^2} dVol \\&\quad \lesssim \int _{\widetilde{{\mathcal {R}}}(\tau _1,\tau _2)} dVol \frac{r^2+a^2}{\Delta \rho ^2} \left( \lambda r^{p-1} |L\Psi ^{[+2]}|^2 + \frac{r^{p+1}}{\lambda } \Big | {\mathcal {J}}^{[+2]}|^2 \right) \\&\quad \lesssim \lambda \overline{{\mathbb {I}}}^{deg}_{p} \left[ \Psi ^{[+2]}\right] (\tau _1,\tau _2) + \frac{a^2}{\lambda }\left( {\mathbb {I}}_\eta \left[ \uppsi ^{[+2]}\right] \left( \tau _1,\tau _2\right) +{\mathbb {I}}_\eta \left[ \upalpha ^{[+2]}\right] \left( \tau _1,\tau _2\right) \right) \, . \end{aligned}$$

Note that there is no ${\mathfrak {G}}^{[+2]}$ error term as $F^{[+2]}=0$ and hence ${\mathfrak {G}}^{[+2]}=0$. Combining the above estimates yields the first estimate of the Proposition after using the basic estimate (236) yields and choosing $\lambda$ sufficiently small (depending only on *M*). The second estimate follows immediately by combining the first one with the non-degenerate (237). $\square$

#### Physical Space Weighted Transport for $\uppsi ^{[+2]}$ and $P^{[+2]}$

We now turn to deriving weighted Morawetz and boundedness estimates for $\uppsi ^{[+2]}$ and $\upalpha ^{[+2]}$ from the transport equations they satisfy. Combining (105) with the basic estimate (236) we immediately obtain

##### Proposition 11.2.2

Under the assumptions of Theorem 11.1 we have for any $\tau _2 > \tau _1\ge 0$ and for $p \in \{\eta ,1,2\}$ the estimate

252 $$\begin{aligned} {\mathbb {E}}_{{\widetilde{\Sigma }}_{\tau },p} \left[ \upalpha ^{[+2]}\right] \left( \tau _2\right) + {\mathbb {I}}_p \left[ \upalpha ^{[+2]}\right] \left( \tau _1,\tau _2\right) \lesssim {\mathbb {I}}_p \left[ \uppsi ^{[+2]}\right] \left( \tau _1,\tau _2\right) + {\mathbb {E}}_{{\widetilde{\Sigma }}_{\tau },p} \left[ \upalpha ^{[+2]}\right] \left( \tau _1\right) \end{aligned}$$

and the estimate

253 $$\begin{aligned}&{\mathbb {E}}_{{\widetilde{\Sigma }}_{\tau },p} \left[ \uppsi ^{[+2]}\right] \left( \tau _2\right) + {\mathbb {I}}_p \left[ \uppsi ^{[+2]}\right] \left( \tau _1,\tau _2\right) \lesssim {\mathbb {I}}^{\mathrm{deg}}_p \left[ \Psi ^{[+2]}\right] \left( \tau _1,\tau _2\right) \nonumber \\&\quad + {\mathbb {E}}_{{\widetilde{\Sigma }}_{\tau },p} \left[ \uppsi ^{[+2]}\right] \left( \tau _1\right) + {\mathbb {E}}_{{\widetilde{\Sigma }}_{\tau },\eta } \left[ \Psi ^{[+2]}\right] \left( \tau _1\right) + {\mathbb {E}}_{{\widetilde{\Sigma }}_{\tau },\eta } \left[ \upalpha ^{[+2]}\right] \left( \tau _1\right) \ \end{aligned}$$

#### Completing the Proof of Theorem 11.1

Combining the estimate of Proposition 11.2.1 with that of Proposition 11.2.2 we deduce for $p \in \{\eta , 1, 2\}$ (first for $K=0$ and then by trivial commutation with the Killing field *T* for any $K \in {\mathbb {N}}$) the estimate

254 $$\begin{aligned}&\sum _{k=0}^K \left( {\mathbb {E}}_{{\widetilde{\Sigma }}_{\tau },p} \left[ T^k\upalpha ^{[+2]}\right] \left( \tau _2\right) + {\mathbb {E}}_{{\widetilde{\Sigma }}_{\tau },p} \left[ T^k \uppsi ^{[+2]}\right] \left( \tau _2\right) + \overline{{\mathbb {E}}}_{{\widetilde{\Sigma }}_{\tau },p} \left[ T^k\Psi ^{[+2]}\right] \left( \tau _2\right) \right) \nonumber \\&\qquad +\sum _{k=0}^K \left( {\mathbb {I}}_p \left[ T^k\upalpha ^{[+2]}\right] \left( \tau _1,\tau _2\right) +{\mathbb {I}}_p \left[ T^k\uppsi ^{[+2]}\right] \left( \tau _1,\tau _2\right) + \overline{{\mathbb {I}}}^{\mathrm{deg}}_{p} \left[ T^k\Psi ^{[+2]}\right] \left( \tau _1,\tau _2\right) \right) \nonumber \\&\quad \lesssim \sum _{k=0}^K \left( {\mathbb {E}}_{{\widetilde{\Sigma }}_{\tau },p} \left[ T^k\upalpha ^{[+2]}\right] \left( \tau _1\right) + {\mathbb {E}}_{{\widetilde{\Sigma }}_{\tau },p} \left[ T^k \uppsi ^{[+2]}\right] \left( \tau _1\right) + \overline{{\mathbb {E}}}_{{\widetilde{\Sigma }}_{\tau },p} \left[ T^k\Psi ^{[+2]}\right] \left( \tau _1\right) \right) \end{aligned}$$

and also

255 $$\begin{aligned}&\sum _{k=0}^K \left( {\mathbb {E}}_{{\widetilde{\Sigma }}_{\tau },p} \left[ T^k\upalpha ^{[+2]}\right] \left( \tau _2\right) + {\mathbb {E}}_{{\widetilde{\Sigma }}_{\tau },p} \left[ T^k \uppsi ^{[+2]}\right] \left( \tau _2\right) + \overline{{\mathbb {E}}}_{{\widetilde{\Sigma }}_{\tau },p} \left[ T^k\Psi ^{[+2]}\right] \left( \tau _2\right) \right) \nonumber \\&\qquad +\sum _{k=0}^K \left( {\mathbb {I}}_p \left[ T^k\upalpha ^{[+2]}\right] \left( \tau _1,\tau _2\right) +{\mathbb {I}}_p \left[ T^k\uppsi ^{[+2]}\right] \left( \tau _1,\tau _2\right) + \overline{{\mathbb {I}}}_{p} \left[ T^k\Psi ^{[+2]}\right] \left( \tau _1,\tau _2\right) \right) \nonumber \\&\quad \lesssim \sum _{k=0}^K \left( {\mathbb {E}}_{{\widetilde{\Sigma }}_{\tau },p} \left[ T^k\upalpha ^{[+2]}\right] \left( \tau _1\right) + {\mathbb {E}}_{{\widetilde{\Sigma }}_{\tau },p} \left[ T^k \uppsi ^{[+2]}\right] \left( \tau _1\right) + \overline{{\mathbb {E}}}_{{\widetilde{\Sigma }}_{\tau },p} \left[ T^k\Psi ^{[+2]}\right] \left( \tau _1\right) \right) \nonumber \\&\qquad + {\mathbb {E}}_{{\widetilde{\Sigma }}_{\tau },\eta } \left[ T^{K+1}\upalpha ^{[+2]}\right] \left( \tau _1\right) +{\mathbb {E}}_{{\widetilde{\Sigma }}_{\tau },\eta } \left[ T^{K+1}\uppsi ^{[+2]}\right] \left( \tau _1\right) + \overline{{\mathbb {E}}}_{{\widetilde{\Sigma }}_{\tau },\eta } \left[ T^{K+1}\Psi ^{[+2]}\right] \left( \tau _1\right) . \end{aligned}$$

Let us denote the right hand side of the second estimate on the initial data slice ${\widetilde{\Sigma }}_0$ (i.e. for $\tau _1=\tau _0$) by ${\mathbb {D}}_{K+1, p} \left[ \Psi ^{[+2]}, \uppsi ^{[+2]}, \upalpha ^{[+2]}\right] \left( \tau _0\right)$.

Applying (255) for $K=1$ and $p=2$ implies (after using a standard argument involving dyadic sequences) along a dyadic sequence $\tau _n \sim 2^{n}\tau _0$ the estimate

$$\begin{aligned}&\sum _{k=0}^1 \left( {\mathbb {E}}_{{\widetilde{\Sigma }}_{\tau },1} \left[ T^k \upalpha ^{[+2]}\right] \left( \tau _n\right) + {\mathbb {E}}_{{\widetilde{\Sigma }}_{\tau },1} \left[ T^k \uppsi ^{[+2]}\right] \left( \tau _n\right) + \overline{{\mathbb {E}}}_{{\widetilde{\Sigma }}_{\tau },1} \left[ T^k\Psi ^{[+2]}\right] \left( \tau _n\right) \right) \\&\quad \lesssim \frac{{\mathbb {D}}_{2, 2} \left[ \Psi ^{[+2]}, \uppsi ^{[+2]}, \upalpha ^{[+2]}\right] \left( \tau _0\right) }{\tau _n} \, . \end{aligned}$$

Using the above and applying (254) for $p=1$, $K=1$ between the time $\tau _1=\tau _n$ and any $\tau _2 \in \left( \tau _n, \tau _{n+1}\right]$ yields the previous estimate for any $\tau$, not only the members of the dyadic sequence. Turning back to (255) now with $K=0$ we use the previous estimate and a similar dyadic argument to produce along a dyadic sequence the estimate

$$\begin{aligned}&{\mathbb {E}}_{{\widetilde{\Sigma }}_{\tau },\eta } \left[ \upalpha ^{[+2]}\right] \left( \tau _n\right) + {\mathbb {E}}_{{\widetilde{\Sigma }}_{\tau },\eta } \left[ \uppsi ^{[+2]}\right] \left( \tau _n\right) + \overline{{\mathbb {E}}}_{{\widetilde{\Sigma }}_{\tau },\eta } \left[ \Psi ^{[+2]}\right] \left( \tau _n\right) \\&\quad \lesssim \frac{{\mathbb {D}}_{2, 2} \left[ \Psi ^{[+2]}, \uppsi ^{[+2]}, \upalpha ^{[+2]}\right] \left( \tau _0\right) }{\tau _n^{2-\eta }} \, . \end{aligned}$$

Using the above and applying (254) with $p=\eta$ and $K=0$ now yields the estimate (246) of Theorem 11.1.

### Proof of Theorem 11.1 for $s=-2$

The $s=-\,2$ case of Theorem 11.1 will be proven in Sect. 11.3.3 by combining basic estimates from the $r^p$ hierarchy associated with the inhomogeneous wave equation satisfied by $\Psi ^{[-2]}$ (derived in Sect. 11.3.1) and basic transport estimates for $\uppsi ^{[-2]}$ and $\upalpha ^{[-2]}$ (derived in Sect. 11.3.2).

#### The Weighted $r^p$ Hierarchy for $\Psi ^{[-2]}$ in Physical Space

##### Proposition 11.3.1

Under the assumptions of Theorem 11.1 we have for any $\tau _2 > \tau _1\ge 0$ and for $p=2$, $p=1$ and $p=\eta$ the estimate

256 $$\begin{aligned}&\overline{{\mathbb {E}}}_{{\widetilde{\Sigma }}_{\tau },p} \left[ \Psi ^{[-2]}\right] \left( \tau _2\right) + \overline{{\mathbb {I}}}^{\mathrm{deg}}_{p} \left[ \Psi ^{[-2]}\right] \left( \tau _1,\tau _2\right) + {\mathbb {E}}_{{\mathcal {I}}^+,p} \left[ \Psi ^{[-2]}\right] \left( \tau _1,\tau _2\right) \nonumber \\&\quad \lesssim \overline{{\mathbb {E}}}_{{\widetilde{\Sigma }}_{\tau },p} \left[ \Psi ^{[-2]}\right] \left( \tau _1\right) +{\mathbb {E}}_{{\widetilde{\Sigma }}_{\tau }} \left[ \uppsi ^{[-2]}\right] \left( \tau _1\right) + {\mathbb {E}}_{{\widetilde{\Sigma }}_{\tau }} \left[ \upalpha ^{[-2]}\right] \left( \tau _1\right) \nonumber \\&\qquad + |a|\left( {\mathbb {E}}_{{\mathcal {I}}^+} \left[ \uppsi ^{[-2]}\right] \left( \tau _1,\tau _2\right) +{\mathbb {E}}_{{\mathcal {I}}^+} \left[ \upalpha ^{[-2]}\right] \left( \tau _1,\tau _2\right) \right) \end{aligned}$$

and the non-degenerate estimate

257 $$\begin{aligned}&\overline{{\mathbb {E}}}_{{\widetilde{\Sigma }}_{\tau },p} \left[ \Psi ^{[-2]}\right] \left( \tau _2\right) + \overline{{\mathbb {I}}}_{p} \left[ \Psi ^{[-2]}\right] \left( \tau _1,\tau _2\right) + {\mathbb {E}}_{{\mathcal {I}}^+,p} \left[ \Psi ^{[-2]}\right] \left( \tau _1,\tau _2\right) \nonumber \\&\quad \lesssim \overline{{\mathbb {E}}}_{{\widetilde{\Sigma }}_{\tau },p} \left[ \Psi ^{[-2]}\right] \left( \tau _1\right) +{\mathbb {E}}_{{\widetilde{\Sigma }}_{\tau }} \left[ \uppsi ^{[-2]}\right] \left( \tau _1\right) + {\mathbb {E}}_{{\widetilde{\Sigma }}_{\tau }} \left[ \upalpha ^{[-2]}\right] \left( \tau _1\right) \nonumber \\&\qquad + \overline{{\mathbb {E}}}_{{\widetilde{\Sigma }}_{\tau },\eta } \left[ T\Psi ^{[-2]}\right] \left( \tau _1\right) +{\mathbb {E}}_{{\widetilde{\Sigma }}_{\tau }} \left[ T\uppsi ^{[-2]}\right] \left( \tau _1\right) + {\mathbb {E}}_{{\widetilde{\Sigma }}_{\tau }} \left[ T\upalpha ^{[-2]}\right] \left( \tau _1\right) \nonumber \\&\qquad + a\left( {\mathbb {E}}_{{\mathcal {I}}^+} \left[ \uppsi ^{[-2]}\right] \left( \tau _1,\tau _2\right) +{\mathbb {E}}_{{\mathcal {I}}^+} \left[ \upalpha ^{[-2]}\right] \left( \tau _1,\tau _2\right) \right) . \end{aligned}$$

##### Proof

The proof is exactly as in Proposition 11.2.1 except that we need to inspect carefully the error term ${\mathcal {J}}^{[-2]}$. (This is of course because the Regge–Wheeler operators are almost identical for $s=\pm \, 2$.) Checking the *r*-weights in the application of the Cauchy–Schwarz inequality, the analogous computation

$$\begin{aligned}&\int _{\widetilde{{\mathcal {R}}}(\tau _1,\tau _2)} \Big | {\mathcal {J}}^{[-2]}| |\xi | |\beta _4| |r^p L \Psi ^{[-2]}| \frac{r^2+a^2}{\Delta \rho ^2} dVol \\&\quad \lesssim \int _{\widetilde{{\mathcal {R}}}(\tau _1,\tau _2)} dVol \frac{r^2+a^2}{\Delta \rho ^2} \left( \lambda r^{p-1} |L\Psi ^{[-2]}|^2 + \frac{r^{p+1}}{\lambda } \Big | {\mathcal {J}}^{[-2]}|^2 \right) \\&\quad \lesssim \lambda \overline{{\mathbb {I}}}^{deg}_{p} \left[ \Psi ^{[-2]}\right] (\tau _1,\tau _2) + \frac{a^2}{\lambda }\left( {\mathbb {I}} \left[ \uppsi ^{[-2]}\right] \left( \tau _1,\tau _2\right) +{\mathbb {I}} \left[ \upalpha ^{[-2]}\right] \left( \tau _1,\tau _2\right) \right) \, \end{aligned}$$

is seen to be valid only for $p\in \left[ \eta ,2-\eta \right]$. For $p=2$ we need to integrate by parts. Note that the two worst (the others being controlled by the above estimate for $\lambda$ depending only on *M*) contributions from the error ${\mathcal {J}}^{[-2]}\beta _4 \xi r^p L \overline{\Psi }$ can be written (omitting taking real parts for the moment)

258 $$\begin{aligned}&ar^{-2} \Phi \left( \sqrt{\Delta } \psi ^{[-2]}\right) r^2 L \overline{\Psi ^{[-2]}} = L \left( a\Phi \left( \sqrt{\Delta } \psi ^{[-2]}\right) \overline{\Psi ^{[-2]}}\right) \nonumber \\&\quad + a\Delta \left( r^2+a^2\right) ^{-2} \left( \Phi \Psi ^{[-2]}\right) \overline{\Psi ^{[-2]}} \, , \end{aligned}$$

and

$$\begin{aligned}&a^2 r^{-2} \left( r^2+a^2\right) ^{-3/2} \upalpha ^{[-2]} \ r^2 L \overline{\Psi ^{[-2]}} = L \left( a^2\left( r^2+a^2\right) ^{-3/2} \upalpha ^{[-2]}\overline{\Psi ^{[-2]}}\right) \\&\quad - \frac{a^2\Delta }{ \left( r^2+a^2\right) ^{2}} \left( \sqrt{\Delta }\psi ^{[-2]} \right) \overline{\Psi ^{[-2]}} \, , \end{aligned}$$

where we have used the relations (52) and (51). Now upon taking real parts and integration, the second term in each line can be controlled by the basic Cauchy–Schwarz inequality, the integration by parts having gained a power in *r*. The first term in each line is a boundary term and controlled by the terms appearing on the right hand side of the estimate (256), where for the boundary term on null infinity we borrow from the term ${\mathbb {E}}_{{\mathcal {I}}^+,p} \left[ \Psi ^{[-2]}\right] \left( \tau _1,\tau _2\right)$ appearing on the left hand side. $\square$

#### Physical Space Weighted Transport for ${\uppsi }^{[-2]}$ and $\Psi ^{[-2]}$

##### Proposition 11.3.2

Under the assumptions of Theorem 11.1 we have for any $\tau _2 > \tau _1\ge 0$ the estimate

259 $$\begin{aligned}&{\mathbb {E}}_{{\widetilde{\Sigma }}_{\tau }} \left[ \upalpha ^{[-2]}\right] \left( \tau _2\right) + {\mathbb {I}} \left[ \upalpha ^{[-2]}\right] \left( \tau _1,\tau _2\right) + {\mathbb {E}}_{{\mathcal {I}}^+} \left[ \upalpha ^{[-2]}\right] \left( \tau _1,\tau _2\right) \nonumber \\&\quad \lesssim {\mathbb {I}} \left[ \uppsi ^{[-2]}\right] \left( \tau _1,\tau _2\right) + {\mathbb {E}}_{{\widetilde{\Sigma }}_{\tau }} \left[ \upalpha ^{[-2]}\right] \left( \tau _1\right) \end{aligned}$$

and the estimate

260 $$\begin{aligned}&{\mathbb {E}}_{{\widetilde{\Sigma }}_{\tau }} \left[ \uppsi ^{[-2]}\right] \left( \tau _2\right) + {\mathbb {I}} \left[ \uppsi ^{[-2]}\right] \left( \tau _1,\tau _2\right) +{\mathbb {E}}_{{\mathcal {I}}^+} \left[ \uppsi ^{[-2]}\right] \left( \tau _1,\tau _2\right) \nonumber \\&\quad \lesssim {\mathbb {I}}_{\eta }^{\mathrm{deg}} \left[ \Psi ^{[-2]}\right] \left( \tau _1,\tau _2\right) + {\mathbb {E}}_{{\widetilde{\Sigma }}_{\tau }} \left[ \uppsi ^{[-2]}\right] \left( \tau _1\right) \nonumber \\&\qquad + {\mathbb {E}}_{{\widetilde{\Sigma }}_{\tau },\eta } \left[ \Psi ^{[-2]}\right] \left( \tau _1\right) + {\mathbb {E}}_{{\widetilde{\Sigma }}_{\tau }} \left[ \upalpha ^{[-2]}\right] \left( \tau _1\right) \, . \end{aligned}$$

The same estimates hold with an overbar on all terms.

##### Proof

Multiply (119) by a cut-off function $\xi$ which is equal to 1 for $r\ge 9M$ and equal to zero for $r\le 8M$. Bringing $\xi$ inside the first bracket produces an error-term supported in $\left[ 8M,9M\right]$, which (upon integration) is for any *n* controlled by the basic estimate (239). Upon integration of the resulting identity we deduce (260) for $\Gamma$ being the identity in the energies appearing. Now we observe that the same estimate holds for the *T* and $\Phi$ commuted equations (note that (117) commutes trivially with the Killing fields *T* and $\Phi$ so the estimate (111) trivially holds for the commuted variables).

The estimate (259) is proven completely analogously except that here no cut-off is required in view of the non-degenerate norm of $\uppsi ^{[-2]}$ appearing on the right hand side: One first applies (118) and the same estimate for the *T* and $\Phi$ commuted variables.

To obtain the estimates with an overbar one first commutes (117) and (116) with ${\underline{L}}$ and notes that the analogue of (119) and (118) can now be applied with the error from the commutator $\left[ L, {\underline{L}}\right] \sim \frac{a}{r^3} \Phi$ being controlled by the previous step. Secondly, one commutes (117) and (116) with the vectorfield $\frac{r^2+a^2}{\Delta } {\underline{L}}$ which extends regularly to the horizon and observes that the additional commutator term leads to a good sign (near the horizon) in the estimates (119) and (118). $\square$

#### Completing the Proof of Theorem 11.1 for $s=-2$

Combining the estimate of Proposition 11.3.1 with that of Proposition 11.3.2 we deduce for $p \in \{\eta , 1, 2\}$ (first for $K=0$ and then by trivial commutation with the Killing field *T* for any $K \in {\mathbb {N}}$) the estimate

261 $$\begin{aligned}&\sum _{k=0}^K \left( {\mathbb {E}}_{{\widetilde{\Sigma }}_{\tau }} \left[ T^k\upalpha ^{[-2]}\right] \left( \tau _2\right) + {\mathbb {E}}_{{\widetilde{\Sigma }}_{\tau }} \left[ T^k \uppsi ^{[-2]}\right] \left( \tau _2\right) + \overline{{\mathbb {E}}}_{{\widetilde{\Sigma }}_{\tau },p} \left[ T^k\Psi ^{[-2]}\right] \left( \tau _2\right) \right) \nonumber \\&\qquad +\sum _{k=0}^K \left( {\mathbb {I}} \left[ T^k\upalpha ^{[-2]}\right] \left( \tau _1,\tau _2\right) +{\mathbb {I}} \left[ T^k\uppsi ^{[-2]}\right] \left( \tau _1,\tau _2\right) + \overline{{\mathbb {I}}}^{\mathrm{deg}}_{p} \left[ T^k\Psi ^{[-2]}\right] \left( \tau _1,\tau _2\right) \right) \nonumber \\&\quad \lesssim \sum _{k=0}^K \left( {\mathbb {E}}_{{\widetilde{\Sigma }}_{\tau }} \left[ T^k\upalpha ^{[-2]}\right] \left( \tau _1\right) + {\mathbb {E}}_{{\widetilde{\Sigma }}_{\tau }} \left[ T^k \uppsi ^{[-2]}\right] \left( \tau _1\right) + \overline{{\mathbb {E}}}_{{\widetilde{\Sigma }}_{\tau },p} \left[ T^k\Psi ^{[-2]}\right] \left( \tau _1\right) \right) \end{aligned}$$

and also

262 $$\begin{aligned}&\sum _{k=0}^K \left( {\mathbb {E}}_{{\widetilde{\Sigma }}_{\tau }} \left[ T^k\upalpha ^{[-2]}\right] \left( \tau _2\right) + {\mathbb {E}}_{{\widetilde{\Sigma }}_{\tau }} \left[ T^k \uppsi ^{[-2]}\right] \left( \tau _2\right) + \overline{{\mathbb {E}}}_{{\widetilde{\Sigma }}_{\tau },p} \left[ T^k\Psi ^{[-2]}\right] \left( \tau _2\right) \right) \nonumber \\&\qquad +\sum _{k=0}^K \left( {\mathbb {I}} \left[ T^k\upalpha ^{[-2]}\right] \left( \tau _1,\tau _2\right) +{\mathbb {I}} \left[ T^k\uppsi ^{[-2]}\right] \left( \tau _1,\tau _2\right) + \overline{{\mathbb {I}}}_{p} \left[ T^k\Psi ^{[-2]}\right] \left( \tau _1,\tau _2\right) \right) \nonumber \\&\quad \lesssim \sum _{k=0}^K \left( {\mathbb {E}}_{{\widetilde{\Sigma }}_{\tau }} \left[ T^k\upalpha ^{[-2]}\right] \left( \tau _1\right) + {\mathbb {E}}_{{\widetilde{\Sigma }}_{\tau }} \left[ T^k \uppsi ^{[-2]}\right] \left( \tau _1\right) + \overline{{\mathbb {E}}}_{{\widetilde{\Sigma }}_{\tau },p} \left[ T^k\Psi ^{[-2]}\right] \left( \tau _1\right) \right) \nonumber \\&\qquad + {\mathbb {E}}_{{\widetilde{\Sigma }}_{\tau }} \left[ T^{K+1}\upalpha ^{[-2]}\right] \left( \tau _1\right) + {\mathbb {E}}_{{\widetilde{\Sigma }}_{\tau }} \left[ T^{K+1} \uppsi ^{[-2]}\right] \left( \tau _1\right) + \overline{{\mathbb {E}}}_{{\widetilde{\Sigma }}_{\tau },\eta } \left[ T^{K+1}\Psi ^{[-2]}\right] \left( \tau _1\right) . \end{aligned}$$

Let us denote the right hand side of the second estimate on the initial data slice ${\widetilde{\Sigma }}_0$ (i.e. for $\tau _1=\tau _0$) by ${\mathbb {D}}_{K+1, p} \left[ \Psi ^{[-2]}, \uppsi ^{[-2]}, \upalpha ^{[-2]}\right] \left( \tau _0\right)$.

Applying the estimate (262) for $K=1$ and $p=2$ implies (after using a standard argument involving dyadic sequences) along a dyadic sequence $\tau _n \sim 2^{n}\tau _0$ the estimate

$$\begin{aligned}&\sum _{k=0}^1 \left( {\mathbb {E}}_{{\widetilde{\Sigma }}_{\tau }} \left[ T^k \upalpha ^{[-2]}\right] \left( \tau _n\right) + {\mathbb {E}}_{{\widetilde{\Sigma }}_{\tau }} \left[ T^k \uppsi ^{[-2]}\right] \left( \tau _n\right) + \overline{{\mathbb {E}}}_{{\widetilde{\Sigma }}_{\tau },1} \left[ T^k\Psi ^{[-2]}\right] \left( \tau _n\right) \right) \\&\quad \lesssim \frac{{\mathbb {D}}_{2, 2} \left[ \Psi ^{[-2]}, \uppsi ^{[-2]}, \upalpha ^{[-2]}\right] \left( \tau _0\right) }{\tau _n} \, . \end{aligned}$$

Using the above and applying (261) for $p=1$, $K=1$ between the time $\tau _1=\tau _n$ and any $\tau _2 \in \left( \tau _n, \tau _{n+1}\right]$ yields the previous estimate for any $\tau$, not only the members of the dyadic sequence. Turning back to (262) now with $K=0$ we use the previous estimate and a similar dyadic argument to produce along a dyadic sequence the estimate

$$\begin{aligned}&{\mathbb {E}}_{{\widetilde{\Sigma }}_{\tau }} \left[ \upalpha ^{[-2]}\right] \left( \tau _n\right) + {\mathbb {E}}_{{\widetilde{\Sigma }}_{\tau }} \left[ \uppsi ^{[-2]}\right] \left( \tau _n\right) + \overline{{\mathbb {E}}}_{{\widetilde{\Sigma }}_{\tau },\eta } \left[ \Psi ^{[-2]}\right] \left( \tau _n\right) \\&\quad \lesssim \frac{{\mathbb {D}}_{2, 2} \left[ \Psi ^{[-2]}, \uppsi ^{[-2]}, \upalpha ^{[-2]}\right] \left( \tau _0\right) }{\tau _n^{2-\eta }} \, . \end{aligned}$$

Using the above and applying (261) with $p=\eta$ and $K=0$ now yields (247) of Theorem 11.1. To obtain the second estimate, one simply repeats the above proof using that the estimate of Proposition 11.3.2 also holds for the energies with an overbar.

## Derivation of the Equation for $\Psi ^{[s]}$

### The Teukolsky Equation

Recall from (13) the definition $w = \frac{\Delta }{\left( r^2+a^2\right) ^2}$. Using (39) the inhomogeneous Teukolsky equations (53) can be written in physical space as

263

and

respectively. Introducing the physical space operators

264

265

as well as recalling the definitions

266 $$\begin{aligned} {\underline{L}} \tilde{\upalpha }^{[+2]}&= - 2w \sqrt{\Delta }\uppsi ^{[+2]} \ \ \ , \ \ \ {\underline{L}} \left( \sqrt{\Delta } \uppsi ^{[+2]}\right) = w \Psi ^{[+2]} \, , \end{aligned}$$

267 $$\begin{aligned} {L} \left( \Delta ^2\tilde{\upalpha }^{[-2]} \right)&= 2w \sqrt{\Delta }\uppsi ^{[-2]} \ \ \ , \ \ \ {L} \left( \sqrt{\Delta } \uppsi ^{[-2]}\right) = -w \Psi ^{[-2]} \, , \end{aligned}$$

we can write (263) as

268 $$\begin{aligned}&2 L \left( \sqrt{\Delta }\uppsi ^{[+2]}\right) - 2\sqrt{\Delta }\uppsi ^{[+2]} \frac{w^\prime }{w} = {\mathcal {L}}^{[+2]}\tilde{\upalpha }^{[+2]} + {\mathcal {Q}}^{[+2]} \tilde{\upalpha }^{[+2]} - \frac{\Delta }{w\rho ^2} F^{[+2]} \, , \, \end{aligned}$$

269 $$\begin{aligned}&-2 {\underline{L}} \left( \sqrt{\Delta }{\uppsi }^{[-2]}\right) - 2\sqrt{\Delta }{\uppsi }^{[-2]} \frac{w^\prime }{w} = {\mathcal {L}}^{[-2]} \left( \Delta ^2 \tilde{\upalpha }^{[-2]} \right) \nonumber \\&\quad +{\mathcal {Q}}^{[-2]} \left( \Delta ^2 \tilde{\upalpha }^{[-2]} \right) - \frac{\Delta ^3}{w\rho ^2} F^{[-2]} \, . \end{aligned}$$

For the *separated* form of the Teukolsky equation, using the relation

$$\begin{aligned}&\frac{w^{\prime \prime }}{w} - 2 \frac{\left( w^\prime \right) ^2}{w^2}-V_0^{[+2]} -2w= \frac{w^{\prime \prime }}{w} - 2 \frac{\left( w^\prime \right) ^2}{w^2}-V_0^{[-2]} +2w \\&\quad = -3w \frac{a^4+a^2r^2-2Mr^3}{(r^2+a^2)^2} +2w \, , \end{aligned}$$

it is easy to see that we can write (153) for spin $s=+2$ and $s=-2$ as^13^

270 $$\begin{aligned}&2 L \left( \sqrt{\Delta }\psi ^{[+2]}\right) - 2\sqrt{\Delta }\psi ^{[+2]} \frac{w^\prime }{w} = \left( \Lambda _{m \ell }^{[+2],(a\omega )} + 2\right) \left( u^{[+2]}w\right) \nonumber \\&\quad + Q^{[+2]} \left( u^{[+2]}w\right) - \frac{\Delta }{w\rho ^2} F_{m \ell }^{[+2],(a\omega )} \, , \end{aligned}$$

271 $$\begin{aligned}&-2 {\underline{L}} \left( \sqrt{\Delta }{\psi }^{[-2]}\right) - 2\sqrt{\Delta }{\psi }^{[-2]} \frac{w^\prime }{w} = \left( \Lambda _{m \ell }^{[-2],(a\omega )} - 2\right) \left( {u}^{[-2]}w\right) \nonumber \\&\quad +Q^{[-2]} \left( u^{[-2]}w\right) - \frac{\Delta ^3}{w\rho ^2} F_{m \ell }^{[-2],(a\omega )} \, , \end{aligned}$$

where

$$\begin{aligned} Q^{[\pm 2]} = \mp 6 \frac{r}{r^2+a^2} iam -3 \frac{a^4+a^2r^2-2Mr^3}{(r^2+a^2)^2} +2 \, . \end{aligned}$$

We observe that $Q^{[\pm 2]}$ is the separated analogue of the physical space operators ${\mathcal {Q}}^{[\pm 2]}$ defined in (264). Similarly, the separated analogue of the operator ${\mathcal {L}}^{[\pm 2]}$ is easily seen to be the operator  which has eigenvalues $\Lambda _{m\ell }^{[\pm 2],(aw)} \pm 2$, see (135), (136) and (149). Note the symmetry between the physical space formulation (268), (269) and the separated form (270), (271).

### Derivation of the $\Psi ^{[s]}$ Equation in Physical Space

We derive the equation for $s=+2$ in physical space. Observing the commutation relation

$$\begin{aligned} \left[ {\underline{L}},L\right] = \frac{4ra}{r^2+a^2} w \cdot \Phi \,, \end{aligned}$$

we obtain after applying ${\underline{L}}$ to the Teukolsky equation (268) recalling (266) and that ${\mathcal {L}}^{[+2]}$ commutes with ${\underline{L}}$:

272 $$\begin{aligned}&2 L \left( w \Psi ^{[+2]}\right) + \frac{8ra}{r^2+a^2} w \cdot \Phi \left( \sqrt{\Delta } \uppsi ^{[+2]}\right) - 2w^\prime \Psi ^{[+2]} + 2\left( \sqrt{\Delta } \uppsi ^{[+2]}\right) \left( \frac{w^\prime }{w}\right) ^\prime \nonumber \\&\quad = -2w {\mathcal {L}}^{[+2]} \left( \sqrt{\Delta } \uppsi ^{[+2]}\right) -2w {\mathcal {Q}}^{[+2]} \left( \sqrt{\Delta }\uppsi ^{[+2]}\right) \nonumber \\&\qquad + \left[ {\underline{L}}, {\mathcal {Q}}^{[+2]}\right] \left( \tilde{\upalpha }^{[+2]} \right) -{\underline{L}} \left( \frac{\Delta }{w\rho ^2} F^{[+2]}\right) \, , \end{aligned}$$

which we immediately simplify to

273 $$\begin{aligned}&L \left( \Psi ^{[+2]}\right) + \frac{4ra}{r^2+a^2} \cdot \Phi \left( \sqrt{\Delta } \uppsi ^{[+2]}\right) + \left( \sqrt{\Delta } \uppsi ^{[+2]}\right) \frac{1}{w} \left( \frac{w^\prime }{w}\right) ^\prime \nonumber \\&\quad = - {\mathcal {L}}^{[+2]} \left( \sqrt{\Delta } \uppsi ^{[+2]}\right) - {\mathcal {Q}}^{[+2]} \left( \sqrt{\Delta }\uppsi ^{[+2]}\right) + \frac{1}{2w} \left[ {\underline{L}} , {\mathcal {Q}}^{[+2]}\right] \tilde{\upalpha }^{[+2]} \nonumber \\&\qquad - \frac{1}{2w}{\underline{L}} \left( \frac{\Delta }{w\rho ^2} F^{[+2]}\right) \, . \end{aligned}$$

We now apply another ${\underline{L}}$ derivative, which produces

274 $$\begin{aligned}&L {\underline{L}} \left( \Psi ^{[+2]}\right) +w \frac{8ra}{r^2+a^2} \cdot \Phi \Psi ^{[+2]} + w Q^{[+2]} \Psi ^{[+2]} + \left( \frac{w^\prime }{w}\right) ^\prime \Psi ^{[+2]} +w {\mathcal {L}}^{[+2]} \Psi ^{[+2]} \nonumber \\&\quad = \left( 4a \left( \frac{r}{r^2+a^2}\right) ^\prime \Phi + \left( \frac{1}{w} \left( \frac{w^\prime }{w}\right) ^\prime \right) ^\prime - 2 \left[ {\underline{L}},{\mathcal {Q}}^{[+2]}\right] \right) \left( \sqrt{\Delta }\uppsi ^{[+2]}\right) \nonumber \\&\qquad + \left[ {\underline{L}}, \frac{1}{2w} \left[ {\underline{L}} , {\mathcal {Q}}^{[+2]}\right] \right] \tilde{\upalpha }^{[+2]} -\frac{1}{2}{\underline{L}} \left( \frac{1}{w} {\underline{L}} \left( \frac{\Delta }{w\rho ^2} F^{[+2]}\right) \right) \, . \end{aligned}$$

Using elementary algebra we simplify the terms in the first line of (274) to

275 $$\begin{aligned} L {\underline{L}} \left( \Psi ^{[+2]}\right) +w \frac{2ra}{r^2+a^2} \cdot \Phi \Psi ^{[+2]} + w \left( {\mathcal {L}}^{[+2]} +4 - \frac{6M}{r} \frac{r^2-a^2}{r^2+a^2} -7a^2 w\right) \Psi ^{[+2]} \, , \end{aligned}$$

which can be written more succinctly as

276 $$\begin{aligned} \frac{1}{2} \left( L {\underline{L}} + {\underline{L}} L \right) \left( \Psi ^{[+2]}\right) + w \left( {\mathcal {L}}^{[+2]} +4 - \frac{6M}{r} \frac{r^2-a^2}{r^2+a^2} -7a^2 w\right) \Psi ^{[+2]} \, . \end{aligned}$$

The terms in the second line of (274) simplify to

277 $$\begin{aligned} w \left[ -8a \cdot \frac{-r^2+a^2}{r^2+a^2} \Phi + 20a^2 \frac{r^3-3Mr^2+ra^2+Ma^2}{\left( r^2+a^2\right) ^2}\right] \sqrt{\Delta }\uppsi ^{[+2]} \, . \end{aligned}$$

Finally, for the double commutator term in the third line of (274) we obtain

278 $$\begin{aligned} \left[ +12a^3 w \frac{r}{r^2+a^2} \Phi -3a^2 w \frac{r^4 -a^4+10Mr^3-6Ma^2r}{(r^2+a^2)^2} \right] \tilde{\upalpha }^{[+2]} \, . \end{aligned}$$

Combining the previous equations we have therefore established the formula of Proposition 3.2.1 for $s=+2$. The formula for $s=-2$ is proven entirely analogously exchanging the roles of ${\underline{L}}$ and *L*. For completeness, we nevertheless explicitly derive the (equivalent) separated form of the equation for $s=-2$ in the next section.

### Derivation of the $\Psi ^{[s]}$ Equation in Separated Form

As mentioned at the end of the previous section and mainly to illustrate the equivalence between deriving equations in the physical space and the separated picture, we now derive the equation for $\Psi ^{[-2]}$ in *separated* form from (271). Recall that ${\underline{L}}$ and *L* are now given by the *separated* frame operators (155) and (154). Observing the commutation relation

$$\begin{aligned} \left[ {\underline{L}},L\right] = \frac{4ra}{r^2+a^2} w \cdot i m \, , \end{aligned}$$

we obtain after applying *L* to the separated Teukolsky equation (271) recalling the separated relations (158) and (158)

$$\begin{aligned}&+2 {\underline{L}} \left( w \Psi ^{[-2]}\right) + \frac{8ra}{r^2+a^2} w \cdot im\left( \sqrt{\Delta } \psi ^{[-2]}\right) + 2w^\prime \Psi ^{[-2]} - 2\left( \sqrt{\Delta } \psi ^{[-2]}\right) \left( \frac{w^\prime }{w}\right) ^\prime \\&\quad = 2w \left( \Lambda _{m \ell }^{[-2],(a\omega )} - 2\right) \left( \sqrt{\Delta } \psi ^{[-2]}\right) +2w Q^{[-2]} \left( \sqrt{\Delta }\psi ^{[-2]}\right) \\&\qquad + \left( \partial _{r^*} Q^{[-2]}\right) \left( u^{[-2]}w\right) - L \left( \frac{\Delta ^3}{w\rho ^2} F^{[-2],(a\omega )}_{m \ell }\right) \, , \end{aligned}$$

which we immediately simplify to

$$\begin{aligned}&{\underline{L}} \left( \Psi ^{[-2]}\right) + \frac{4ra}{r^2+a^2} \cdot im\left( \sqrt{\Delta } \psi ^{[-2]}\right) - \left( \sqrt{\Delta } \psi ^{[-2]}\right) \frac{1}{w} \left( \frac{w^\prime }{w}\right) ^\prime \\&\quad = \left( \Lambda _{m \ell }^{[-2],(a\omega )} - 2\right) \left( \sqrt{\Delta } \psi ^{[-2]}\right) + Q^{[-2]} \left( \sqrt{\Delta }\psi ^{[-2]}\right) \\&\qquad +\frac{1}{2w}\left( Q^{[-2]}\right) ^\prime \left( u^{[-2]}w\right) - \frac{1}{2w}L \left( \frac{\Delta ^3}{w\rho ^2} F^{[-2],(a\omega )}_{m \ell }\right) . \end{aligned}$$

We now apply another *L* derivative, which produces

279 $$\begin{aligned}&L {\underline{L}} \left( \Psi ^{[-2]}\right) -w \frac{4ra}{r^2+a^2} \cdot im \Psi ^{[-2]} + w Q^{[-2]} \Psi ^{[-2]} \nonumber \\&\qquad + \left( \frac{w^\prime }{w}\right) ^\prime \Psi ^{[-2]} +w \left( \Lambda _{m \ell }^{[-2],(a\omega )} - 2\right) \Psi ^{[-2]}\nonumber \\&\quad = \left[ -4aim \left( \frac{r}{r^2+a^2}\right) ^\prime + \left( \frac{1}{w} \left( \frac{w^\prime }{w}\right) ^\prime \right) ^\prime + 2\left( Q^{[+2]}\right) ^\prime \right] \left( \sqrt{\Delta }\psi ^{[-2]}\right) \nonumber \\&\qquad + \left[ \left( \frac{1}{2w} \left( Q^{[+2]}\right) ^\prime \right) ^\prime \right] \left( u^{[-2]}w\right) - \frac{1}{2} L \left( \frac{1}{w}L \left( \frac{\Delta ^3}{w\rho ^2} F^{[-2],(a\omega )}_{m \ell }\right) \right) \, . \end{aligned}$$

Using elementary algebra we simplify the terms in the first line of (279) to

280 $$\begin{aligned}&L {\underline{L}} \left( \Psi ^{[-2]}\right) +w \frac{2ra}{r^2+a^2} \cdot im \Psi ^{[-2]} \nonumber \\&\quad + w \left( \Lambda _{m \ell }^{[-2],(a\omega )} +2 - \frac{6M}{r} \frac{r^2-a^2}{r^2+a^2} -7a^2 w\right) \Psi ^{[-2]} \,, \end{aligned}$$

which can be written more succinctly as

281 $$\begin{aligned} \frac{1}{2} \left( L {\underline{L}} + {\underline{L}} L \right) \left( \Psi ^{[-2]}\right) + w \left( \Lambda _{m \ell }^{[-2],(a\omega )} +2 - \frac{6M}{r} \frac{r^2-a^2}{r^2+a^2} -7a^2 w\right) \Psi ^{[-2]} \, . \end{aligned}$$

The terms in the second line simplify to

282 $$\begin{aligned} w \left[ +8aim \cdot \frac{-r^2+a^2}{r^2+a^2} + 20a^2 \frac{r^3-3Mr^2+ra^2+Ma^2}{\left( r^2+a^2\right) ^2}\right] \sqrt{\Delta }\psi ^{[-2]} \, . \end{aligned}$$

Finally, for the third line of (274) excluding the inhomogeneous term we obtain

283 $$\begin{aligned} \left[ -12a^3 w \frac{r}{r^2+a^2} im -3a^2 w \frac{r^4 -a^4+10Mr^3-6Ma^2r}{(r^2+a^2)^2} \right] \left( u^{[-2]} w\right) \,. \end{aligned}$$

In summary, we have established the following formula for $s=-2$:

284 $$\begin{aligned}&-\frac{1}{2} \left( L {\underline{L}}+ {\underline{L}} L\right) \Psi ^{[s]} - \frac{\Delta }{\left( r^2+a^2\right) ^2} \left( \lambda _{m \ell }^{[s]} -2am\omega + a^2 \omega ^2 +s^2 +s \right) \Psi ^{[s]} \nonumber \\&\quad + \frac{\Delta }{\left( r^2+a^2\right) ^2} \frac{6Mr}{r^2+a^2} \frac{r^2-a^2}{r^2+a^2}\Psi ^{[s]} +7a^2 \frac{\Delta ^2}{(r^2+a^2)^4} \Psi ^{[s]} = \frac{\Delta }{\rho ^2}{\mathcal {J}}^{[s]} + {\mathfrak {G}}^{[s]} \, , \end{aligned}$$

where the right hand is side given by

285 $$\begin{aligned}&{\mathcal {J}}^{[s]} = \frac{\rho ^2}{\left( r^2+a^2\right) ^2} \left[ -4s\frac{r^2-a^2}{r^2+a^2} aim -20a^2 \frac{r^3-3Mr^2+ra^2+Ma^2}{\left( r^2+a^2\right) ^2}\right] \left( \sqrt{\Delta }\psi ^{[s]} \right) \nonumber \\&\quad +a^2 \frac{\rho ^2}{\left( r^2+a^2\right) ^2} \left[ -6s \frac{r}{r^2+a^2} aim +3 \left( \frac{r^4 -a^4+10Mr^3-6Ma^2r}{(r^2+a^2)^2}\right) \right] \left( u^{[s]}w\right) \, , \end{aligned}$$

286 $$\begin{aligned}&{\mathfrak {G}}^{[+2]}= \frac{1}{2} {\underline{L}} \left( \frac{1}{w}{\underline{L}} \left( \frac{\Delta }{w\rho ^2} F^{[+2]}\right) \right) \ \ \ , \ \ \ {\mathfrak {G}}^{[-2]}= \frac{1}{2} L \left( \frac{1}{w}L \left( \frac{\Delta ^3}{w\rho ^2} F^{[-2]}\right) \right) \, . \end{aligned}$$

We have therefore proven Proposition 7.3.1 for $s=-2$. The $s=+2$ case is proven entirely analogously or can be easily deduced directly from the physical space formula of Proposition 3.2.1.

Finally, note that we can write (284) also as

$$\begin{aligned} \left( {\Psi }^{[s]}\right) ^{\prime \prime } + \left( \omega ^2 - {\mathcal {V}}^{[+2]} \right) {\Psi }^{[s]} = \frac{\Delta }{\rho ^2}{\mathcal {J}}^{[s]} +{\mathfrak {G}}^{[s]}\, \end{aligned}$$

for the potential

287 $$\begin{aligned} {\mathcal {V}}^{[s]}&=\frac{ \Delta \left( \lambda ^{[s]}_{m \ell } +a^2\omega ^2+s^2+s\right) + 4Mram\omega -a^2 m^2 }{\left( r^2+a^2\right) ^2}\nonumber \\&\quad -\frac{\Delta }{(r^2+a^2)^2}\frac{6Mr (r^2-a^2)}{(r^2+a^2)^2} -7 a^2 \frac{\Delta ^2}{(r^2+a^2)^4} \nonumber \\&= {\mathcal {V}}^{[s]}_0 + {\mathcal {V}}^{[s]}_1 + {\mathcal {V}}^{[s]}_2 \, . \end{aligned}$$

## Auxilliary Calculations for Physical Space Multipliers

We first recall the relations

$$\begin{aligned} L + {\underline{L}} = 2T +2\frac{a}{r^2+a^2} \Phi \, \ \ , \ \ L-{\underline{L}} = 2 \partial _{r^*} \, . \end{aligned}$$

We will consider the identities generated by the following four multipliers (the smooth radial cut-offs $\chi$, $\xi$ and the smooth radial functions *f*, *h*, *y* are chosen appropriately in the body of the paper)

1. The *T*-energy: $$T{\overline{\Psi }}$$
2. The Lagragian multiplier: $h {\overline{\Psi }}$
3. The $\Phi$-multiplier: $\omega _+\chi \Phi {\overline{\Psi }}$ ($\chi$ a radial cut-off)
4. The *y*-multiplier: $$f \left( L-{\underline{L}}\right) {\overline{\Psi }}$$
5. The redshift multiplier: $\frac{1}{w}\xi {\underline{L}} {\overline{\Psi }}$ ($\xi$ a radial cut-off near the horizon)
6. The $r^p$ weighed multiplier: $r^p \beta _k \xi L\overline{\Psi }$ with $\beta _k=1+k\frac{M}{r}$ ($\xi$ a radial cut-off near infinity)

each acting on the second order terms in the equation (54), namely (recall $w=\frac{\Delta }{\left( r^2+a^2\right) ^2}$)

I. $$\frac{1}{2} \left( L {\underline{L}} + {\underline{L}} L \right) \Psi $$
III. $$ w 2 a T \Phi \Psi $$
IV. $$ w a^2 \sin ^2\theta TT\Psi $$

The point is that the $0^{th}$ order terms in (54) are easy to handle while for the (only) first order term in (54), $2is w a \cos \theta T\Psi$, we observe that for *X* any real vectorfield commuting with *T* we have

288 $$\begin{aligned}&2is w a \cos \theta T\Psi X \overline{\Psi } = T \left( 2is w a \cos \theta \Psi X \overline{\Psi }\right) - X \left( 2is w a \cos \theta \Psi T \overline{\Psi } \right) \nonumber \\&\quad + X \left( 2isw a \cos \theta \right) \Psi T \overline{\Psi } \nonumber \\&\quad +2is w a \cos \theta X\Psi T\overline{\Psi } \, , \end{aligned}$$

and hence

289 $$\begin{aligned}&\text {Re} \left( 2is wa \cos \theta T\Psi X\overline{\Psi } \right) = T \left( iswa \cos \theta \Psi X \overline{\Psi } \right) - X \left( is w a \cos \theta \Psi T \overline{\Psi } \right) \nonumber \\&\qquad + X \left( isw a \cos \theta \right) \Psi T \overline{\Psi } \, \nonumber \\&\quad = -T \left( swa \cos \theta \text {Im} \Psi X \overline{\Psi } \right) + X \left( s w a \cos \theta \text {Im} \Psi T \overline{\Psi } \right) - X \left( sw a \cos \theta \right) \text {Im} \Psi T \overline{\Psi }. \end{aligned}$$

In particular for $X=T$ the right hand side is zero while for $X=\omega _+ \chi \Phi$ only the first two terms survive (and only the first after integration in $\phi$).

### The *T*-multiplier: $T{\overline{\Psi }}$

#### Part I: $\frac{1}{2} \left( L {\underline{L}} + {\underline{L}} L \right) \Psi$

290 $$\begin{aligned}&\frac{1}{2}\mathrm{Re} \left( L {\underline{L}} + {\underline{L}} L \right) \Psi \, T {\overline{\Psi }}= \frac{1}{4} \mathrm{Re}\left( L+{\underline{L}}\right) \left( L + {\underline{L}}\right) \Psi T {\overline{\Psi }}- \frac{1}{4} \mathrm{Re}\left( L-{\underline{L}}\right) \left( L - {\underline{L}}\right) \Psi T {\overline{\Psi }}\nonumber \\&\quad = \frac{1}{4} \mathrm{Re}\left( L + {\underline{L}}\right) \Big \{ \left( L + {\underline{L}}\right) \Psi T {\overline{\Psi }}\Big \} - \frac{1}{8} T \Big \{ |\left( L+{\underline{L}}\right) \Psi |^2 \Big \} \nonumber \\&\qquad - \frac{1}{4} \mathrm{Re}\left( L - {\underline{L}}\right) \Big \{ \left( L - {\underline{L}}\right) \Psi T {\overline{\Psi }}\Big \} + \frac{1}{8} T \Big \{ |\left( L-{\underline{L}}\right) \Psi |^2 \Big \} \end{aligned}$$

which we write as

291 $$\begin{aligned}&\frac{1}{2} \text {Re} \left( L {\underline{L}} + {\underline{L}} L \right) \Psi T \overline{\Psi } = \frac{1}{16} \left( L+{\underline{L}}\right) \nonumber \\&\quad \times \Big \{ |\left( L+{\underline{L}}\right) \Psi |^2+ |\left( L-{\underline{L}}\right) \Psi |^2 -\frac{4a}{r^2+a^2}\mathrm{Re} \Phi {\overline{\Psi }}\left( L+{\underline{L}}\right) \Psi \Big \} \nonumber \\&\quad \ \ +\frac{1}{8} \Phi \Big \{ \frac{a}{r^2+a^2} \left( |\left( L+{\underline{L}}\right) \Psi |^2-|\left( L-{\underline{L}}\right) \Psi |^2\right) \Big \} \nonumber \\&\quad \ \ - \frac{1}{4} \left( L - {\underline{L}}\right) \mathrm{Re} \Big \{ \left( L - {\underline{L}}\right) \Psi T {\overline{\Psi }}\Big \} \end{aligned}$$

#### Part II: (After Integration Over $\int \sin \theta d\theta d\phi$, See (33))

292

#### Part III: $w 2 a T \Phi \Psi$

293 $$\begin{aligned} w 2 a \,\mathrm{Re}\, T \Phi \Psi T {\overline{\Psi }}= \Phi \Big \{ aw |T\Psi |^2\Big \} \end{aligned}$$

#### Part IV: $w a^2 \sin ^2\theta TT\Psi$

294 $$\begin{aligned} w a^2 \sin ^2\theta \mathrm{Re}\, TT\Psi T {\overline{\Psi }}= \frac{1}{2} T \Big \{ wa^2 \sin ^2\theta |T\Psi |^2 \Big \} \end{aligned}$$

### The Lagrangian Term: $h {\overline{\Psi }}$

#### Part I: $\frac{1}{2} \left( L {\underline{L}} + {\underline{L}} L \right) \Psi$

295 $$\begin{aligned}&\frac{1}{2} \left( L {\underline{L}} + {\underline{L}} L \right) \mathrm{Re} \Psi h {\overline{\Psi }}= \frac{1}{4} \left( L+{\underline{L}}\right) \left( L + {\underline{L}}\right) \mathrm{Re}\Psi h {\overline{\Psi }}- \frac{1}{4} \left( L-{\underline{L}}\right) \left( L - {\underline{L}}\right) \mathrm{Re}\Psi h {\overline{\Psi }}\nonumber \\&\quad = \frac{1}{4} \left( L+{\underline{L}} \right) \mathrm{Re}\Big \{ \left( L + {\underline{L}}\right) \Psi h {\overline{\Psi }}\Big \} - \frac{1}{4} \left( L-{\underline{L}} \right) \mathrm{Re}\Big \{ \left( L - {\underline{L}}\right) \Psi h {\overline{\Psi }}\Big \} \nonumber \\&\qquad +\frac{1}{4} h \Big [ |(L-{\underline{L}})\Psi |^2 - |(L+{\underline{L}})\Psi |^2 \Big ] + \frac{1}{4}h^\prime \left( L-{\underline{L}}\right) |\Psi |^2 \nonumber \\&\quad = \frac{1}{4} \left( L+{\underline{L}} \right) \mathrm{Re}\Big \{ \left( L + {\underline{L}}\right) \Psi h \Psi \Big \} - \frac{1}{4} \left( L-{\underline{L}} \right) \Big \{ \left( L - {\underline{L}}\right) \mathrm{Re}\Psi h \Psi - h^\prime |\Psi |^2 \Big \} \nonumber \\&\qquad +\frac{1}{4} h \Big [ |(L-{\underline{L}})\Psi |^2 - |(L+{\underline{L}})\Psi |^2 \Big ] - \frac{1}{2}h^{\prime \prime } |\Psi |^2 \end{aligned}$$

#### Part II: (After Integration Over $\int \sin \theta d\theta d\phi$)

296

#### Part III: $w 2 a T \Phi \Psi$

297 $$\begin{aligned} w 2 a\, \mathrm{Re} (T \Phi \Psi h {\overline{\Psi }}) = \Phi \mathrm{Re}\left( w 2 a T \Psi h {\overline{\Psi }}\right) - w2 a h\, \mathrm{Re}\{(T \Psi ) (\Phi {\overline{\Psi }})\} \end{aligned}$$

#### Part IV: $wa^2 \sin ^2\theta TT\Psi$

298 $$\begin{aligned} w a^2 \sin ^2\theta \mathrm{Re}\{ TT\Psi h {\overline{\Psi }}\} = T\, \mathrm{Re}\left( w a^2 \sin ^2\theta T\Psi h{\overline{\Psi }}\right) - w a^2 \sin ^2\theta h |T\Psi |^2 \end{aligned}$$

### The $\Phi$ multipier: $\omega _+\chi \Phi {\overline{\Psi }}$

#### Part I: $\frac{1}{2} \left( L {\underline{L}} + {\underline{L}} L \right) \Psi$

299 $$\begin{aligned}&\frac{1}{2} \left( L {\underline{L}} + {\underline{L}} L \right) \mathrm{Re}\{\Psi \upomega _+\chi \Phi {\overline{\Psi }}\} = \frac{1}{4} \left( L+{\underline{L}}\right) \left( L + {\underline{L}}\right) \mathrm{Re}\{\Psi \upomega _+\chi \Phi {\overline{\Psi }}\} \nonumber \\&\qquad - \frac{1}{4} \left( L-{\underline{L}}\right) \left( L - {\underline{L}}\right) \mathrm{Re}\{\Psi \upomega _+\chi \Phi {\overline{\Psi }}\}\nonumber \\&\quad = \frac{1}{4} \left( L+{\underline{L}}\right) \mathrm{Re}\Big \{ \left( L + {\underline{L}}\right) \Psi \upomega _+\chi \Phi {\overline{\Psi }}\Big \} - \frac{1}{8} \Phi \Big \{ \upomega _+ \chi \left| \left( L + {\underline{L}}\right) \Psi \right| ^2 \Big \} \nonumber \\&\qquad -\frac{1}{4} \left( L-{\underline{L}}\right) \mathrm{Re}\Big \{ \left( L - {\underline{L}}\right) \Psi \upomega _+\chi \Phi \overline{\Psi } \Big \} + \frac{1}{8} \Phi \Big \{ \upomega _+ \chi \left| \left( L - {\underline{L}}\right) \Psi \right| ^2 \Big \} \nonumber \\&\qquad +\frac{1}{2} \upomega _+ \chi ^\prime \mathrm{Re}\left( (L-{\underline{L}})\Psi \right) (\Phi \overline{\Psi }) \end{aligned}$$

#### Part II: (After Integration Over $\int \sin \theta d\theta d\phi$)

300

#### Part III: $w2 a T \Phi \Psi$

301 $$\begin{aligned} w2a\mathrm{Re}\{T\Phi \Psi \upomega _+\chi \Phi {\overline{\Psi }}\} = T \Big \{ wa \upomega _+\chi |\Phi \Psi |^2 \Big \} \end{aligned}$$

#### Part IV: $w a^2 \sin ^2\theta TT\Psi$

302 $$\begin{aligned}&w a^2 \sin ^2\theta \mathrm{Re}\{TT\Psi \upomega _+\chi \Phi {\overline{\Psi }}\} = T \mathrm{Re}\Big \{ w a^2 \sin ^2\theta \upomega _+\chi (T\Psi ) \Phi \overline{\Psi } \Big \} \nonumber \\&\quad - \frac{1}{2} \Phi \Big \{w a^2 \sin ^2\theta \upomega _+\chi |T\Psi |^2 \Big \} \end{aligned}$$

### The *y*-Multiplier: $y (L-\underline{L}){\overline{\Psi }}$

#### Part I: $\frac{1}{2} \left( L {\underline{L}} + {\underline{L}} L \right) \Psi$

303 $$\begin{aligned}&\frac{1}{2} \mathrm{Re}\{\left( L {\underline{L}} + {\underline{L}} L \right) \Psi \left( y (L-{\underline{L}}){\overline{\Psi }}\right) \} = \frac{1}{4} \mathrm{Re}\{\left( L+{\underline{L}}\right) \left( L + {\underline{L}}\right) \Psi \left( y (L-{\underline{L}}){\overline{\Psi }}\right) \}\nonumber \\&\qquad - \frac{1}{4} \mathrm{Re}\{\left( L-{\underline{L}}\right) \left( L - {\underline{L}}\right) \Psi \left( y (L-{\underline{L}}){\overline{\Psi }}\right) \} \nonumber \\&\quad =\frac{1}{4} \mathrm{Re}\left( L+ {\underline{L}}\right) \Big \{ y \left( L + {\underline{L}}\right) \Psi \left( (L-{\underline{L}})\Psi \right) \Big \}-\frac{1}{8} \left( L- {\underline{L}}\right) \Big \{ y \left| (L-{\underline{L}})\Psi \right| ^2 \Big \} \nonumber \\&\qquad -\frac{1}{8}\left( L-{\underline{L}} \right) \Big \{ y \left| (L+{\underline{L}})\Psi \right| ^2 \Big \} + \frac{1}{4} y\, \mathrm{Re}\{\left[ L-{\underline{L}},L+{\underline{L}}\right] \Psi \left( L+{\underline{L}}\right) {\overline{\Psi }}\} \nonumber \\&\qquad +\frac{1}{4} y^\prime \left[ \left| (L+{\underline{L}})\Psi \right| ^2 + \left| (L-{\underline{L}})\Psi \right| ^2 \right] \end{aligned}$$

Using the commutator identity

$$\begin{aligned} \left[ L-{\underline{L}},L+{\underline{L}}\right] = 2 \left[ L,{\underline{L}} \right] = 4 \partial _{r^*} \left( \frac{a}{r^2+a^2}\right) \Phi = - \frac{8r a}{\left( r^2+a^2\right) ^2} \frac{\Delta }{r^2+a^2} \Phi \end{aligned}$$

we conclude

$$\begin{aligned}&\frac{1}{2} \left( L {\underline{L}} + {\underline{L}} L \right) \Psi \left( y (L-{\underline{L}})\Psi \right) =\frac{1}{4} \mathrm{Re}\left( L+ {\underline{L}}\right) \Big \{ y \left( L + {\underline{L}}\right) \Psi \left( (L-{\underline{L}})\Psi \right) \Big \} \\&\quad -\frac{1}{8}\left( L-{\underline{L}} \right) \Big \{ y \left| (L+{\underline{L}})\Psi \right| ^2 +y \left| (L-{\underline{L}})\Psi \right| ^2\Big \}\\&\quad +\frac{1}{4} y^\prime \left[ \left| (L+{\underline{L}})\Psi \right| ^2 + \left| (L-{\underline{L}})\Psi \right| ^2 \right] \\&\quad -2y \frac{r a}{\left( r^2+a^2\right) ^2} \frac{\Delta }{r^2+a^2} \mathrm{Re}\{\Phi \Psi \left( L+{\underline{L}}\right) {\overline{\Psi }}\} \end{aligned}$$

#### Part II: (After Integration Over $\int \sin \theta d\theta d\phi$)

304

#### Part III: $w 2 a T \Phi \Psi$

305 $$\begin{aligned}&w 2 a\, \mathrm{Re}\{T \Phi \Psi \left( y (L-{\underline{L}}){\overline{\Psi }}\right) \} =w a y\, \mathrm{Re}\{\left( L+{\underline{L}} -2\frac{a}{r^2+a^2}\Phi \right) \Phi \Psi \left( (L-{\underline{L}}){\overline{\Psi }}\right) \} \nonumber \\&\quad =-\Phi \mathrm{Re}\Big \{\frac{2a^2}{r^2+a^2} w y (\Phi \Psi ) \left( (L-{\underline{L}}){\overline{\Psi }}\right) \Big \} + (L-{\underline{L}}) \Big \{ \frac{a^2}{r^2+a^2} w y |\Phi \Psi |^2 \Big \} \nonumber \\&\qquad - \left[ (L-{\underline{L}}) \left( \frac{a^2}{r^2+a^2} w y \right) \right] |\Phi \Psi |^2 +\frac{1}{2} \Phi \Big \{ way |L\Psi |^2\Big \} - \frac{1}{2} \Phi \Big \{ way |{\underline{L}}\Psi |^2\Big \} \nonumber \\&\qquad -L\, \mathrm{Re}\Big \{ way \Phi \Psi {\underline{L}} {\overline{\Psi }}\Big \} + {\underline{L}} \mathrm{Re}\Big \{ way \Phi \Psi {L} {\overline{\Psi }}\Big \} -\frac{4ra^2}{(r^2+a^2)^2} \frac{\Delta }{r^2+a^2} w y |\Phi \Psi |^2 \nonumber \\&\qquad + a \left[ L \left( wy\right) \right] \mathrm{Re}\{ (\Phi \Psi )({\underline{L}} {\overline{\Psi }})\} - a \left[ {\underline{L}} \left( wy\right) \right] \mathrm{Re}\{(\Phi \Psi )({L} {\overline{\Psi }})\} \end{aligned}$$

#### Part IV: $wa^2 \sin ^2\theta TT\Psi$

306 $$\begin{aligned}&w a^2 \sin ^2\theta \mathrm{Re}\{TT\Psi \left( y (L-{\underline{L}}){\overline{\Psi }}\right) \} = T\, \mathrm{Re}\Big \{ w a^2 \sin ^2\theta T\Psi \left( y (L-{\underline{L}}){\overline{\Psi }}\right) \Big \}\nonumber \\&\quad - \frac{1}{2} \left( L-{\underline{L}}\right) \Big \{w a^2 \sin ^2\theta y |T\Psi |^2 \Big \} \nonumber \\&\quad +\left( w y\right) ^\prime a^2 \sin ^2\theta | T\Psi |^2 \end{aligned}$$

### The Redshift Multiplier: $\frac{1}{w}\xi \underline{L}\overline{\Psi }$

#### Part I: $\frac{1}{2} \left( L {\underline{L}} + {\underline{L}} L \right) \Psi$

307 $$\begin{aligned}&\frac{1}{2} \text {Re} \left( L {\underline{L}} + {\underline{L}} L \right) \Psi \left( \frac{1}{w} \xi {\underline{L}}\Psi \right) = L {\underline{L}} \Psi \left( \frac{1}{w} \xi {\underline{L}}\Psi \right) - \frac{1}{2} \text {Re} \left( \left[ L,{\underline{L}}\right] \Psi \left( \frac{1}{w} \xi {\underline{L}}\Psi \right) \right) \nonumber \\&\quad =\frac{1}{2} L \left( \frac{1}{w} \xi |{\underline{L}}\Psi |^2\right) -\frac{1}{2} \left( \frac{\xi }{w}\right) ^\prime |{\underline{L}}\Psi |^2 + \frac{2r a \xi }{r^2+a^2} \text {Re} \left( \Phi \Psi {\underline{L}}\Psi \right) \, . \end{aligned}$$

#### Part II: (After Integration Over $\int \sin \theta d\theta d\phi$)

308

#### Part III: $w 2 a T \Phi \Psi$

309 $$\begin{aligned}&w 2 a T \Phi \Psi \left( \frac{1}{w} \xi {\underline{L}}\overline{\Psi }\right) = a \xi \left( L+{\underline{L}} -2\frac{a}{r^2+a^2}\Phi \right) \Phi \Psi {\underline{L}}\overline{\Psi } \nonumber \\&\quad =-\Phi \Big \{ \frac{2a^2}{r^2+a^2} \xi (\Phi \Psi ) {\underline{L}}\overline{\Psi }\Big \} + {\underline{L}} \Big \{ \frac{a^2}{r^2+a^2} \xi |\Phi \Psi |^2 \Big \} \nonumber \\&\qquad - \left[ {\underline{L}} \left( \frac{a^2}{r^2+a^2} \xi \right) \right] |\Phi \Psi |^2 +\frac{1}{2} \Phi \Big \{ a\xi |{\underline{L}}\Psi |^2\Big \} + a \xi L \Phi \Psi {\underline{L}}\overline{\Psi } \, . \end{aligned}$$

In view of

310 $$\begin{aligned}&a\xi L\Phi \Psi {\underline{L}} \overline{\Psi } = L \left( a \xi \Phi \Psi {\underline{L}} \overline{\Psi } \right) - a \xi ^\prime \Phi \Psi {\underline{L}} \overline{\Psi } - a\xi \Phi \Psi \left[ L, {\underline{L}} \right] \overline{\Psi } - a\xi \Phi \Psi {\underline{L}} L\overline{\Psi } \nonumber \\&\quad =L \left( a \xi \Phi \Psi {\underline{L}} \overline{\Psi } \right) - a \xi ^\prime \Phi \Psi {\underline{L}} \overline{\Psi } - a\xi \Phi \Psi \left[ L, {\underline{L}} \right] \overline{\Psi } - {\underline{L}} \left( a \xi \Phi \Psi L \overline{\Psi } \right) \nonumber \\&\qquad - a\xi ^\prime \Phi \Psi L\overline{\Psi } + a\xi \Phi {\underline{L}}\Psi L \overline{\Psi } \end{aligned}$$

and hence

$$\begin{aligned}&2 \text {Re} \left( a\xi L\Phi \Psi {\underline{L}} \overline{\Psi }\right) = L \left( a \xi \text {Re} \left( \Phi \Psi {\underline{L}} \overline{\Psi }\right) \right) - a \xi ^\prime \text {Re} \left( \Phi \Psi \left( {\underline{L}} + L\right) \overline{\Psi }\right) \\&\quad - a\xi \text {Re} \left( \Phi \Psi \left[ L, {\underline{L}} \right] \overline{\Psi }\right) \\&\quad - {\underline{L}} \left( a \xi \text {Re} \left( \Phi \Psi L\overline{\Psi }\right) \right) + \Phi \left( a \xi \text {Re} \left( {\underline{L}}\Psi L \overline{\Psi } \right) \right) \end{aligned}$$

we conclude from (309)

311 $$\begin{aligned}&\text {Re} \left( w 2 a T \Phi \Psi \left( \frac{1}{w} \xi {\underline{L}}\overline{\Psi }\right) \right) \nonumber \\&\quad =\Phi \Big \{-\frac{2a^2}{r^2+a^2} \xi \text {Re} \left( \Phi \Psi {\underline{L}}\overline{\Psi } \right) + \frac{1}{2} a \xi \text {Re} \left( {\underline{L}}\Psi L \overline{\Psi } \right) +\frac{1}{2} a\xi |{\underline{L}}\Psi |^2\Big \}\nonumber \\&\qquad + {\underline{L}} \Big \{ \frac{a^2}{r^2+a^2} \xi |\Phi \Psi |^2 - \frac{1}{2}a\xi \text {Re} \left( \Phi \Psi L\overline{\Psi } \right) \Big \} +L \left( \frac{1}{2} a \xi \text {Re} \left( \Phi \Psi {\underline{L}} \overline{\Psi } \right) \right) \nonumber \\&\qquad - \left[ {\underline{L}} \left( \frac{a^2}{r^2+a^2} \xi \right) \right] |\Phi \Psi |^2- \frac{1}{2} a \xi ^\prime \text {Re} \left( \Phi \Psi \left( {\underline{L}} + L\right) \overline{\Psi }\right) - \frac{1}{2} a\xi \text {Re} \left( \Phi \Psi \left[ L, {\underline{L}} \right] \overline{\Psi }\right) \, . \end{aligned}$$

#### Part IV: $wa^2 \sin ^2\theta TT\Psi$

312 $$\begin{aligned}&\text {Re} \left( w a^2 \sin ^2\theta TT\Psi \left( \frac{1}{w} \xi {\underline{L}}\overline{\Psi }\right) \right) = T \Big \{ \xi a^2 \sin ^2\theta \text {Re} \left( T\Psi {\underline{L}}\overline{\Psi }\right) \Big \} \nonumber \\&\quad - \frac{1}{2} {\underline{L}} \Big \{\xi a^2 \sin ^2\theta |T\Psi |^2 \Big \} \nonumber \\&\quad -\frac{1}{2} \xi ^\prime a^2 \sin ^2\theta | T\Psi |^2 \, . \end{aligned}$$

### The $r^p$ Multiplier: $r^p \beta _k \xi L\overline{\Psi }$ with $\beta _k=1+k\frac{M}{r}$

#### Part I: $\frac{1}{2} \left( L {\underline{L}} + {\underline{L}} L \right) \Psi$

313 $$\begin{aligned}&\text {Re} \left( \frac{1}{2} \left( L {\underline{L}} + {\underline{L}} L \right) \Psi \left( r^p\beta _k\xi L\overline{\Psi }\right) \right) = \text {Re} \left( {\underline{L}} L\Psi \left( r^p\beta _k \xi L\overline{\Psi }\right) \right) \nonumber \\&\qquad + \text {Re} \left( \frac{1}{2} \left[ L,{\underline{L}}\right] \Psi \left( r^p \beta _k\xi L\overline{\Psi }\right) \right) \nonumber \\&\quad =\frac{1}{2} {\underline{L}} \left( \xi r^p \beta _k |{L}\Psi |^2\right) +\frac{1}{2} \left( \xi \left( pr^{p-1} + {\mathcal {O}}\left( r^{p-2}\right) \right) + \xi ^\prime r^p \beta _k \right) |{L}\Psi |^2 \nonumber \\&\qquad - \frac{2r a \xi r^p \beta _kw}{r^2+a^2} \text {Re} \left( \Phi \Psi {L}\overline{\Psi }\right) \end{aligned}$$

#### Part II: (After Integration Over $\int \sin \theta d\theta d\phi$)

314

#### Part III: $w 2 a T \Phi \Psi$

315 $$\begin{aligned}&\text {Re} \left( w 2 a T \Phi \Psi \left( r^p \beta _k \xi {L}\overline{\Psi }\right) \right) = \text {Re} \left( a \xi w r^p \beta _k \left( L+{\underline{L}} -2\frac{a}{r^2+a^2}\Phi \right) \Phi \Psi {L}\overline{\Psi }\right) \nonumber \\&\quad =-\Phi \Big \{\text {Re} \left( \frac{2a^2}{r^2+a^2} \xi w r^p \beta _k (\Phi \Psi ) L\overline{\Psi } \right) \Big \} + {L} \Big \{ \frac{a^2}{r^2+a^2} \xi w r^p \beta _k |\Phi \Psi |^2 \Big \} \nonumber \\&\qquad - \left( \frac{a^2}{r^2+a^2} \xi w r^p \beta _k \right) ^\prime |\Phi \Psi |^2 +\frac{1}{2} \Phi \Big \{ a\xi w r^p \beta _k |L\Psi |^2\Big \} + \text {Re} \left( a \xi w r^p \beta _k {\underline{L}} \Phi \Psi {L}\overline{\Psi }\right) \, . \end{aligned}$$

In view of

$$\begin{aligned}&a\xi w r^p \beta _k {\underline{L}}\Phi \Psi {L} \overline{\Psi } = {\underline{L}} \left( a \xi w r^p \beta _k \Phi \Psi {L} \overline{\Psi } \right) + \left( a \xi w r^p \beta _k\right) ^\prime \Phi \Psi {L} \overline{\Psi } \\&\qquad + a\xi w r^p \beta _k \Phi \Psi \left[ L, {\underline{L}} \right] \overline{\Psi } - a\xi w r^p \beta _k \Phi \Psi L{\underline{L}}\overline{\Psi } \\&\quad = {\underline{L}} \left( a \xi w r^p \beta _k \Phi \Psi {L} \overline{\Psi } \right) + \left( a \xi w r^p \beta _k\right) ^\prime \Phi \Psi {L} \overline{\Psi } + a\xi w r^p \beta _k \Phi \Psi \left[ L, {\underline{L}} \right] \overline{\Psi } \\&\qquad - {L} \left( a \xi w r^p \beta _k \Phi \Psi {\underline{L}}\overline{\Psi } \right) + \left( a\xi w r^p \beta _k\right) ^\prime \Phi \Psi {\underline{L}}\overline{\Psi }\\&\qquad + \Phi \left( a\xi w r^p \beta _k L\Psi {\underline{L}} \overline{\Psi } \right) - a\xi w r^p \beta _k L \Psi {\underline{L}} \Phi \overline{\Psi } \end{aligned}$$

we conclude from (315)

$$\begin{aligned}&\text {Re} \left( w 2 a T \Phi \Psi \left( r^p \beta _k \xi {L}\overline{\Psi }\right) \right) =\Phi \Big \{-\text {Re} \left( \frac{2a^2 w r^p}{r^2+a^2} \xi \beta _k (\Phi \Psi ) L\overline{\Psi } \right) + \frac{1}{2} a\xi w r^p \beta _k |L\Psi |^2 \\&\quad + \frac{1}{2} \text {Re}\left( a\xi w r^p \beta _k L\Psi {\underline{L}} \overline{\Psi }\right) \Big \}\\&\quad + {L} \Big \{ \frac{a^2 w r^p}{r^2+a^2} \xi \beta _k |\Phi \Psi |^2 -\frac{1}{2} \text {Re} \left( a \xi w r^p \beta _k \Phi \Psi {\underline{L}}\overline{\Psi }\right) \Big \} \\&\quad + {\underline{L}} \Big \{\frac{1}{2} \text {Re} \left( a \xi w r^p \beta _k \Phi \Psi {L} \overline{\Psi } \right) \Big \} \\&\quad - \left( \frac{a^2}{r^2+a^2} \xi w r^p \beta _k \right) ^\prime |\Phi \Psi |^2 + \frac{1}{2} \text {Re} \left( \left( a \xi w r^p \beta _k\right) ^\prime \Phi \Psi \left( {L} + {\underline{L}}\right) \overline{\Psi } \right) \\&\quad + \frac{1}{2} \text {Re} \left( a\xi w r^p \beta _k \Phi \Psi \left[ L, {\underline{L}} \right] \overline{\Psi }\right) \, . \end{aligned}$$

#### Part IV: $wa^2 \sin ^2\theta TT\Psi$

316 $$\begin{aligned}&\text {Re} \left( w a^2 \sin ^2\theta TT\Psi \left( r^p\beta _k \xi {L}\overline{\Psi }\right) \right) = T \Big \{ w a^2 \sin ^2\theta r^p \beta _k \xi \text {Re} \left( T\Psi {L}\overline{\Psi }\right) \Big \}\nonumber \\&\quad - \frac{1}{2} {L} \Big \{w a^2 \sin ^2\theta \xi r^p\beta _k |T\Psi |^2 \Big \} \nonumber \\&\quad +\frac{1}{2} \left( \xi \left( w r^p \beta _k\right) ^\prime + \xi ^\prime w r^p \beta _k \right) a^2 \sin ^2\theta | T\Psi |^2 \, . \end{aligned}$$

## Footnotes

1 In the non-trapping case, such estimates are non-degenerate and can be derived by classical virial identities whose use goes back to [86].

2 Unlike in [31] the definitions are not entirely symmetric for $s=\pm \, 2$. This is because the null vectors and overall radial weights defining the $\alpha ^{[\pm 2]}$ scale differently from the null vectors defining the tensorial $\alpha$ and $\underline{\alpha }$ in [31]. See Sect. 2.4.

3 See Sects. 2.4 and 3.3 for the precise relation between the tensorial Regge–Wheeler equation defined in [31] and equation (7). Note in particular that $\Psi ^{[+2]}$ and $\overline{\Psi ^{[-2]}}$ satisfy the same equation, which explains the appearance of a single Regge–Wheeler equation in [31]. We also note that both the operators  and  have non-negative eigenvalues. See Sects. 6.2.1 and 6.2.2.

4 In contrast, good quantitative decay rates for solutions the Bianchi equations on pure AdS with dissipative boundary conditions have been proven in [61], suggesting nonlinear stability.

5 Euler coordinates cover $S^3$ everywhere except the north and southpole at $\theta =0$ and $\theta =\pi$ respectively. The ranges of the coordinates are $0<\theta <\pi$, $0\le \phi < 2\pi$ and $0\le \rho < 4\pi$.

6 In fact, the right hand side vanishes for the first spin-weighted spherical harmonics.

7 Recall that the Riemann tensor agrees with the Weyl tensor for a Ricci flat metric.

8 Note that in this paper $\Psi ^{[+2]} = r^3 P^{[+2]}$ for $a=0$ so when relating orthonormal components of the tensor *P* and the complex function $P^{[2]}$ there is an additional factor of $r^2$. This factor disappears when replacing the orthonormal frame on the spheres of symmetry with an orthonormal frame on the *unit* sphere to express the components of *P*.

9 Note that in contrast to the $\left[ +2\right]$-energies, no *p*-weights appear. The underlying reason is that the transport estimates for $\uppsi ^{[-2]}$ and $\upalpha ^{[-2]}$ will always be applied with the same *r*-weight. Note also in this context that the ${\mathbb {E}}$-energies for $\upalpha ^{[-2]}$ and $\uppsi ^{[-2]}$ on the slices $\widetilde{\Sigma }_{\tau }$ in (81)–(82) carry the same *r*-weight as the corresponding spacetime ${\mathbb {I}}$-energies in (85)–(86). This arises from the fact that the transport for the $[-2]$-quantities happens in the *L*-direction and the relation (24) between *L* and the unit normal to the slices $\widetilde{\Sigma }_{\tau }$.

10 The prolate case corresponds to the $\xi$ being purely imaginary.

11 We note that this renormalisation is slightly different from [68].

12 This will not be a source of confusion because we will never apply frequency analysis directly to $\upalpha ^{[\pm 2]}$.
